# Dimerization of Allylbenzenes into Cyclolignans by a Metathesis-Oxidation Sequence

**DOI:** 10.1021/acs.joc.5c02949

**Published:** 2026-02-26

**Authors:** Kiryl Vasiutovich, Alexander A. Fadeev, Peter Čambal, Eliška Matoušová

**Affiliations:** † Department of Organic Chemistry, Faculty of Science, Charles University, Hlavova 8, 128 00 Praha 2, Czech Republic; ‡ Department of Analytical Chemistry, Faculty of Science, Charles University, Hlavova 8, 128 00 Praha 2, Czech Republic

## Abstract

Allylbenzenes form dimeric cyclolignans in a two-step, one-pot sequence involving Ru-catalyzed olefin metathesis followed by iron- or acid-catalyzed oxidation with DDQ. Alternatively, performing the oxidation step under wet conditions leads to benzyl styryl ketones as the major products. The mechanistic investigation suggests that the oxidation process relies on the combination of single electron transfer, hydrogen atom transfer, and proton transfer steps. The developed metathesis-oxidation sequence enables a straightforward and scalable preparation of arylnaphthalene and aryltetralin types of cyclolignans, such as oleralignan B and its derivatives.

## Graphical abstract

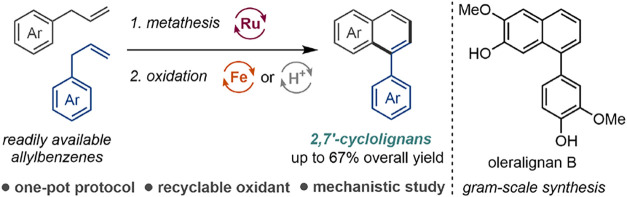

## Introduction

Lignans represent a large family of natural bioactive small molecules of phenolic origin that are found in numerous plant and animal sources.^1–5^ Although the first known lignans have been recognized as a family since 1936,^6^ new members are still being discovered. Lignans are considered to play a vital role in plant defense by expressing their antimicrobial, antifungal, antiviral, antioxidant, and insecticidal activities.^1–5,7–9^ At the same time, these properties have invigorated the research aiming at the preparation of lignans^10–13^ and their use as pharmaceuticals^14–17^ and food chemicals.^5,7^

Cyclolignans, such as arylnaphthalenes and aryltetralins, are among the most prominent types of bioactive lignans (Scheme 1a).^11,12,14,18,19^ For example, anti-inflammatory arylnaphthalenes oleralignan B and alashinol D have been recently isolated from *Portulaca oleracea* ^20^ and *Syringa pinnatifolia*,^21^ respectively. Arylnaphthalene lactones, such as justicidin B and taiwanin C, have been found in several plants over the decades and are known in particular for their activity against cancer cell lines.^19,22^ Podophyllotoxin and analogous aryltetralins from *Podophyllum* species exhibit a wide spectrum of biological activities and have been tailored into semisynthetic *O*-glycoside derivatives etoposide and teniposide for cancer treatment.^16,17^

**Scheme 1:**
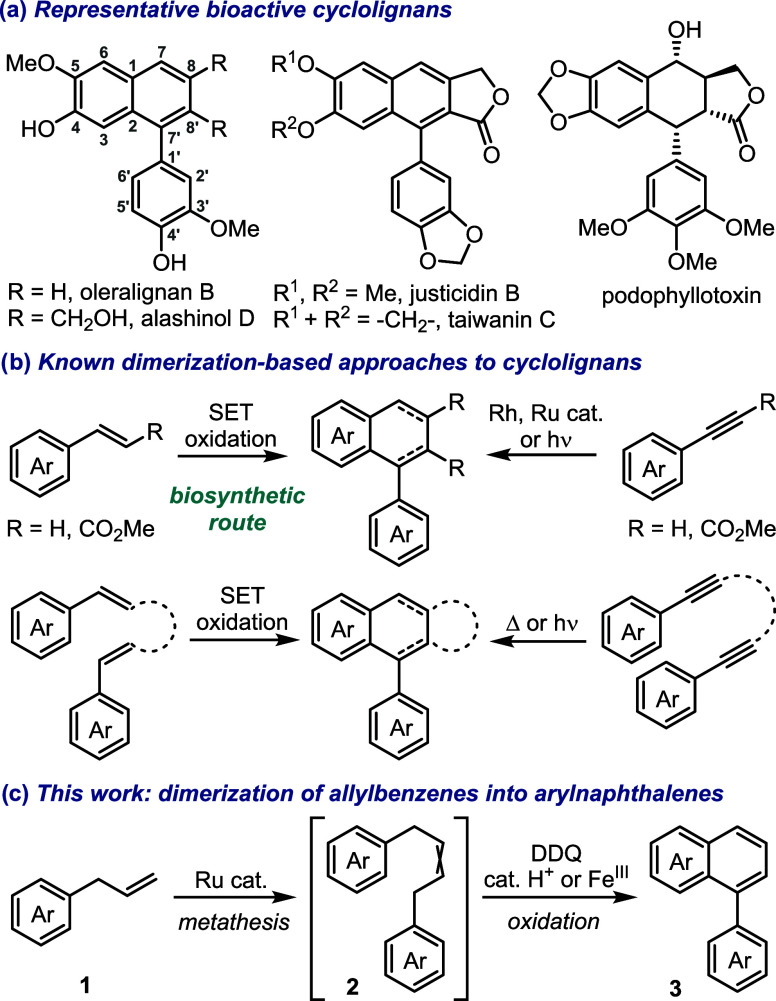
Cyclolignans and Their Synthesis from the Dimerization Precursors (Lignan Numbering is Shown)

Biosynthetically, the inherently dimeric structures of lignans originate from the dimerization of phenylpropanoid units.^23^ This fact makes dimerization an effective strategy for the synthesis of lignans from a single building block, and cyclolignans are not an exception. Thus, chemical dimerization of styrenes resembles the biosynthetic dimerization of hydroxycinnamic acid derivatives^10,24^ and is typically initiated by one-electron oxidation to afford aryltetralins,^25–27^ aryltetralones,^28–32^ aryldihydronaphthalenes,^31,33–35^ and arylnaphthalenes,^32,36–38^ depending on the reaction conditions (Scheme 1b). The related dimerization of phenylacetylenes into arylnaphthalenes was first demonstrated by the thermal condensation of arylpropiolic acids^39–41^ and later extended in scope by the means of transition metal catalysis^42–44^ and the photodehydro-Diels–Alder reaction.^45–47^ However, controlling the chemo- and regioselectivity of the intermolecular dimerizations presents a challenge, and product mixtures are often formed. Tethering the reacting molecules *in situ* ^39–41^ or as a separate reaction step^46–54^ markedly improves the effectiveness and the selectivity of the dimerization process. Nevertheless, the tethering approaches rely on specific functional groups in the reactants and create an additional ring that may not be desired in the final product. Besides that, the availability of the suitably substituted styrenes and phenylacetylenes is limited, and only a few can be obtained from renewable resources.

Conversely, the preparation of lignans by dimerization of allylbenzenes remains documented only by a handful of examples that do not include the synthesis of cyclolignans.^55–57^ Nonetheless, the increasing availability of allylbenzenes from renewable materials, including lignin and essential oils, makes these arenes attractive building blocks for organic synthesis. We suggested that dimerization of allylbenzenes **1** into 1,4-diaryl-2-butenes **2** by olefin metathesis could serve as a traceless tether approach enabling the subsequent oxidative annulation of **2** into arylnaphthalene lignans **3**, such as oleralignan B and its congeners (Scheme 1c). At the same time, we expected that the strain induced by the double bond in **2** would prevent the 8-membered ring formation observed earlier in the oxidation of electron-rich 1,4-diarylbutanes.^58–60^ Notably, alkenes **2** are common byproducts in the synthesis of other valuable compounds via olefin cross-metathesis,^61–63^ and harnessing the synthetic potential of these alkenes could reduce the waste generated with implementing such protocols. Lastly, the synthetic sequence described herein is scalable and shorter than most of the existing routes that do not rely on dimerization to access analogous cyclolignans.^10–13,60,64–73^

## Results and Discussion

Knowing that one-electron oxidants, such as DDQ (2,3-dichloro-5,6-dicyano-1,4-benzoquinone) and iron­(III) chloride, are particularly effective in annulation reactions,^60,72,73^ we tested these reagents in the oxidation of *E*-alkene **2a**, which was in turn obtained by the self-metathesis of methyleugenol using Grubbs I catalyst. Gratifyingly, treating *E*-(**2a**) with 3 equiv of DDQ in dichloromethane afforded the desired annulation product **3a** in 40% yield at 40 °C and in 34% yield at 25 °C (Table 1). Chloranil was considerably less effective than DDQ and gave only 6% of **3a**. Likewise, a rapid formation of **3a** was observed at 25 °C with FeCl_3_ as the sole oxidant (15% yield), with FeCl_3_ and MnO_2_ as the co-oxidant (37% yield), and using the FeCl_3_-DDQ system (39% yield). Next, we screened the performance of organic acids as additives. The oxidation of **2a** with DDQ in the presence of 10 mol % of trifluoroacetic or *p*-toluenesulfonic acid improved the yield of **3a** only to 42% and 46%, respectively. In contrast, the addition of methanesulfonic acid led to a nearly 2-fold increase in the product yield (74%), even when the amount of DDQ was reduced to 2.5 equiv. However, lowering the amount of DDQ to 2 equiv or the amount of acid to 5 mol % decreased the yield to 60% and 70%, respectively. The attempted use of DDQ in a catalytic amount with MnO_2_ as the terminal oxidant gave only a trace amount of **3a**.

**Table 1:**
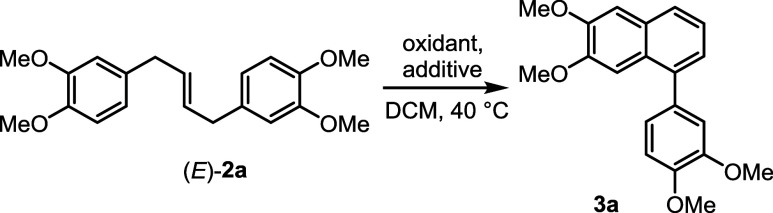
Screening of the Aromatization Conditions^a^

entry	oxidant (equiv)	additive (mol %)	yield of **3a**, %^a^
1	DDQ (3)		40 (34)^b^
2	chloranil (3)		6
3	FeCl_3_ (3)		15^b^ ^,^ ^c^
4	FeCl_3_ (3), MnO_2_ (6)		37^b^ ^,^ ^c^
5	FeCl_3_ (0.15), DDQ (3)		39^b^ ^,^ ^c^
6	DDQ (3)	TFA (10)	42
7	DDQ (3)	*p*-TsOH (10)	46
8	DDQ (3)	MsOH (10)	74
**9**	**DDQ (2**.**5)**	**MsOH (10)**	**74 (73)** ^c^
10	DDQ (2)	MsOH (10)	60
11	DDQ (2.5)	MsOH (5)	70
12	DDQ (0.1), MnO_2_ (6)	MsOH (10)	6

a The reactions were performed on 0.05 mmol scale, the yields were measured by ^1^H NMR at full conversion of (*E*)-**2a** (see Supporting Information for details)

b At room temperature (25 °C)

c Isolated yield.

With the optimized conditions of the annulation reaction in hand, we paired the metathesis with the annulation in the one-pot process and explored the applicability of this sequence. Thus, subjecting methyleugenol to metathesis using a Grubbs I catalyst and treating the reaction mixture with DDQ and MsOH afforded lignan **3a** in 67% overall yield (Scheme 2). Likewise, natural eugenol analogs such as ethyleugenol, safrole, and prenyleugenol provided lignans **3b**–**3d** in 66%, 41%, and 42% yields, respectively. Products **3e** and **3f** were obtained in similar yields from silylated eugenol **1e** (44%) and chloroacetate derivative **1f** (43%). Presumably, the yields of **3c**–**3f** were affected by the cleavage of the aryl ethers during the annulation step, leading to decomposition. Nonetheless, unsubstituted eugenol permitted the first synthesis of oleralignan B (**3g**), although in only 12% yield (the improved synthesis is described in Scheme 6).

**Scheme 2:**
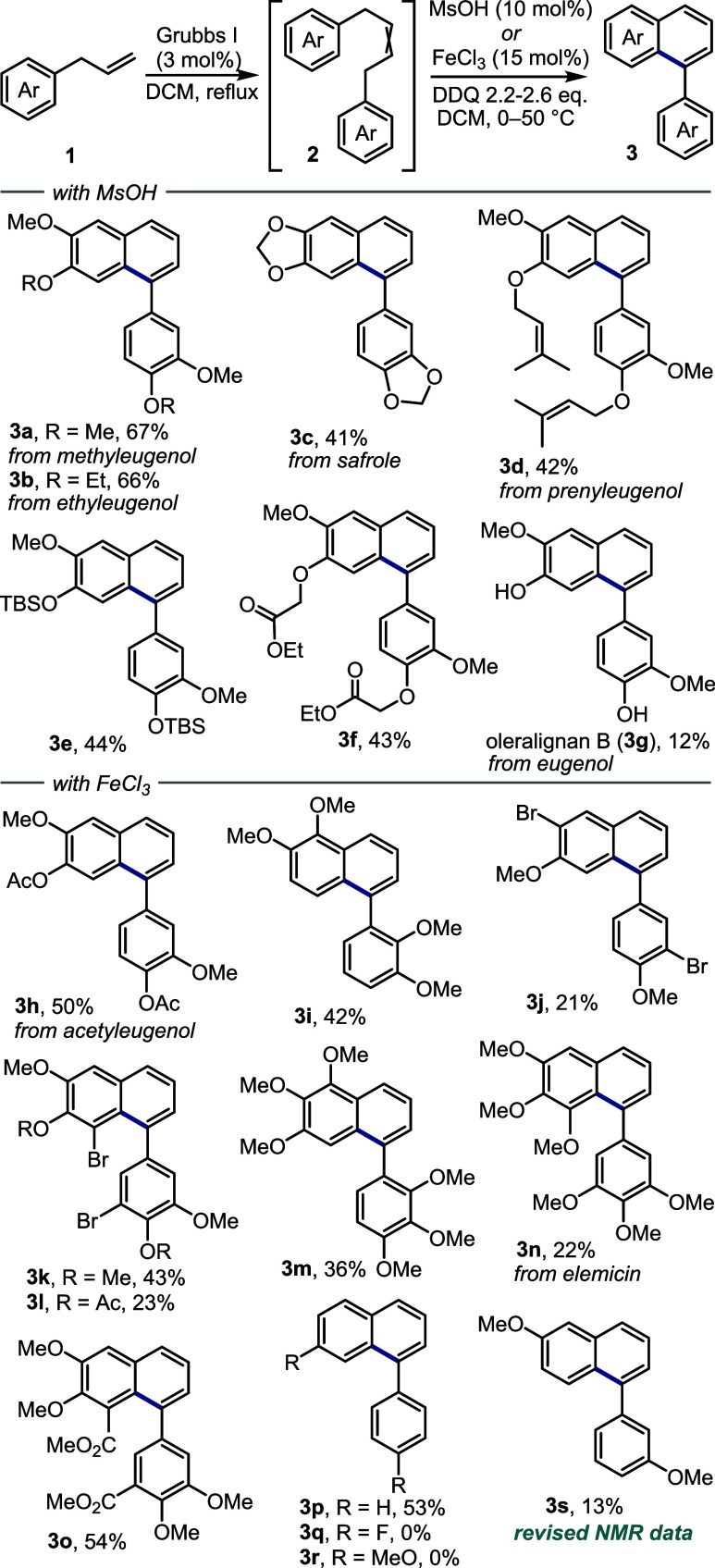
Scope of the Lignans Prepared by the One-Pot Metathesis-Oxidation Sequence (Overall Isolated Yields are Given)

However, when we tried to apply the protocol to alkenes of different substitution character, such as acetyleugenol, allylbenzene, and 2,3-dimethoxyallylbenzene, the desired products were not detected after the annulation step. Complex product mixtures were formed, in which adducts **5** (*vide infra*) were observed by ^1^H and ^13^C NMR. In these cases, extending the reaction time and introducing more methanesulfonic acid led to only the decomposition of **5**. Nevertheless, using the FeCl_3_-DDQ system for annulation gave better results. Thus, acetyleugenol afforded a diacetate derivative of oleralignan B (**3h**) in 50% yield. Next, 2,3-dimethoxyallylbenzene yielded 42% of contiguously substituted arylnaphthalene **3i**. The cyclization of intermediate **2j** arising from 3-bromo-4-methoxyallylbenzene gave regioselectively the product of *para* attack with respect to the position of bromine, albeit in low yield (**3j**, 21%). At the same time, brominated eugenol derivatives **1k** and **1l** provided the cyclization products **3k** (43% yield) and **3l** (23% yield) resulting from the *ortho* attack with respect to the bromine atom, evidently due to the activation of this position by the *para*-methoxy group. Electronically rich 2,3,4-trimethoxyallylbenzene and 3,4,5-trimethoxyallylbenzene (elemicin) furnished lignans **3m** and **3n** in 36% and 22% yields, respectively. Replacing the methoxy group in position 3 of elemicin with a methoxycarbonyl group more than doubled the yield of the product **3o** (54%). Electronically neutral allylbenzene gave 1-phenylnaphthalene (**3p**) in 53% yield. Surprisingly, *para*-substituted allylbenzenes **1q** and **1r** did not give the desired products neither in the presence of MsOH nor FeCl_3_. Presumably, the positive mesomeric effect of the individual *para*-methoxy and *para*-fluoro substituents induces an electronic mismatch that hampers the cyclization step. Lastly, *meta*-methoxyallylbenzene afforded lignan **3s** (13% yield), the NMR data of which were revised to resolve the inconsistencies in the previous reports describing its preparation.^37,42,43^

To uncover whether unsymmetrical alkenes produced by cross-metathesis could selectively undergo the annulation reaction, we prepared alkenes **2rs** and **2ar** and subjected them to the standard acid-catalyzed annulation protocol. As shown in Scheme 3, in both cases, the cyclization occurred regioselectively at the free *para* position to the methoxy group, giving rise to arenes **3rs** (58%) and **3ar** (36%).

**Scheme 3:**
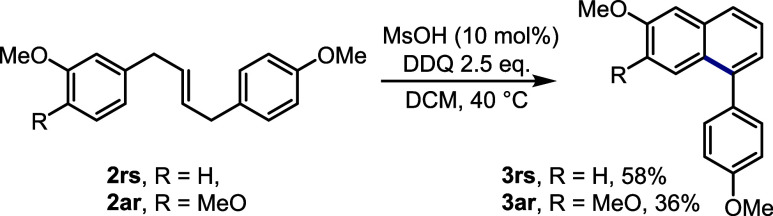
Annulation of the Unsymmetrical Alkenes Obtained by the Cross-Metathesis

The inability of metathesis products **2q** and **2r** to cyclize motivated us to investigate the intermolecular reactivity of the generated intermediates. Aiming to trap the presumably cationic species with water, we performed the annulation step with DDQ in the presence of wet silica gel without any other additives.^74,75^ As shown in Scheme 4, when **2a** was generated by the metathesis of **1a** and subjected to oxidation under these conditions, benzyl styryl ketone **4a** was isolated in 20% yield together with lignan **3a** (33% yield). As expected, higher yields of enones **4** were observed starting from alkenes bearing less electron-rich aromatic rings, and only trace amounts of lignans **3** were detected. Thus, enones **4h** and **4p**–**4r** were obtained in 35–38% yields exclusively as the *trans*-isomers (no other isolable products were attained). The yield of **4r** was increased in the presence of iron trichloride as an additive, albeit only to 41%.

**Scheme 4:**
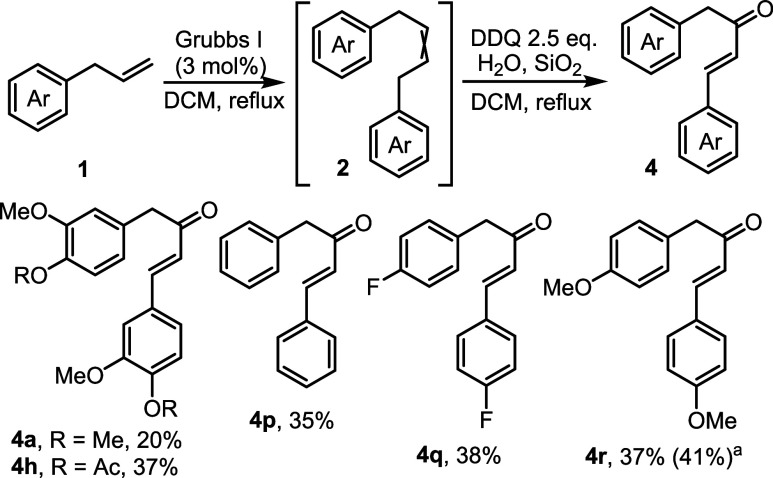
One-Pot Synthesis of Benzyl Styryl Ketones from Allylbenzenes

a In the presence of FeCl_3_·6H_2_O (15 mol %).

The mechanism of the annulation process was then probed by studying the possible reaction intermediates and cyclic voltammetry (CV) profiles. Thus, when the reaction of (*E*)-**2a** with 2 equiv of DDQ was stopped after 2 h, **3a** was obtained in 18% yield together with Diels–Alder adduct **5a** as the major reaction product isolated in 37% yield (Scheme 5a). The subsequent treatment of **5a** with DDQ under the same conditions provided **3a** in a 24% yield. Similarly, methanesulfonic acid catalyzed the cleavage of **5a** leading to *trans*-*trans*-diene **6a** (26%) and **3a** (8%). Although both **5a** and **6a** were also detected during the oxidation of (*E*)-**2a** with DDQ in the presence of MsOH, no isolable intermediates were found when FeCl_3_ was used as the additive. Treating (*E*)-**2a** in DCM with 10 mol % MsOH at 40 °C had no effect. To investigate the possibility of the double bond migration prior the cyclization, isomeric alkene **7a** (ficusnotin D) was prepared by Ru-catalyzed isomerization of (*E*)-**2a** ^76^ and subjected to oxidation with DDQ. Diene **6a** was not detected during the reaction course, and **3a** was the only observed product, albeit in only 10% yield. Finally, the CV profiles of (*E*)-**2a** and **3a** showed that both compounds are irreversibly oxidized at potentials slightly above 1.0 V vs. Ag/Ag^+^ (Scheme 5b; see Supporting Information for details). The small difference in the oxidation potentials of these molecules indicates that further oxidation reactions may occur after the annulation step, negatively affecting the product yield. The one-electron reduction potential of Fe^III^/Fe^II^ (−0.25 V vs. Ag/Ag^+^) is considerably lower than that of DDQ/DDQ^●–^ (0.22 V vs. Ag/Ag^+^), suggesting that DDQ likely initiates the oxidation also when FeCl_3_ is present in the mixture.

**Scheme 5:**
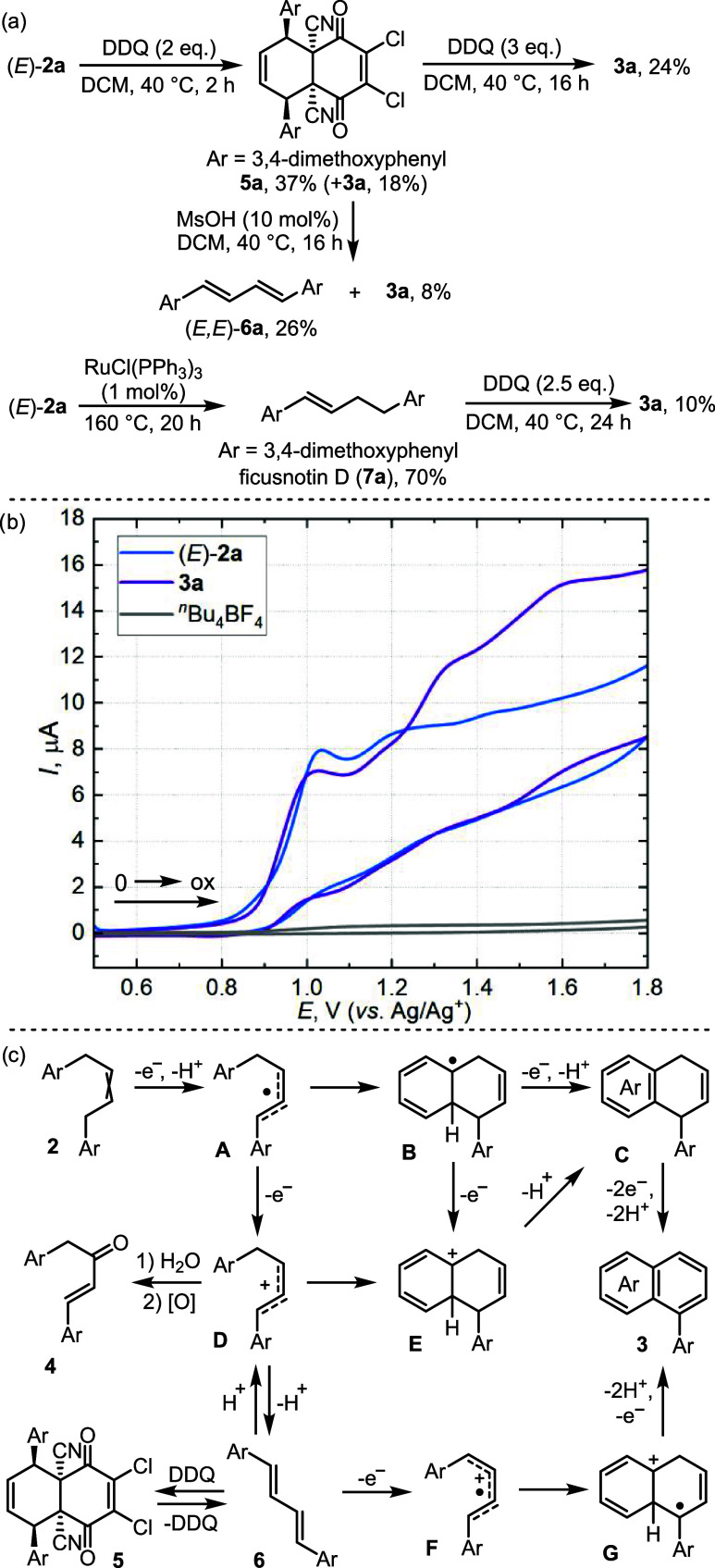
Control Experiments (a), CV Profiles of Compounds (*E*)-**2a** and **3a** (b), and the Proposed Reaction Mechanism (c)

Based on the above experimental observations and the literature data, we propose the mechanistic pathways of the oxidative annulation, as shown in Scheme 5c. Initially, abstraction of a hydrogen atom from the benzylic position of alkene **2** by DDQ leads to the delocalized radical **A**. In the case of alkenes **2** equipped with electron-rich aromatic rings (e.g. **2a**, **2m**, **2n**), single electron oxidation followed by deprotonation may be the preferred pathway to this intermediate. After that, if a radical-accepting aromatic ring is present in **A**, the direct radical cyclization could form intermediate **B**, and the ensuing hydrogen abstraction steps would consecutively give 1,4-dihydronaphthalene **C** and the final aromatic product **3**.^73^ Alternatively, single electron oxidation of radical **A** to cation **D** paves the way for the ionic reactivity.^75,77^ First, cation **D** could undergo Friedel–Crafts-type cyclization leading to product **3** through intermediates **E** and **C**. Second, elimination of a proton from cation **D** leads to diene **6** that can react with DDQ to form Diels–Alder adduct **5** or undergo oxidation into **3**, possibly through delocalized radical-cation intermediates **F** and **G**. Third, hydration of **D** followed by oxidation of the resulting alcohol leads to thermodynamically favorable *trans*-enone **4**.^74,75^

Overall, the mechanism of the oxidative annulation of **2** with DDQ depends on the substitution pattern of the aromatic rings and the additive used. The acid additive does not affect alkene **2**, but it catalyzes the retro-Diels–Alder cleavage of adduct **5**, reversibly protonates diene **6**, and may also promote shifts and isomerizations of the double bonds in the reaction intermediates. In contrast, FeCl_3_ alone can convert **2** into **3**, yet using it as the stoichiometric oxidant dramatically decreases the yield of **3**, likely due to unwanted intermolecular Scholl reactions.^78^ Presumably, the catalytic amount of FeCl_3_ facilitates the cyclization step. Besides that, FeCl_3_ can act as a source of hydrochloric acid.

To demonstrate the applicability of the developed protocol, we improved and upscaled the synthesis of bioactive natural product oleralignan B (Scheme 6). Starting from inexpensive acetyleugenol (**1h**), we avoided exposing the labile phenolic rings to the oxidative conditions of the annulation process. Thus, self-metathesis of **1h** on a gram scale showed full conversion even with the loading of the Grubbs I catalyst reduced to 1 mol %. Engaging the formed alkene **2h** directly into the oxidation step using the FeCl_3_-DDQ system gave diacetate **3h** in 43% yield, and 84% of the reduced DDQ was recovered (the yield of **3h** on this scale was likely lowered by the additional extraction step during workup). Finally, base-mediated hydrolysis of **3h** provided oleralignan B in 95% yield (1.02 g, 40% overall from **1h**).

**Scheme 6:**
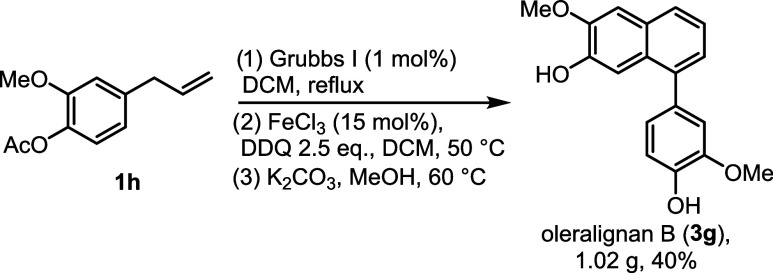
Gram-Scale Preparation of Oleralignan B from Acetyleugenol

Additionally, using **3a** as a stable model substrate, we explored the options for a selective postfunctionalization of the oleralignan B core with substituents that are difficult to introduce through the annulation process. First, selective bromination was achieved by controlling the reaction temperature and the amount of *N*-bromosuccinimide (NBS) used as the bromine source, allowing to prepare either bromoarene **8** in 70% yield or dibromoarene **9** in 91% yield (Scheme 7). However, nitration was more difficult to control; therefore, only 7,6′-dinitroderivative **10** was obtained in 58% yield. Phosphorylation with triethyl phosphite under photoredox conditions^79^ exhibited monoselectivity toward position 7 leading to phosphonate **11**, albeit only in 44% yield due to an incomplete conversion of **3a**. In contrast to the reactions with heteroatom-based electrophiles, Friedel–Crafts monoalkylation with *tert*-butanol in the acidic medium resulted in the substitution of position 8 and furnished alkylarene **12** in 89% yield. Finally, ring-selective Birch reduction^80^ afforded aryltetralin **13** in 73% yield. Overall, the reactivity of lignan **3a** follows the general aromatic substitution patterns, with a high preference for the attack on the central aromatic ring.

**Scheme 7:**
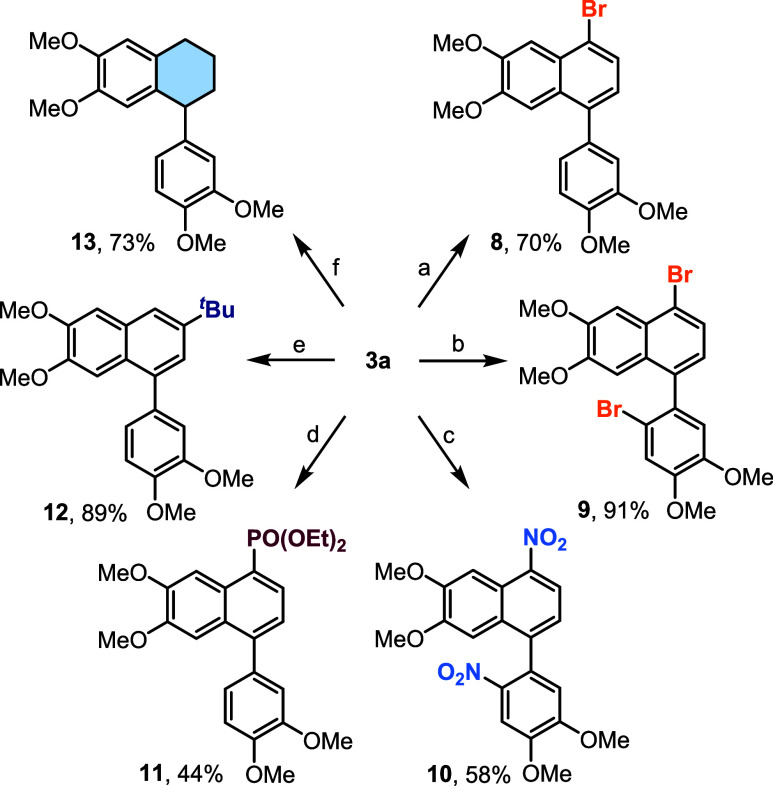
Synthetic Modification of Lignan **3a** ^a^

a (a) NBS (1.1 equiv), HBr (5 mol %), DCM, 0 °C; (b) NBS (2.2 equiv), HBr (5 mol %), DCM, RT; (c) HNO_3_ (4 equiv), AcOH, RT; (d) P­(OEt)_3_ (5 equiv), Ru­(bpy)_3_Cl_2_ (5 mol %), (NH_4_)_2_S_2_O_8_ (2.2 equiv), blue LEDs (470 nm), MeCN, RT; (e) ^*t*^BuOH, HBr (10 mol %), HFIP, RT; (f) Na, EtOH, reflux.

## Conclusions

In summary, we found that both symmetrical and unsymmetrical 1,4-diaryl-2-butenes undergo oxidation into arylnaphthalenes with DDQ in the presence of catalytic amounts of methanesulfonic acid or iron­(III) chloride. At the same time, oxidation of such alkenes with DDQ in the presence of water and silica gel leads preferentially to benzyl styryl ketones. Although modest yields are typical for these transformations, the reactions can be performed in a one-pot manner directly after preparation of the starting alkenes by the olefin metathesis of abundant allylbenzenes. By relying on this reaction sequence, the first synthesis of oleralignan B was accomplished on a gram scale. Additionally, we demonstrated that the oleralignan B core allows for a selective synthetic modification, including bromination, nitration, phosphorylation, alkylation, and reduction.

## Safety Statement

**Caution**! DDQ can be decomposed in the presence of water and potentially release highly toxic hydrogen cyanide. Reactions involving large amounts of DDQ under wet conditions and the subsequent aqueous workup should be conducted in a fume hood. Bromine is a volatile, corrosive, and toxic liquid. Chemical-resistant gloves and safety goggles are essential for a safe handling. Toxic and corrosive hydrogen bromide vapors are released during bromination. A fume hood with proper ventilation is necessary to avoid a direct inhalation or skin contact. Sodium vigorously reacts with water. Exposure of sodium to moist air should be avoided. Sodium metal reacts violently with ethanol and releases flammable hydrogen gas. Therefore, the reaction should be conducted in a fume hood, especially on a large scale.

## Supplementary Material



## Data Availability

The data underlying this study are available in the published article and its Supporting Information.

## Notes

Experimental procedures, compound characterization data, cyclic voltammetry measurements, copies of NMR spectra (PDF)
