# Supplementary material for: Dimerization of Allylbenzenes into Cyclolignans by a Metathesis-Oxidation Sequence
Source: J Org Chem. 2026 Feb 26;91(10):3669–76. doi: 10.1021/acs.joc.5c02949 (PMC12993860; doi:10.1021/acs.joc.5c02949)

Supporting Information for

Dimerization of Allylbenzenes into Cyclolignans by a Metathesis-Oxidation Sequence

Kiryl Vasiutovich[a], Alexander A. Fadeev[a]*, Peter Čambal[b], and Eliška Matoušová[a]*

[a] Department of Organic Chemistry, Faculty of Science, Charles University, Hlavova 8, 128 00 Praha 2, Czech Republic

[b] Department of Analytical Chemistry, Faculty of Science, Charles University, Hlavova 8, 128 00 Praha 2, Czech Republic

* E-mail (AAF): aleksandr.fadeev@natur.cuni.cz

* E-mail (EM): eliska.matousova@natur.cuni.cz

1. General S2

2. Experimental procedures and compound characterisation data S3

2.1. Preparation of allylbenzenes S3
2.2. Preparation of internal alkenes by metathesis of allylbenzenes S8
2.3. Synthesis of lignans S12
2.3.1. One-pot synthesis of lignans from allylbenzenes S12
2.3.2. Synthesis of lignans from the cross-metathesis products S22
2.3.3. Gram-scale synthesis of oleralignan B S24
2.4. One-pot synthesis of benzyl styryl ketones S27
2.5. Assessment of the possible reaction intermediates S30
2.6. Functionalization of lignan **3a** S33

3. Cyclic voltammetry measurements S38

4. Copies of ^{1}H and ^{13}C NMR spectra S39

1. General

All chemicals and reagents were used as received from commercial suppliers without further purification. Acetone was dried by distillation from magnesium sulfate. Tetrahydrofuran and diethyl ether were dried by distillation from sodium/benzophenone. Anhydrous dichloromethane and methanol were purchased from Thermo Fisher Scientific. Solvents used for chromatographic separations were distilled prior to use. Analytical thin-layer chromatography (TLC) was performed on SiliCycle Silica gel 60-F254 coated aluminium plates. UV light (254 and 365 nm), phosphomolybdic acid, and basic potassium permanganate were used to visualize TLC plates. Column chromatography was performed using SiliaFlash P60 silica gel (40–60 µm, irregular) from SiliCycle. The NMR spectra were recorded on Bruker NEO 400 and Bruker AVANCE III HD 400 spectrometers (^{1}H at 400 MHz and ^{13}C at 101 MHz) as solutions in $CDCl_3$, CD_3OD, and DMSO-d_6. Chemical shifts are reported in parts per million and referenced to the residual solvent peaks: $CDCl_3$ (^{1}H, δ = 7.26; ^{13}C, δ = 77.16), CD_3OD (^{1}H, δ = 3.31; ^{13}C, δ = 49.00), and DMSO-d_6 (^{1}H, δ = 2.50; ^{13}C, δ = 39.52). Coupling constants J are given in Hz (Hertz), splitting patterns are abbreviated as s (singlet), br. s (broad singlet), d (doublet), t (triplet), q (quartet), m (multiplet). Structural assignments were made with the additional information from DEPT, APT, COSY, HSQC, and HMBC experiments. Infrared spectra were recorded in the range between 4000 and 400 cm^{-1} with Thermo Nicolet AVATAR 370 FT-IR spectrometer in KBr via ATR method and are reported in wavenumbers (cm^{-1}). High-resolution mass spectra were recorded on Agilent 6530 Accurate-Mass Q-TOF LC/MS, Bruker amaZon SL and Bruker Esquire 3000. Melting points were determined on a Büchi B-545 melting point apparatus and a Kofler KB T300 melting point apparatus. Cyclic voltammograms (CV) were recorded from the initial potential of 0 V in the direction of oxidation (scan rate 0.1 V/s) to the switching potential of +2.0 V using a Metrohm µAutolab type III potentiostat. Tetrabutylammonium tetrafluoroborate (0.01 M) in acetonitrile was used as the supporting electrolyte. The analyte concentration was 1×10^{-4} M. Commercially available boron doped diamond (BDD) electrode (d = 3 mm, BioLogic SAS, France) was used as the working electrode. The electrode was polished using Al_2O_3 suspension (Elektrochemické detektory, Turnov, Czechia, particle size 1.1 µm) on a polyurethane-fixated polishing pad (Elektrochemické detektory, Turnov, Czechia) prior each measurement. Pleskov Ag/Ag^+ reference electrode (Ag wire in 0.01 M $AgNO_3$ and 1 M $NaClO_4$ in acetonitrile) together with a Pt wire as the auxiliary electrode were employed to complete the three-electrode electrochemical cell setup. All electrochemical measurements were performed at room temperature (293 ± 1K). The recorded CVs were plotted according to the IUPAC convention.

2. Experimental procedures and compound characterisation data

2.1. Preparation of allylbenzenes

The preparation of the starting allylbenzenes is outlined in Scheme S1. Compounds **1a**, **1c**, **1g**, **1h**, **1p**, **1q** and **1r** were obtained from commercial sources. Compounds **1b**, **1d**, **1e**, **1f**, **1i**, **1k**, **1n**, **1o**, **1s**, and **1t** were synthesized according to the previously published procedures mentioned in Chapters 2.2. and 2.3.1. The synthesis of compounds **1j**, **1l**, **1m**, and **1u** is described below.

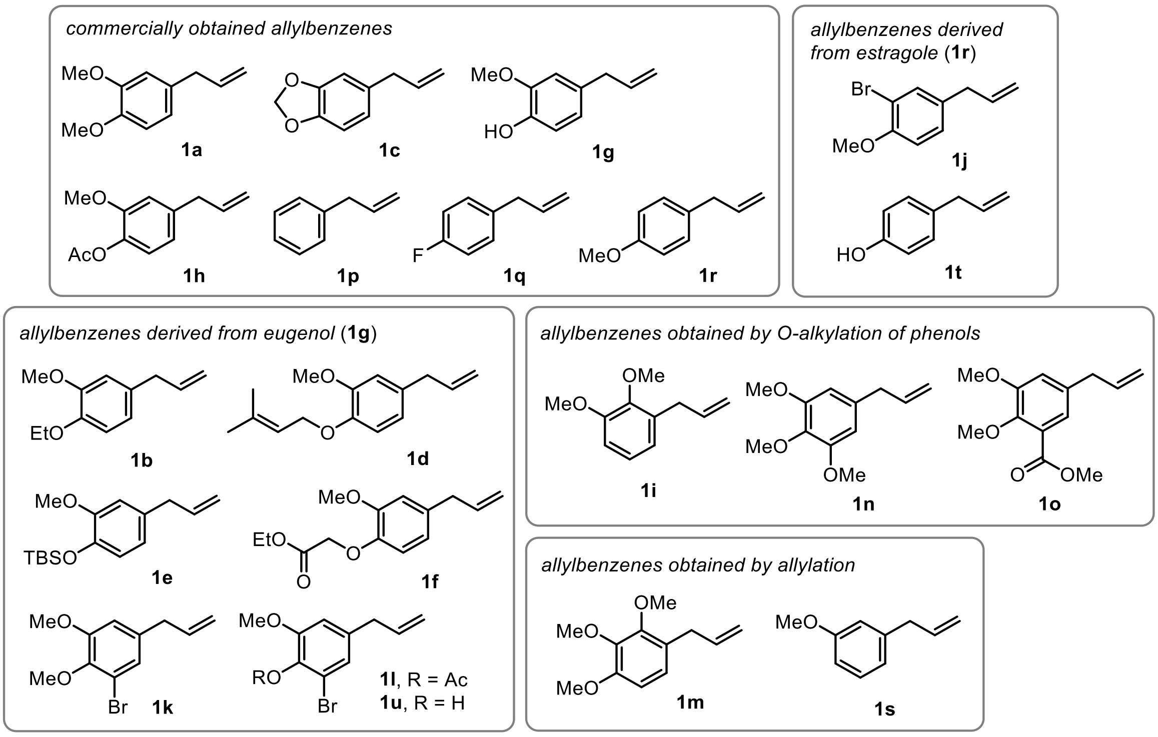

Scheme S1. Allylbenzenes used as the starting materials.

4-Allyl-2-bromo-1-methoxybenzene (1j) and **2-bromo-4-cyclopropyl-1-methoxybenzene (1j')**

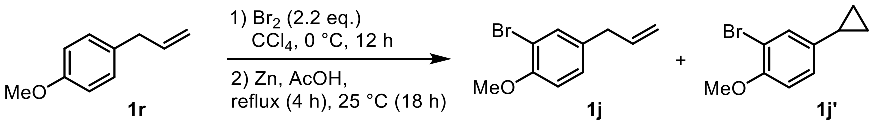

Caution! The addition of bromine causes a vigorous evolution of toxic and corrosive hydrogen bromide vapors!

Following the published procedure,[1] bromine (13.2 mmol, 0.68 mL, 2.11 g, 2.2 eq.) was slowly added to the solution of estragole (**1r**, 6 mmol, 890 mg) in CCl_4 (10 mL) dropwise at 0 °C. The mixture turned orange upon addition of the first equivalent of bromine and became colourless as it was consumed. Vigorous HBr release occurred during the addition of the second equivalent of Br_2. The solution was stirred for an additional 12 h, after which the volatiles were removed under reduced pressure. The residue was redissolved in 20/1 hexanes/EtOAc (50 mL), filtered through a pad of silica

[1] El-Feraly, F. S.; Cheatham, S. F.; Breedlove, R. L. *J. Nat. Prod.* **1983**, *46*, 493–498.

gel, and the latter was washed with 20/1 hexanes/EtOAc (100 mL). The solvent was removed under reduced pressure, and the residue was dissolved in Et_2O (10 mL). Acetic acid (0.2 mL) and zinc dust (15 mmol, 982 mg) were subsequently added to this solution. The mixture was refluxed for 4 h (oil bath) and then stirred at room temperature (25 °C) for 18 h. The inorganic precipitate was filtered, the filtrate was washed with Et_2O (20 mL), dried over anhydrous Na_2SO_4 and concentrated under reduced pressure. Column chromatography of the residue on silica gel using 98/2 hexanes/EtOAc as the eluent afforded compound **1j** as a colourless oil (689 mg, 51%) and by-product cyclopropane **1j'** as a colourless oil (138 mg, 10 %).

Allyl-2-bromo-1-methoxybenzene (1j)

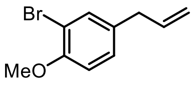

TLC R_f = 0.43 (20/1 hexanes/EtOAc);

^{1}H NMR (400 MHz, $CDCl_3$): δ = 7.37 (d, *J* = 2.2 Hz, 1H), 7.08 (dd, *J* = 8.4, 2.2 Hz, 1H), 6.83 (d, *J* = 8.4 Hz, 1H), 5.92 (ddt, *J* = 18.0, 9.3, 6.7 Hz, 1H), 5.11 – 5.07 (m, 1H), 5.07 – 5.04 (m, 1H), 3.87 (s, 3H), 3.31 (d, *J* = 6.7 Hz, 2H);

^{13}C{^{1}H} NMR (101 MHz, $CDCl_3$): δ = 154.4, 137.2, 133.8, 133.5, 128.6, 116.2, 112.1, 111.6, 56.4, 39.0.

The recorded spectroscopic values agree with the previously reported data.[1]

2-Bromo-4-cyclopropyl-1-methoxybenzene (1j')

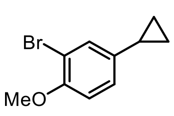

TLC R_f = 0.41 (20/1 hexanes/EtOAc);

^{1}H NMR (400 MHz, $CDCl_3$): δ = 7.26 (d, *J* = 2.3 Hz, 1H), 7.00 (dd, *J* = 8.4, 2.3 Hz, 1H), 6.79 (d, *J* = 8.4 Hz, 1H), 3.86 (s, 3H), 1.83 (tt, *J* = 8.4, 5.1 Hz, 1H), 0.94 – 0.89 (m, 2H), 0.64 – 0.58 (m, 2H);

^{13}C{^{1}H} NMR (101 MHz, $CDCl_3$): δ = 153.9, 137.8, 130.9, 126.1, 112.0, 111.6, 56.5, 14.5, 8.8 (2C).

The recorded spectroscopic values agree with the previously reported data.[2]

4-Allyl-2-bromo-6-methoxyphenol (1u)

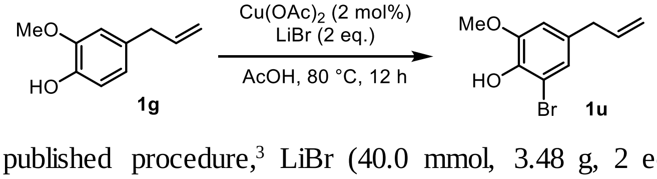

Following the published procedure,[3] LiBr (40.0 mmol, 3.48 g, 2 eq.) and $Cu(OAc)_2{\cdot}H_2O$ (0.4 mmol, 80 mg, 0.02 eq.) were added to a solution of eugenol (**1g**, 20.0 mmol, 3.29 g) in glacial acetic acid (50 mL). The reaction flask was connected to a thick-wall balloon filled with oxygen, and reaction mixture was stirred at 80 °C for 12 h (oil bath). After that, the resulting suspension was diluted with water (100 mL) carefully neutralized with saturated aqueous $NaHCO_3$, and the aqueous layer was

[2] Karpova, V. V.; Mochalov, S. S.; Shabarov, Yu. S. *J. Org. Chem. USSR (Eng. Transl.)* **1982**, *18*, 268–277.
[3] Menini, L.; Parreira, L. A.; Gusevskaya, E. V. *Tetrahedron Lett.* **2007**, *48*, 6401–6404.

extracted with EtOAc (3×200 mL). The combined organic layers were subsequently washed with water (100 mL), brine (100 mL), dried over anhydrous Na_2SO_4, and concentrated under reduced pressure. Column chromatography of the residue on silica gel using 9/1 hexanes/EtOAc as the eluent afforded 4-allyl-2-bromo-6-methoxyphenol as a yellow oil (1.485 g, 31 %). This compound was obtained as a technical grade product (^{1}H NMR showed 90% purity) and used directly in the synthesis.

TLC R_f = 0.58 (7/3 hexanes/EtOAc);

^{1}H NMR (400 MHz, $CDCl_3$): δ = 6.93 (d, *J* = 1.8 Hz, 1H), 6.63 (d, *J* = 1.8 Hz, 1H), 5.91 (ddt, *J* = 16.5, 9.5, 6.8 Hz, 1H), 5.78 (br. s, 1H), 5.12 – 5.08 (m, 1H), 5.08 – 5.05 (m, 1H), 3.88 (s, 3H), 3.29 (d, *J* = 6.8 Hz, 2H);

^{13}C{^{1}H} NMR (101 MHz, $CDCl_3$): δ = 147.2, 141.5, 137.1, 132.9, 124.5, 116.4, 110.6, 108.2, 56.4, 39.7.

The recorded spectroscopic values agree with the previously reported data.[4]

5-Allyl-1-bromo-2,3-dimethoxybenzene (1k)

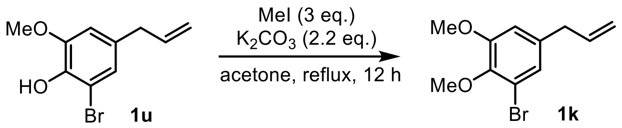

To a solution of 4-allyl-2-bromo-6-methoxyphenol (**1u**, 2.5 mmol, 608 mg) in dry acetone (10 mL) were added K_2CO_3 (5.5 mmol, 760 mg, 2.2 eq.) and methyl iodide (7.5 mmol, 0.47 mL, 3.0 eq.). The resulting suspension was heated under reflux with stirring for 12 hours (oil bath). After that, the solvent was removed under reduced pressure. Water (20 mL) was added and the product was extracted with ethyl acetate (3×20 mL). The combined organic extracts were washed with water (10 mL) and brine (10 mL), dried over anhydrous Na_2SO_4, and concentrated under reduced pressure. Column chromatography of the residue on silica gel using 20/1 hexanes/EtOAc as the eluent afforded 562 mg of the title compound as a yellowish oil (87 % yield).

TLC R_f = 0.59 (9/1 hexanes/EtOAc);

^{1}H NMR (400 MHz, $CDCl_3$): δ = 6.96 (d, *J* = 1.9 Hz, 1H), 6.67 (d, *J* = 1.9 Hz, 1H), 5.92 (ddt, *J* = 17.5, 9.5, 6.7 Hz, 1H), 5.13 – 5.10 (m, 1H), 5.09 – 5.07 (m, 1H), 3.85 (s, 3H), 3.83 (s, 3H), 3.31 (dd, *J* = 6.7, 1.5 Hz, 2H);

^{13}C{^{1}H} NMR (101 MHz, $CDCl_3$): δ = 153.7, 144.9, 137.4, 136.7, 124.6, 117.6, 116.6, 112.2, 60.7, 56.2, 39.8.

The recorded spectroscopic values agree with the previously reported data.[5]

[4] Pilkington, L.; Barker, D. *Synlett* **2015**, *26*, 2425–2428.

[5] Sciacca, C.; Cardullo, N.; Pulvirenti, L.; Travagliante, G.; D'Urso, A.; D'Agata, R.; Peri, E.; Cancemi, P.; Cornu, A.; Deffieux, D.; Pouységu, L.; Quideau, S.; Muccilli, V. *Bioorg. Chem.* **2024**, *147*, 107392.

4-Allyl-2-bromo-6-methoxyphenyl acetate (1l)

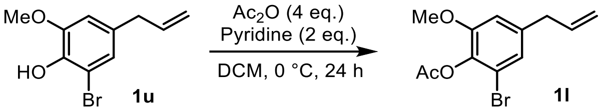

A solution of 4-allyl-2-bromo-6-methoxyphenol (**1u**, 2.5 mmol, 607 mg) in DCM (5 mL) held at 0 °C was treated with pyridine (5.0 mmol, 0.40 mL, 2.0 eq.) and acetic anhydride (10.0 mmol, 0.94 mL, 4.0 eq.). The cooling bath was removed and the mixture was stirred until the starting material was consumed. After completion monitored by TLC (24 h), the reaction mixture was poured into water and extracted with DCM (3×20 mL). The organic extract was subsequently washed with 1M aq. HCl (10 mL), sat. $NaHCO_3$ (10 mL), water (10 mL), dried over anhydrous Na_2SO_4, and concentrated under reduced pressure. Column chromatography of the residue on silica gel using 9/1 hexanes/EtOAc as the eluent afforded compound **1l** (579 mg, 81 %) as a colourless oil.

TLC R_f = 0.38 (9/1 hexanes/EtOAc);

^{1}H NMR (400 MHz, $CDCl_3$): δ = 7.01 (d, *J* = 1.8 Hz, 1H), 6.72 (d, *J* = 1.8 Hz, 1H), 5.92 (ddt, *J* = 17.5, 9.6, 6.8 Hz, 1H), 5.16 – 5.12 (m, 1H), 5.12 – 5.08 (m, 1H), 3.81 (s, 3H), 3.34 (d, *J* = 6.8 Hz, 2H), 2.35 (s, 3H);

^{13}C{^{1}H} NMR (101 MHz, $CDCl_3$): δ = 168.2, 152.3, 139.9, 136.3, 136.2, 124.4, 117.04, 116.96, 111.9, 56.3, 39.9, 20.6;

IR (KBr): ṽ = 3078, 3007, 2976, 2941, 2914, 2841, 1767, 1597, 1578, 1485, 1464, 1416, 1367, 1317, 1277, 1182, 1140, 1045, 1009, 899, 845, 829, 804, 696 cm^{-1};

HRMS (ESI) m/z: $[M+Na]^+$ Calcd for $C_{12}H_{13}BrO_3Na^+$: 306.9940; Found: 306.9942.

1-Allyl-2,3,4-trimethoxybenzene (1m)

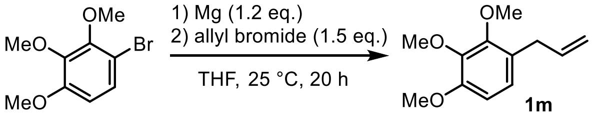

The solution of 1-bromo-2,3,4-trimethoxybenzene[6] (4.5 mmol, 1.114 g) in THF (20 mL) was added to dry magnesium turnings (5.4 mmol, 132 mg, 1.2 eq.) under argon atmosphere. One small crystal of iodine was added, and the reaction mixture was stirred for a further 1 h. Allyl bromide (6.8 mmol, 584 µL, 1.5 eq.) was slowly added, and stirring was continued at 25 °C for 20 h. Upon completion (TLC monitoring), the reaction mixture was partitioned between saturated NH_4Cl and Et_2O (50 mL each), the organic layer was separated, and the aqueous layer was extracted with Et_2O (3×25 mL). The combined organic extracts were dried over anhydrous Na_2SO_4, and concentrated under reduced pressure. Column chromatography of the residue on silica gel using 97/3 hexanes/EtOAc as the eluent afforded the title compound as a colourless oil (659 mg, 70 %).

TLC R_f = 0.46 (9/1 hexanes/EtOAc);

[6] Yadav, J. S.; Reddy, B. V. S.; Reddy, P. S. R.; Basak, A. K.; Narsaiah, A. V. *Adv. Synth. Catal.* **2004**, *346*, 77–82.

^{1}H NMR (400 MHz, $CDCl_3$): δ = 6.83 (d, *J* = 8.5 Hz, 1H), 6.62 (d, *J* = 8.5 Hz, 1H), 5.96 (ddt, *J* = 17.7, 9.5, 6.5 Hz, 1H), 5.08 – 5.03 (m, 1H), 5.04 – 4.98 (m, 1H), 3.87 (s, 3H), 3.86 (s, 3H), 3.84 (s, 3H), 3.34 (d, *J* = 6.5 Hz, 2H);

^{13}C{^{1}H} NMR (101 MHz, $CDCl_3$): δ = 152.4, 151.9, 142.5, 137.8, 126.3, 124.1, 115.4, 107.4, 61.1, 60.9, 56.1, 33.9.

The recorded spectroscopic values agree with the previously reported data.[7]

[7] Bochicchio, A.; Cefola, R.; Choppin, S.; Colobert, F.; Di Noia, M. A.; Funicello, M.; Hanquet, G.; Pisano, I.; Todisco, S.; Chiummiento, L. *Tetrahedron Lett.* **2016**, *57*, 4053–4055.

2.2. Preparation of internal alkenes by metathesis of allylbenzenes

(*E*)-1,4-Bis(3,4-dimethoxyphenyl)but-2-ene (2a)

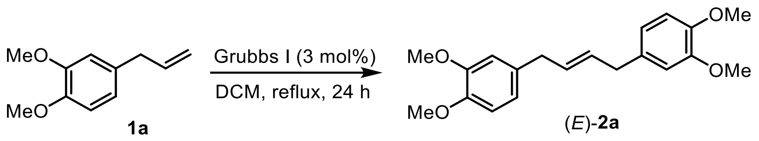

To a solution of 1st generation Grubbs catalyst (75 µmol, 62 mg) in degassed DCM (5 mL) under argon atmosphere was added methyl eugenol (2.5 mmol, 445 mg, 450 µL) and the reaction mixture was refluxed for 24 h (oil bath). After that, the volatiles were removed under reduced pressure. Column chromatography of the residue on silica gel using 5/1 hexanes/EtOAc as the eluent gave 408 mg of the title compound as a brown solid (99% yield, 5/1 *E*/*Z* ratio according to ^{1}H NMR). Recrystallization of this material from hexanes/EtOAc gave 307 mg (75%) of the major *E*-isomer as greyish crystals (leaflets).

TLC R_f = 0.47 (1/1 hexanes/EtOAc);

M.p. 88 °C (from hexanes/EtOAc);

^{1}H NMR (400 MHz, $CDCl_3$): δ = 6.81 – 6.79 (m, 2H), 6.75 – 6.71 (m, 4H), 5.66 (tt, *J* = 3.7, 1.6 Hz, 2H), 3.86 – 3.85 (m, 12H), 3.33 – 3.30 (m, 4H);

^{13}C{^{1}H} NMR (101 MHz, $CDCl_3$): δ = 149.0 (2C), 147.4 (2C), 133.5 (2C), 130.7 (2C), 120.4 (2C), 112.0 (2C), 111.4 (2C), 56.1 (2C), 55.9 (2C), 38.6 (2C).

The recorded spectroscopic values agree with the previously reported data.[8]

1,4-Bis(3-methoxyphenyl)but-2-ene (2s) and 4-(4-(3-Methoxyphenyl)but-2-en-1-yl)phenol (2st)

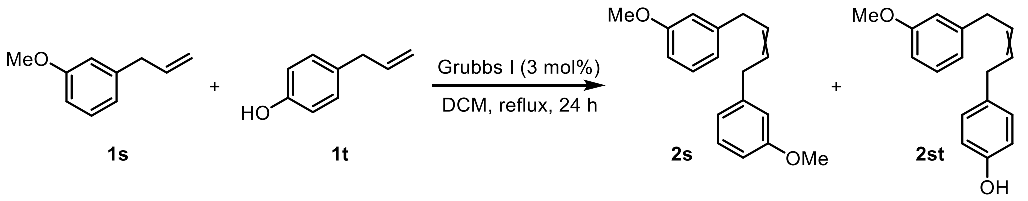

To a solution of 1st generation Grubbs catalyst (39 µmol, 32 mg) in degassed DCM (15 mL) under argon atmosphere were added 4-allylphenol[9] (**1t**, 1.3 mmol, 174 mg, 1 eq.) and 1-allyl-3-methoxybenzene[10,11,12] (**1s**, 7.8 mmol, 1.152 g, 6.0 eq.), and the reaction mixture was refluxed for 24 h (oil bath). After that, the volatiles were removed under reduced pressure. Column chromatography of the residue on silica gel using 20/1 hexanes/EtOAc as the eluent afforded alkene **2s** as a colourless oil (813 mg, 78% yield based on **1s**, 5/1 *E*/*Z* ratio according to ^{1}H NMR). Further elution with 5/1

---

[8] Kundu, K.; Nayak, S. K. *J. Nat. Prod.* **2017**, *80*, 1776–1782.

[9] Jo, H.; Choi, M.; Viji, M.; Lee, Y. H.; Kwak, Y.-S.; Lee, K.; Choi, N. S.; Lee, Y.-J.; Lee, H.; Hong, J. T.; Lee, M. K.; Jung, J.-K. *Molecules* **2015**, *20*, 15966–15975.

[10] Lusch, L. J.; Woller, K. R.; Keller, A. M.; Turk, M. C. *Synthesis* **2005**, 551–554.

[11] Tyman, J. H. P.; Payne, P. B. *J. Chem. Res.* **2006**, 691–695.

[12] Kawamura, K. E.; Chang, A. S.; Martin, D. J.; Smith, H. M.; Morris, P. T.; Cook, A. K. *Organometallics* **2022**, *41*, 486–496.

hexanes/EtOAc gave 204 mg of compound **2st** as a yellowish oil (62% yield based on **1t**, 4/1 *E*/*Z* ratio according to ^{1}H NMR).

1,4-Bis(3-methoxyphenyl)but-2-ene (2s)

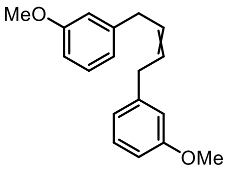

TLC R_f = 0.51 (9/1 hexanes/EtOAc);

^{1}H NMR (400 MHz, $CDCl_3$), major *E*-isomer: δ = 7.25 – 7.13 (m, 2H), 6.86 – 6.71 (m, 6H), 5.70 – 5.67 (m, 2H), 3.80 (s, 6H), 3.36 (d, *J* = 3.1 Hz, 4H);

^{13}C{^{1}H} NMR (101 MHz, $CDCl_3$), major *E*-isomer: δ = 159.8 (2C), 142.5 (2C), 130.5 (2C), 129.5 (2C), 121.0 (2C), 114.3 (2C), 111.5 (2C), 55.3 (2C), 39.1 (2C);

^{1}H NMR (400 MHz, $CDCl_3$), minor *Z*-isomer: δ = 7.25 – 7.13 (m, 2H), 6.86 – 6.71 (m, 6H), 5.75 – 5.71 (m, 2H), 3.80 (s, 6H), 3.51 (d, *J* = 4.9 Hz, 4H).

The recorded spectroscopic values agree with the previously reported data.[13]

4-(4-(3-Methoxyphenyl)but-2-en-1-yl)phenol (2st)

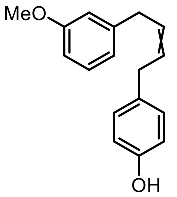

TLC R_f = 0.43 (7/3 hexanes/EtOAc);

^{1}H NMR (400 MHz, $CDCl_3$), major *E*-isomer: δ = 7.26 – 7.18 (m, 1H), 7.12 – 7.02 (m, 2H), 6.86 – 6.70 (m, 5H), 5.74 – 5.58 (m, 2H), 4.86 (br. s, 1H), 3.80 (s, 3H), 3.39 – 3.25 (m, 4H);

^{13}C{^{1}H} NMR (101 MHz, $CDCl_3$), major *E*-isomer: δ = 159.8, 153.9, 142.6, 133.0, 131.1, 130.0, 129.8 (2C), 129.5, 121.1, 115.3 (2C), 114.3, 111.5, 55.3, 39.1, 38.2;

^{1}H NMR (400 MHz, $CDCl_3$), minor *Z*-isomer: δ = 7.26 – 7.18 (m, 1H), 7.12 – 7.02 (m, 2H), 6.86 – 6.70 (m, 5H), 5.74 – 5.58 (m, 2H), 4.86 (br. s, 1H), 3.80 (s, 3H), 3.55 – 3.41 (m, 4H);

IR (KBr): ṽ = 3379, 3022, 2904, 2835, 1600, 1584, 1511, 1489, 1452, 1435, 1254, 1225, 1147, 1041, 970, 822, 777, 694 cm^{-1};

HRMS (ESI) m/z: $[M-H]^-$ Calcd for $C_{17}H_{17}O_2^-$: 253.1234; Found: 253.1237.

1-Methoxy-3-(4-(4-methoxyphenyl)but-2-en-1-yl)benzene (2rs)

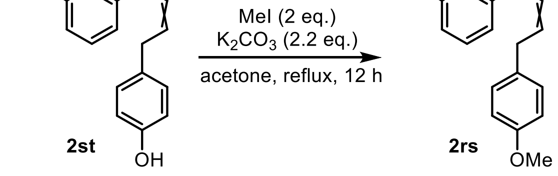

To the solution of 4-(4-(3-methoxyphenyl)but-2-en-1-yl)phenol (**2st**, 0.7 mmol, 178 mg) in acetone (5 mL) was added K_2CO_3 (1.5 mmol, 213 mg, 2.2 eq.) and methyl iodide (1.4 mmol, 87 µL, 2.0 eq.) under argon atmosphere. The resulting suspension was heated under reflux with stirring for 12 h (oil bath). After that, acetone was evaporated under reduced pressure. Water (10 mL) was added, and

[13] Gresser, M. J.; Wales, S. M.; Keller, P. A. *Tetrahedron* **2010**, *66*, 6965–6976.

the product was extracted with EtOAc (3×15 mL). The combined organic extracts were washed with water (10 mL), brine (10 mL), dried over anhydrous Na_2SO_4, and concentrated under reduced pressure. Column chromatography of the residue on silica gel using 20/1 hexanes/EtOAc as the eluent afforded alkene **2rs** as a colourless oil (132 mg, 70 %, 5/1 *E*/*Z* ratio according to ^{1}H NMR).

TLC R_f = 0.50 (9/1 hexanes/EtOAc);

^{1}H NMR (400 MHz, $CDCl_3$), major *E*-isomer: δ = 7.25 – 7.18 (m, 1H), 7.17 – 7.09 (m, 2H), 6.89 – 6.72 (m, 5H), 5.69 – 5.63 (m, 2H), 3.80 (s, 6H), 3.40 – 3.28 (m, 4H);

^{13}C{^{1}H} NMR (101 MHz, $CDCl_3$), major *E*-isomer: δ = 159.8, 158.0, 142.6, 132.9, 131.2, 130.0, 129.6 (2C), 129.5, 121.0, 114.3, 114.0 (2C), 111.5, 55.4, 55.3, 39.1, 38.2;

^{1}H NMR (400 MHz, $CDCl_3$), minor *Z*-isomer: δ = 7.25 – 7.18 (m, 1H), 7.17 – 7.09 (m, 2H), 6.89 – 6.72 (m, 5H), 5.74 – 5.68 (m, 2H), 3.80 (s, 6H), 3.56 – 3.42 (m, 4H);

IR (KBr): ṽ = 2954, 2920, 2835, 1601, 1583, 1510, 1489, 1464, 1454, 1437, 1246, 1174, 1151, 1036, 970, 818, 779, 696 cm^{-1};

HRMS (ESI) m/z: $[M+Na]^+$ Calcd for $C_{18}H_{20}O_2Na^+$: 291.1356; Found: 291.1354.

1,4-Bis(4-methoxyphenyl)but-2-ene (2r) and **1,2-dimethoxy-4-(4-(4-methoxyphenyl)but-2-en-1-yl)benzene (2ar)**

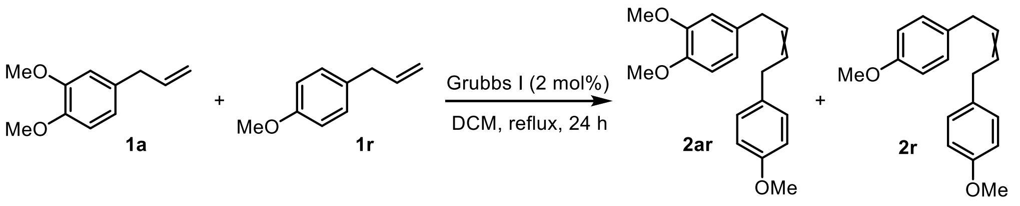

To a solution of 1st generation Grubbs catalyst (41 µmol, 34 mg) in degassed DCM (20 mL) under argon atmosphere were added methyl eugenol **1a** (2.1 mmol, 367 mg, 1 eq.) and estragole **1r** (12.4 mmol, 1.861 g, 6.1 eq.), and the reaction mixture was refluxed for 24 h (oil bath). After that, the volatiles were removed under reduced pressure. Column chromatography of the residue on silica gel using 20/1 hexanes/EtOAc as the eluent gave **2r** as a white solid (1.194 g, 71% yield based on **1r**, 4/1 *E*/*Z* ratio according to ^{1}H NMR). Further elution with 4/1 hexanes/EtOAc gave 419 mg of compound **2ar** as a colourless oil (68% yield based on **1a**, 3/1 *E*/*Z* ratio according to ^{1}H NMR).

1,4-Bis(4-methoxyphenyl)but-2-ene (2r)

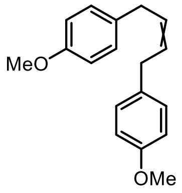

TLC R_f = 0.75 (4/1 hexanes/EtOAc);

^{1}H NMR (400 MHz, $CDCl_3$), major *E*-isomer: δ = 7.13 (d, *J* = 8.5 Hz, 4H), 6.86 (d, *J* = 8.5 Hz, 4H), 5.67 – 5.61 (m, 2H), 3.81 (s, 6H), 3.33 (d, *J* = 3.4 Hz, 4H);

^{13}C{^{1}H} NMR (101 MHz, $CDCl_3$), major *E*-isomer: δ = 158.0 (2C), 133.0 (2C), 130.6 (2C), 129.5 (4C), 113.9 (4C), 55.4 (2C), 38.2 (2C);

^{1}H NMR (400 MHz, $CDCl_3$), minor *Z*-isomer: δ = 7.21 – 7.04 (m, 4H), 6.90 – 6.79 (m, 4H), 5.73 – 5.67 (m, 2H), 3.87 – 3.74 (m, 6H), 3.47 (d, *J* = 4.3 Hz, 4H).

The recorded spectroscopic values agree with the previously reported data.[14]

1,2-Dimethoxy-4-(4-(4-methoxyphenyl)but-2-en-1-yl)benzene (2ar)

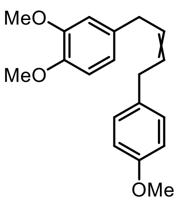

TLC R_f = 0.55 (7/3 hexanes/EtOAc);

^{1}H NMR (400 MHz, $CDCl_3$), major *E*-isomer: δ = 7.12 (d, *J* = 8.6 Hz, 2H), 6.91 – 6.67 (m, 5H), 5.68 – 5.62 (m, 2H), 3.87 (s, 3H), 3.86 (s, 3H), 3.79 (s, 3H), 3.38 – 3.24 (m, 4H);

^{13}C{^{1}H} NMR (101 MHz, $CDCl_3$), major *E*-isomer: δ = 158.0, 149.0, 147.4, 133.5, 132.9, 130.9, 130.4, 129.5 (2C), 120.4, 113.9 (2C), 111.9, 111.3, 56.0, 55.9, 55.4, 38.6, 38.2;

^{1}H NMR (400 MHz, $CDCl_3$), minor *Z*-isomer: δ = 7.19 – 7.08 (m, 2H), 6.91 – 6.68 (m, 5H), 5.77 – 5.67 (m, 2H), 3.92 – 3.75 (m, 9H), 3.52 – 3.42 (m, 4H);

IR (KBr): ṽ = 3492, 3049, 3028, 2999, 2952, 2933, 2906, 2835, 1670, 1601, 1512, 1464, 1300, 1246, 1176, 1155, 1140, 812, 764 cm^{-1};

HRMS (ESI) m/z: $[M+Na]^+$ Calcd for $C_{19}H_{22}O_3Na^+$: 321.1461; Found: 321.1459.

[14] McLane, R. D.; Le Cozannet-Laidin, L.; Boyle, M. S.; Lanzillotta, L.; Taylor, Z. L.; Anthony, S. R.; Tranter, M.; Onorato, A. J. *Bioorg. Med. Chem. Lett.* **2018**, *28*, 334–338.

2.3. Synthesis of lignans

Procedure for the screening of the annulation reaction conditions

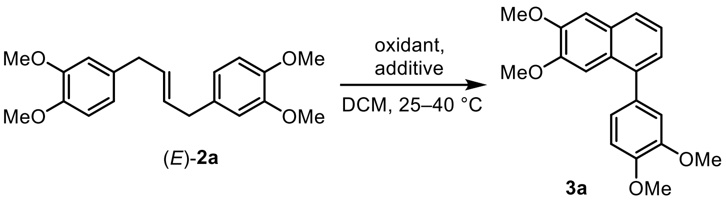

In a 3 mL screw cap vial containing a solution of (*E*)-**2a** (0.05 mmol, 16.4 mg) in DCM (0.5 mL) was added the additive as a 0.1 M solution in DCM (2.5–7.5 μmol, 25–75 μL; also applies for $FeCl_3$ in a catalytic amount), followed by the oxidant(s) (0.1–0.3 mmol, DDQ was always added last), and the reaction mixture was stirred at 25 or 40 °C in a metal block under air atmosphere until full conversion of **2a** into **3a** was observed (24 h). Next, the reaction mixture was diluted with hexanes (0.5 mL) and the resulting suspension was filtered through a pad of silica gel (d = 5 mm, h = 50 mm, not pre-wetted), and the pad was washed with 1:1 hexanes/EtOAc mixture (10 mL). The solution was concentrated under reduced pressure, and the residue was dissolved in $CDCl_3$ (0.6 mL) and analyzed by ^{1}H NMR with dibromomethane (0.1 mmol, 7 μL) as the internal standard. The exact amounts of reagents and the obtained results are given in Table 1 of the main text.

2.3.1. One-pot synthesis of lignans from allylbenzenes

General Procedure A (MsOH as the additive)

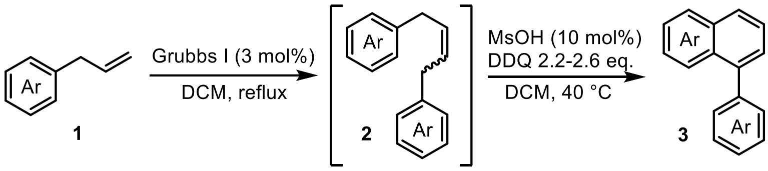

To a solution of 1st generation Grubbs catalyst (0.06 mmol, 49 mg) in dry degassed DCM (4 mL) under argon atmosphere was added allylbenzene **1** (2 mmol) and the reaction mixture was refluxed for 24 h (oil bath). After that, the mixture was cooled to room temperature (25 °C) and diluted with DCM to 10 mL. Next, a solution of methanesulfonic acid in DCM (0.1 mmol, 0.5 M, 0.2 mL) and DDQ (2.2–2.6 mmol) were added and the resulting mixture was stirred at 40 °C for 24 h under air atmosphere (oil bath). After that, the volatiles were removed under reduced pressure and the residue was subjected to column chromatography to afford the desired lignan **3**.

1-(3,4-Dimethoxyphenyl)-6,7-dimethoxynaphthalene (3a)

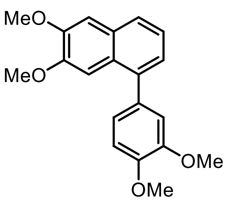

The title compound was prepared from methyl eugenol **1a** (2 mmol, 356 mg) and DDQ (2.5 mmol, 568 mg) according to the General Procedure A. Column chromatography on silica gel using 7/1 hexanes/EtOAc as the eluent gave 217 mg (67%) of the title compound as a colourless solid.

TLC R_f = 0.46 (1/1 hexanes/EtOAc);

M.p. 156 °C (from hexanes/EtOAc);

^{1}H NMR (400 MHz, $CDCl_3$): δ = 7.69 (d, *J* = 8.1 Hz, 1H), 7.38 (dd, *J* = 8.1, 7.1 Hz, 1H), 7.29 (dd, *J* = 7.1, 1.3 Hz, 1H), 7.27 (s, 1H), 7.19 (s, 1H), 7.07 (dd, *J* = 8.1, 1.9 Hz, 1H), 7.05 (d, *J* = 1.9 Hz, 1H), 7.01 (d, *J* = 8.1 Hz, 1H), 4.02 (s, 3H), 3.98 (s, 3H), 3.90 (s, 3H), 3.83 (s, 3H);

^{13}C{^{1}H} NMR (101 MHz, $CDCl_3$): δ = 149.6, 149.4, 148.8, 148.3, 138.7, 134.0, 129.9, 127.5, 125.9, 125.5, 124.0, 122.1, 113.2, 111.3, 106.8, 104.9, 56.1 (2C), 56.0, 55.8;

IR (KBr): ṽ = 3001, 2983, 2966, 2935, 2835, 1622, 1597, 1576, 1506, 1483, 1456, 1431, 1360, 1248, 1228, 1155, 1142, 1101, 1024, 1007, 876, 849, 814, 795, 756 cm^{-1};

HRMS (ESI) m/z: $[M+H]^+$ Calcd for $C_{20}H_{21}O_4^+$: 325.1434; Found: 325.1435.

The recorded spectroscopic values agree with the previously reported data.[15]

7-Ethoxy-1-(4-ethoxy-3-methoxyphenyl)-6-methoxynaphthalene (3b)

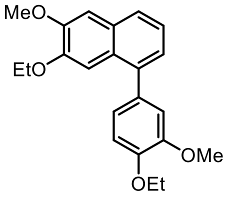

The title compound was prepared from ethyl eugenol[16] **1b** (2 mmol, 385 mg) and DDQ (2.5 mmol, 568 mg) according to the General Procedure A. Column chromatography on silica gel using 85/15 hexanes/EtOAc as the eluent gave 234 mg (66%) of the title compound as a colourless solid.

TLC R_f = 0.59 (7/3 hexanes/EtOAc);

M.p. 111 °C (from MeOH);

^{1}H NMR (400 MHz, $CDCl_3$): δ = 7.68 (d, *J* = 8.1 Hz, 1H), 7.37 (dd, *J* = 8.1, 7.0 Hz, 1H), 7.28 (dd, *J* = 7.0, 1.3 Hz, 1H), 7.27 (s, 1H), 7.19 (s, 1H), 7.05 – 6.98 (m, 3H), 4.20 (q, *J* = 7.0 Hz, 2H), 4.03 (q, *J* = 7.0 Hz, 2H), 4.01 (s, 3H), 3.89 (s, 3H), 1.54 (t, *J* = 7.0 Hz, 3H), 1.46 (t, *J* = 7.0 Hz, 3H);

^{13}C{^{1}H} NMR (101 MHz, $CDCl_3$): δ =149.6, 148.9, 148.8, 147.6, 138.7, 134.0, 129.8, 127.5, 125.9, 125.3, 123.9, 122.1, 113.5, 112.6, 106.8, 106.0, 64.4, 64.1, 56.1, 56.0, 15.0, 14.6;

IR (KBr): ṽ = 3049, 2972, 2949, 2904, 2864, 2831, 1624, 1608, 1581, 1506, 1468, 1452, 1390, 1246, 1207, 1157, 1134, 1038, 1018, 922, 835, 781, 768, 741 cm^{-1};

HRMS (ESI) m/z: $[M+H]^+$ Calcd for $C_{22}H_{25}O_4^+$: 353.1747; Found: 353.1742.

---

[15] Nair, V.; Sheeba, V.; Panicker, S. B.; George, T. G.; Rajan, R.; Balagopal, L.; Vairamani, M.; Prabhakar, S. *Tetrahedron* **2000**, *56*, 2461–2467.

[16] Maurya, R.; Ahmad, A.; Gupta, P.; Chand, K.; Kumar, M.; Jayendra; Rawat, P.; Rasheed, N.; Palit, G. *Med. Chem. Res.*, **2011**, *20*, 139–145.

5-(Benzo[*d*][1,3]dioxol-5-yl)naphtho[2,3-*d*][1,3]dioxole (3c)

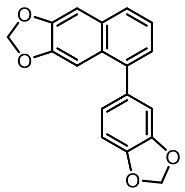

The title compound was prepared from safrole **1c** (2 mmol, 324 mg) and DDQ (2.5 mmol, 568 mg) according to the General Procedure A. Column chromatography on silica gel using 30/1 hexanes/EtOAc as the eluent gave 119 mg (41%) of the title compound as a colourless solid.

TLC R_f = 0.50 (3/1 hexanes/EtOAc);

M.p. 141 °C (from hexanes/EtOAc);

^{1}H NMR (400 MHz, $CDCl_3$): δ = 7.66 (d, *J* = 8.1 Hz, 1H), 7.35 (dd, *J* = 8.1, 7.1 Hz, 1H), 7.27 – 7.21 (m, 2H), 7.17 (s, 1H), 6.97 – 6.89 (m, 3H), 6.04 (s, 2H), 6.01 (s, 2H);

^{13}C{^{1}H} NMR (101 MHz, $CDCl_3$): δ = 148.0, 147.6, 147.4, 146.9, 139.2, 135.1, 131.1, 129.0, 126.7, 125.7, 124.1, 123.3, 110.6, 108.4, 104.2, 102.5, 101.23, 101.17;

IR (KBr): ṽ = 3037, 2912, 2783, 1620, 1485, 1458, 1245, 1223, 1169, 1103, 1036, 935, 856, 795, 752 cm^{-1};

HRMS (APPI) m/z: $[M]^{\bullet+}$ Calcd for $C_{18}H_{12}O_4^{\bullet+}$: 292.0730; Found: 292.0731.

6-Methoxy-1-(3-methoxy-4-((3-methylbut-2-en-1-yl)oxy)phenyl)-7-((3-methylbut-2-en-1-yl)oxy)naphthalene (3d)

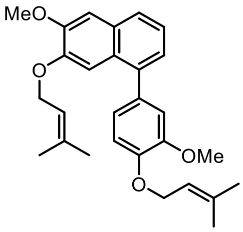

The title compound was prepared from prenyleugenol[17] (**1d**, 2 mmol, 465 mg) and DDQ (2.6 mmol, 590 mg) according to the General Procedure A. Column chromatography on silica gel using 9/1 hexanes/EtOAc as the eluent gave 182 mg (42%) of the title compound as a white solid.

TLC R_f = 0.49 (4/1 hexanes/EtOAc);

M.p. 120 °C (from MeOH);

^{1}H NMR (400 MHz, $CDCl_3$): δ = 7.68 (d, *J* = 8.0 Hz, 1H), 7.36 (dd, *J* = 8.0, 7.1 Hz, 1H), 7.27 (d, *J* = 7.1 Hz, 1H), 7.26 (s, 1H), 7.18 (s, 1H), 7.04 – 7.00 (m, 3H), 5.65 – 5.56 (m, 1H), 5.48 – 5.42 (m, 1H), 4.68 (d, *J* = 6.7 Hz, 2H), 4.55 (d, *J* = 6.6 Hz, 2H), 4.01 (s, 3H), 3.88 (s, 3H), 1.81 (s, 3H), 1.78 (s, 3H), 1.72 (s, 3H), 1.57 (s, 3H);

^{13}C{^{1}H} NMR (101 MHz, $CDCl_3$): δ = 149.8, 149.2, 148.7, 147.6, 138.8, 137.8, 137.7, 134.2, 129.8, 127.5, 125.9, 125.3, 123.8, 122.0, 120.2, 119.8, 113.4, 113.0, 106.7, 106.4, 66.0, 65.6, 56.03, 55.97, 26.0, 25.9, 18.4, 18.2;

IR (KBr): ṽ = 2985, 2962, 2931, 2912, 2873, 2835, 1682, 1622, 1593, 1574, 1510, 1479, 1452, 1388, 1327, 1306, 1252, 1238, 1225, 1136, 1028, 982, 854, 789, 768 cm^{-1};

HRMS (ESI) m/z: $[M+Na]^+$ Calcd for $C_{28}H_{32}O_4Na^+$: 455.2193; Found: 455.2196.

[17] Jiang, T.-S.; Cai, Y.; Zhang, Q.; Li, G.; Cheng, X. *Synthesis* **2018**, *50*, 4611–4616.

Tert-butyl((8-(4-((_tert_-butyldimethylsilyl)oxy)-3-methoxyphenyl)-3-methoxynaphthalen-2-yl)oxy)dimethylsilane (3e)

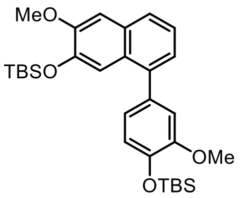

The title compound was prepared from *O*-(*tert*-butyl)dimethylsilyleugenol[18] (**1e**, 2 mmol, 557 mg) and DDQ (2.6 mmol, 590 mg) according to the General Procedure A. Column chromatography on silica gel using 99/1 hexanes/EtOAc as the eluent gave 231 mg (44%) of the title compound as a colourless oil.

TLC $R_f = 0.59$ (20/1 hexanes/EtOAc);

^{1}H NMR (400 MHz, $CDCl_3$): δ = 7.68 (d, *J* = 8.1 Hz, 1H), 7.36 (dd, *J* = 8.1, 7.1 Hz, 1H), 7.33 – 7.31 (m, 1H), 7.28 (dd, *J* = 7.1, 1.3 Hz, 1H), 7.17 (s, 1H), 6.99 – 6.96 (m, 2H), 6.95 – 6.91 (m, 1H), 3.95 (s, 3H), 3.83 (s, 3H), 1.07 – 1.05 (m, 9H), 1.00 – 0.97 (m, 9H), 0.25 – 0.23 (m, 6H), 0.16 – 0.14 (m, 6H);

^{13}C{^{1}H} NMR (101 MHz, $CDCl_3$): δ = 151.5, 150.7, 145.7, 144.3, 139.0, 134.9, 130.6, 127.8, 125.8, 124.9, 124.0, 122.4, 120.8, 115.0, 114.0, 107.0, 55.6, 55.5, 25.94 (3C), 25.86 (3C), 18.69, 18.65, -4.45 (2C), -4.47 (2C);

IR (KBr): ṽ = 3045, 2997, 2952, 2929, 2885, 2856, 1622, 1603, 1574, 1502, 1473, 1462, 1265, 1254, 1201, 1159, 1124, 1105, 1038, 1028, 974, 908, 866, 839, 783, 750, 735, 683 cm^{-1};

HRMS (ESI) m/z: $[M+H]^+$ Calcd for $C_{30}H_{45}O_4Si_2^+$: 525.2851; Found: 525.2840.

Ethyl 2-((8-(4-(2-ethoxy-2-oxoethoxy)-3-methoxyphenyl)-3-methoxynaphthalen-2-yl)oxy)acetate (3f)

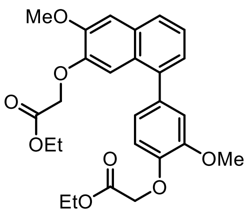

The title compound was prepared from ethyl 2-(4-allyl-2-methoxyphenoxy)acetate[19] (**1f**, 2 mmol, 501 mg) and DDQ (2.6 mmol, 590 mg) according to the General Procedure A. Column chromatography on silica gel using 3/2 hexanes/EtOAc as the eluent gave 203 mg (43%) of the title compound as a pale yellow solid (^{1}H NMR showed 94% purity).

TLC $R_f = 0.44$ (1/1 hexanes/EtOAc);

M.p. 102–104 °C (from hexanes/EtOAc);

^{1}H NMR (400 MHz, $CDCl_3$): δ = 7.68 (d, *J* = 8.1 Hz, 1H), 7.38 (dd, *J* = 8.1, 7.1 Hz, 1H), 7.26 (dd, *J* = 7.1, 1.3 Hz, 1H), 7.20 (s, 1H), 7.17 (s, 1H), 7.01 (d, *J* = 1.9 Hz, 1H), 6.97 (dd, *J* = 8.2, 1.9 Hz, 1H), 6.93 (d, *J* = 8.2 Hz, 1H), 4.76 (s, 2H), 4.61 (s, 2H), 4.30 (q, *J* = 7.1 Hz, 2H), 4.19 (q, *J* = 7.1 Hz, 2H), 4.01 (s, 3H), 3.90 (s, 3H), 1.32 (t, *J* = 7.1 Hz, 3H), 1.22 (t, *J* = 7.1 Hz, 3H);

^{13}C{^{1}H} NMR (101 MHz, $CDCl_3$): δ = 169.1, 168.5, 149.6, 149.5, 147.8, 146.7, 138.7, 135.4, 130.5, 127.0, 126.0, 125.5, 124.5, 121.9, 114.2, 114.0, 107.4, 107.3, 66.8, 66.2, 61.44, 61.43, 56.1, 56.0, 14.3, 14.2;

[18] Gamre, S.; Tyagi, M.; Chatterjee, S.; Patro, B. S.; Chattopadhyay, S.; Goswami, D. *J. Nat. Prod.* **2021**, *84*, 352–363.
[19] Labarrios, F.; Garduño, L.; Vidal, M. D. R.; Garcia, R.; Salazar, M.; Martinez, E.; Diaz, F.; Chamorro, G.; Tamariz, J. *J. Pharm. Pharmacol.* **1999**, *51*, 1–7.

IR (KBr): ṽ = 3082, 3041, 3016, 2993, 2980, 2960, 2949, 2929, 2912, 2873, 2837, 1755, 1622, 1601, 1508, 1477, 1441, 1381, 1242, 1192, 1167, 1153, 1111, 1076, 1014, 860, 849, 793, 769 cm^{-1};

HRMS (ESI) m/z: $[M+Na]^+$ Calcd for $C_{26}H_{28}O_8Na^+$: 491.1676; Found: 491.1660.

8-(4-Hydroxy-3-methoxyphenyl)-3-methoxynaphthalen-2-ol (oleralignan B, 3g)

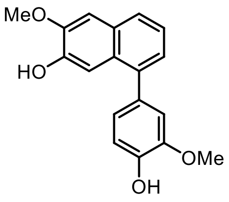

The title compound was prepared from eugenol (**1g**, 2 mmol, 328 mg) according to the General Procedure A and DDQ (2.2 mmol, 499 mg, added at 0 °C). Column chromatography on silica gel using 7/3 hexanes/EtOAc as the eluent gave 35 mg (12%) of the title compound as a pale yellow solid.

TLC R_f = 0.27 (7/3 hexanes/EtOAc);

M.p. 198–200 °C (from MeOH);

^{1}H NMR (400 MHz, MeOD): δ = 7.63 (d, *J* = 8.1 Hz, 1H), 7.26 (dd, *J* = 8.1, 7.1 Hz, 1H), 7.24 (s, 1H), 7.23 (s, 1H), 7.16 (dd, *J* = 7.1, 1.2 Hz, 1H), 6.97 (d, *J* = 1.9 Hz, 1H), 6.91 (d, *J* = 8.0 Hz, 1H), 6.86 (dd, *J* = 8.0, 1.9 Hz, 1H), 3.97 (s, 3H), 3.85 (s, 3H);

^{1}H NMR (400 MHz, DMSO-d_6): δ = 9.46 (s, 1H), 9.09 (s, 1H), 7.67 (d, *J* = 8.0 Hz, 1H), 7.31 (s, 1H), 7.27 (dd, *J* = 8.0, 6.9 Hz, 1H), 7.23 (s, 1H), 7.15 (d, *J* = 6.9 Hz, 1H), 6.96 (d, *J* = 1.8 Hz, 1H), 6.91 (d, *J* = 8.0 Hz, 1H), 6.83 (dd, *J* = 8.0, 1.8 Hz, 1H), 3.89 (s, 3H), 3.80 (s, 3H);

^{13}C{^{1}H} NMR (101 MHz, MeOD): δ = 149.9, 148.7, 147.8, 146.7, 139.7, 134.5, 131.1, 129.2, 126.9, 125.9, 124.1, 123.6, 116.1, 114.6, 109.3, 107.7, 56.5, 56.2;

^{13}C{^{1}H} NMR (101 MHz, DMSO-d_6): δ = 148.8, 147.4, 147.2, 145.8, 137.7, 131.9, 129.0, 127.3, 125.6, 124.5, 122.8, 122.0, 115.4, 113.8, 107.9, 107.1, 55.7, 55.4;

IR (KBr): ṽ = 3444, 3365, 3080, 3049, 3008, 2958, 2929, 2906, 2835, 1628, 1603, 1593, 1508, 1460, 1444, 1387, 1367, 1298, 1259, 1244, 1227, 1190, 1165, 1149, 1122, 1097, 1030, 1018, 856, 835, 827, 787, 769, 748 cm^{-1};

HRMS (ESI) m/z: $[M+H]^+$ Calcd for $C_{18}H_{17}O_4^+$: 297.1121; Found: 297.1120.

The recorded spectroscopic values agree with the previously reported data.[20]

General Procedure B ($FeCl_3$ as the additive)

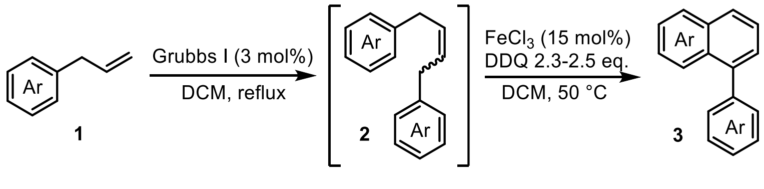

To a solution of 1st generation Grubbs catalyst (0.06 mmol, 49 mg) in dry degassed DCM (4 mL) under argon atmosphere was added allylbenzene **1** (2 mmol), and the reaction mixture was refluxed for 24 h (oil bath). After that, the mixture was cooled down to room temperature (25 °C) and diluted with dry DCM to 20 mL. Next, anhydrous $FeCl_3$ (0.15 mmol, 24 mg) was added to the solution under argon,

[20] Duan, Y.; Ying, Z.; He, F.; Ying, X.; Jia, L.; Yang, G. *Fitoterapia* **2021**, *153*, 104993.

and DDQ (2.3–2.5 mmol) was then carefully added in one portion. The resulting mixture was heated at 50 °C under argon atmosphere for 12 h (oil bath). Upon reaction completion (TLC monitoring), the mixture was filtered through a pad of Celite®, and the filtrate was treated with Et_3N (3 mmol, 0.42 mL). The volatiles were removed under reduced pressure and the residue was subjected to column chromatography to afford the desired lignan **3**.

8-(4-Acetoxy-3-methoxyphenyl)-3-methoxynaphthalen-2-yl acetate (3h)

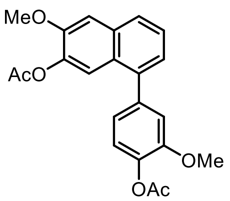

The title compound was prepared from eugenol acetate (**1h**, 2 mmol, 412 mg) and DDQ (2.5 mmol, 568 mg) according to the General Procedure B. Column chromatography on silica gel using 4/1 hexanes/EtOAc as the eluent gave 189 mg (50%) of the title compound as a white solid.

TLC R_f = 0.45 (7/3 hexanes/EtOAc);

M.p. 155 °C (from hexanes/EtOAc);

^{1}H NMR (400 MHz, $CDCl_3$) δ = 7.76 (d, *J* = 8.2, 1H), 7.56 (s, 1H), 7.47 (dd, *J* = 8.2, 7.1 Hz, 1H), 7.31 (dd, *J* = 7.1, 1.1 Hz, 1H), 7.28 (s, 1H), 7.14 (d, *J* = 7.9 Hz, 1H), 7.06 (d, *J* = 1.8 Hz, 1H), 7.04 (dd, *J* = 7.9, 1.8 Hz, 1H), 3.96 (s, 3H), 3.84 (s, 3H), 2.37 (s, 3H), 2.33 (s, 3H);

^{13}C{^{1}H} NMR (101 MHz, $CDCl_3$) δ = 169.3 (2C), 150.9, 150.4, 140.6, 139.5, 139.4, 139.2, 133.3, 126.8, 126.5, 125.8, 125.4, 122.7, 122.3, 119.0, 114.4, 107.7, 56.1, 56.0, 20.9, 20.8;

IR (KBr): ṽ = 3080, 3051, 3018, 2999, 2939, 2918, 2841, 1759, 1630, 1599, 1508, 1473, 1443, 1406, 1364, 1314, 1252, 1213, 1196, 1169, 1152, 1122, 1097, 1032, 1018, 917, 862, 839, 800, 760, 727 cm^{-1};

HRMS (ESI) m/z: $[M+Na]^+$ Calcd for $C_{22}H_{20}O_6Na^+$: 403.1152; Found: 403.1164.

5-(2,3-Dimethoxyphenyl)-1,2-dimethoxynaphthalene (3i)

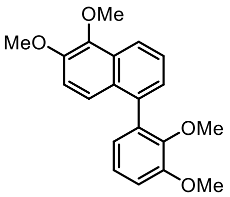

The title compound was prepared from 1-allyl-2,3-dimethoxybenzene[21] (**1i**, 2 mmol, 356 mg) and DDQ (2.3 mmol, 522 mg) according to the General Procedure B. The reaction was stopped after 6 h. Column chromatography on silica gel using 20/1 hexanes/EtOAc as the eluent gave 137 mg (42%) of the title compound as colourless crystals.

TLC R_f = 0.46 (4/1 hexanes/EtOAc);

M.p. 95–97 °C (from MeOH);

^{1}H NMR (400 MHz, $CDCl_3$): δ = 8.18 (d, *J* = 8.4 Hz, 1H), 7.52 (dd, *J* = 8.4, 7.0 Hz, 1H), 7.41 (d, *J* = 9.3 Hz, 1H), 7.31 (d, *J* = 7.0 Hz, 1H), 7.20 (d, *J* = 9.3 Hz, 1H), 7.15 (d, *J* = 8.2, 7.6 Hz, 1H), 7.02 (dd, *J* = 8.2, 1.4 Hz, 1H), 6.89 (dd, *J* = 7.6, 1.4 Hz, 1H), 4.04 (s, 3H), 3.97 (s, 3H), 3.95 (s, 3H), 3.47 (s, 3H);

^{13}C{^{1}H} NMR (101 MHz, $CDCl_3$): δ = 152.9, 148.2, 147.3, 142.8, 136.5, 135.0, 129.4, 128.4, 125.7, 125.5, 123.9, 123.8, 123.1, 121.1, 114.9, 111.9, 61.2, 61.0, 56.9, 56.0;

[21] Lahiri, S.; Ramarao, C.; Rao, B. V.; Rama Rao, A. V.; Chorghade, M. S. *Org. Process Res. Dev.* **1999**, *3*, 71–72.

IR (KBr): ṽ = 3072, 3003, 2929, 2900, 2837, 2821, 1616, 1589, 1574, 1510, 1477, 1446, 1423, 1400, 1385, 1367, 1323, 1308, 1271, 1257, 1230, 1171, 1120, 1074, 1039, 997, 933, 850, 800, 791, 768, 752, 744, 708 cm^{-1};

HRMS (ESI) m/z: $[M+Na]^+$ Calcd for $C_{20}H_{20}O_4Na^+$: 347.1254; Found: 347.1265.

6-Bromo-1-(3-bromo-4-methoxyphenyl)-7-methoxynaphthalene (3j)

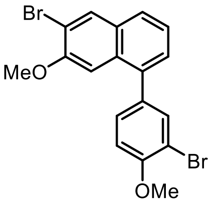

The title compound was prepared from 4-allyl-2-bromo-1-methoxybenzene (**1j**, 2 mmol, 454 mg) and DDQ (2.3 mmol, 522 mg, added at 0 °C) according to the General Procedure B. Column chromatography on silica gel using 97/3 hexanes/EtOAc as the eluent gave 89 mg (21%) of the title compound as a white solid. This product was found to be contaminated with an unidentified inseparable impurity (^{1}H NMR showed 92% purity).

TLC R_f = 0.64 (4/1 hexanes/EtOAc);

^{1}H NMR (400 MHz, $CDCl_3$): δ = 8.11 (s, 1H), 7.74 – 7.67 (m, 2H), 7.43 – 7.34 (m, 3H), 7.21 (s, 1H), 7.04 (d, *J* = 8.4 Hz, 1H), 3.99 (s, 3H), 3.84 (s, 3H);

$^{13}C\{^1H\}$ NMR (101 MHz, $CDCl_3$): δ = 155.4, 154.0, 137.4, 134.7, 134.4, 132.8, 131.9, 130.1, 129.9, 128.0, 126.7, 124.3, 113.6, 111.9, 111.8, 105.0, 56.5, 56.3.

The recorded spectroscopic values agree with the previously reported data.[22]

1-Bromo-8-(3-bromo-4,5-dimethoxyphenyl)-2,3-dimethoxynaphthalene (3k)

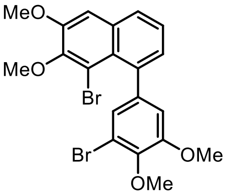

The title compound was prepared from 5-allyl-1-bromo-2,3-dimethoxybenzene (**1k**, 2 mmol, 514 mg) and DDQ (2.3 mmol, 522 mg, added at 0 °C) according to the General Procedure B. Column chromatography on silica gel using 9/1 hexanes/EtOAc as the eluent afforded 206 mg (43%) of the title compound as a white solid.

TLC R_f = 0.61 (4/1 hexanes/EtOAc);

M.p. 100 °C (from MeOH);

^{1}H NMR (400 MHz, $CDCl_3$): δ = 7.75 (dd, *J* = 8.1, 1.4 Hz, 1H), 7.39 (dd, *J* = 8.1, 7.1 Hz, 1H), 7.28 (dd, *J* = 7.1, 1.4 Hz, 1H), 7.23 (s, 1H), 7.12 (d, *J* = 1.9 Hz, 1H), 6.80 (d, *J* = 1.9 Hz, 1H), 4.01 (s, 3H), 3.94 (s, 3H), 3.88 (s, 3H), 3.83 (s, 3H);

$^{13}C\{^1H\}$ NMR (101 MHz, $CDCl_3$): δ = 152.7, 152.1, 148.6, 145.7, 140.4, 138.6, 133.1, 129.2, 127.8, 126.2, 125.4, 124.9, 116.7, 114.9, 114.4, 108.0, 61.0, 60.5, 56.3, 56.0;

IR (KBr): ṽ = 3049, 3006, 2937, 2871, 2850, 2829, 1606, 1593, 1556, 1485, 1464, 1415, 1363, 1309, 1290, 1246, 1227, 1147, 1109, 1047, 1030, 997, 908, 852, 818, 791, 758, 739, 725, 685 cm^{-1};

HRMS (ESI) m/z: $[M+Na]^+$ Calcd for $C_{20}H_{18}Br_2O_4Na^+$: 502.9464; Found: 502.9456.

[22] Xu, Z.; Weng, J.; Wei, L.; Huang, T.; Wu, J.; Chen, Z. *J. Org. Chem.* **2024**, *89*, 2264–2271.

8-(4-Acetoxy-3-bromo-5-methoxyphenyl)-1-bromo-3-methoxynaphthalen-2-yl acetate (3l)

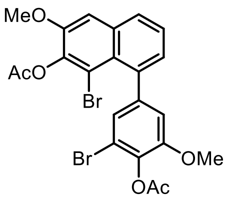

The title compound was prepared from 4-allyl-2-bromo-6-methoxyphenyl acetate (**1l**, 2 mmol, 570 mg) and DDQ (2.5 mmol, 568 mg) according to the General Procedure B. Column chromatography on silica gel using 4/1 hexanes/EtOAc as the eluent gave 126 mg (23%) of the title compound as a white solid.

TLC R_f = 0.46 (7/3 hexanes/EtOAc);

M.p. 185–200 °C (from hexanes/EtOAc);

^{1}H NMR (400 MHz, $CDCl_3$): δ = 7.80 (dd, *J* = 8.2, 1.4 Hz, 1H), 7.45 (dd, *J* = 8.2, 7.1 Hz, 1H), 7.34 (dd, *J* = 7.1, 1.4 Hz, 1H), 7.27 (s, 1H), 7.17 (d, *J* = 1.8 Hz, 1H), 6.84 (d, *J* = 1.8 Hz, 1H), 3.97 (s, 3H), 3.79 (s, 3H), 2.40 (s, 3H), 2.36 (s, 3H);

^{13}C{^{1}H} NMR (101 MHz, $CDCl_3$): δ = 168.2, 168.1, 151.5, 150.4, 142.3, 140.6, 138.6, 137.1, 134.3, 129.4, 128.2, 126.1, 125.7, 125.0, 116.4, 114.9, 114.2, 108.0, 56.5, 56.3, 20.68, 20.66;

IR (KBr): ṽ = 3082, 3016, 2949, 2843, 1768, 1614, 1591, 1566, 1468, 1429, 1362, 1277, 1248, 1186, 1147, 1107, 1043, 1011, 903, 850, 760 cm^{-1};

HRMS (ESI) m/z: $[M+Na]^+$ Calcd for $C_{22}H_{18}Br_2O_6Na^+$: 560.9344; Found: 560.9336.

1,2,3-Trimethoxy-5-(2,3,4-trimethoxyphenyl)naphthalene (3m)

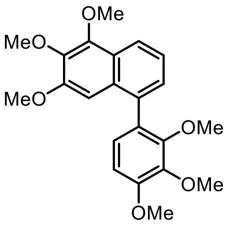

The title compound was prepared from 1-allyl-2,3,4-trimethoxybenzene (**1m**, 2 mmol, 417 mg) and DDQ (2.3 mmol, 522 mg, added at 0 °C) according to the General Procedure B. The reaction was stopped after 4 h. Column chromatography on silica gel using 9/1 hexanes/EtOAc as the eluent gave 112 mg (36%) of the title compound as colourless crystals.

TLC R_f = 0.41 (7/3 hexanes/EtOAc);

M.p. 80 °C (from MeOH);

^{1}H NMR (400 MHz, $CDCl_3$): δ = 8.08 (d, *J* = 8.2 Hz, 1H), 7.40 (dd, *J* = 8.2, 7.0 Hz, 1H), 7.32 (dd, *J* = 7.0, 1.3 Hz, 1H), 6.97 (d, *J* = 8.5 Hz, 1H), 6.78 (d, *J* = 8.5 Hz, 1H), 6.74 (s, 1H), 4.07 (s, 3H), 3.98 (s, 3H), 3.96 (s, 3H), 3.95 (s, 3H), 3.77 (s, 3H), 3.54 (s, 3H);

^{13}C{^{1}H} NMR (101 MHz, $CDCl_3$): δ = 153.4, 152.8, 152.1, 147.9, 142.3, 140.8, 135.3, 129.9, 127.7, 127.5, 126.0, 124.8, 123.4, 121.3, 107.3, 101.3, 61.6, 61.3, 61.23, 61.20, 56.2, 55.8;

IR (KBr): ṽ = 3072, 2998, 2962, 2935, 2864, 2841, 2829, 1618, 1603, 1580, 1489, 1460, 1417, 1375, 1319, 1286, 1267, 1238, 1232, 1205, 1192, 1113, 1088, 1043, 1024, 999, 958, 903, 858, 837, 808, 762, 748, 712, 690 cm^{-1};

HRMS (ESI) m/z: $[M+Na]^+$ Calcd for $C_{22}H_{24}O_6Na^+$: 407.1465; Found: 407.1464.

1,2,3-Trimethoxy-8-(3,4,5-trimethoxyphenyl)naphthalene (3n)

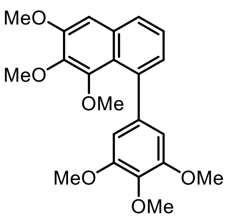

The title compound was prepared from elemicin[23,24] (**1n**, 2 mmol, 417 mg) and DDQ (2.3 mmol, 522 mg, added at 0 °C) according to the General Procedure B. Column chromatography on silica gel using 7/3 hexanes/EtOAc as the eluent gave 86 mg (22%) of the title compound as colourless needles.

TLC R_f = 0.38 (7/3 hexanes/EtOAc);

M.p. 119–121 °C (from MeOH);

^{1}H NMR (400 MHz, $CDCl_3$): δ = 7.70 (d, *J* = 8.2 Hz, 1H), 7.36 (dd, *J* = 8.1, 7.1 Hz, 1H), 7.17 (dd, *J* = 7.1, 1.2 Hz, 1H), 7.03 (s, 1H), 6.60 (s, 2H), 4.00 (s, 3H), 3.92 (s, 3H), 3.90 (s, 3H), 3.85 (s, 6H), 3.38 (s, 3H);

^{13}C{^{1}H} NMR (101 MHz, $CDCl_3$): δ = 152.9, 151.9 (2C), 149.7, 142.5, 140.4, 138.2, 136.5, 132.0, 127.4, 126.8, 124.7, 122.6, 106.6 (2C), 103.2, 61.14, 61.12, 61.0, 56.2 (2C), 55.9;

IR (KBr): ṽ = 3001, 2958, 2933, 2835, 1614, 1576, 1506, 1477, 1462, 1446, 1408, 1356, 1265, 1232, 1207, 1178, 1153, 1117, 1101, 1032, 1003, 978, 953, 889, 849, 822, 781, 737, 712, 661 cm^{-1};

HRMS (ESI) m/z: $[M+H]^+$ Calcd for $C_{22}H_{25}O_6^+$: 385.1646; Found: 385.1651.

Methyl 8-(3,4-dimethoxy-5-(methoxycarbonyl)phenyl)-2,3-dimethoxy-1-naphthoate (3o)

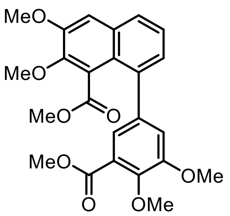

The title compound was prepared from methyl 5-allyl-2,3-dimethoxybenzoate[25] (**1o**, 2 mmol, 473 mg) and DDQ (2.5 mmol, 568 mg) according to the General Procedure B. Column chromatography on silica gel using 7/3 hexanes/EtOAc as the eluent gave 239 mg (54%) of the title compound as a white solid (^{1}H NMR showed 96% purity).

TLC R_f = 0.65 (1/1 hexanes/EtOAc);

M.p. 130 °C (from MeOH);

^{1}H NMR (400 MHz, $CDCl_3$): δ = 7.74 (dd, *J* = 8.3, 1.4 Hz, 1H), 7.41 (dd, *J* = 8.3, 7.1 Hz, 1H), 7.32 (d, *J* = 2.2 Hz, 1H), 7.29 (s, 1H), 7.22 (dd, *J* = 7.1, 1.4 Hz, 1H), 7.07 (d, *J* = 2.2 Hz, 1H), 4.01 (s, 3H), 3.97 (s, 3H), 3.91 – 3.88 (m, 6H), 3.87 (s, 3H), 3.29 (s, 3H);

^{13}C{^{1}H} NMR (101 MHz, $CDCl_3$): δ = 167.8, 166.7, 152.3, 151.6, 148.1, 148.1, 137.7, 137.0, 132.4, 128.5, 127.3, 125.33, 125.25, 125.1, 123.7, 123.3, 118.6, 109.6, 62.0, 61.6, 56.2, 55.9, 52.3, 51.8;

IR (KBr): ṽ = 3458, 3005, 2983, 2945, 2883, 2846, 2235, 1726, 1714, 1687, 1608, 1595, 1576, 1489, 1468, 1450, 1435, 1416, 1371, 1327, 1255, 1240, 1153, 1065, 1049, 1020, 995, 885, 854, 785, 725, 631 cm^{-1};

HRMS (ESI) m/z: $[M+Na]^+$ Calcd for $C_{24}H_{24}O_8Na^+$: 463.1363; Found: 463.1359.

[23] Patil, S. N.; Tilve, S. G. *Tetrahedron Lett.* **2016**, *57*, 3371–3375.
[24] Rudroff, F.; Rydz, J.; Ogink, F. H.; Fink, M.; Mihovilovic, M. D. *Adv. Synth. Catal.* **2007**, *349*, 1436–1444.
[25] Kim, K.; Miller, N. R.; Sulikowski, G. A.; Lindsley, C. W. *Bioorg. Med. Chem. Lett.* **2008**, *18*, 4467–4469.

1-Phenylnaphthalene (3p)

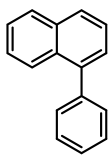

The title compound was prepared from allylbenzene (**1p**, 2 mmol, 236 mg) and DDQ (2.5 mmol, 568 mg) according to the General Procedure B, except after the addition of DDQ the resulting mixture was heated at 60 °C for 24 h (oil bath). Column chromatography on silica gel using hexanes as the eluent gave 108 mg (53%) of the title compound as a colourless oil.

TLC R_f = 0.49 (hexanes);

^{1}H NMR (400 MHz, $CDCl_3$): δ = 7.92 (d, *J* = 8.5 Hz, 2H), 7.88 (d, *J* = 8.2 Hz, 1H), 7.59 – 7.38 (m, 9H);

^{13}C{^{1}H} NMR (101 MHz, $CDCl_3$) δ = 140.9, 140.4, 133.9, 131.8, 130.2 (2C), 128.40, 128.39 (2C), 127.8, 127.4, 127.1, 126.17, 126.15, 125.9, 125.5.

The recorded spectroscopic values agree with the previously reported data.[26]

6-Methoxy-1-(3-methoxyphenyl)naphthalene (3s)

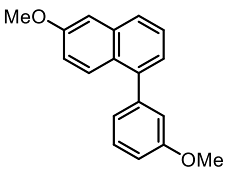

The title compound was prepared from 1-allyl-3-methoxybenzene[10,11,12] (**1s**, 2 mmol, 296 mg) and DDQ (2.5 mmol, 568 mg) according to the General Procedure B. Column chromatography on silica gel using 97/3 hexanes/EtOAc as the eluent gave 33 mg (13%) of the title compound as a colourless oil. This product was found to be contaminated with an unidentified inseparable impurity (^{1}H NMR showed 87% purity).

TLC R_f = 0.37 (20/1 hexanes/EtOAc);

^{1}H NMR (400 MHz, $CDCl_3$): δ = 7.84 (d, *J* = 9.3 Hz, 1H), 7.76 (dt, *J* = 8.2, 1.2 Hz, 1H), 7.48 (dd, *J* = 8.2, 7.1 Hz, 1H), 7.40 (dd, *J* = 8.3, 7.5 Hz, 1H), 7.30 (dd, *J* = 7.1, 1.2 Hz, 1H), 7.21 (d, *J* = 2.7 Hz, 1H), 7.10 (dd, *J* = 9.3, 2.7 Hz, 1H), 7.08 (ddd, *J* = 7.5, 1.6, 1.0 Hz, 1H), 7.04 (dd, *J* = 2.6, 1.6 Hz, 1H), 6.98 (ddd, *J* = 8.3, 2.6, 1.0 Hz, 1H), 3.95 (s, 3H), 3.86 (s, 3H);

^{13}C{^{1}H} NMR (101 MHz, $CDCl_3$): δ = 159.6, 157.6, 142.4, 140.3, 135.2, 129.3, 127.8, 127.2, 126.7, 126.1, 124.8, 122.7, 118.8, 115.7, 113.0, 106.2, 55.5, 55.4;

IR (KBr): ṽ = 3049, 2999, 2954, 2933, 2833, 1622, 1595, 1576, 1510, 1485, 1464, 1450, 1433, 1369, 1315, 1294, 1259, 1236, 1217, 1167, 1111, 1045, 980, 935, 847, 829, 779, 756, 704 cm^{-1};

HRMS (ESI) m/z: $[M+H]^+$ Calcd for $C_{18}H_{17}O_2^+$: 265.1223; Found: 265.1224.

The recorded spectroscopic values revise the previously reported data.[22,27,28]

[26] Kawamoto, T.; Sato, A.; Ryu, I. *Org. Lett.* **2014**, *16*, 2111–2113.
[27] Conte, V.; Elakkari, E.; Floris, B.; Mirruzzo, V.; Tagliatesta, P. *Chem. Commun.* **2005**, 1587–1588.
[28] Elakkari, E.; Floris, B.; Galloni, P.; Tagliatesta, P. *Eur. J. Org. Chem.* **2005**, 889–894.

2.3.2. Synthesis of lignans from the cross-metathesis products

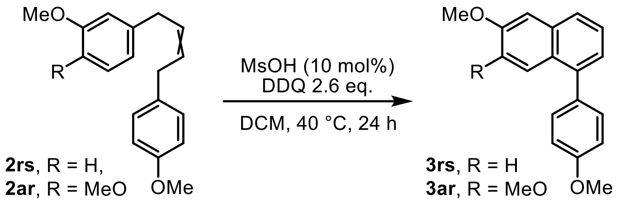

6-Methoxy-1-(4-methoxyphenyl)naphthalene (3rs)

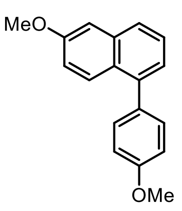

A solution of methanesulfonic acid in DCM (0.5 M, 0.1 mmol, 40 µL) and DDQ (2.6 mmol, 119 mg) were added to the solution of alkene **2rs** (0.2 mmol, 54 mg) in dry DCM (15 mL), and the resulting mixture was stirred at 40 °C for 24 h under air atmosphere (oil bath). After that, the volatiles were removed under reduced pressure and the residue was subjected to column chromatography on silica gel using 97/3 hexanes/Et_2O as the eluent to afford lignan **3rs** as a white solid (31 mg, 58%).

TLC R_f = 0.39 (20/1 hexanes/EtOAc);

^{1}H NMR (400 MHz, $CDCl_3$): δ = 7.84 (d, *J* = 9.3 Hz, 1H), 7.74 (d, *J* = 8.2 Hz, 1H), 7.48 (dd, *J* = 8.2, 7.1 Hz, 1H), 7.42 (d, *J* = 8.7 Hz, 2H), 7.27 (dd, *J* = 7.1, 1.3 Hz, 1H), 7.21 (d, *J* = 2.7 Hz, 1H), 7.10 (dd, *J* = 9.3, 2.7 Hz, 1H), 7.03 (d, *J* = 8.7 Hz, 2H), 3.95 (s, 3H), 3.90 (s, 3H);

^{13}C{^{1}H} NMR (101 MHz, $CDCl_3$): δ = 159.1, 157.6, 140.1, 135.3, 133.4, 131.2 (2C), 127.9, 127.4, 126.3, 126.2, 124.9, 118.7, 113.8 (2C), 106.3, 55.49, 55.45.

The recorded spectroscopic values agree with the previously reported data.[29]

6,7-Dimethoxy-1-(4-methoxyphenyl)naphthalene (3ar)

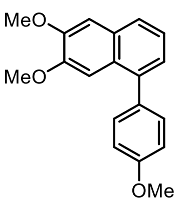

A solution of methanesulfonic acid in DCM (0.5 M, 0.1 mmol, 0.2 mL) and DDQ (2.6 mmol, 589 mg) were added to the solution of alkene **2ar** (1 mmol, 298 mg) in dry DCM (15 ml), and the resulting mixture was stirred at 40 °C for 24 h under air atmosphere (oil bath). After that, the volatiles were removed under reduced pressure and the residue was subjected to column chromatography on silica gel using 10/1 hexanes/EtOAc as the eluent to afford lignan **3ar** as a white solid (107 mg, 36%).

TLC R_f = 0.43 (4/1 hexanes/EtOAc);

M.p. 92 °C (from MeOH);

^{1}H NMR (400 MHz, $CDCl_3$): δ = 7.69 (d, *J* = 8.1 Hz, 1H), 7.46 (d, *J* = 8.8 Hz, 2H), 7.39 (dd, *J* = 8.1, 7.1 Hz, 1H), 7.28 (dd, *J* = 7.1, 1.3 Hz, 1H), 7.28 (s, 1H), 7.20 (s, 1H), 7.05 (d, *J* = 8.8 Hz, 2H), 4.03 (s, 3H), 3.90 (s, 3H), 3.85 (s, 3H);

[29] Shen, H.-C.; Pal, S.; Lian, J.-J.; Liu, R.-S. *J. Am. Chem. Soc.* **2003**, *125*, 15762–15763.

$^{13}C\{^1H\}$ NMR (101 MHz, $CDCl_3$): δ = 158.9, 149.5, 149.3, 138.5, 133.6, 130.9 (2C), 129.8, 127.5, 125.8, 125.5, 124.0, 113.8 (2C), 106.7, 104.9, 55.9, 55.7, 55.3;

IR (KBr): ṽ = 3022, 3001, 2953, 2931, 2904, 2833, 1622, 1608, 1587, 1504, 1481, 1456, 1435, 1425, 1391, 1284, 1254, 1240, 1219, 1173, 1157, 1105, 1032, 1005, 943, 858, 834, 807, 789, 768, 750 cm^{-1};

HRMS (ESI) m/z: $[M+H]^+$ Calcd for $C_{19}H_{19}O_3{}^+$: 295.1329; Found: 295.1328.

2.3.3. Gram-scale synthesis of oleralignan B

Gram-scale synthesis of oleralignan B diacetate (3h)

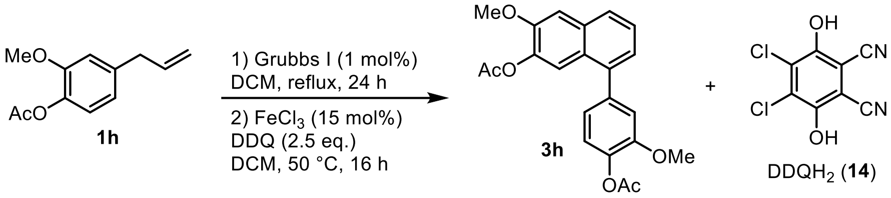

Caution! Large quantities of DDQ can release a significant amount of hydrogen cyanide by reacting with water![30]

For the gram-scale synthesis of oleralignan B (**3g**) depicted in Figure S1, diacetate intermediate **3h** was prepared following the modified General Procedure B. To a solution of 1st generation Grubbs catalyst (0.17 mmol, 140 mg, 1 mol%) in dry degassed DCM (40 mL) under argon atmosphere was added eugenol acetate (**1h**, 17 mmol, 3.506 g), and the reaction mixture was refluxed for 24 h (oil bath). After that, the mixture was cooled down to room temperature (25 °C) and diluted with dry DCM to 100 mL under argon atmosphere. Next, DDQ (21.25 mmol, 4.83 g) and anhydrous $FeCl_3$ (1.28 mmol, 207 mg) were added together in a single portion to this solution while stirring, and the resulting mixture was heated at reflux for 16 h (oil bath). After that, the resulting mixture was poured into a 1 L beaker containing 100 mL of cold water, treated with aqueous Na_2SO_3 (160 mg in 10 mL H_2O), aqueous Na_2CO_3 (2.25 g in 100 mL H_2O) and stirred for 0.5 h. After that, the organic phase was separated, and the aqueous layer was extracted with DCM (3×50 mL). The combined organic extracts were dried over anhydrous Na_2SO_4 and concentrated under reduced pressure. Column chromatography of the residue on silica gel using 4/1 hexanes/EtOAc as the eluent afforded diacetate **3h** as a white solid (1.382 g, 43% yield). The aqueous layer obtained in the extraction step was carefully acidified with concentrated aqueous HCl until pH = 2 leading to the formation of a white precipitate. This solid was collected by filtration, washed with water, and dried *in vacuo* to give 4.089 g of $DDQH_2$ (**14**, 84% yield based on DDQ).

4,5-Dichloro-3,6-dihydroxyphthalonitrile (14)

^{1}H NMR (400 MHz, DMSO-d_6): δ = 7.33 (s, 2H);

^{13}C{^{1}H} NMR (101 MHz, DMSO-d_6) δ = 150.9 (2C), 129.2 (2C), 113.7 (2C), 101.7 (2C).

The recorded spectroscopic values agree with the previously reported data.[31]

---

[30] Buckle, D. R.; Collier, S. J.; McLaws, M. D. 2,3-Dichloro-5,6-Dicyano-1,4-Benzo-Quinone. In *Encyclopedia of Reagents for Organic Synthesis*; John Wiley & Sons, Ltd., Ed.; John Wiley & Sons, Ltd: Chichester, 2005; p rd114.

[31] Nakayama, K.; Okada, Y. *J. Org. Chem.* **2023**, *88*, 5913–5922.

Base-mediated hydrolysis of diacetate 3h into oleralignan B (3g)

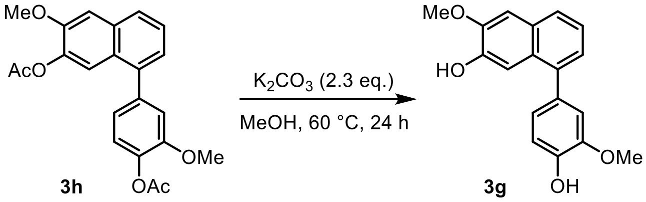

A suspension of diacetate **3h** (3.63 mmol, 1.382 g) and K_2CO_3 (8.36 mmol, 1.150 g, 2.3 eq.) in MeOH (60 mL) was heated at 60 °C with stirring for 24 h under argon atmosphere (oil bath). Upon completion (TLC monitoring), the resulting solution was cooled to room temperature and carefully acidified with AcOH (1 mL). After stirring for additional 0.5 h the saturated red colour gradually disappeared. Next, the mixture was concentrated under reduced pressure and diluted with EtOAc/H_2O (1/1, 200 mL). The organic layer was separated, and the aqueous layer was extracted with EtOAc (3×100 mL). The combined organic extracts were washed with saturated aqueous $NaHCO_3$ and brine (50 mL each), dried over anhydrous Na_2SO_4, and concentrated under reduced pressure to afford the crude product as a brownish powder (1.070 g). This material was subjected to flash column chromatography on silica gel using 1/1 hexanes/EtOAc as the eluent to give oleralignan B (**3g**) as a beige solid (1.018 g, 95% yield, 40% overall yield from **1h**).

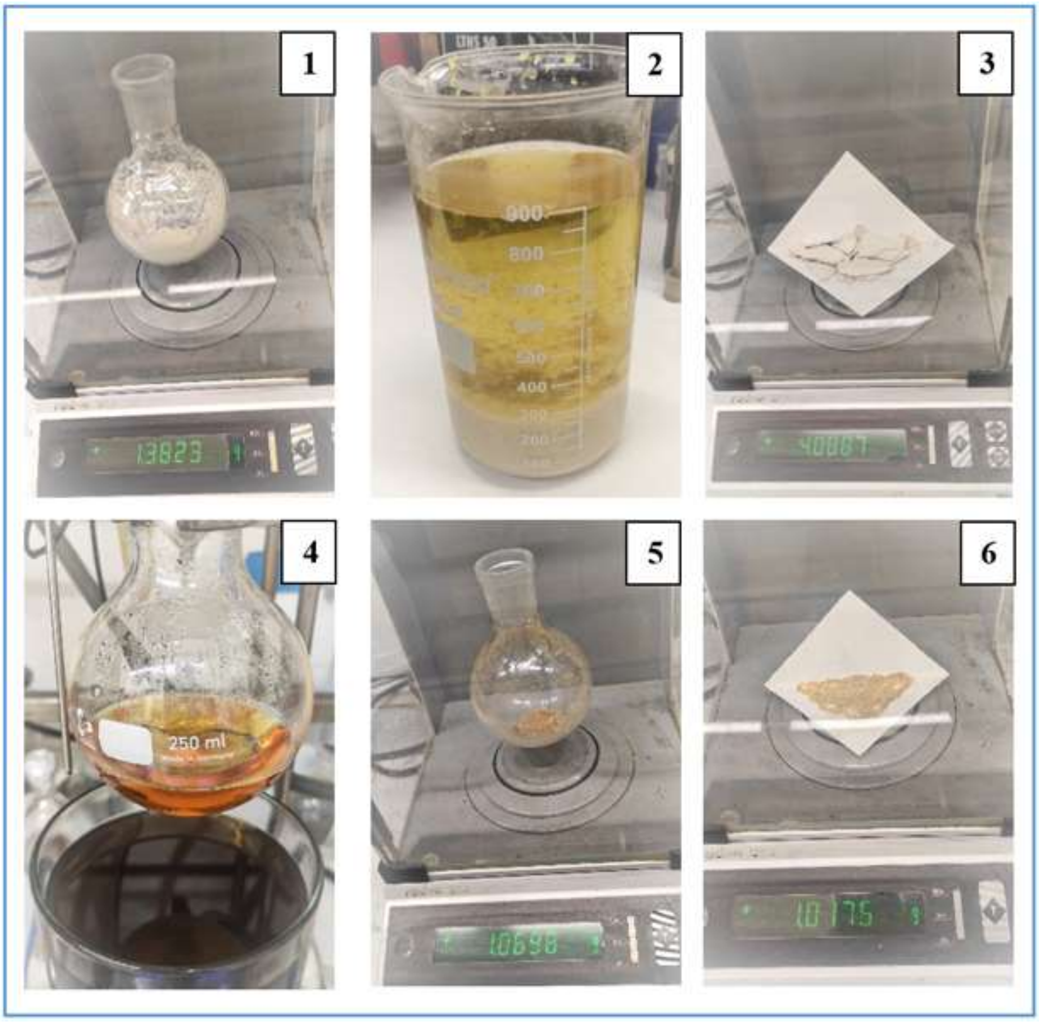

Figure S1. The progress of the oleralignan B synthesis: 1) isolated oleralignan B diacetate; 2) the precipitate of hydroquinone **14**; 3) dried hydroquinone **14**; 4) the reaction mixture containing oleralignan B after quenching; 5) crude oleralignan B after extraction; 6) pure oleralignan B.

2.4. One-pot synthesis of benzyl styryl ketones

General procedure C

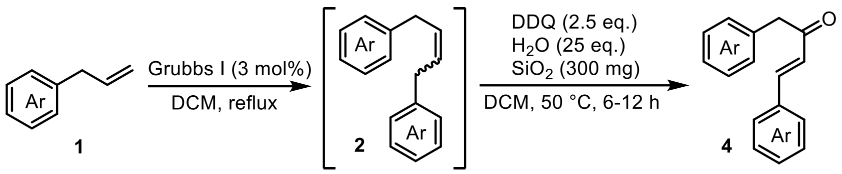

To a solution of 1st generation Grubbs catalyst (0.06 mmol, 49 mg) in dry degassed DCM (4 mL) under argon atmosphere was added allylbenzene **1** (2 mmol) and the reaction mixture was refluxed for 24 h (oil bath). The reaction mixture was cooled to room temperature (25 °C) and diluted with DCM (11 mL) to a total volume of 15 mL. Water (25 mmol, 25 eq., 0.45 mL) and silica gel (5 mmol, 5 eq., 300 mg) were then introduced, and the resulting heterogeneous mixture was cooled to 0 °C. DDQ (2.5 mmol, 2.5 eq., 568 mg) was carefully added in a single portion, and the stirring was continued at 50 °C under air atmosphere (oil bath) until the reaction completion (TLC monitoring). Upon completion, the reaction mixture was filtered through a pad of Celite®, washed with 1/1 hexanes/EtOAc (10 mL), and the filtrate was concentrated under reduced pressure. The residue was subjected to column chromatography on silica gel to afford desired ketone **4**.

(*E*)-1,4-Bis(3,4-dimethoxyphenyl)but-3-en-2-one (4a)

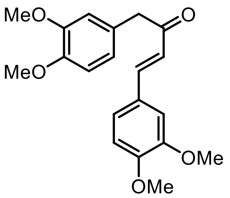

The title compound was prepared from methyl eugenol (**1a**, 2 mmol, 356 mg) according to the General Procedure C. Column chromatography on silica gel using 7/1 hexanes/EtOAc as the eluent gave lignan **3a** as a colourless solid (107 mg, 33%). Further elution with 3/2 hexanes/EtOAc afforded enone **4a** as a yellowish solid (68 mg, 20%).

TLC R_f = 0.52 (1/1 hexanes/EtOAc);

M.p. 114 °C (from hexanes/EtOAc);

^{1}H NMR (400 MHz, $CDCl_3$): δ = 7.58 (d, *J* = 16.0 Hz, 1H), 7.11 (dd, *J* = 8.3, 2.0 Hz, 1H), 7.03 (d, *J* = 2.0 Hz, 1H), 6.89 – 6.77 (m, 4H), 6.65 (d, *J* = 16.0 Hz, 1H), 3.91 (s, 3H), 3.90 (s, 3H), 3.88 – 3.86 (m, 5H), 3.86 (s, 3H);

^{13}C{^{1}H} NMR (101 MHz, $CDCl_3$): δ = 197.7, 151.6, 149.4, 149.3, 148.2, 143.6, 127.5, 127.2, 123.3, 123.2, 121.8, 112.6, 111.5, 111.2, 110.1, 56.13, 56.07, 56.03, 56.02, 47.9;

IR (KBr): ṽ = 3010, 2962, 2922, 2841, 1668, 1593, 1510, 1468, 1448, 1421, 1312, 1257, 1227, 1157, 1134, 1020, 972, 874, 801. 779, 758 cm^{-1};

HRMS (ESI) m/z: $[M+H]^+$ Calcd for $C_{20}H_{23}O_5^+$: 343.1540; Found: 343.1534.

(*E*)-(3-Oxobut-1-ene-1,4-diyl)bis(2-methoxy-4,1-phenylene) diacetate (4h)

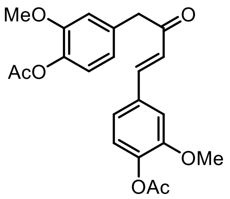

The title compound was prepared from eugenol acetate (**1h**, 2 mmol, 412 mg) according to the General Procedure C. Column chromatography on silica gel using 3/2 hexanes/EtOAc as the eluent afforded 149 mg (37%) of the title compound as white crystals.

TLC R_f = 0.57 (1/1 hexanes/EtOAc);

M.p. 165–175 °C (from MeOH);

^{1}H NMR (400 MHz, $CDCl_3$): δ = 7.58 (d, *J* = 16.0 Hz, 1H), 7.12 (dd, *J* = 8.1, 1.7 Hz, 1H), 7.09 (d, *J* = 1.6 Hz, 1H), 7.05 (d, *J* = 8.1 Hz, 1H), 7.00 (d, *J* = 8.0 Hz, 1H), 6.86 (d, *J* = 1.6 Hz, 1H), 6.83 (dd, *J* = 8.0, 1.7 Hz, 1H), 6.72 (d, *J* = 16.0 Hz, 1H), 3.92 (s, 2H), 3.85 (s, 3H), 3.82 (s, 3H), 2.32 (s, 3H), 2.30 (s, 3H);

^{13}C{^{1}H} NMR (101 MHz, $CDCl_3$): δ = 197.0, 169.2, 168.9, 151.6, 151.3, 143.1, 141.9, 138.9, 133.4, 133.3, 125.4, 123.4, 123.1, 121.8, 121.7, 113.6, 111.7, 56.1, 56.0, 48.1, 20.80, 20.76;

IR (KBr): ṽ = 3207, 3078, 3008, 2978, 2941, 2912, 2845, 1755, 1695, 1666, 1601, 1510, 1450, 1419, 1369, 1271, 1219, 1196, 1169, 1153, 1119, 1076, 1032, 1011, 906, 827, 764, 731 cm^{-1};

HRMS (ESI) m/z: $[M+Na]^+$ Calcd for $C_{22}H_{22}O_7Na^+$: 421.1258; Found: 421.1254.

(*E*)-1,4-Diphenylbut-3-en-2-one (4p)

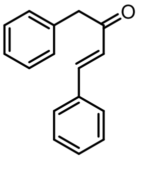

The title compound was prepared from allylbenzene (**1p**, 2 mmol, 236 mg) according to the General Procedure C. Column chromatography on silica gel using 9/1 hexanes/EtOAc as the eluent afforded 78 mg (35%) of the title compound as yellowish crystals.

TLC R_f = 0.39 (9/1 hexanes/EtOAc);

^{1}H NMR (400 MHz, $CDCl_3$): δ = 7.64 (d, *J* = 16.1 Hz, 1H), 7.58 – 7.47 (m, 2H), 7.41 – 7.32 (m, 5H), 7.31 – 7.26 (m, 3H), 6.79 (d, *J* = 16.1 Hz, 1H), 3.95 (s, 2H);

^{13}C{^{1}H} NMR (101 MHz, $CDCl_3$): δ = 197.4, 143.5, 134.53, 134.52, 130.7, 129.6 (2C), 129.0 (2C), 128.9 (2C), 128.5 (2C), 127.1, 125.3, 48.5.

The recorded spectroscopic values agree with the previously reported data.[32]

(*E*)-1,4-Bis(4-fluorophenyl)but-3-en-2-one (4q)

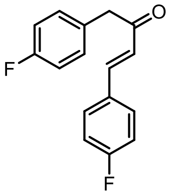

The title compound was prepared from 1-allyl-4-fluorobenzene (**1q**, 2 mmol, 272 mg) according to the General Procedure C. Column chromatography on silica gel using 9/1 hexanes/EtOAc as the eluent afforded 98 mg (38%) of the title compound as white crystals.

TLC R_f = 0.31 (9/1 hexanes/EtOAc);

[32] Kikuchi-Igarashi, K.; Tahara, Y.; Hirano, H.; Ambe, C.; Kinoshita, H.; Miura, K. *Org. Lett.* **2024**, *26*, 5689–5694.

^{1}H NMR (400 MHz, $CDCl_3$): δ = 7.59 (d, *J* = 16.0 Hz, 1H), 7.55 – 7.46 (m, 2H), 7.25 – 7.17 (m, 2H), 7.13 – 6.97 (m, 4H), 6.70 (d, *J* = 16.0 Hz, 1H), 3.91 (s, 2H);

^{13}C{^{1}H} NMR (101 MHz, $CDCl_3$): δ = 196.9, 165.5 (d, *J* = 251.4 Hz), 160.9 (d, *J* = 245.2 Hz), 142.3, 131.1 (d, *J* = 8.0 Hz, 2C), 130.7 (d, *J* = 3.4 Hz), 130.4 (d, *J* = 8.6 Hz, 2C), 130.1 (d, *J* = 3.3 Hz), 124.8 (d, *J* = 2.3 Hz), 116.3 (d, *J* = 22.0 Hz, 2C), 115.7 (d, *J* = 21.4 Hz, 2C), 47.5.

The recorded spectroscopic values agree with the previously reported data.[32]

(*E*)-1,4-Bis(4-methoxyphenyl)but-3-en-2-one (4r)

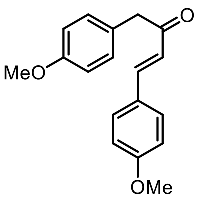

The title compound was prepared from estragole (**1r**, 2 mmol, 296 mg) according to the General Procedure C. Column chromatography on silica gel using 9/1 hexanes/EtOAc as the eluent afforded 104 mg (37%) of the title compound as white crystals. When the synthesis was performed in the presence of $FeCl_3{\cdot}6H_2O$ (0.15 mmol, 41 mg, 0.15 eq.), the product was obtained in a slightly higher yield (116 mg, 41%).

TLC R_f = 0.57 (7/3 hexanes/EtOAc);

^{1}H NMR (400 MHz, $CDCl_3$): δ = 7.58 (d, *J* = 16.0 Hz, 1H), 7.47 (d, *J* = 8.6 Hz, 2H), 7.18 (d, *J* = 8.6, 2H), 6.93 – 6.83 (m, 4H), 6.65 (d, *J* = 16.0 Hz, 1H), 3.85 (s, 2H), 3.83 (s, 3H), 3.79 (s, 3H);

^{13}C{^{1}H} NMR (101 MHz, $CDCl_3$): δ = 197.7, 161.7, 158.7, 143.1, 130.6 (2C), 130.2 (2C), 127.2, 126.8, 123.1, 114.5 (2C), 114.3 (2C), 55.5, 55.3, 47.6.

The recorded spectroscopic values agree with the previously reported data.[33]

[33] Buchbinder, N. W.; Nguyen, L. H.; Beck, O. N.; Bage, A. D.; Slebodnick, C.; Santos, W. L. *Org. Lett.* **2024**, *26*, 6136–6141.

2.5. Assessment of the possible reaction intermediates

2,3-Dichloro-5,8-bis(3,4-dimethoxyphenyl)-1,4-dioxo-1,4,5,8-tetrahydronaphthalene-4a,8a-dicarbonitrile (5a)

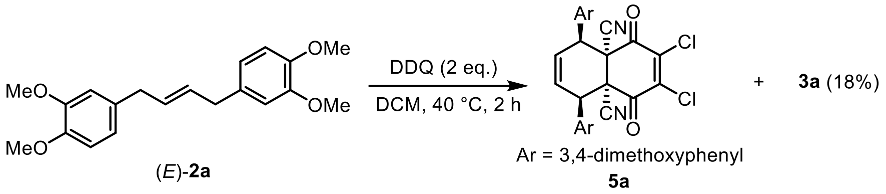

To a solution of (*E*)-**2a** (0.5 mmol, 164 mg) in DCM (5 mL) was added DDQ (1 mmol, 228 mg) and the reaction mixture was stirred at 40 °C for 2 h (oil bath). After that, the volatiles were removed under reduced pressure. Column chromatography of the residue on silica gel using 7/1 hexanes/EtOAc as the eluent provided 29 mg (18%) of **3a** as a colourless solid. Further elution with 2/1 hexanes/EtOAc as the eluent gave 103 mg (37%) of **5a** as a yellow solid.

TLC R_f = 0.30 (1/1 hexanes/EtOAc);

M.p. 190 °C, dec. (from hexanes/EtOAc);

^{1}H NMR (400 MHz, $CDCl_3$): δ = 6.90 – 6.85 (m, 4H), 6.84 – 6.81 (m, 2H), 6.19 (s, 2H), 4.33 (s, 2H), 3.90 (s, 6H), 3.89 (s, 6H);

^{13}C{^{1}H} NMR (101 MHz, $CDCl_3$): δ = 178.1 (2C), 150.0 (2C), 149.0 (2C), 143.6 (2C), 128.2 (2C), 126.8 (2C), 122.9 (2C), 113.3 (2C), 112.7 (2C), 111.1 (2C), 57.6 (2C), 56.1 (2C), 56.0 (2C), 43.9 (2C);

IR (KBr): ṽ = 3001, 2956, 2924, 2839, 1680, 1593, 1578, 1510, 1452, 1421, 1331, 1259, 1242, 1161, 1136, 1022, 993, 880, 797, 764 cm^{-1};

HRMS (ESI) m/z: $[M+NH_4]^+$ Calcd for $C_{28}H_{26}Cl_2N_3O_6^+$: 570.1193; Found: 570.1186.

(1*E*,3*E*)-1,4-Bis(3,4-dimethoxyphenyl)buta-1,3-diene (6a)

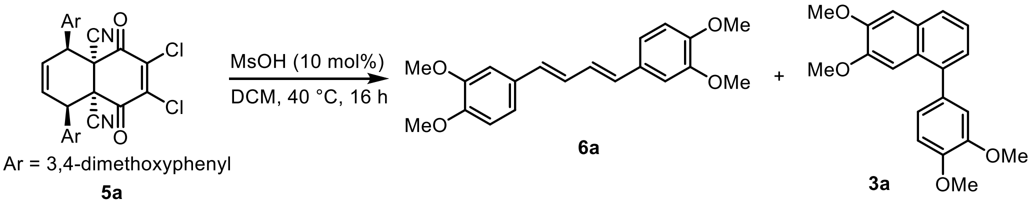

To a solution of Diels-Alder adduct **5a** (0.2 mmol, 111 mg) in DCM (2 mL) was added the solution of methanesulfonic acid in DCM (0.02 mmol, 0.5 M, 40 μL) and the reaction mixture was stirred at 40 °C for 16 h (oil bath). After that, the volatiles were removed under reduced pressure. Column chromatography of the residue on silica gel using 8/1 hexanes/EtOAc as the eluent gave 5 mg (8%) of **3a** as a colourless solid. Further elution (6/1 hexanes/EtOAc) gave 17 mg (26%) of diene **6a** as a colourless solid.

TLC R_f = 0.46 (1/1 hexanes/EtOAc);

M.p. 201 °C, dec. (from hexanes/EtOAc);

^{1}H NMR (400 MHz, $CDCl_3$): δ = 7.04 – 6.92 (m, 4H), 6.88 – 6.76 (m, 4H), 6.64 – 6.54 (m, 2H), 3.93 (s, 6H), 3.89 (s, 6H);

^{13}C{^{1}H} NMR (101 MHz, $CDCl_3$): δ = 149.3 (2C), 149.0 (2C), 131.9 (2C), 130.8 (2C), 127.8 (2C), 119.8 (2C), 111.4 (2C), 108.7 (2C), 56.1 (2C), 56.0 (2C).

The recorded spectroscopic values agree with the previously reported data.[34]

(*E*)-4,4'-(But-1-ene-1,4-diyl)bis(1,2-dimethoxybenzene), ficusnotin D (7a)

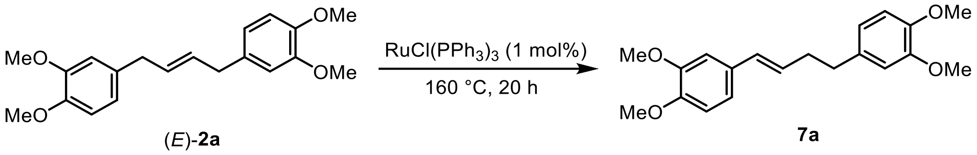

Following the literature method,[35] a mixture of (*E*)-**2a** (0.5 mmol, 164 mg) and $RuCl_2(PPh_3)_3$ (0.005 mmol, 4.8 mg) was heated at 160 °C under argon atmosphere without solvent in a sealed 10 mL vial placed in a metal block for 20 h. Column chromatography of the formed solid on silica gel using 6/1 hexanes/EtOAc as the eluent gave 115 mg (70%) of the title compound as a colourless solid.

M.p. 112 °C (from hexanes/EtOAc);

TLC R_f = 0.27 (3/1 hexanes/EtOAc);

^{1}H NMR (400 MHz, $CDCl_3$): δ = 6.92 – 6.84 (m, 2H), 6.83 – 6.74 (m, 4H), 6.35 (d, *J* = 15.8 Hz, 1H), 6.13 (dt, *J* = 15.8, 6.8 Hz, 1H), 3.91 – 3.84 (m, 12H), 2.80 – 2.67 (m, 2H), 2.57 – 2.43 (m, 2H);

^{13}C{^{1}H} NMR (101 MHz, $CDCl_3$): δ = 149.1, 148.9, 148.4, 147.3, 134.6, 131.0, 130.1, 128.2, 120.3, 119.0, 112.0, 111.33, 111.29, 108.7, 56.0 (2C), 55.9 (2C), 35.7, 35.2.

The recorded spectroscopic values agree with the previously reported data.[36]

[34] Ho, S. S. H.; Go, M. L. *Bioorg. Med. Chem. Lett.* **2013**, *23*, 6127–6133.
[35] Blum, J.; Becker, Y. *J. Chem. Soc. Perkin Trans. 2* **1972**, 982–989.
[36] Latayada, F. S.; Uy, M. M.; Akihara, Y.; Ohta, E.; Nehira, T.; Ômura, H.; Ohta, S. *Phytochemistry* **2017**, *141*, 98–104.

Formation of lignan 3a from Diels-Alder adduct 5a or from isomeric alkene 7a

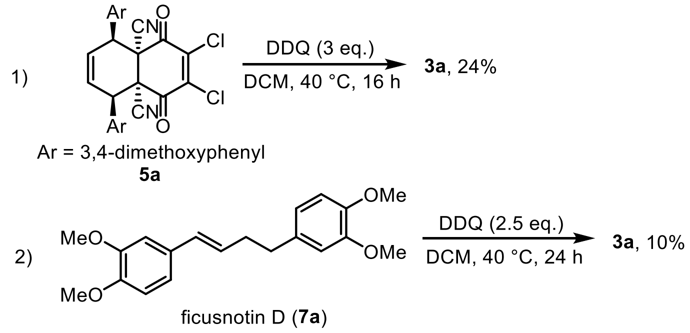

Example 1. To a solution of compound **5a** (0.05 mmol, 28 mg) in DCM (0.5 mL) was added DDQ (0.15 mmol, 34 mg) and the reaction mixture was stirred at 40 °C for 16 h (oil bath). The reaction mixture was filtered, washed with DCM, and concentrated under reduced pressure. Analysis of the residue by ^{1}H NMR using dibromomethane as the internal standard showed that compound **3a** was formed in 24% yield.

Example 2. To a solution of compound **7a** (0.2 mmol, 66 mg) in DCM (2 mL) was added DDQ (0.5 mmol, 114 mg) and the reaction mixture was stirred at 40 °C for 24 h (oil bath). The reaction mixture was filtered, washed with DCM, and concentrated under reduced pressure. Analysis of the residue by ^{1}H NMR using dibromomethane as the internal standard showed that compound **3a** was formed in 10% yield.

2.6. Functionalization of lignan 3a

1-Bromo-4-(3,4-dimethoxyphenyl)-6,7-dimethoxynaphthalene (8)

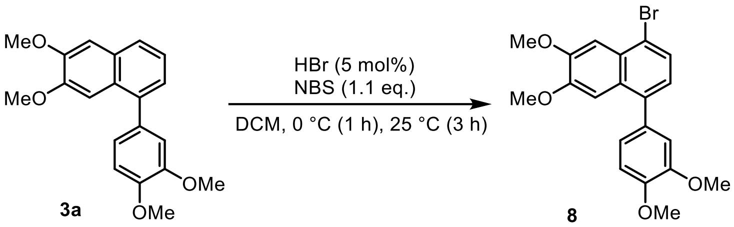

To a stirred solution of compound **3a** (0.25 mmol, 81 mg) in DCM (2.5 mL) was added *N*-bromosuccinimide (0.275 mmol, 49 mg) at 0 °C in one portion. Next, aqueous HBr (48% wt., 12.5 μmol, 2.1 mg) was introduced and the stirring was continued for 1 h at 0 °C and for 3 h at room temperature (25 °C). After that, the reaction mixture was poured into water (10 mL) and extracted with DCM (3×5 mL). The combined organic extracts were dried over anhydrous Na_2SO_4 and concentrated under reduced pressure. Column chromatography of the residue on silica gel using 8/1 hexanes/EtOAc as the eluent gave 71 mg (70%) of the title compound as a colourless solid.

TLC R_f = 0.50 (1/1 hexanes/EtOAc);

M.p. 173 °C (from hexanes/EtOAc);

^{1}H NMR (400 MHz, $CDCl_3$): δ = 7.64 (d, *J* = 7.8 Hz, 1H), 7.57 (s, 1H), 7.25 (s, 1H), 7.10 (d, *J* = 7.8 Hz, 1H), 7.02 – 6.96 (m, 3H), 4.06 (s, 3H), 3.96 (s, 3H), 3.88 (s, 3H), 3.82 (s, 3H);

^{13}C{^{1}H} NMR (101 MHz, $CDCl_3$): δ = 150.4, 149.9, 148.8, 148.5, 138.6, 133.1, 128.6, 128.2, 127.8, 125.7, 122.0, 120.3, 113.1, 111.2, 106.4, 105.4, 56.04, 56.02, 56.00, 55.9;

IR (KBr): ṽ = 3093, 3070, 3003, 2951, 2931, 2904, 2829, 1622, 1604, 1579, 1506, 1477, 1462, 1446, 1429, 1408, 1334, 1246, 1227, 1209, 1169, 1138, 1120, 1028, 918, 850, 833, 808, 783, 768, 754 cm^{-1};

HRMS (ESI) m/z: $[M+H]^+$ Calcd for $C_{20}H_{20}BrO_4^+$: 403.0539; Found: 403.0539.

1-Bromo-4-(2-bromo-4,5-dimethoxyphenyl)-6,7-dimethoxynaphthalene (9)

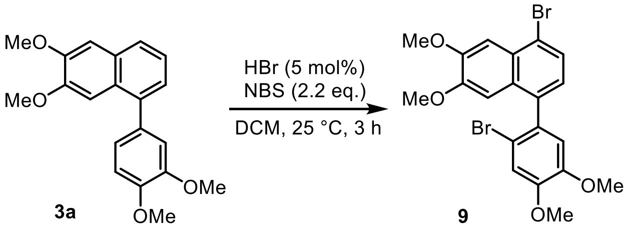

To a stirred solution of compound **3a** (0.25 mmol, 81 mg) in DCM (2.5 mL) was added *N*-bromosuccinimide (0.55 mmol, 98 mg) at room temperature (25 °C) in one portion. Next, aqueous HBr (48% wt., 12.5 μmol, 2.1 mg) was introduced and the stirring was continued for 3 h. After that, the reaction mixture was poured into water (10 mL) and extracted with DCM (3×5 mL). The combined organic extracts were dried over anhydrous Na_2SO_4 and concentrated under reduced pressure. Column

chromatography of the residue on silica gel using 10/1 hexanes/EtOAc as the eluent gave 110 mg (91%) of the title compound as a colourless solid.

TLC R_f = 0.55 (1/1 hexanes/EtOAc);

M.p. 157 °C (from hexanes/EtOAc);

^{1}H NMR (400 MHz, $CDCl_3$): δ = 7.68 (d, *J* = 7.7 Hz, 1H), 7.58 (s, 1H), 7.19 (s, 1H), 7.07 (d, *J* = 7.7 Hz, 1H), 6.84 (s, 1H), 6.79 (s, 1H), 4.07 (s, 3H), 3.96 (s, 3H), 3.84 (s, 3H), 3.80 (s, 3H);

^{13}C{^{1}H} NMR (101 MHz, $CDCl_3$): δ = 150.6, 150.2, 149.2, 148.3, 137.6, 132.9, 128.7, 128.0, 127.7, 126.2, 121.1, 115.5, 114.3, 114.1, 106.4, 105.3, 56.3, 56.2, 56.1, 56.0;

IR (KBr): ṽ = 3078, 3001, 2978, 2951, 2931, 2900, 2835, 1618, 1599, 1568, 1504, 1483, 1462, 1435, 1377, 1259, 1240, 1205, 1167, 1126, 1026, 1011, 920, 870, 837, 797, 775 cm^{-1};

HRMS (ESI) m/z: $[M+Na]^+$ Calcd for $C_{20}H_{18}Br_2O_4Na^+$: 502.9464; Found: 502.9459.

1-(4,5-Dimethoxy-2-nitrophenyl)-6,7-dimethoxy-4-nitronaphthalene (10)

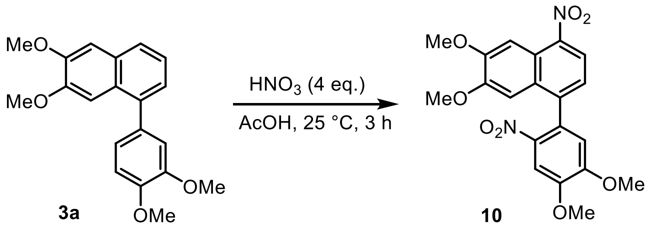

To a solution of compound **3a** (0.25 mmol, 81 mg) in glacial acetic acid (2.5 mL) was added aqueous nitric acid (65% wt., 1 mmol, 70 µl) at room temperature (25 °C). After stirring for 3 h, the reaction mixture was poured into water (25 mL) and extracted with EtOAc (3×10 mL). The combined organic extracts were dried over anhydrous Na_2SO_4 and concentrated under reduced pressure. Column chromatography of the residue on silica gel using 3/1 hexanes/EtOAc as the eluent gave 60 mg (58%) of the title compound as a yellow solid.

TLC R_f = 0.48 (1/1 hexanes/EtOAc);

M.p. 204 °C (from hexanes/EtOAc);

^{1}H NMR (400 MHz, $CDCl_3$): δ = 8.18 (d, *J* = 7.9 Hz, 1H), 8.09 (s, 1H), 7.78 (s, 1H), 7.25 (d, *J* = 7.9 Hz, 1H), 6.79 (s, 1H), 6.72 (s, 1H), 4.08 – 4.04 (m, 6H), 3.94 (s, 3H), 3.76 (s, 3H);

^{13}C{^{1}H} NMR (101 MHz, $CDCl_3$): δ = 153.2, 152.4, 150.7, 149.1, 144.9, 141.9, 141.3, 129.1, 128.7, 122.9, 122.4, 122.0, 113.5, 107.9, 104.2, 102.8, 56.8, 56.7, 56.3, 55.9;

IR (KBr): ṽ = 3091, 3070, 3024, 3001, 2974, 2933, 2843, 1620, 1570, 1514, 1485, 1458, 1437, 1331, 1315, 1265, 1250, 1215, 1182, 1059, 1012, 833, 793, 737 cm^{-1};

HRMS (ESI) m/z: $[M+H]^+$ Calcd for $C_{20}H_{19}N_2O_8^+$: 415.1136; Found: 415.1134.

Diethyl (4-(3,4-dimethoxyphenyl)-6,7-dimethoxynaphthalen-1-yl)phosphonate (11)

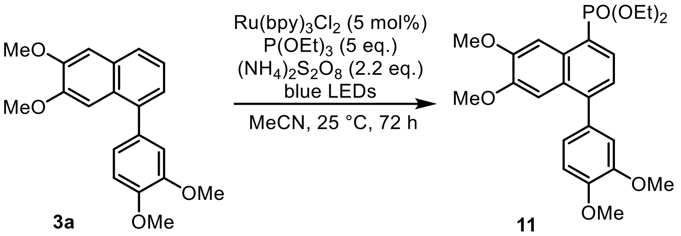

Using the literature method,[37] a suspension of compound **3a** (0.25 mmol, 81 mg), tris(bipyridine)ruthenium(II) chloride (12.5 µmol, 8 mg), triethyl phosphite (1.25 mmol, 208 mg, 0.21 mL) and ammonium persulfate (0.55 mmol, 125 mg) in acetonitrile (2.5 mL) was stirred under irradiation with blue LEDs (470 nm, 14.4 W/m, 1 m)[38] at room temperature (25 °C) for 72 h. After that, the reaction mixture was filtered, the solid was discarded, and the solution was concentrated under reduced pressure. Column chromatography of the residue on silica gel using Et_2O as the eluent gave 51 mg (44%) of the title compound as a colourless viscous oil.

TLC R_f = 0.28 (EtOAc);

M.p. 133 °C (from hexanes/EtOAc);

^{1}H{^{31}P} NMR (400 MHz, $CDCl_3$): δ = 8.08 (d, *J* = 7.4 Hz, 1H), 8.02 (s, 1H), 7.32 (d, *J* = 7.4 Hz, 1H), 7.30 (s, 1H), 7.06 – 6.98 (m, 3H), 4.26 – 4.18 (m, 2H), 4.13 – 4.06 (m, 2H), 4.04 (s, 3H), 3.96 (s, 3H), 3.89 (s, 3H), 3.81 (s, 3H), 1.35 – 1.30 (m, 6H);

^{13}C{^{1}H} NMR (101 MHz, $CDCl_3$): δ = 150.1, 149.4, 148.9, 148.8, 144.0 (d, *J* = 3.5 Hz), 133.2, 132.3 (d, *J* = 8.8 Hz), 129.8 (d, *J* = 11.2 Hz), 128.1 (d, *J* = 13.4 Hz), 124.3 (d, *J* = 16.4 Hz), 122.0, 121.6 (d, *J* = 183.7 Hz), 113.0, 111.3, 106.1 (d, *J* = 4.4 Hz), 105.6, 62.2 (d, *J* = 5.2 Hz, 2C), 56.09, 56.07, 56.0, 55.8, 16.5 (d, *J* = 6.6 Hz, 2C);

^{31}P{^{1}H} NMR (162 MHz, $CDCl_3$): δ = 20.0;

IR (KBr): ṽ = 3020, 2968, 2953, 2935, 2902, 2835, 1620, 1605, 1583, 1510, 1481,1433, 1363, 1246, 1169, 1138, 1047, 1016, 962, 922, 862, 841, 801, 765, 676 cm^{-1};

HRMS (ESI) m/z: $[M+H]^+$ Calcd for $C_{24}H_{30}O_7P^+$: 461.1724; Found: 461.1723.

[37] Shaikh, R. S.; Ghosh, I.; König, B. *Chem. Eur. J.* **2017**, *23*, 12120−12124.

[38] For the detailed description of the experimental setup used, see: Fadeev, A. A.; Kotora, M. *Org. Biomol. Chem.* **2023**, *21*, 6174–6179.

3-(*tert*-Butyl)-1-(3,4-dimethoxyphenyl)-6,7-dimethoxynaphthalene (12)

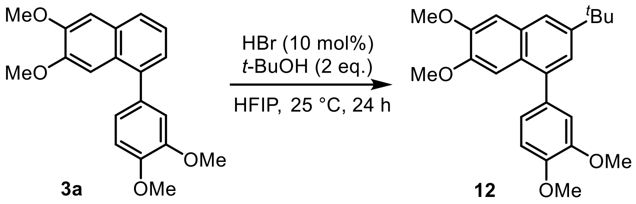

To a stirred solution of compound **3a** (0.25 mmol, 81 mg) in hexafluoroisopropanol (2.5 mL) was added *tert*-butanol (0.5 mmol, 37 mg, 48 µl) followed by aqueous HBr (48% wt., 25 µmol, 4.2 mg) at room temperature (25 °C). The stirring was continued for 24 h, after which the resulting solution was concentrated under reduced pressure. Column chromatography of the residue on silica gel using 10/1 hexanes/EtOAc as the eluent gave 85 mg (89%) of the title compound as a colourless oil.

TLC R_f = 0.52 (1/1 hexanes/EtOAc);

^{1}H NMR (400 MHz, $CDCl_3$): δ = 7.66 (d, *J* = 2.0 Hz, 1H), 7.37 (d, *J* = 2.0 Hz, 1H), 7.21 (s, 1H), 7.19 (s, 1H), 7.10 – 7.06 (m, 2H), 7.02 (d, *J* = 8.0 Hz, 1H), 4.02 (s, 3H), 3.98 (s, 3H), 3.91 (s, 3H), 3.82 (s, 3H), 1.43 (s, 9H);

^{13}C{^{1}H} NMR (101 MHz, $CDCl_3$): δ = 149.5, 149.2, 148.7, 148.3, 146.7, 138.4, 134.5, 129.8, 125.5, 124.3, 122.1, 121.4, 113.3, 111.3, 107.0, 104.7, 56.10, 56.09, 56.0, 55.9, 34.8, 31.5 (3C);

IR (KBr): ṽ = 3055, 2999, 2953, 2935, 2904, 2868, 2833, 1626, 1603, 1579, 1506, 1479, 1462, 1431, 1242, 1163, 1138, 1026, 1014, 874, 851, 733 cm^{-1};

HRMS (ESI) m/z: $[M+H]^+$ Calcd for $C_{24}H_{29}O_4^+$: 381.2060; Found: 381.2056.

1-(3,4-Dimethoxyphenyl)-6,7-dimethoxy-1,2,3,4-tetrahydronaphthalene (13)

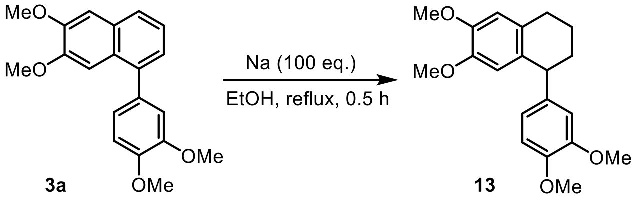

Caution! Sodium metal reacts violently with ethanol and releases flammable hydrogen gas!

Using the literature method,[39] to a refluxing solution of compound **3a** (0.25 mmol, 81 mg) in absolute ethanol (5 mL) under argon atmosphere was carefully added metallic sodium (25 mmol, 575 mg) in small pieces over the course of 1.5 h. At the end of addition, a heavy inorganic precipitate was formed, which was dissolved upon dilution with ethanol (2 mL). The reaction mixture was refluxed for 0.5 h (oil bath) after the whole amount of sodium was added. Next, the solution was cooled to room temperature (25 °C) and poured into water (50 mL), diluted with brine (50 mL) and the product was extracted with EtOAc (3×20 mL). The combined organic extracts were dried over anhydrous Na_2SO_4

[39] Carreño, M. C.; González-López, M.; Latorre, A.; Urbano, A. *Synlett* **2005**, 1601−1605.

and concentrated under reduced pressure. Column chromatography of the residue on silica gel using 10/1 hexanes/EtOAc as the eluent gave 60 mg (73%) of the title compound as a white solid.

TLC R_f = 0.53 (1/1 hexanes/EtOAc);

M.p. 73 °C (from hexanes/EtOAc);

^{1}H NMR (400 MHz, $CDCl_3$): δ = 6.78 (d, *J* = 7.9 Hz, 1H), 6.65 – 6.59 (m, 3H), 6.36 (s, 1H), 3.99 (t, *J* = 6.5 Hz, 1H), 3.87 – 3.85 (m, 6H), 3.80 (s, 3H), 3.65 (s, 3H), 2.87 – 2.71 (m, 2H), 2.16 – 2.06 (m, 1H), 1.90 – 1.77 (m, 2H), 1.76 – 1.66 (m, 1H);

^{13}C{^{1}H} NMR (101 MHz, $CDCl_3$): δ = 148.8, 147.4, 147.3, 147.1, 140.2, 131.2, 129.7, 121.0, 112.9, 111.9, 111.4, 110.9, 55.94, 55.93, 55.92, 55.87, 44.9, 33.6, 29.5, 21.0;

IR (KBr): ṽ = 3003, 2931, 2918, 2843, 1606, 1593, 1512, 1466, 1446, 1356, 1255, 1244, 1213, 1142, 1113, 1016, 852, 806, 759, 743 cm^{-1};

HRMS (ESI) m/z: $[M+Na]^+$ Calcd for $C_{20}H_{24}O_4Na^+$: 351.1567; Found: 351.1565.

3. Cyclic voltammetry measurements

The three consecutive anodic scans of compounds (*E*)-**2a** and **3a** are shown in Figure S2. Both of the compounds exhibit an irreversible well-developed anodic peak at ~+1.0–1.1 V. The structural similarity of the compounds and the close values of the oxidation potentials suggest that the oxidation of the dimethoxyaryl moieties takes place in both cases. These findings agree with the oxidation of other dimethoxyarenes, such as 1,2-dimethoxybenzene[40] and eugenol,[41] which are known to undergo one-electron oxidation into the corresponding delocalized radical cations. These radical cations can react with the starting dimethoxyarenes forming Scholl-type oligomeric products. Alternatively, if the substrate bears an allylic substituent, as in the case of eugenol, the loss of hydrogen from the benzylic position provides quinoid products.

Additionally, an indistinctive peak was observed for (*E*)-**2a** at a potential of ~+1.2 V, and two indistinctive peaks were observed for **3a** at ~+1.35 V and ~+1.6 V. These peaks may be attributed to the subsequent oxidation of the products generated by oxidation (Fig. S2, peak at ~+1.0–1.1 V) of the initial compounds. The peak current decreased with subsequent scans for both of the studied compounds due to blocking of the BDD electrode surface by the formed oligomeric layer.

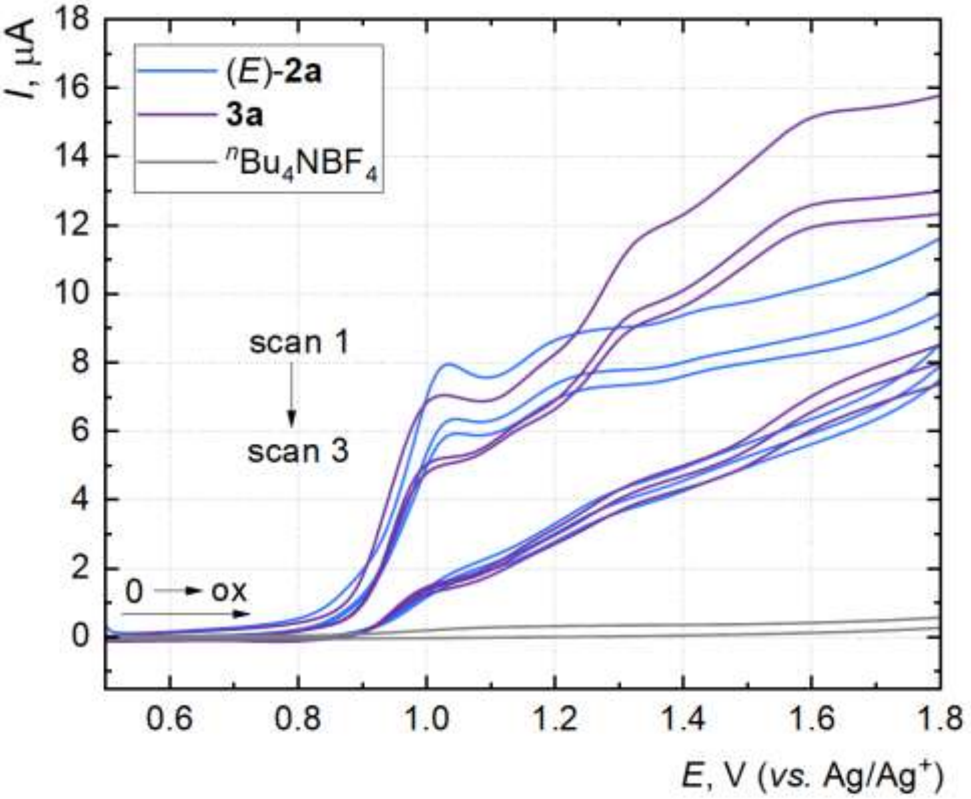

Figure S2. CV profiles of compounds (*E*)-**2a** and **3a** (3 anodic scans).

[40] Márquez, O. P.; Fontal, B.; Márquez, J.; Ortíz, R.; Castillo, R.; Choy, M.; Lárez, C. *J. Electrochem. Soc.* **1995**, *142*, 707–712.

[41] Yildiz, G.; Aydogmus, Z.; Cinar, M. E.; Senkal, F.; Ozturk, T. *Talanta* **2017**, *173*, 1–8.

4. Copies of ^{1}H and ^{13}C NMR spectra

1j, ^{1}H NMR (400 MHz, $CDCl_3$)

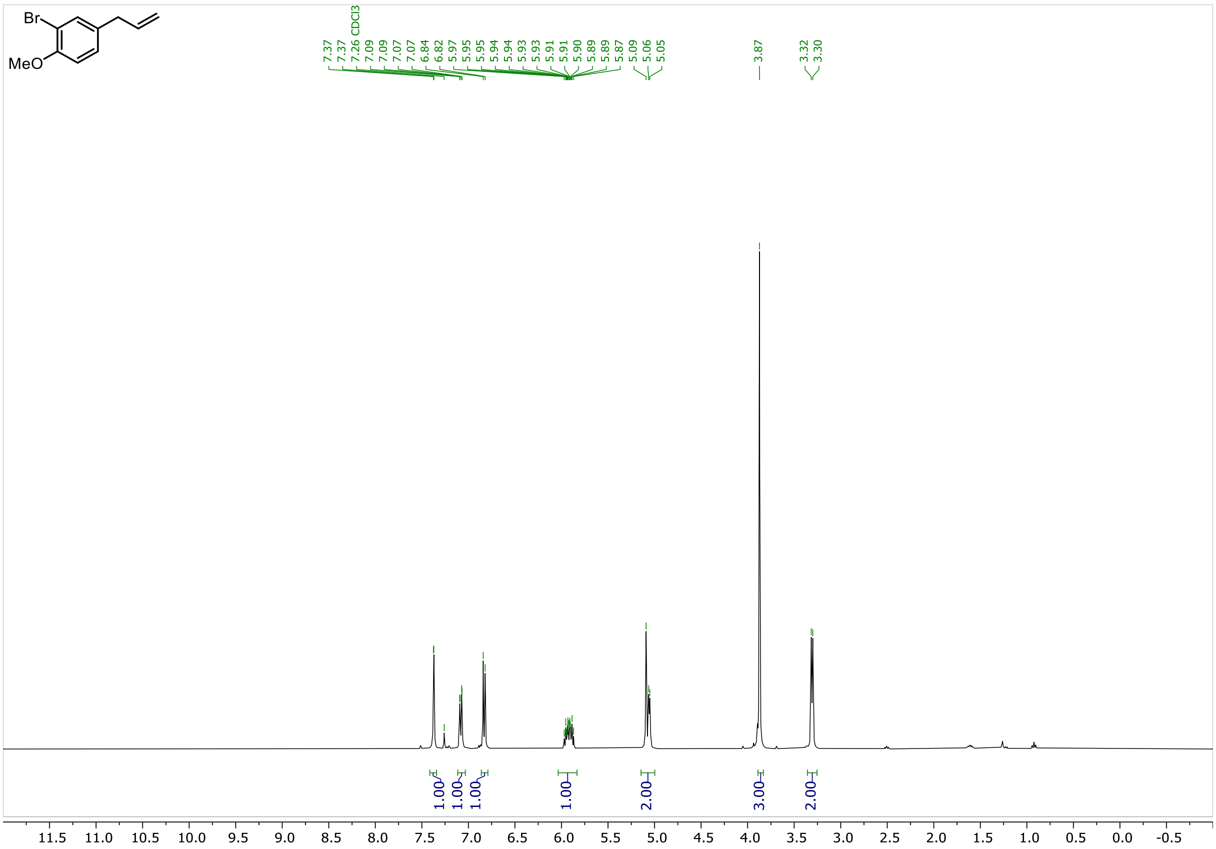

1j, ^{13}C{^{1}H} NMR (101 MHz, $CDCl_3$)

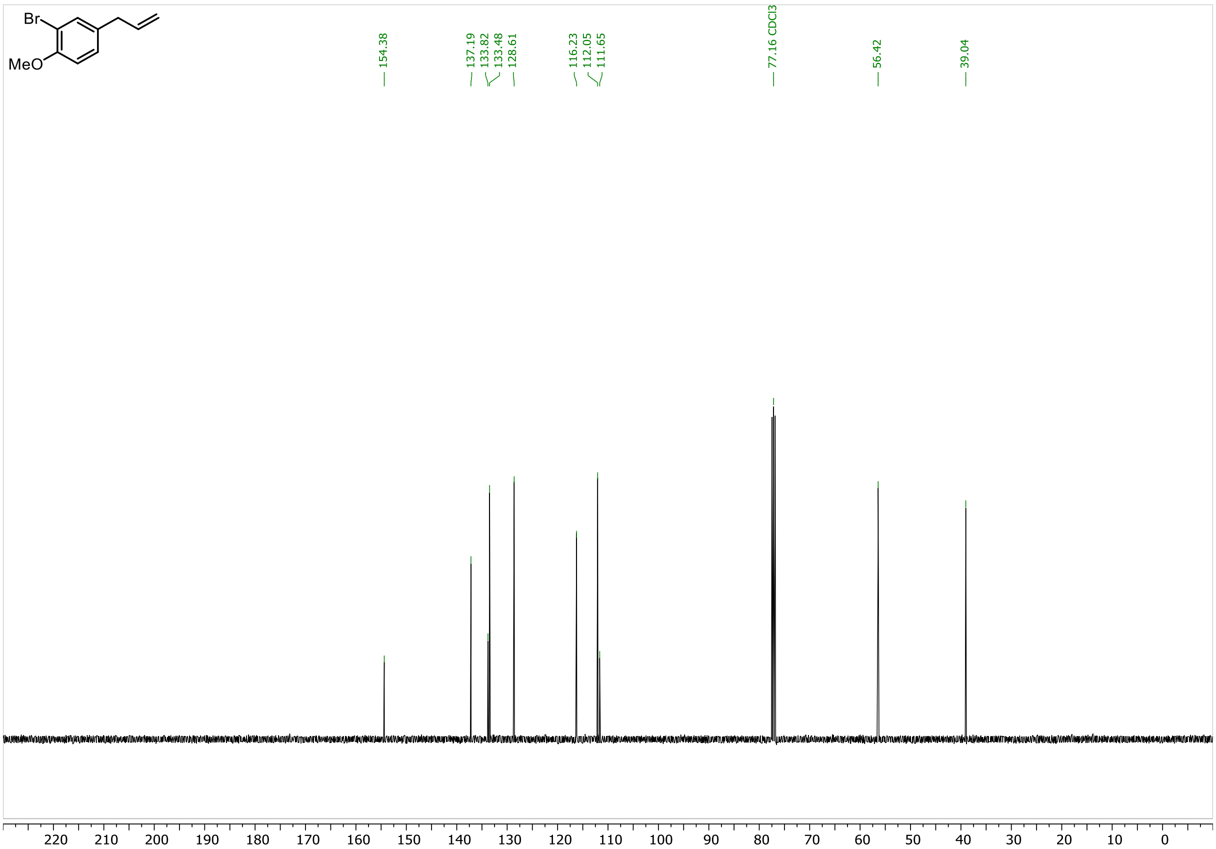

1j', ^{1}H NMR (400 MHz, $CDCl_3$)

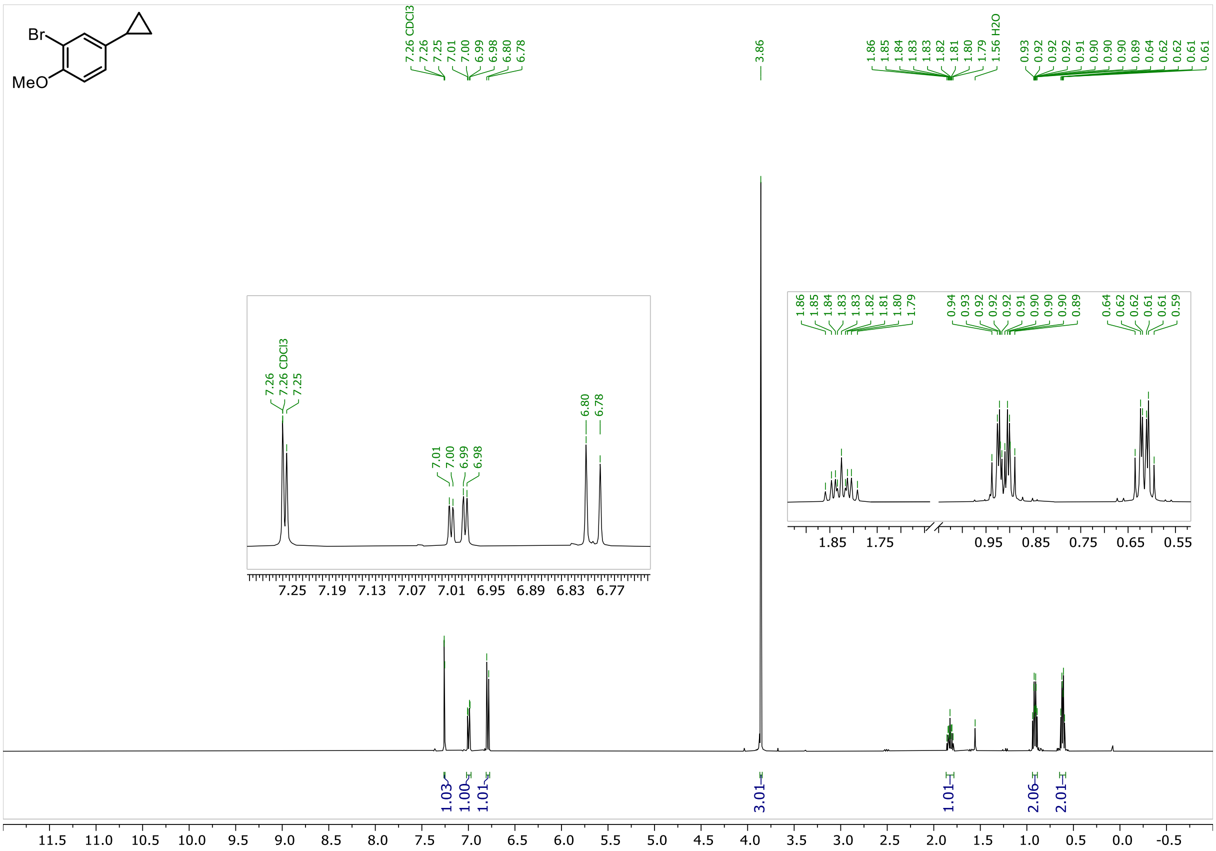

1j', $^{13}C\{^{1}H\}$ NMR (101 MHz, $CDCl_3$)

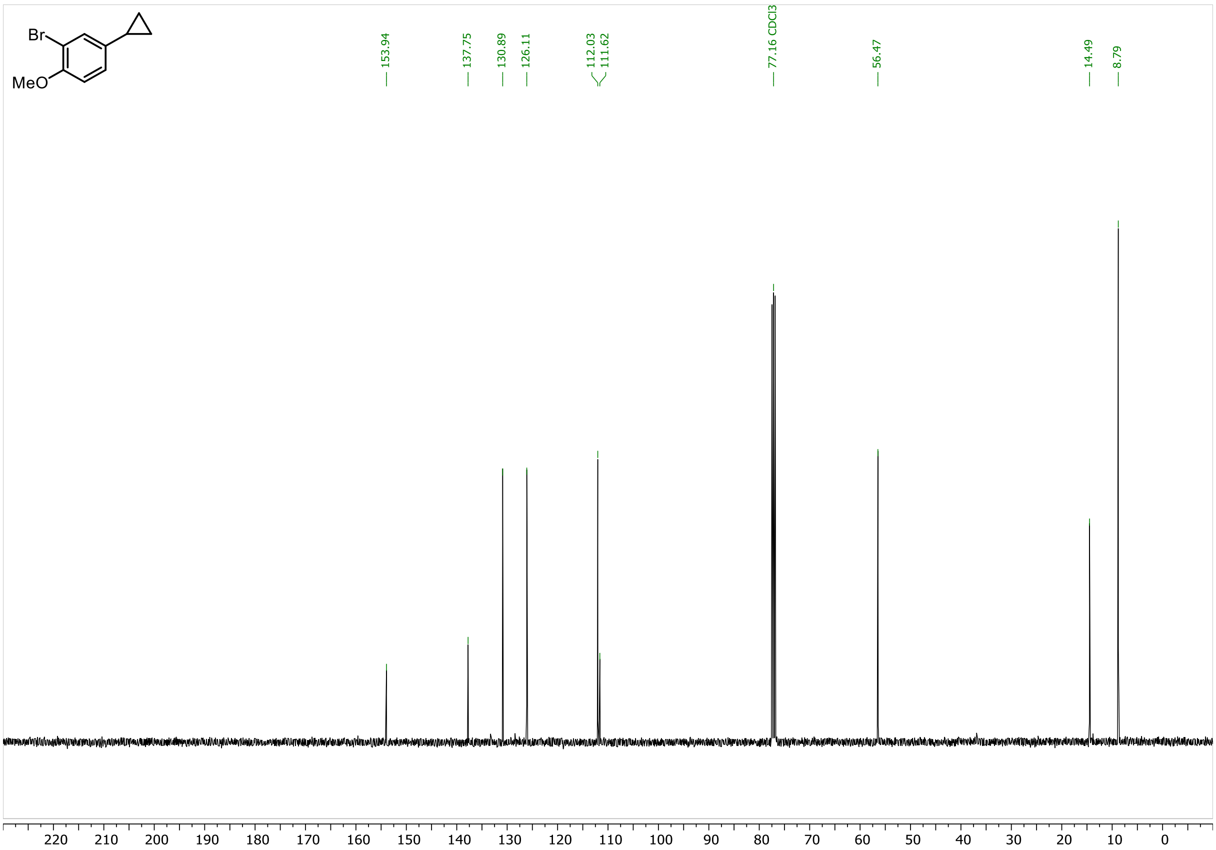

1u, ^{1}H NMR (400 MHz, CDCl$_3$)

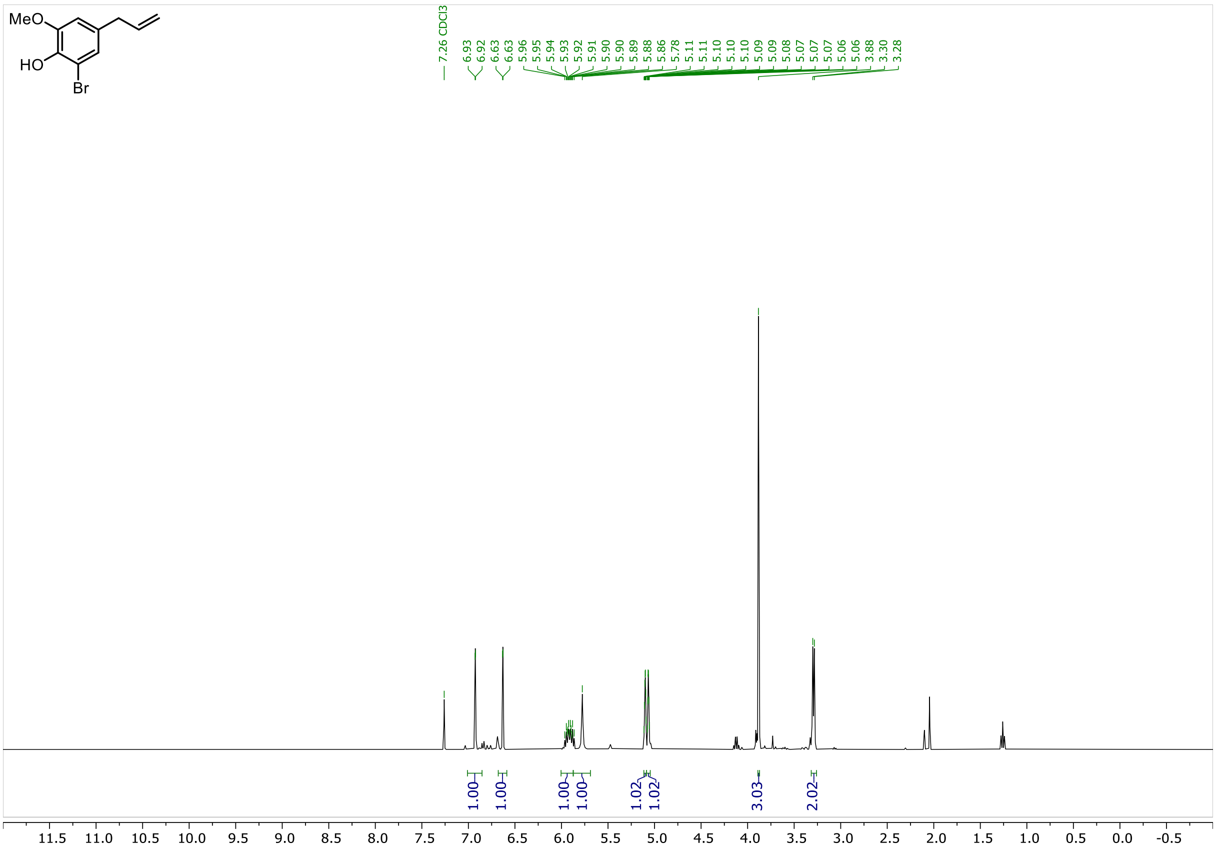

1u, ^{13}C{^{1}H} NMR (101 MHz, CDCl$_3$)

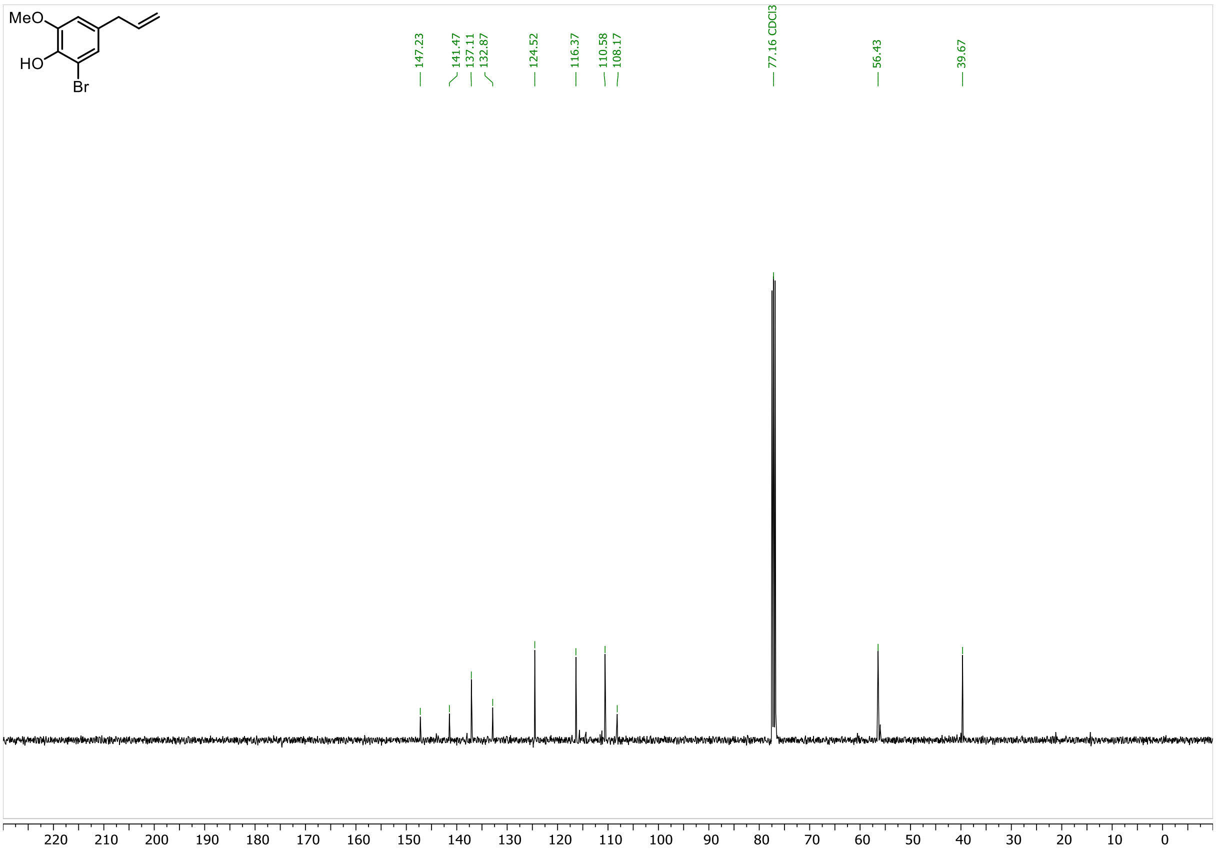

1k, ^{1}H NMR (400 MHz, CDCl$_3$)

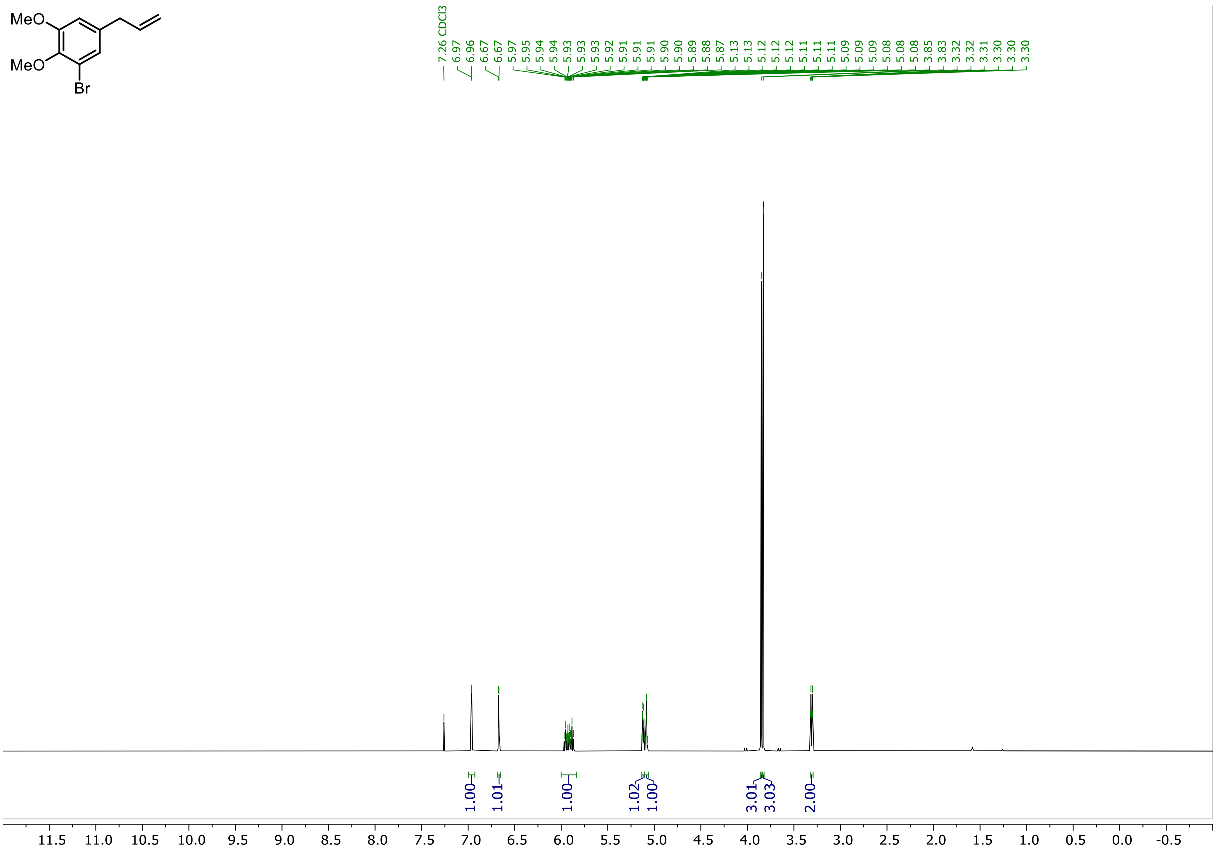

1k, ^{13}C{^{1}H} NMR (101 MHz, CDCl$_3$)

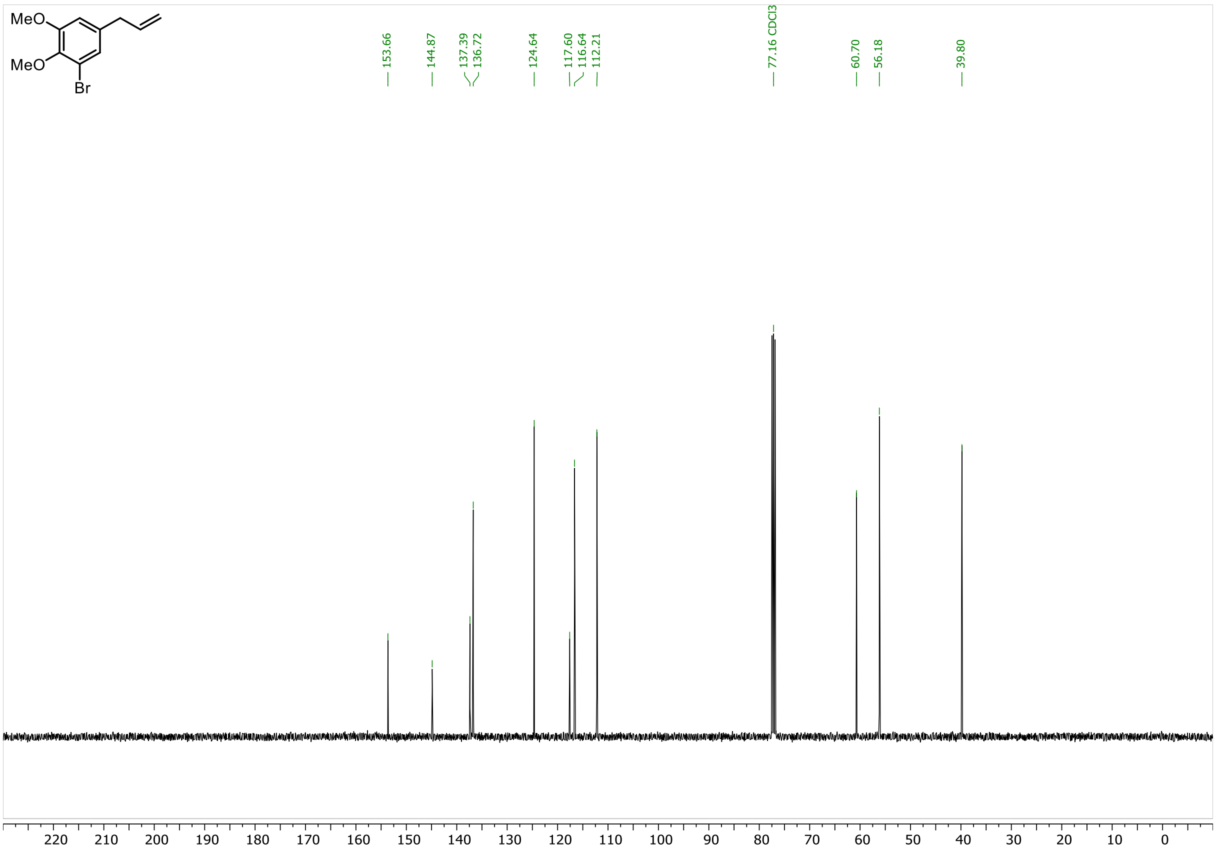

1l, ^{1}H NMR (400 MHz, CDCl$_3$)

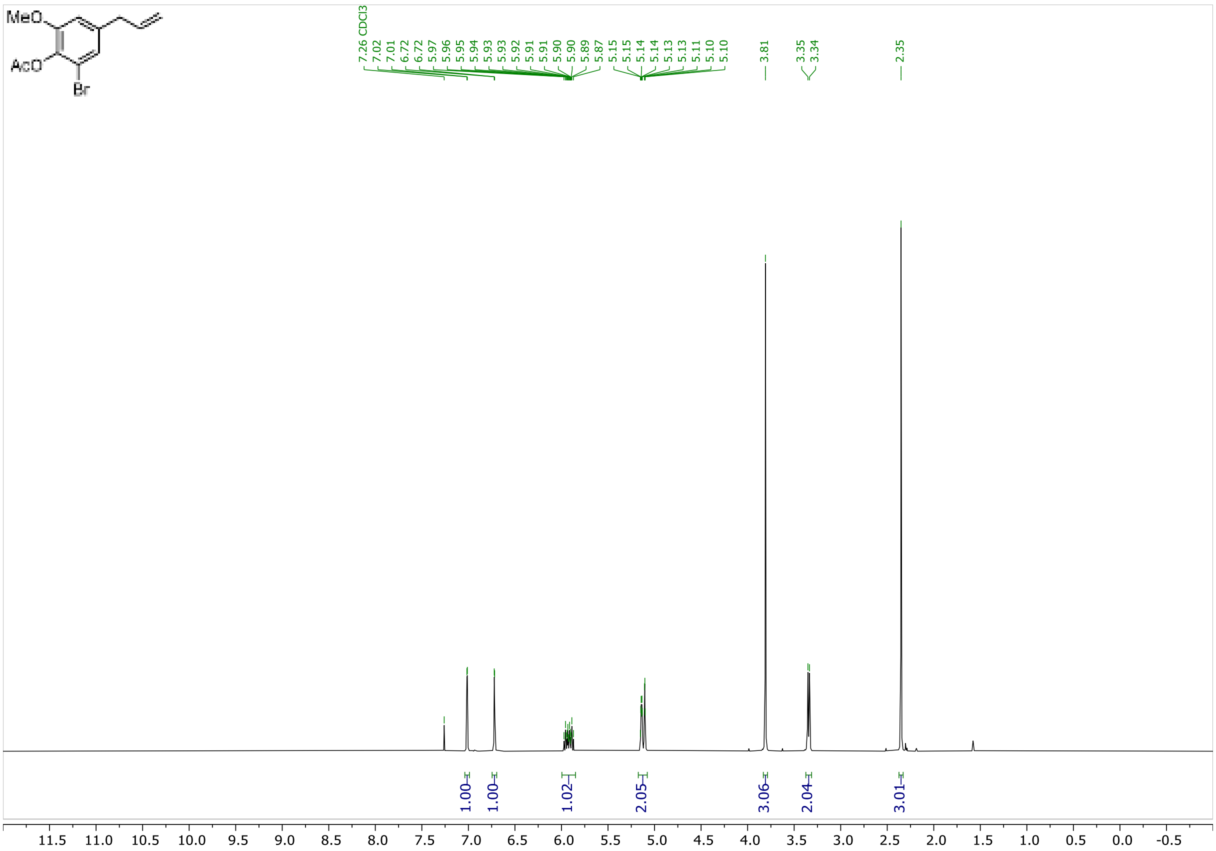

1l, ^{13}C{^{1}H} NMR (101 MHz, CDCl$_3$)

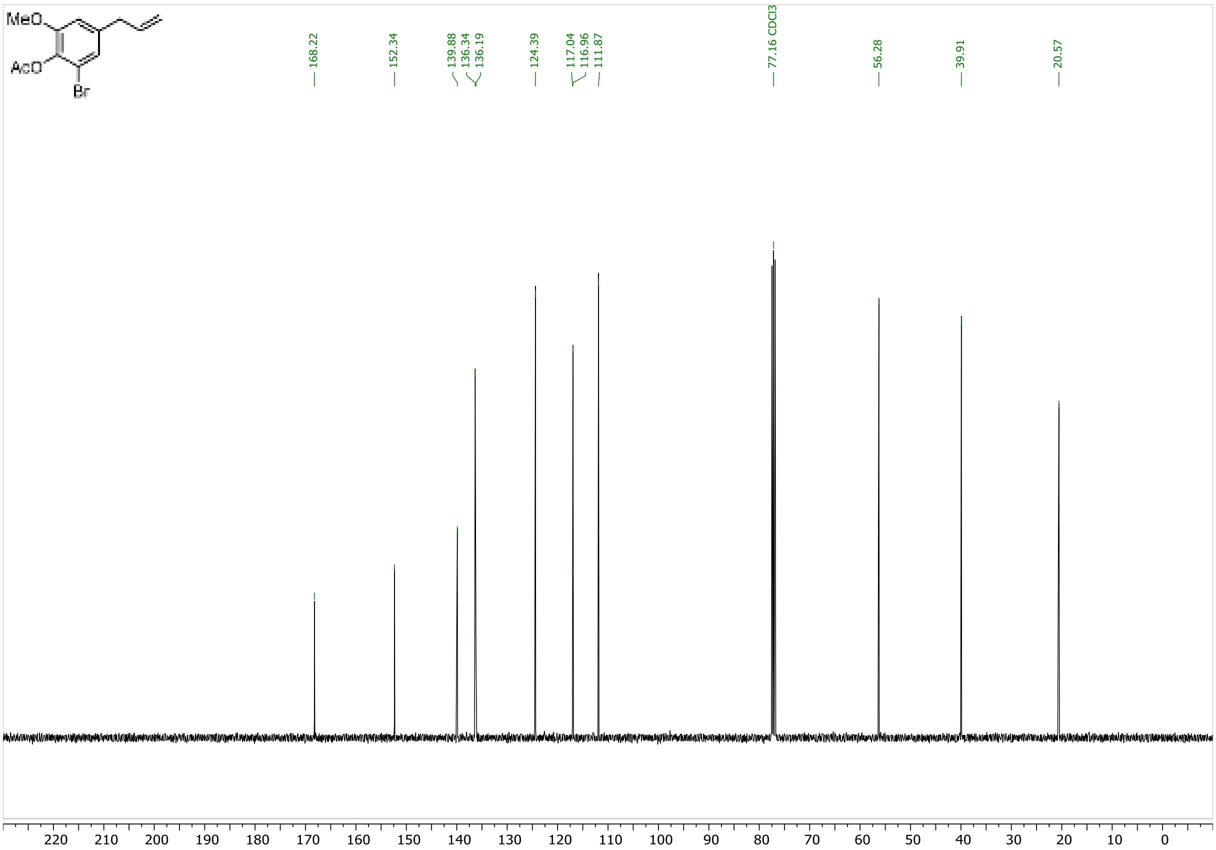

1m, ^{1}H NMR (400 MHz, $CDCl_3$)

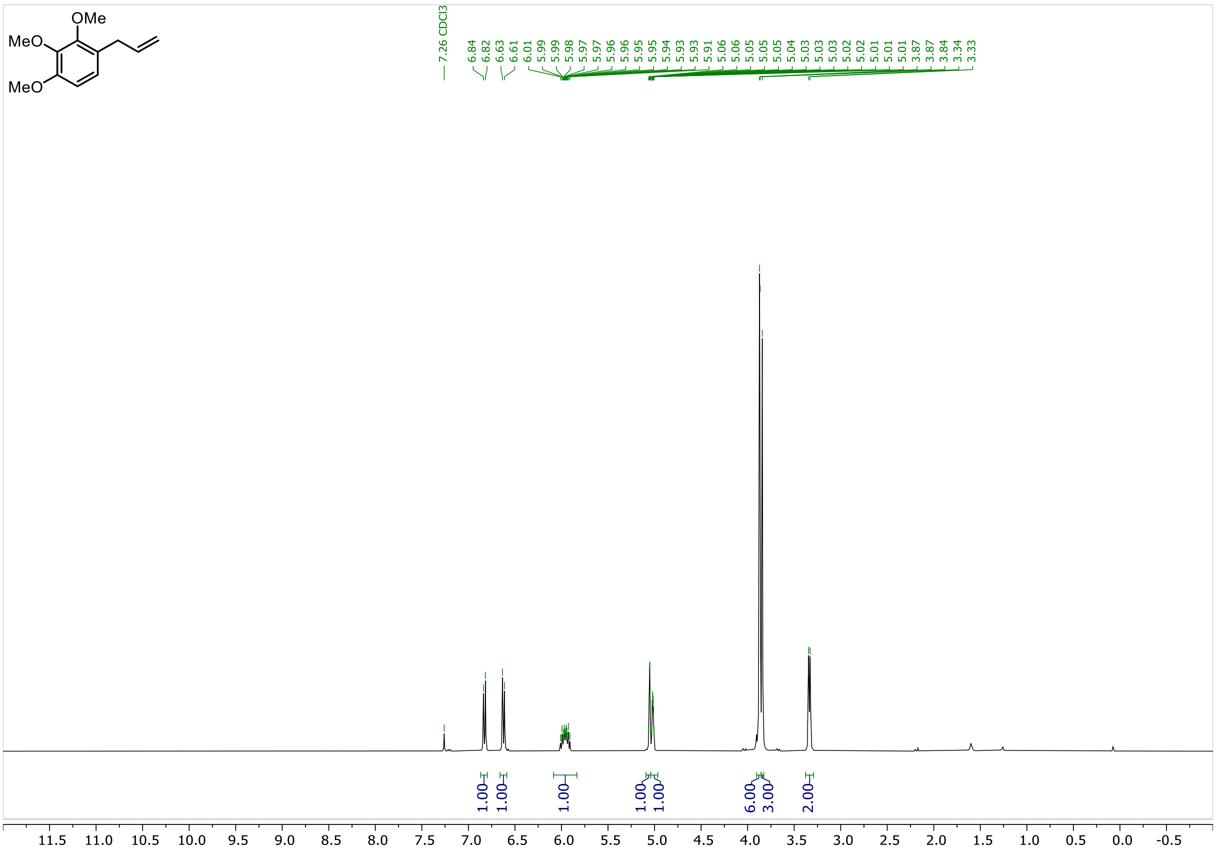

1m, $^{13}C\{^{1}H\}$ NMR (101 MHz, $CDCl_3$)

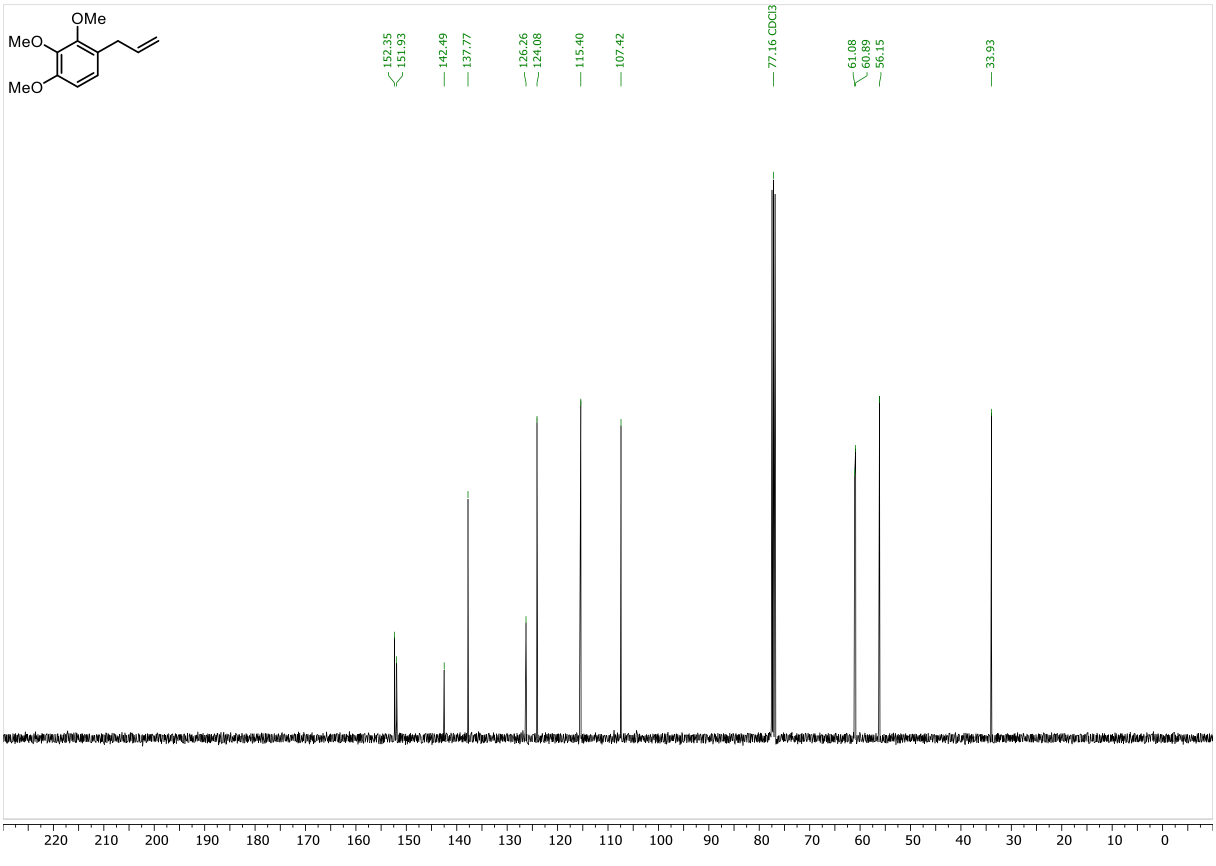

(*E*)-2a, ^{1}H NMR (400 MHz, $CDCl_3$)

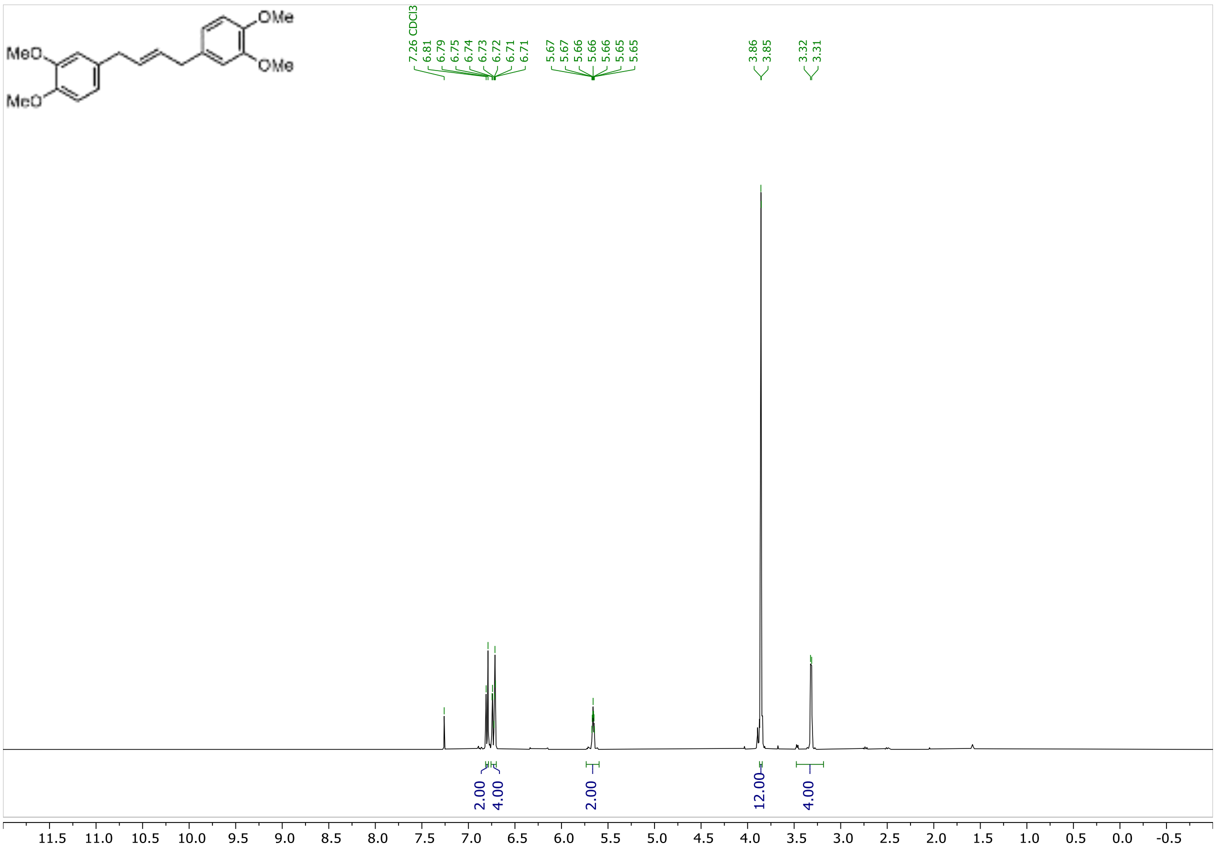

(*E*)-2a, ^{13}C{^{1}H} NMR (101 MHz, $CDCl_3$)

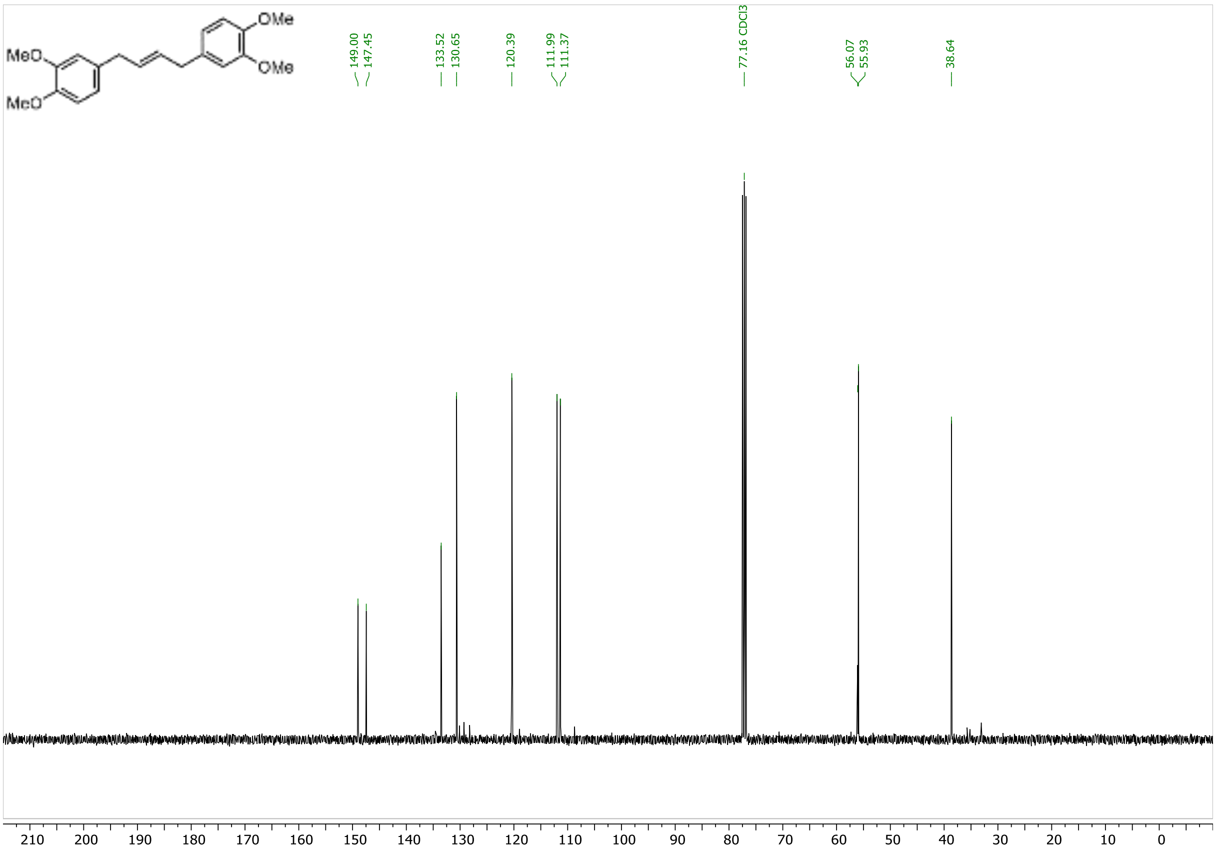

2s, ^{1}H NMR (400 MHz, $CDCl_3$)

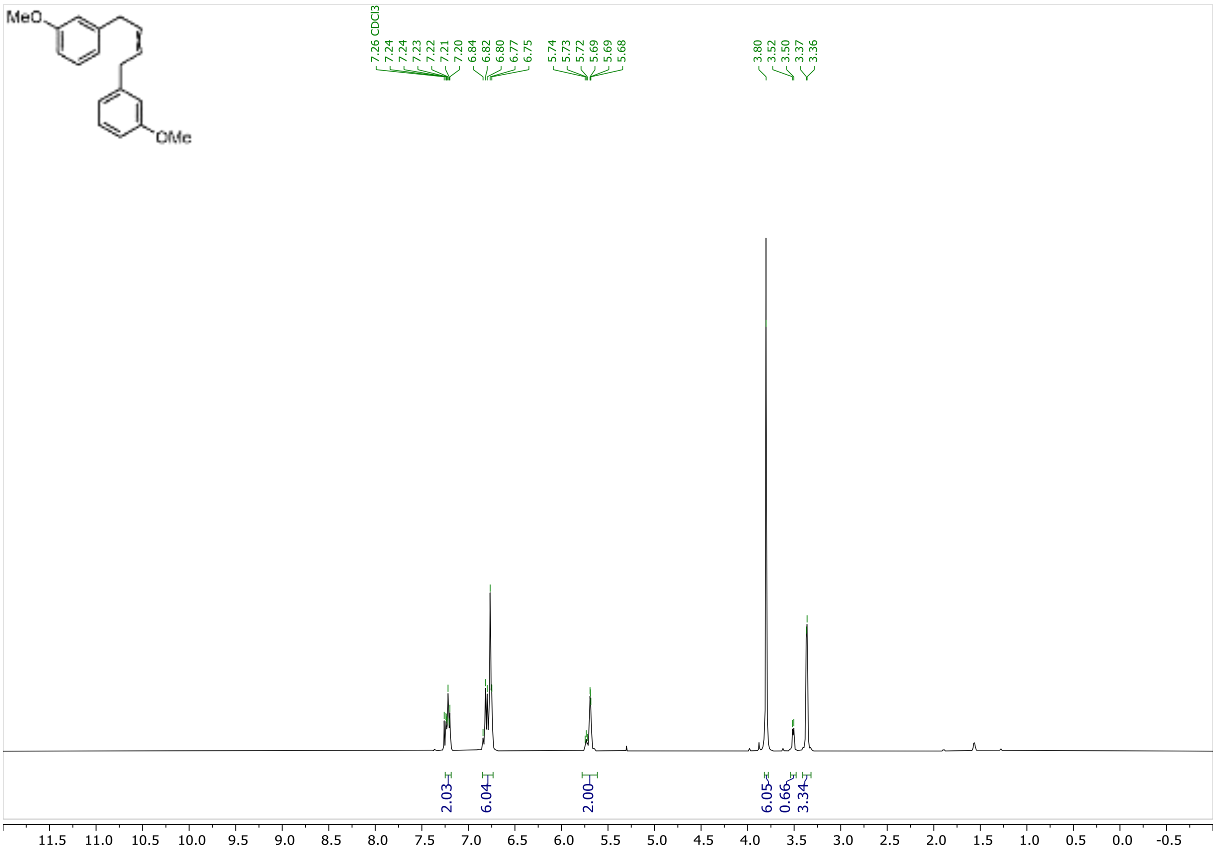

2s, ^{13}C{^{1}H} NMR (101 MHz, $CDCl_3$)

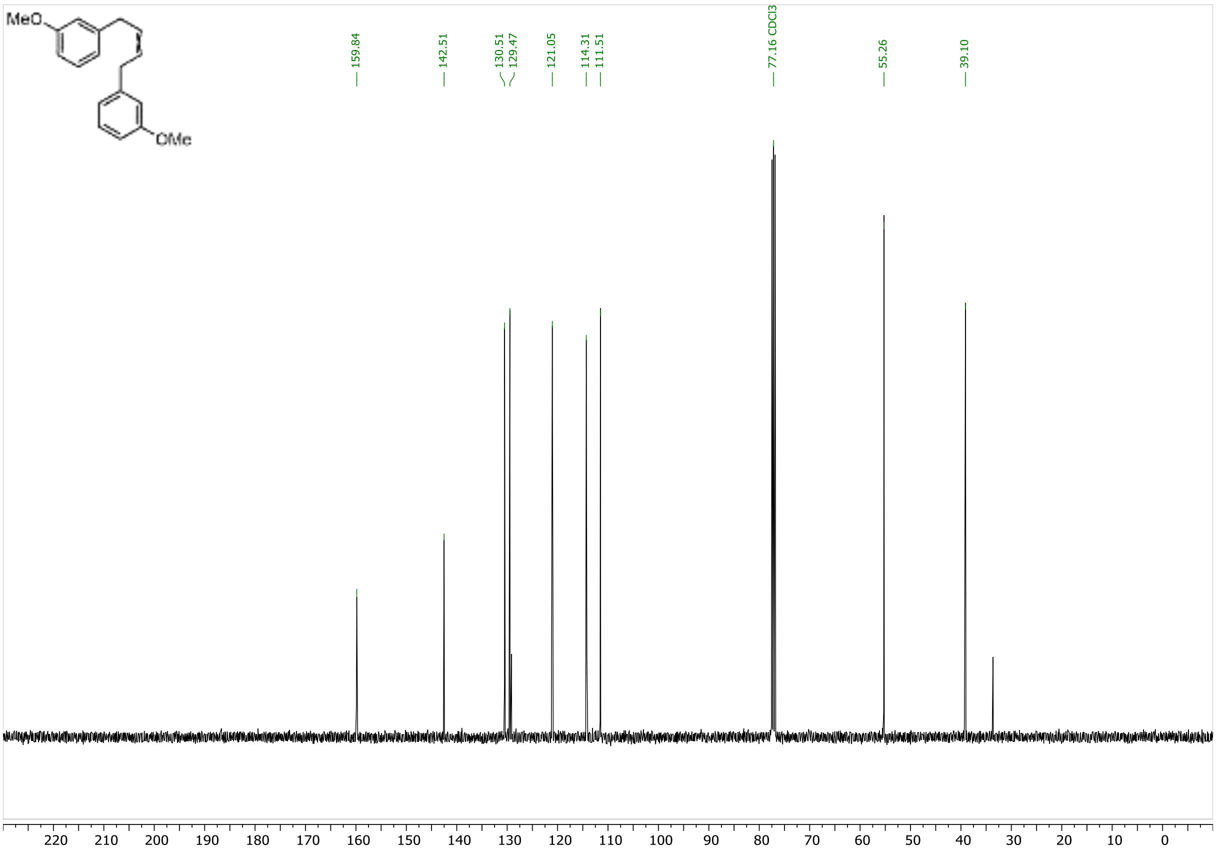

2st, ^{1}H NMR (400 MHz, $CDCl_3$)

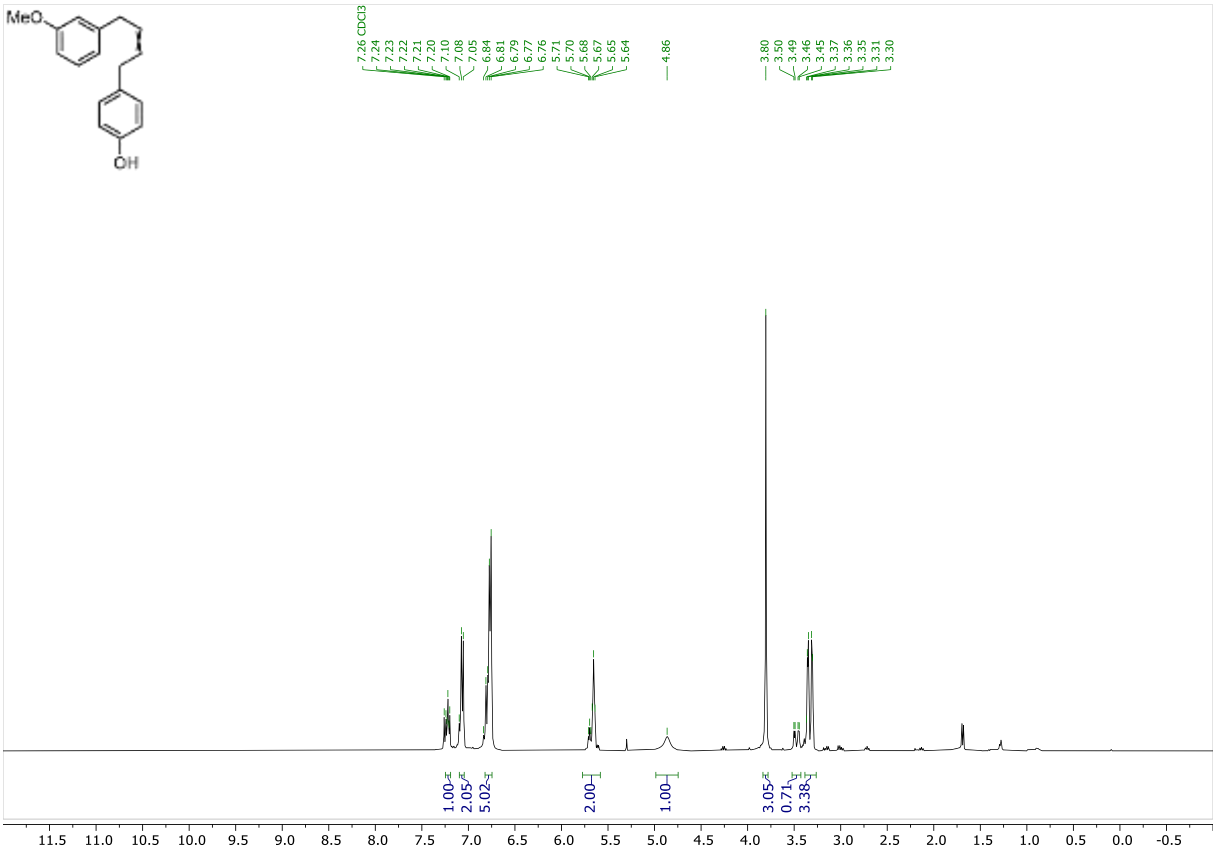

2st, $^{13}C\{^{1}H\}$ NMR (101 MHz, $CDCl_3$)

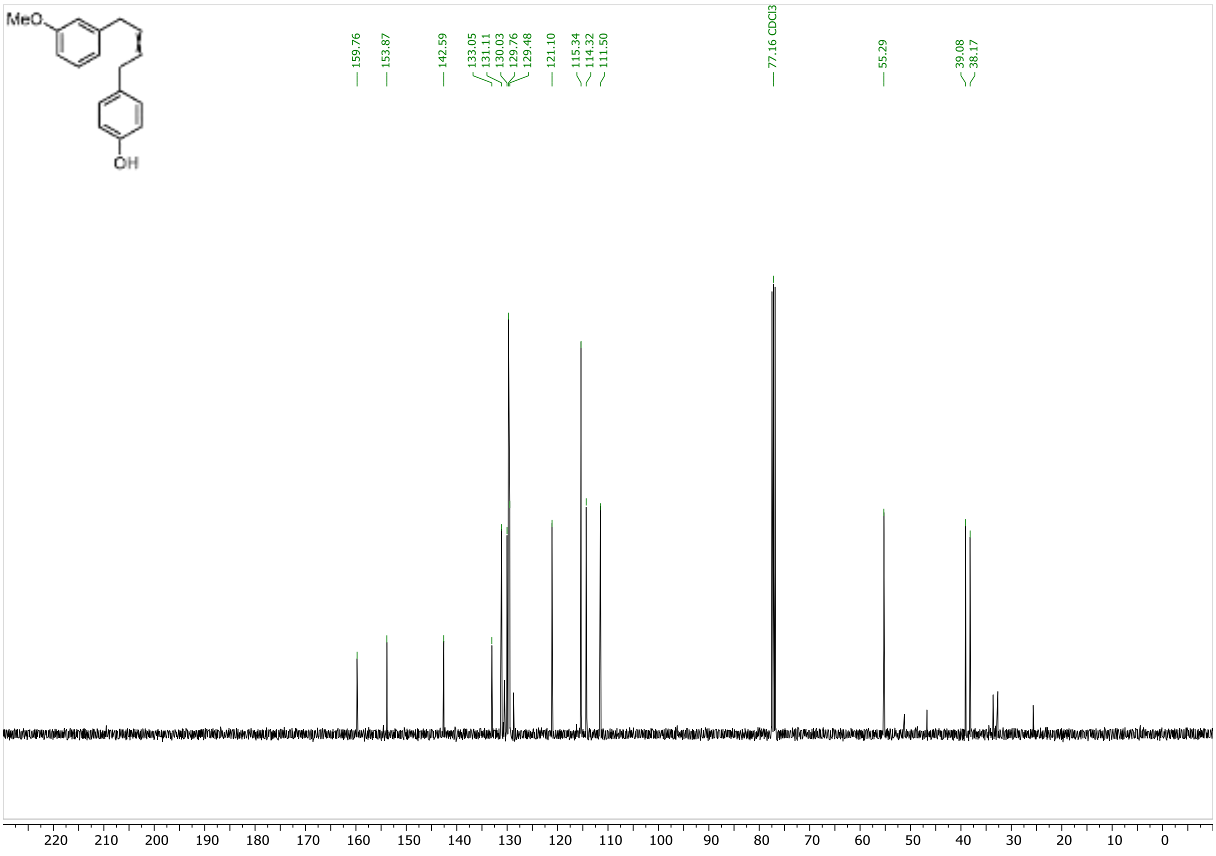

2rs, ^{1}H NMR (400 MHz, $CDCl_3$)

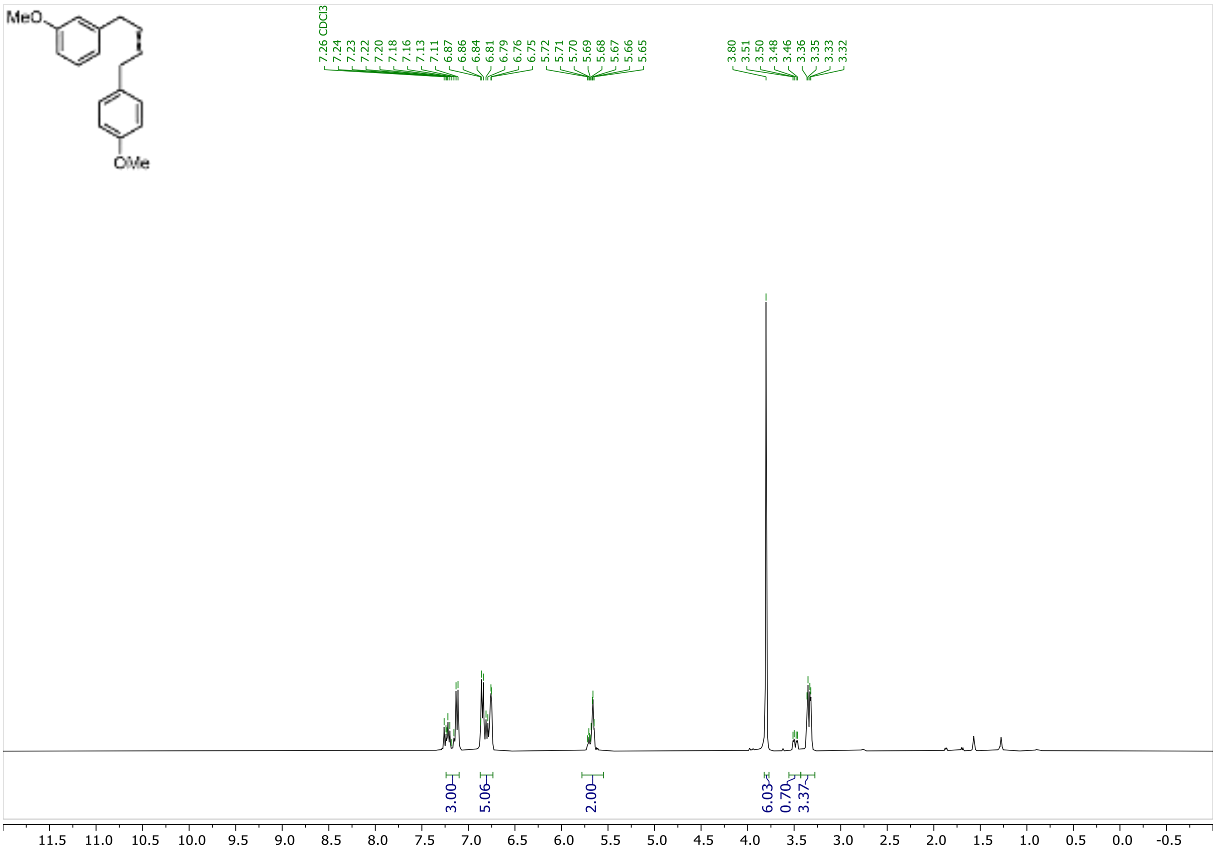

2rs, ^{13}C{^{1}H} NMR (101 MHz, $CDCl_3$)

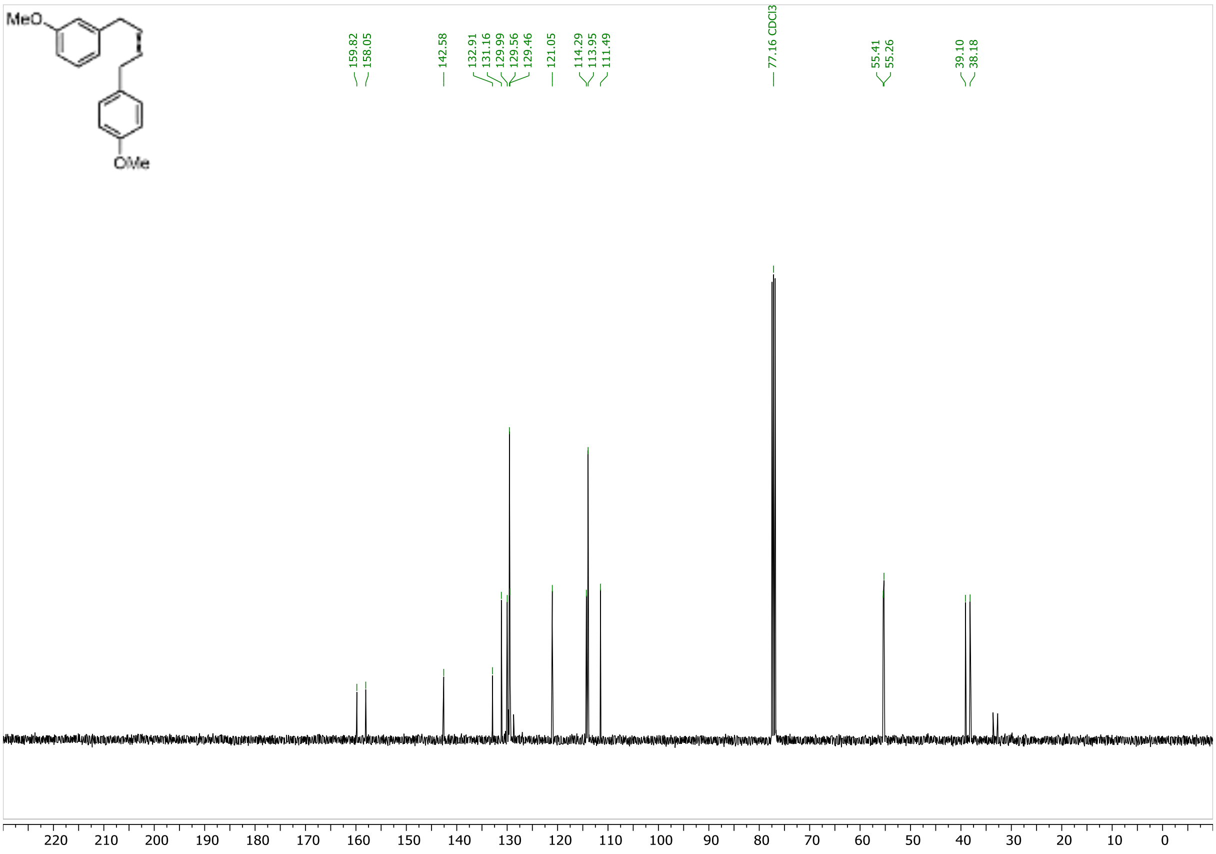

2ar, ^{1}H NMR (400 MHz, $CDCl_3$)

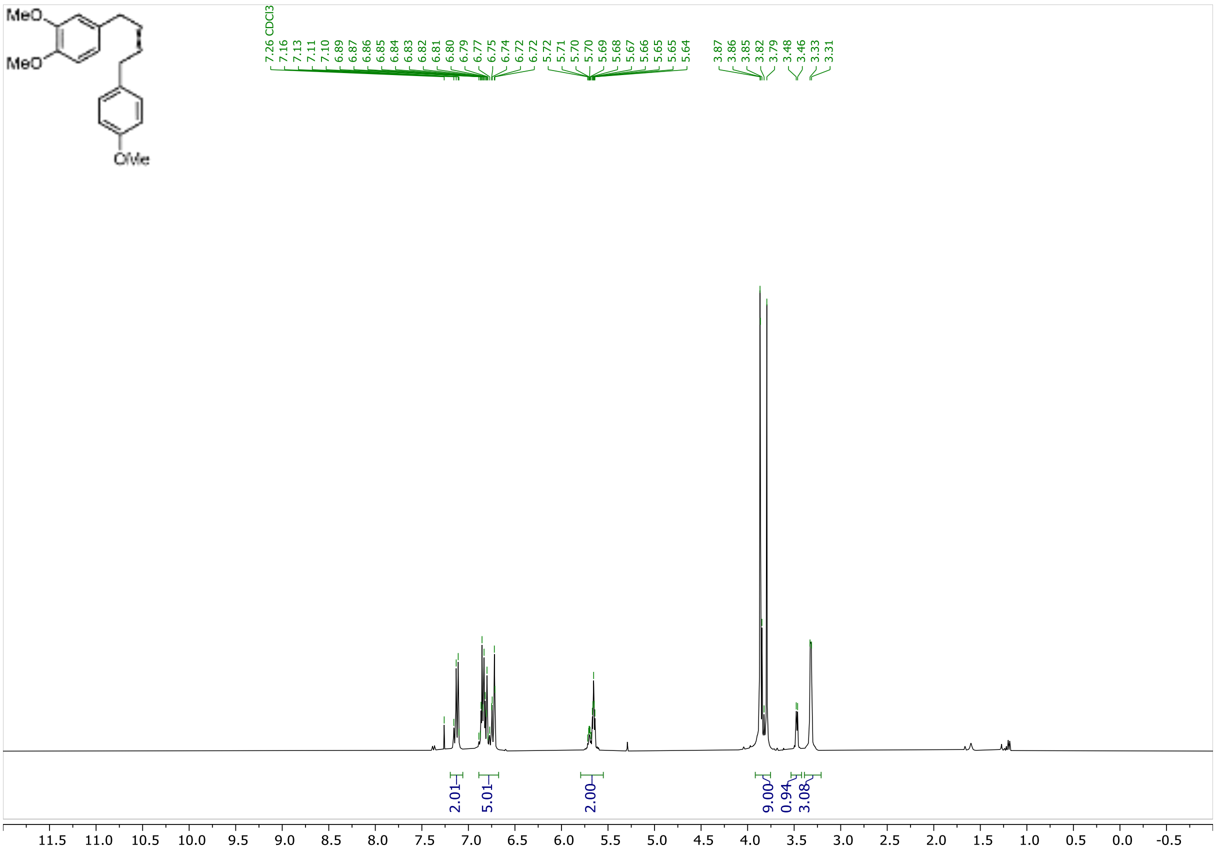

2ar, ^{13}C{^{1}H} NMR (101 MHz, $CDCl_3$)

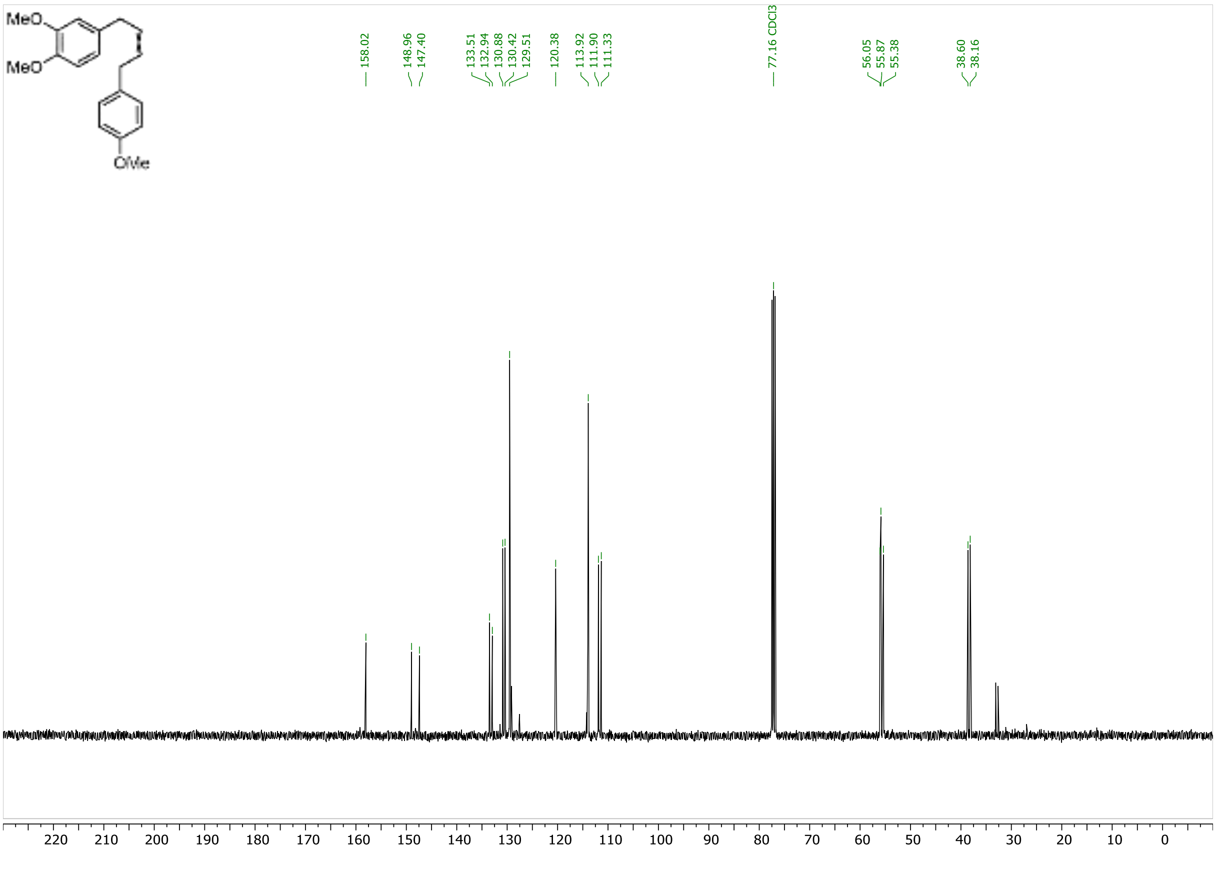

2r, ^{1}H NMR (400 MHz, $CDCl_3$)

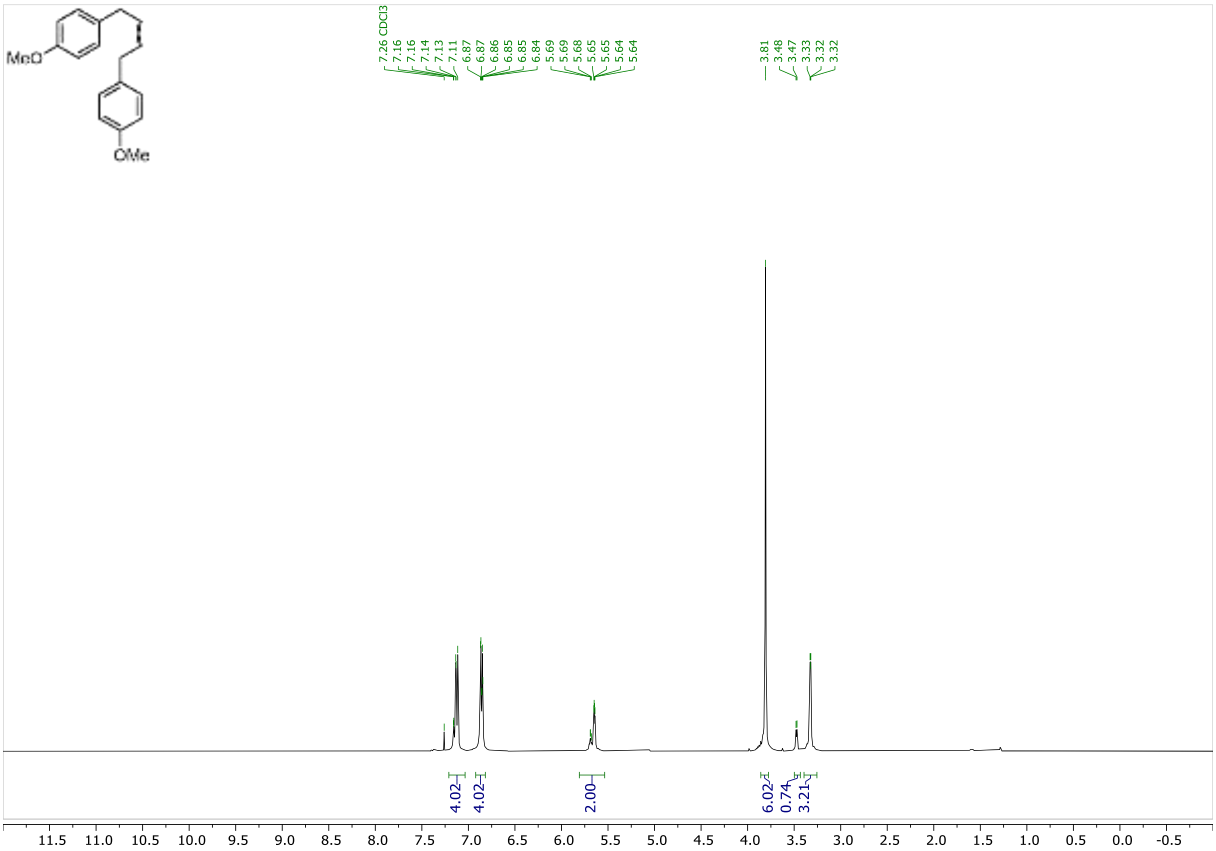

2r, ^{13}C{^{1}H} NMR (101 MHz, $CDCl_3$)

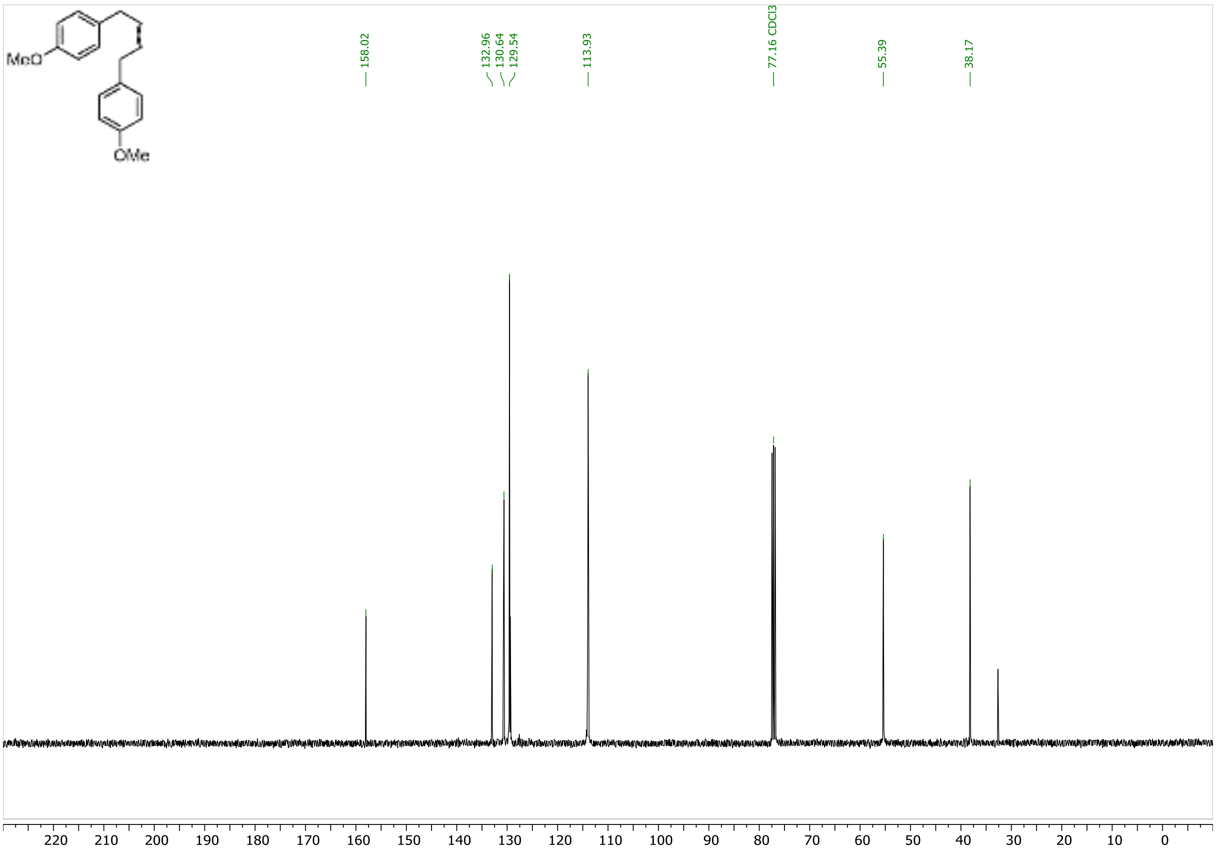

3a, ^{1}H NMR (400 MHz, $CDCl_3$)

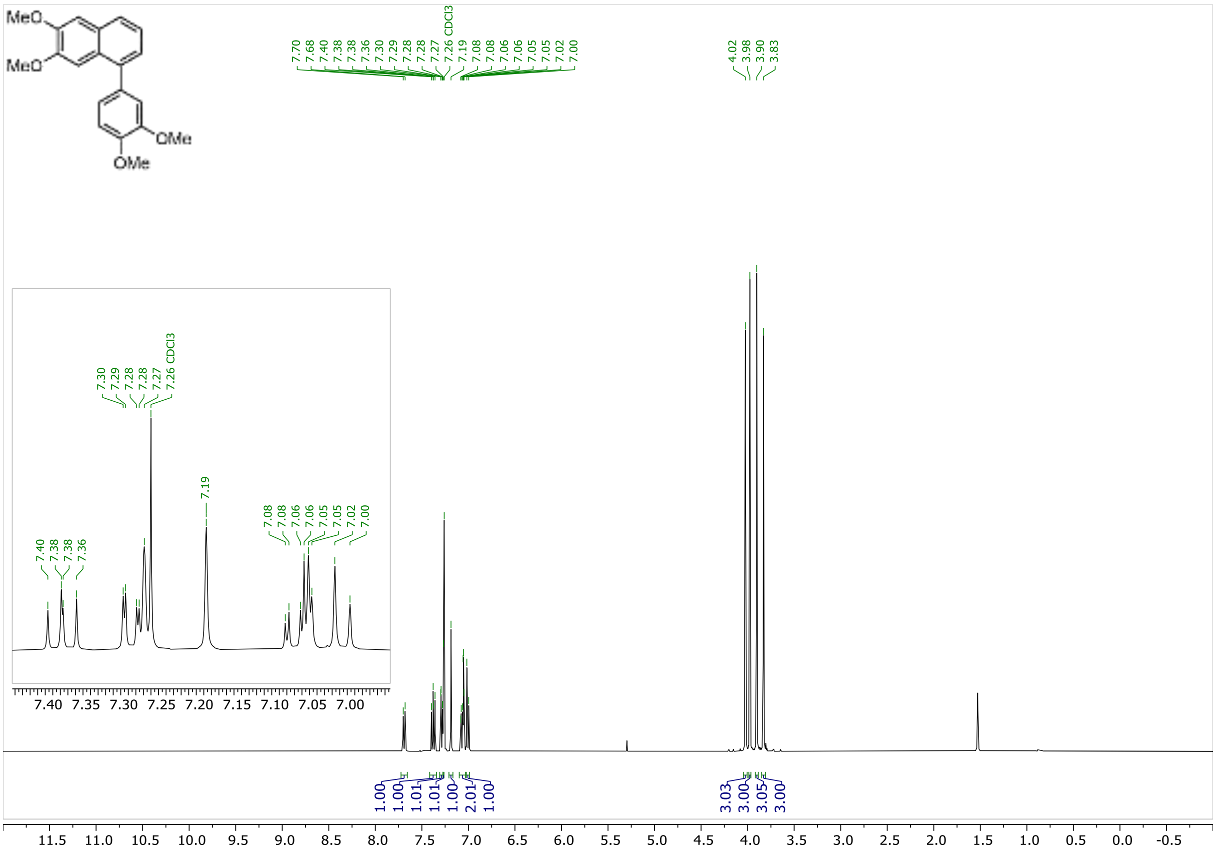

3a, $^{13}C\{^{1}H\}$ NMR (101 MHz, $CDCl_3$)

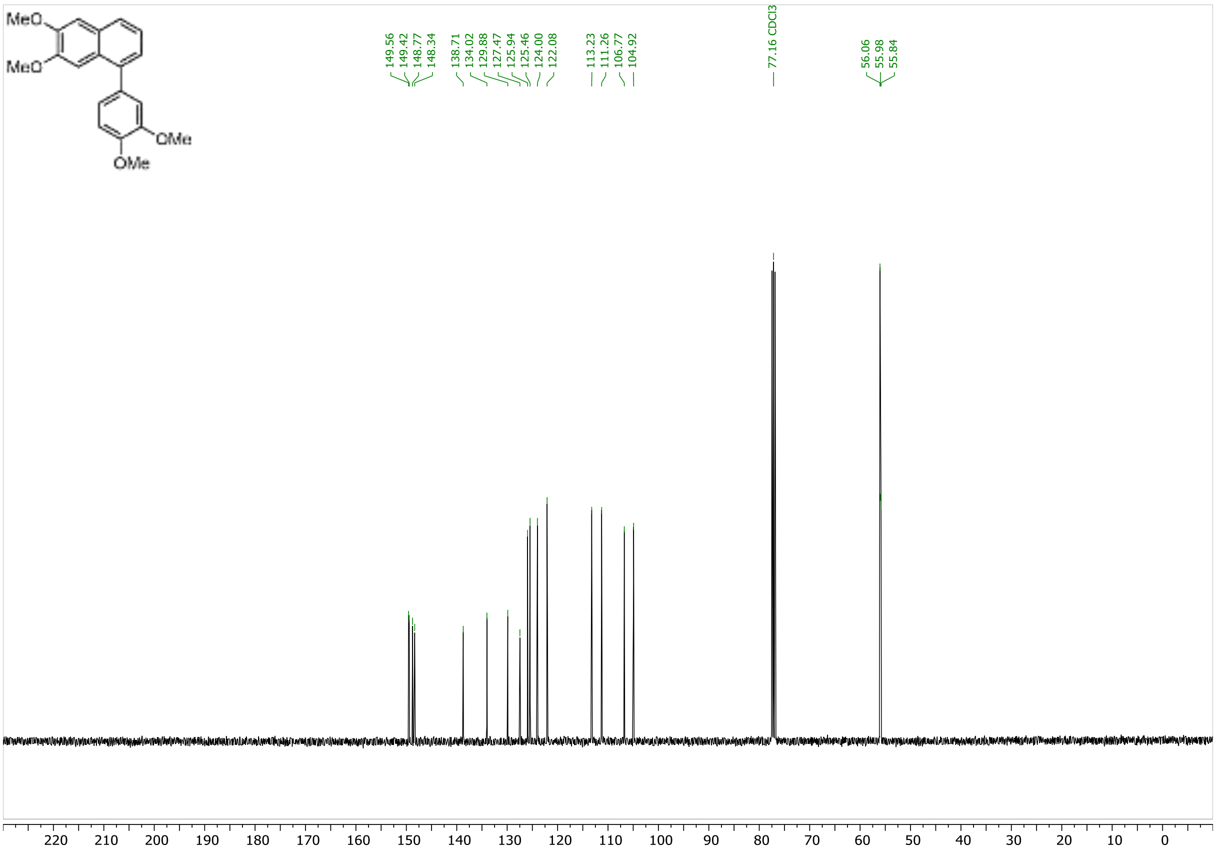

3b, ^{1}H NMR (400 MHz, $CDCl_3$)

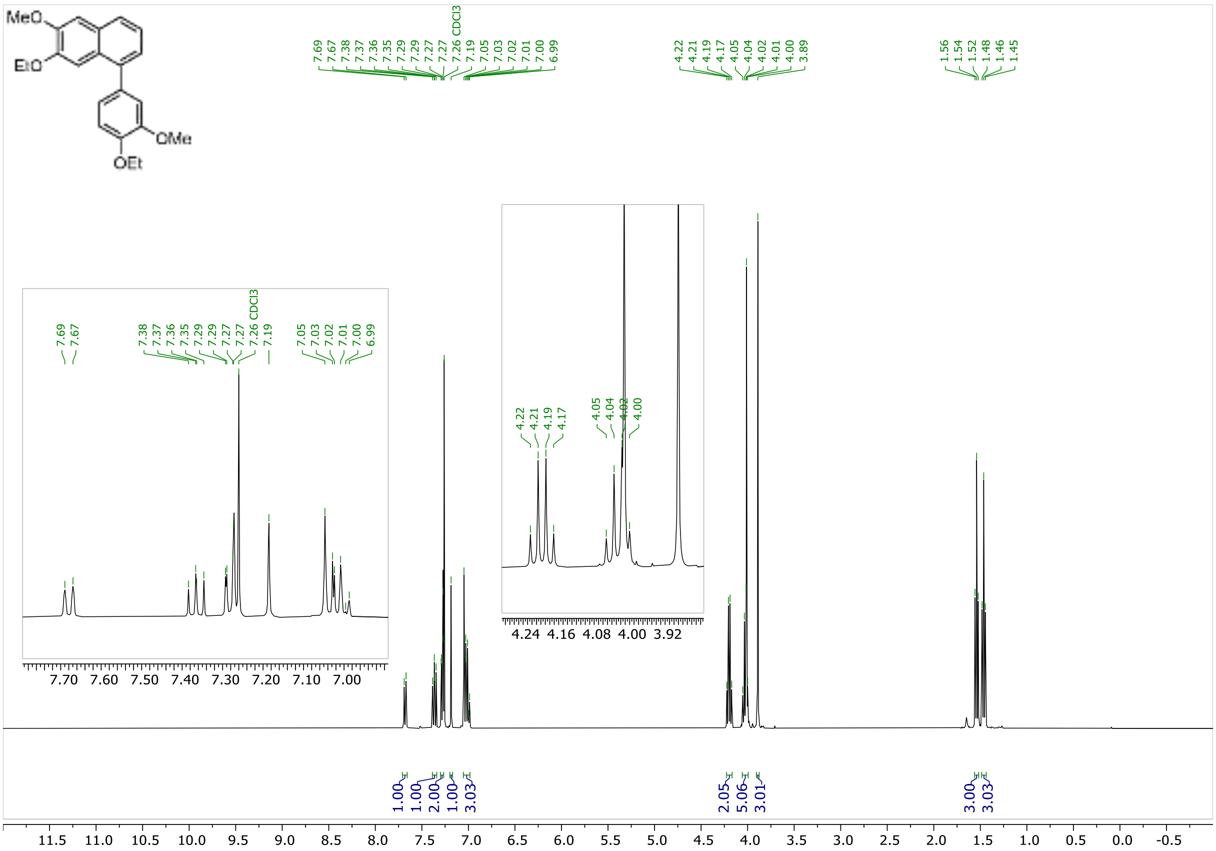

3b, $^{13}C\{^{1}H\}$ NMR (101 MHz, $CDCl_3$)

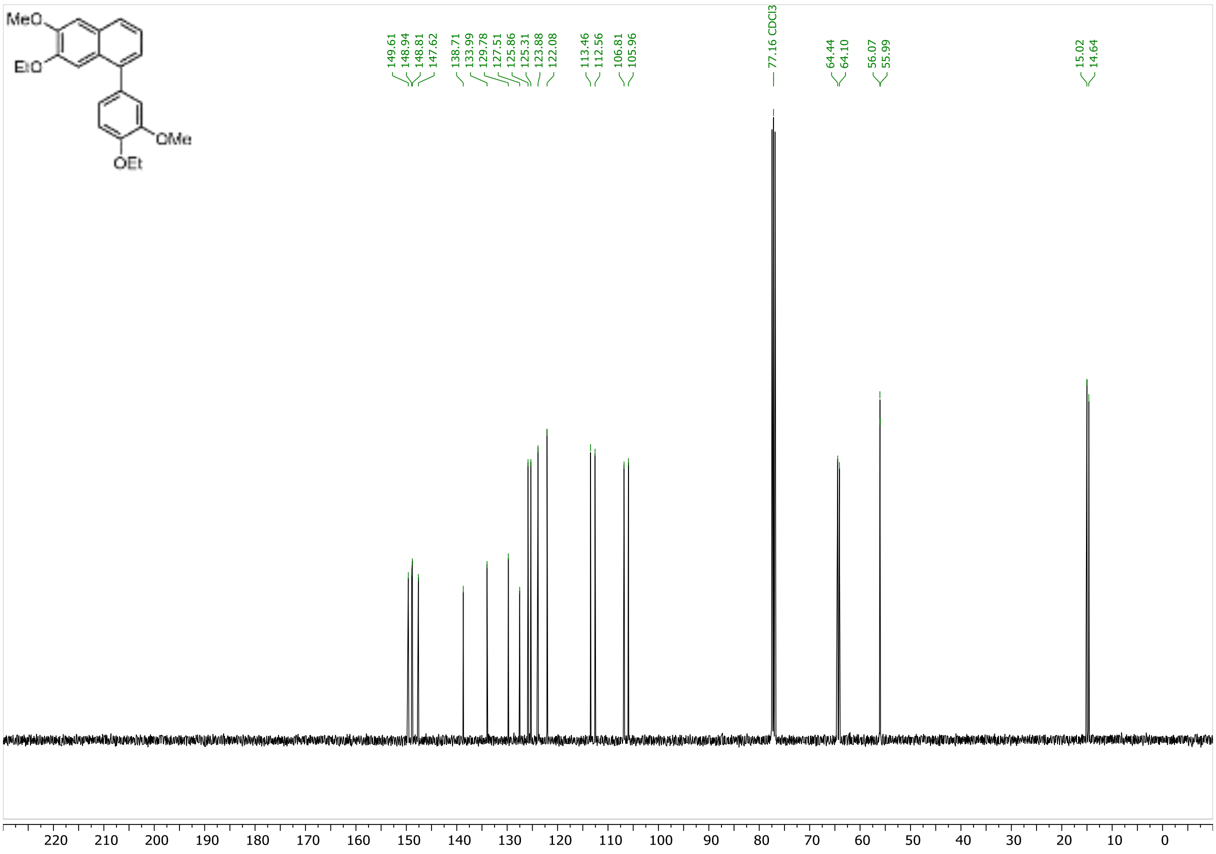

3c, ^{1}H NMR (400 MHz, $CDCl_3$)

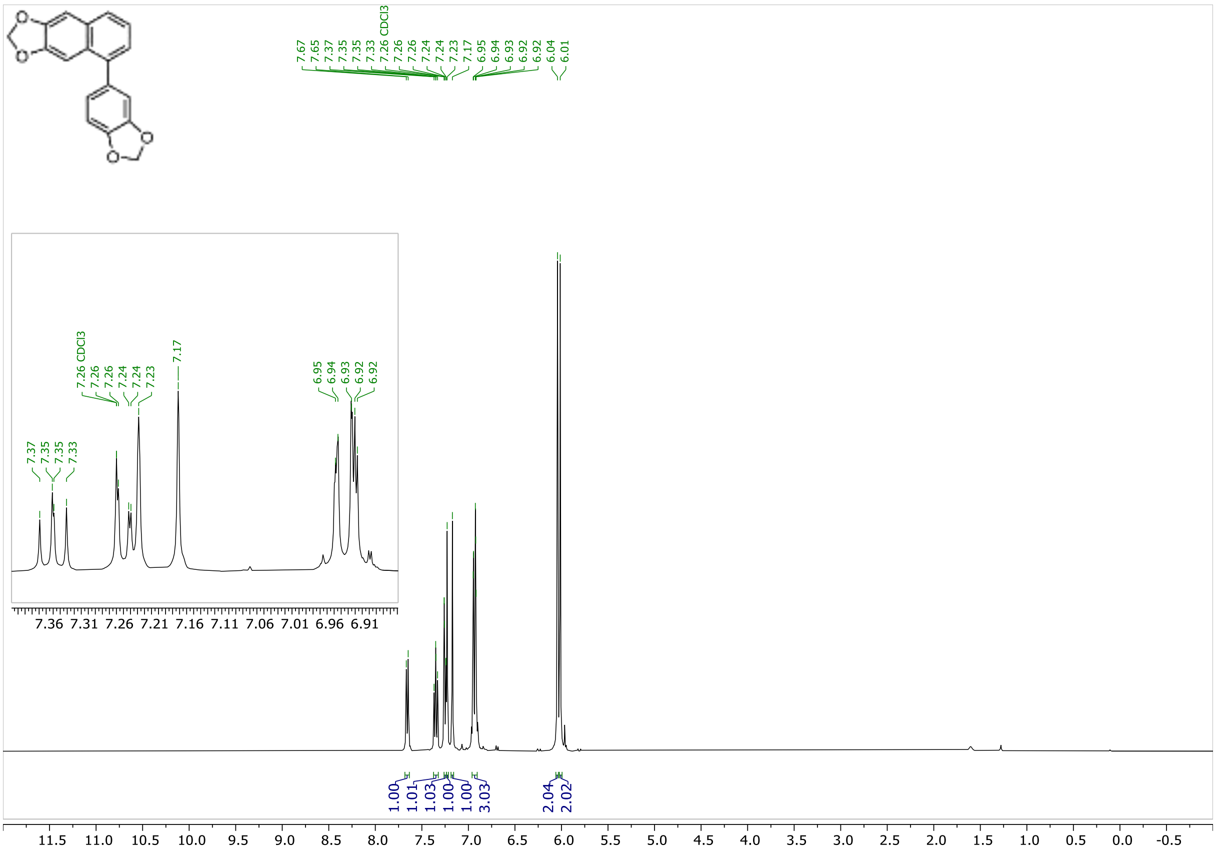

3c, ^{13}C{^{1}H} NMR (101 MHz, $CDCl_3$)

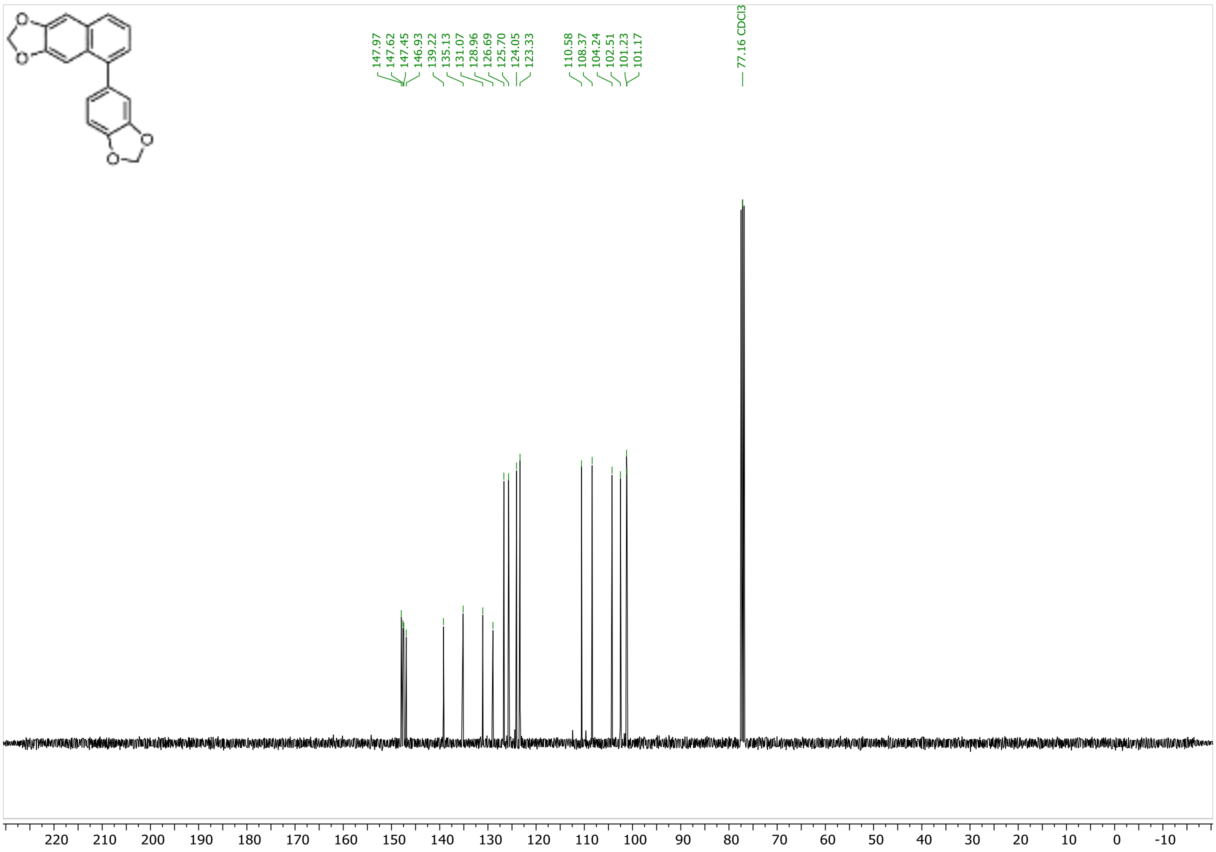

3d, ^{1}H NMR (400 MHz, $CDCl_3$)

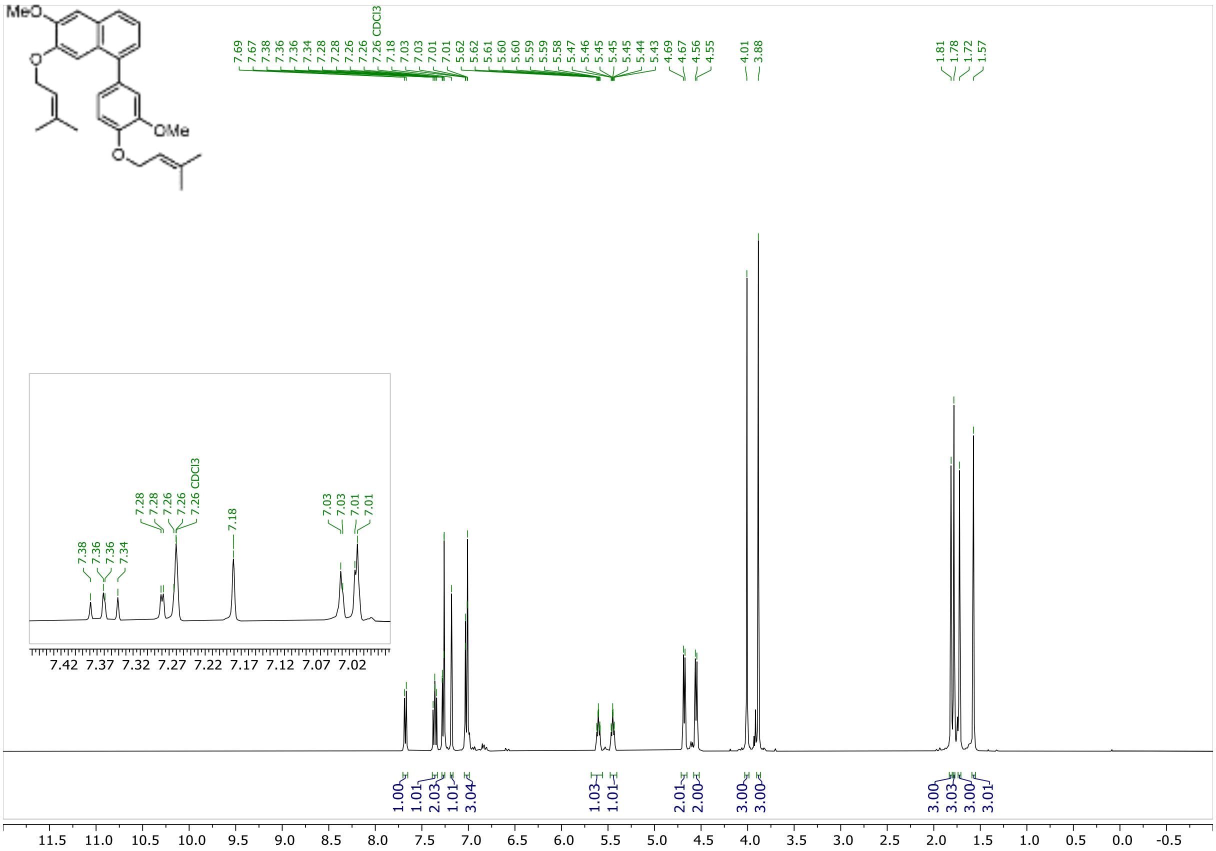

3d, ^{13}C{^{1}H} NMR (101 MHz, $CDCl_3$)

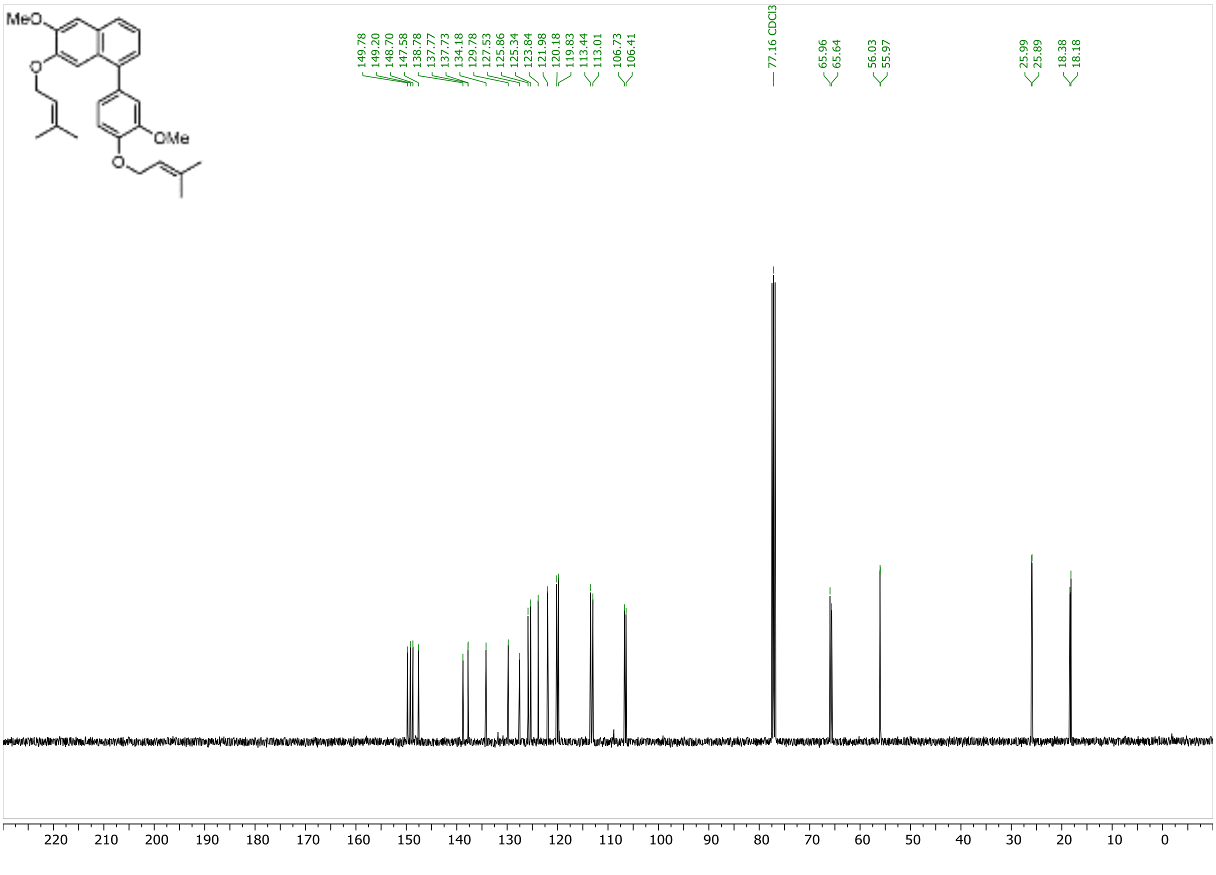

3e, ^{1}H NMR (400 MHz, $CDCl_3$)

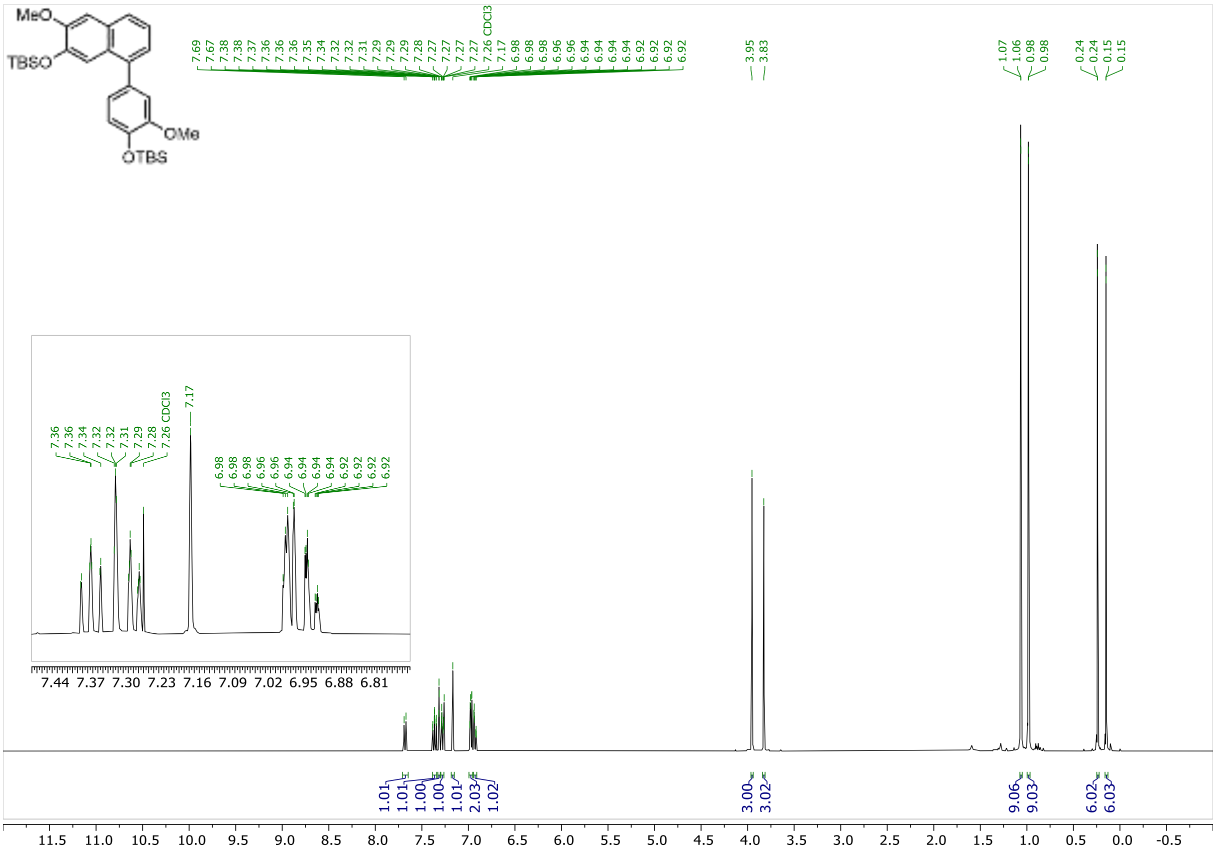

3e, ^{13}C{^{1}H} NMR (101 MHz, $CDCl_3$)

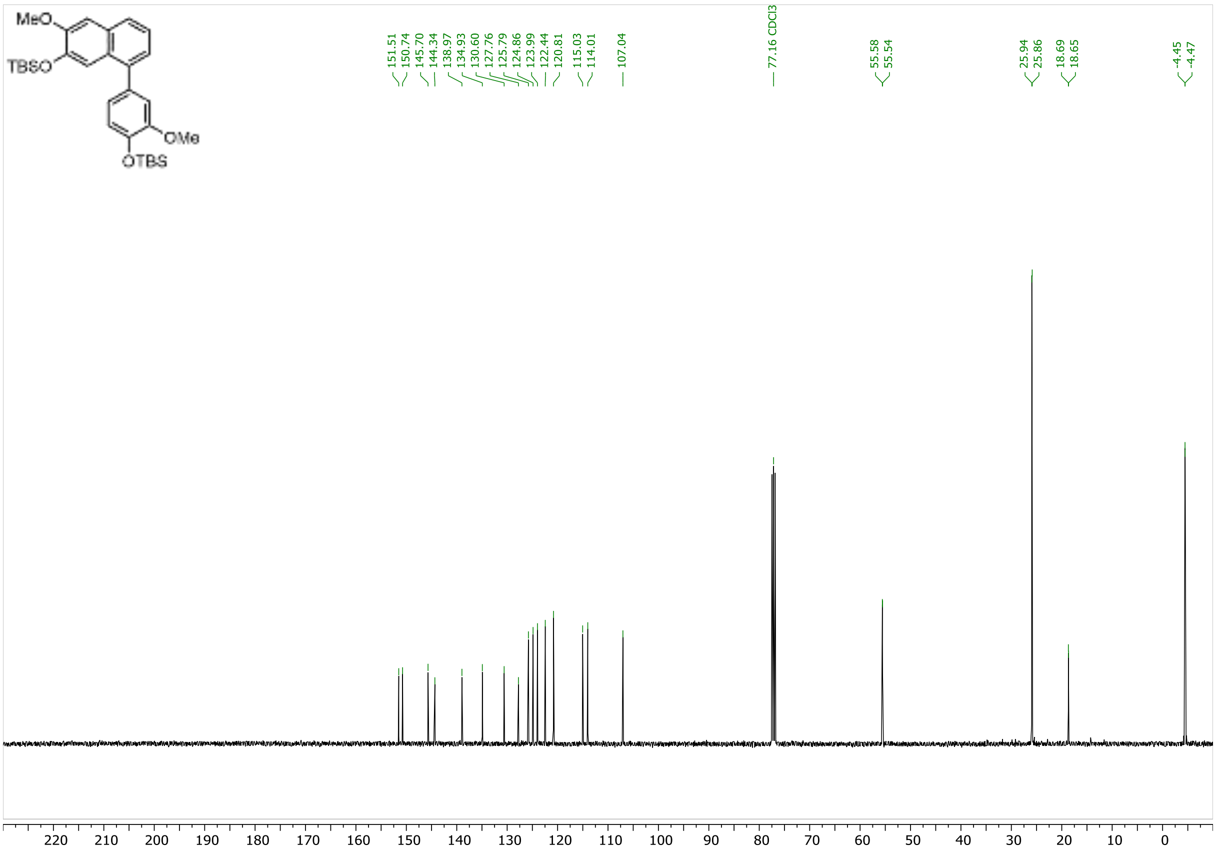

3f, ^{1}H NMR (400 MHz, $CDCl_3$)

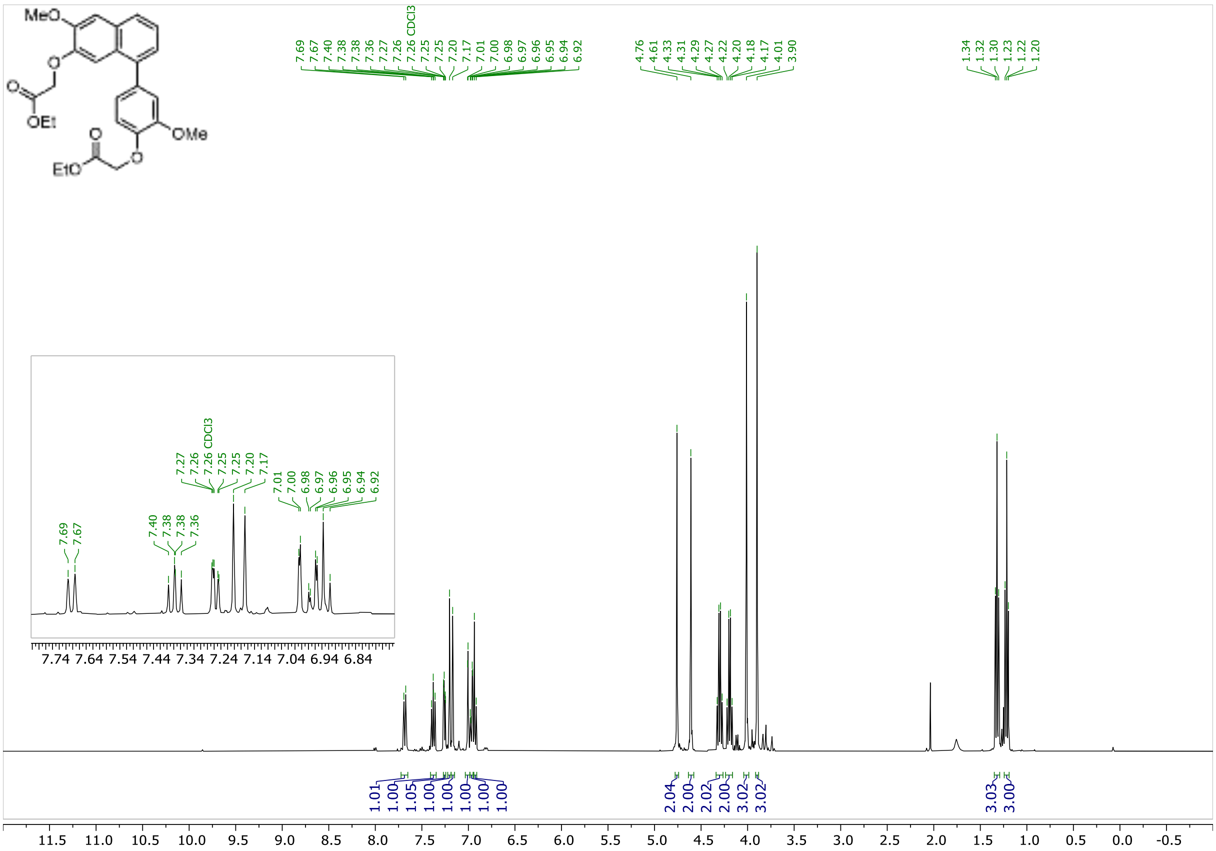

3f, ^{13}C{^{1}H} NMR (101 MHz, $CDCl_3$)

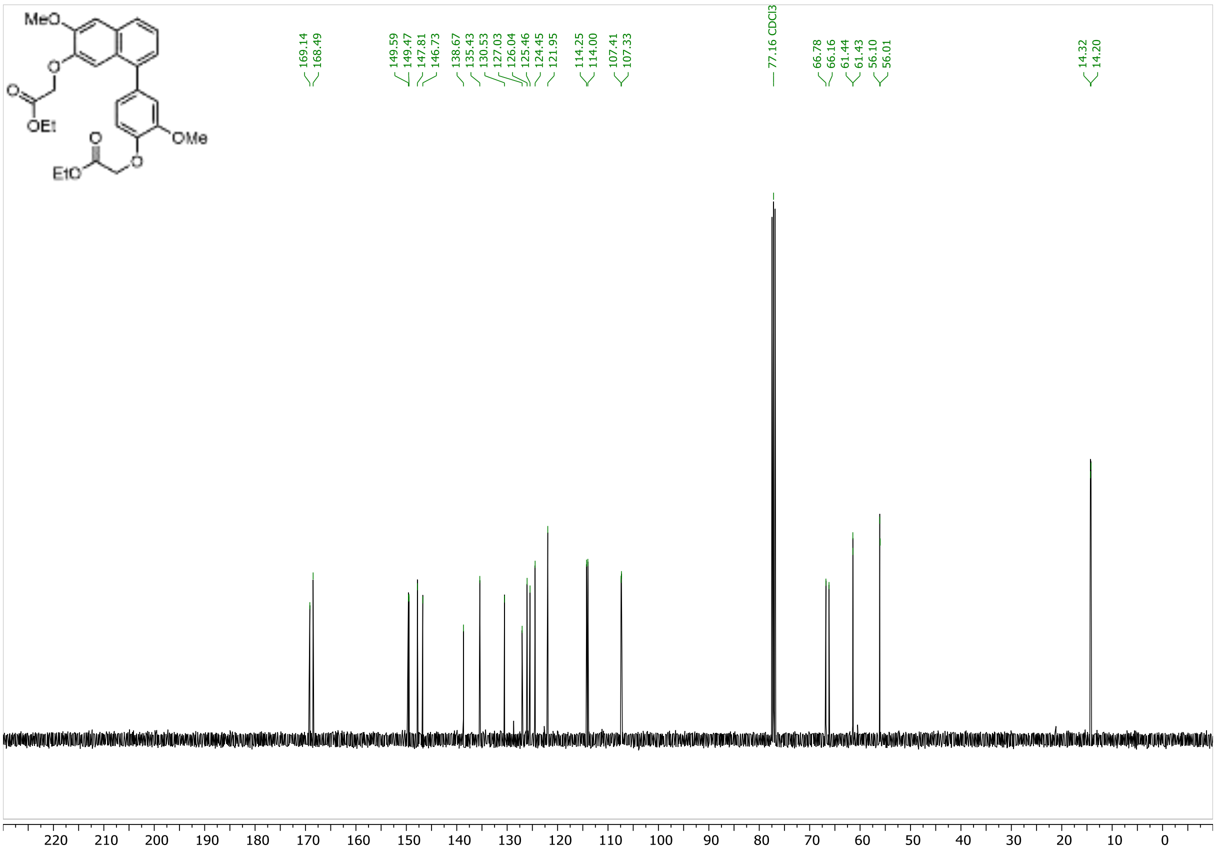

3g, ^{1}H NMR (400 MHz, MeOD)

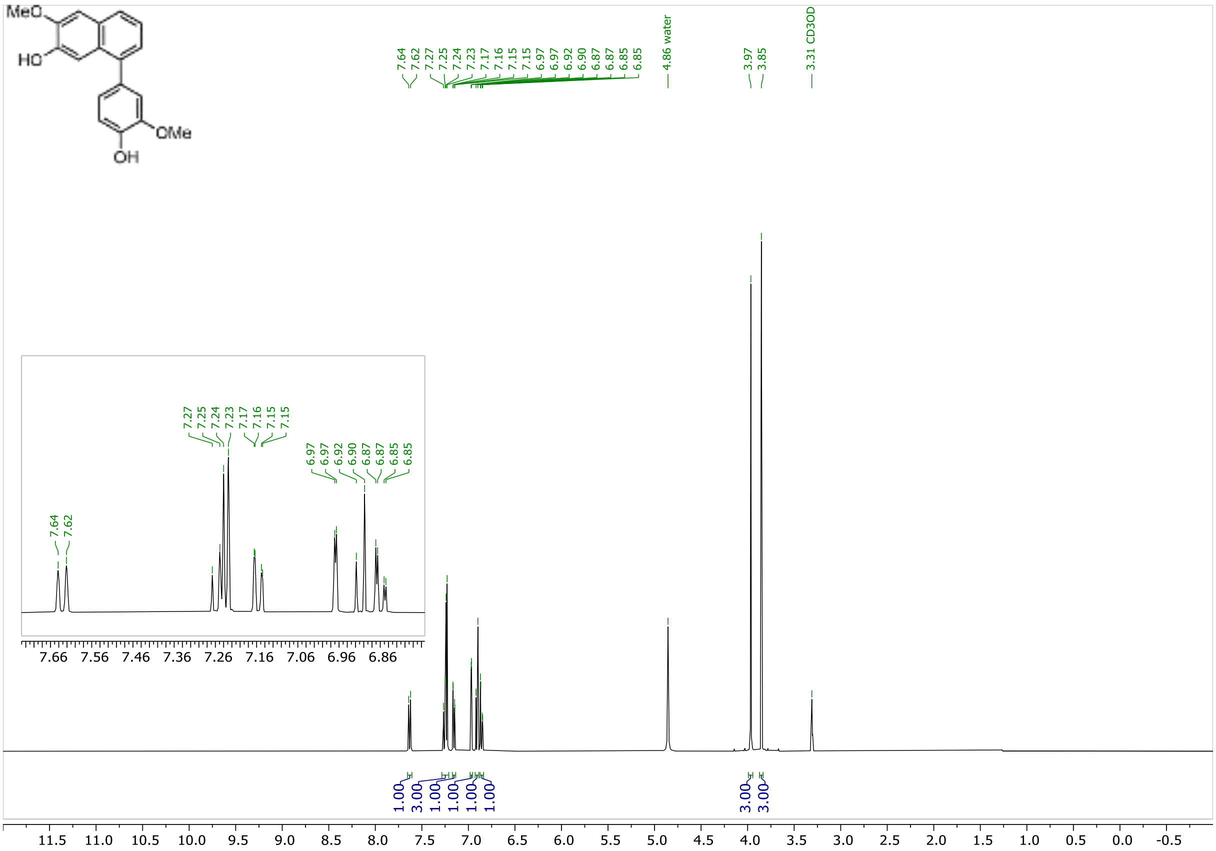

3g, ^{13}C{^{1}H} NMR (101 MHz, MeOD)

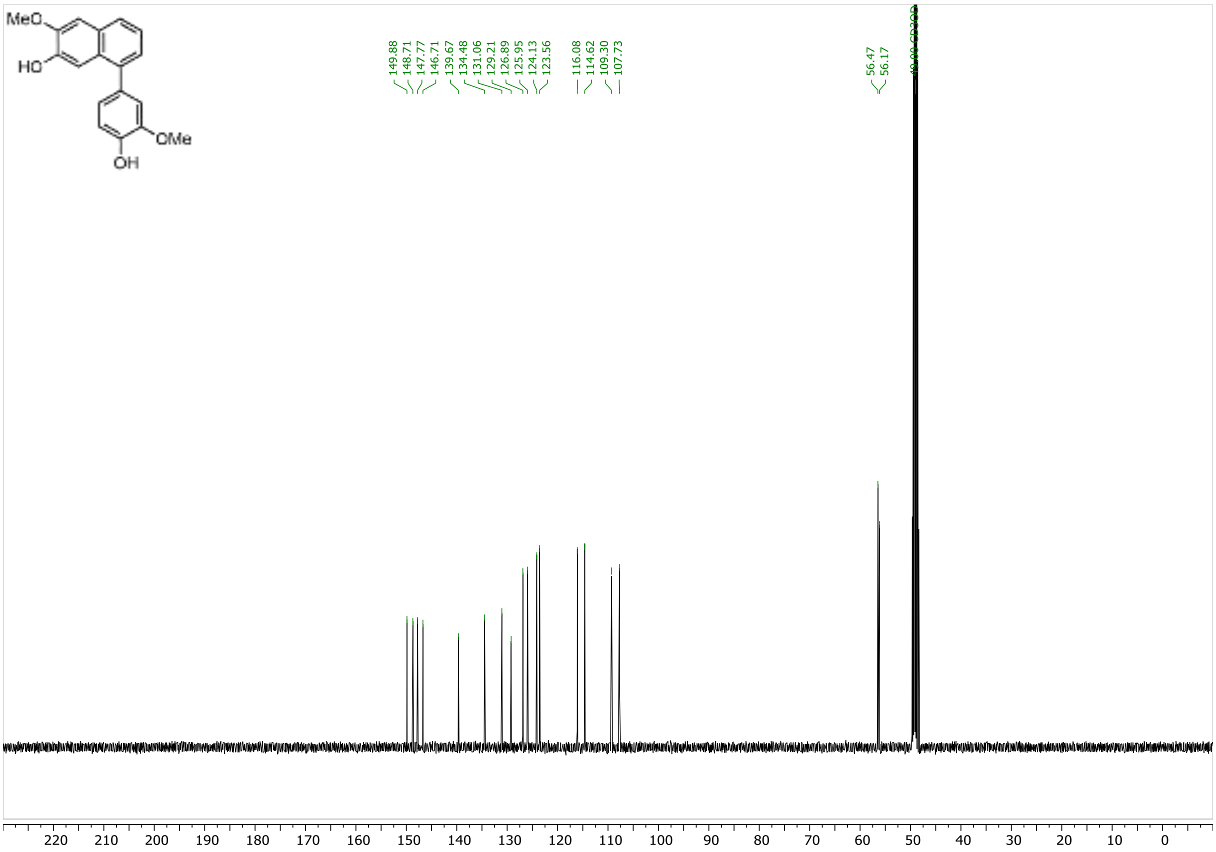

3g, ^{1}H NMR (400 MHz, DMSO-*d*6)

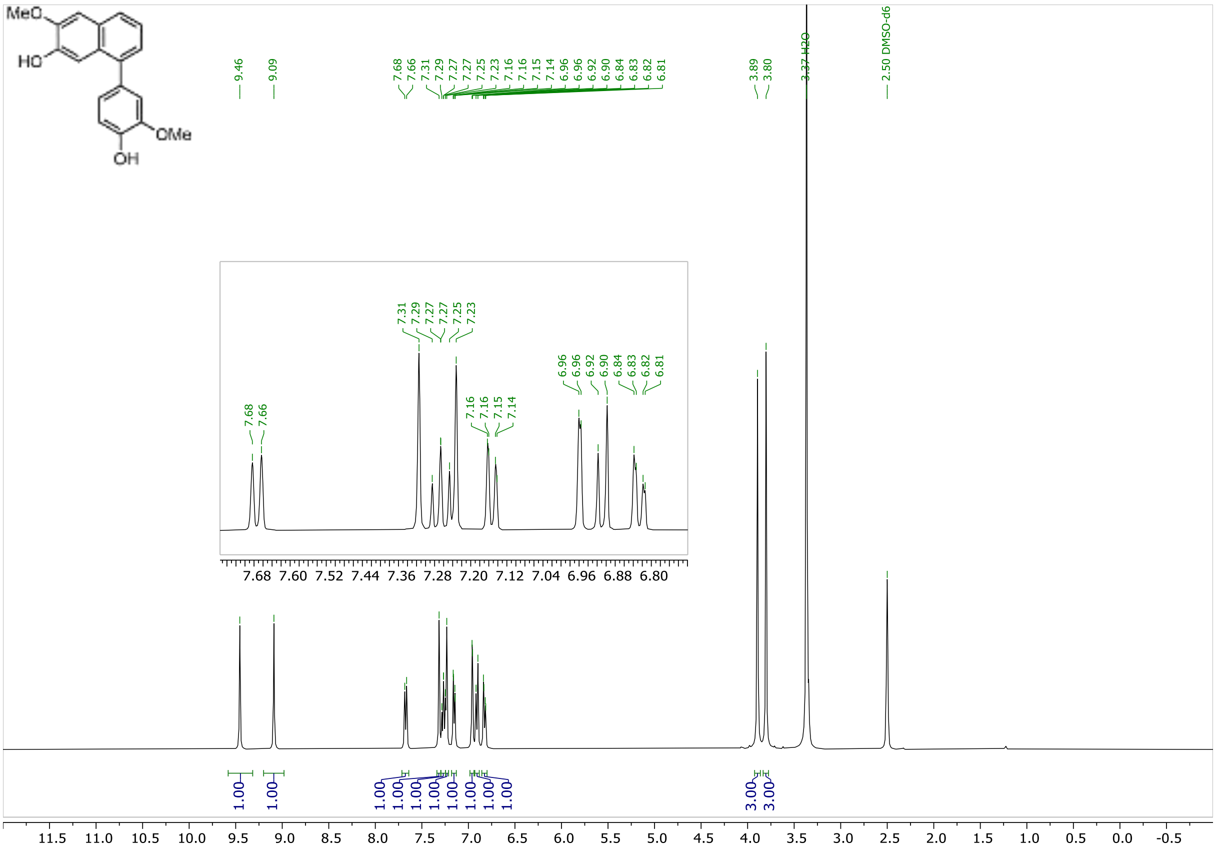

3g, ^{13}C{^{1}H} NMR (101 MHz, DMSO-*d*6)

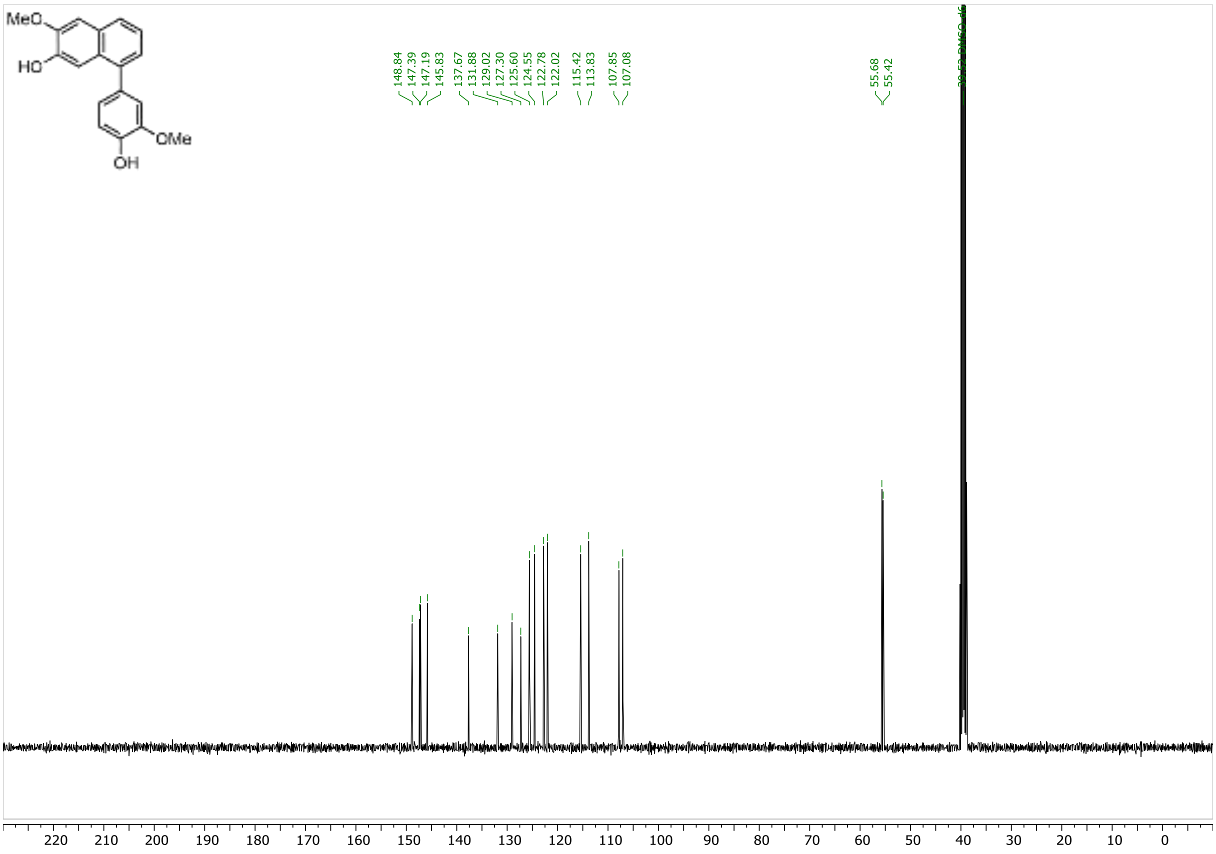

3h, ^{1}H NMR (400 MHz, $CDCl_3$)

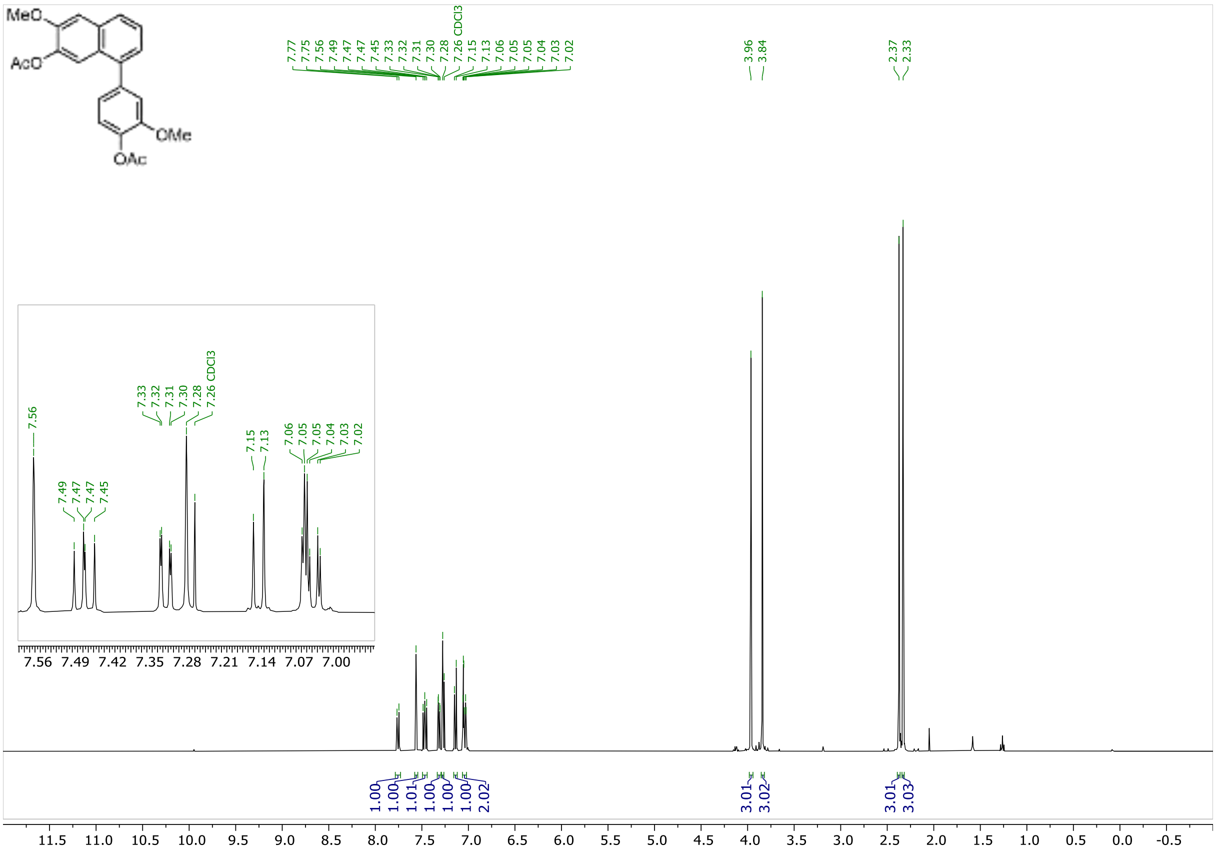

3h, $^{13}C\{^{1}H\}$ NMR (101 MHz, $CDCl_3$)

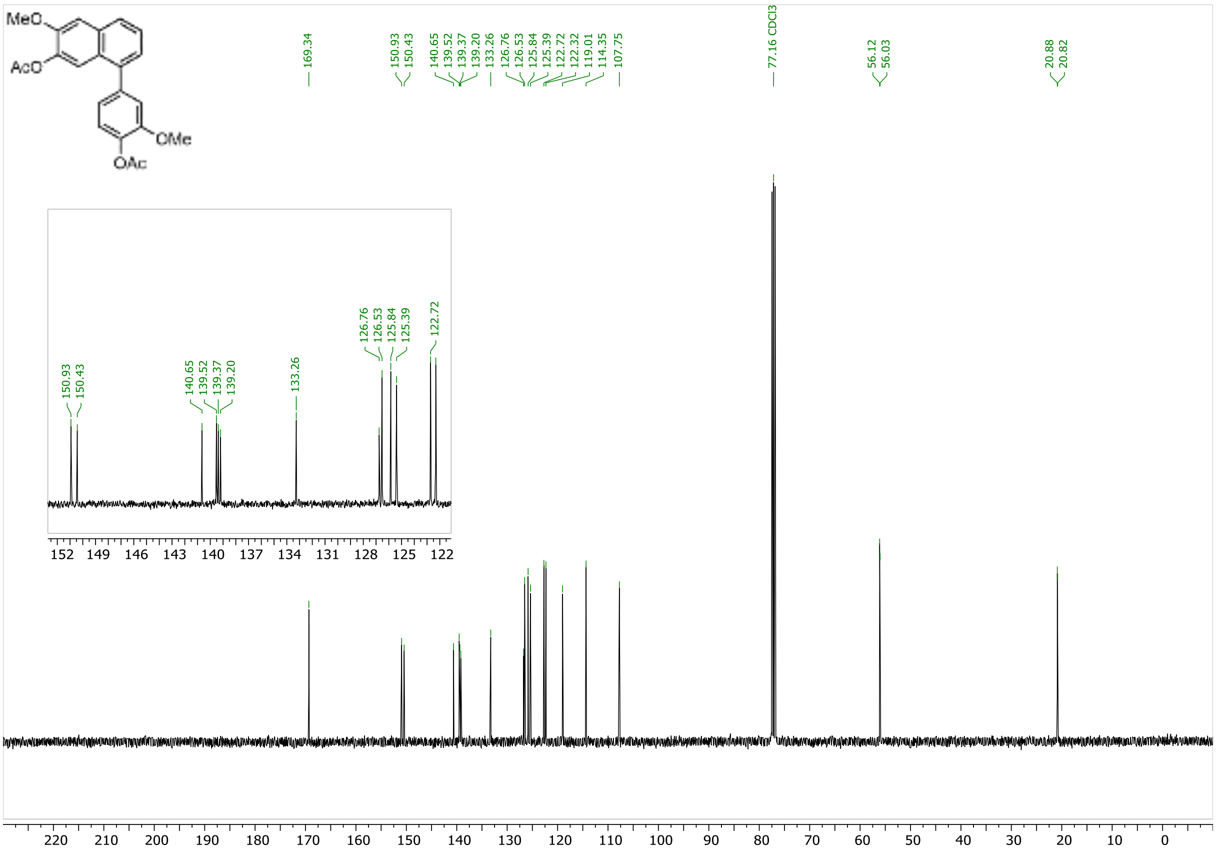

3i, ^{1}H NMR (400 MHz, $CDCl_3$)

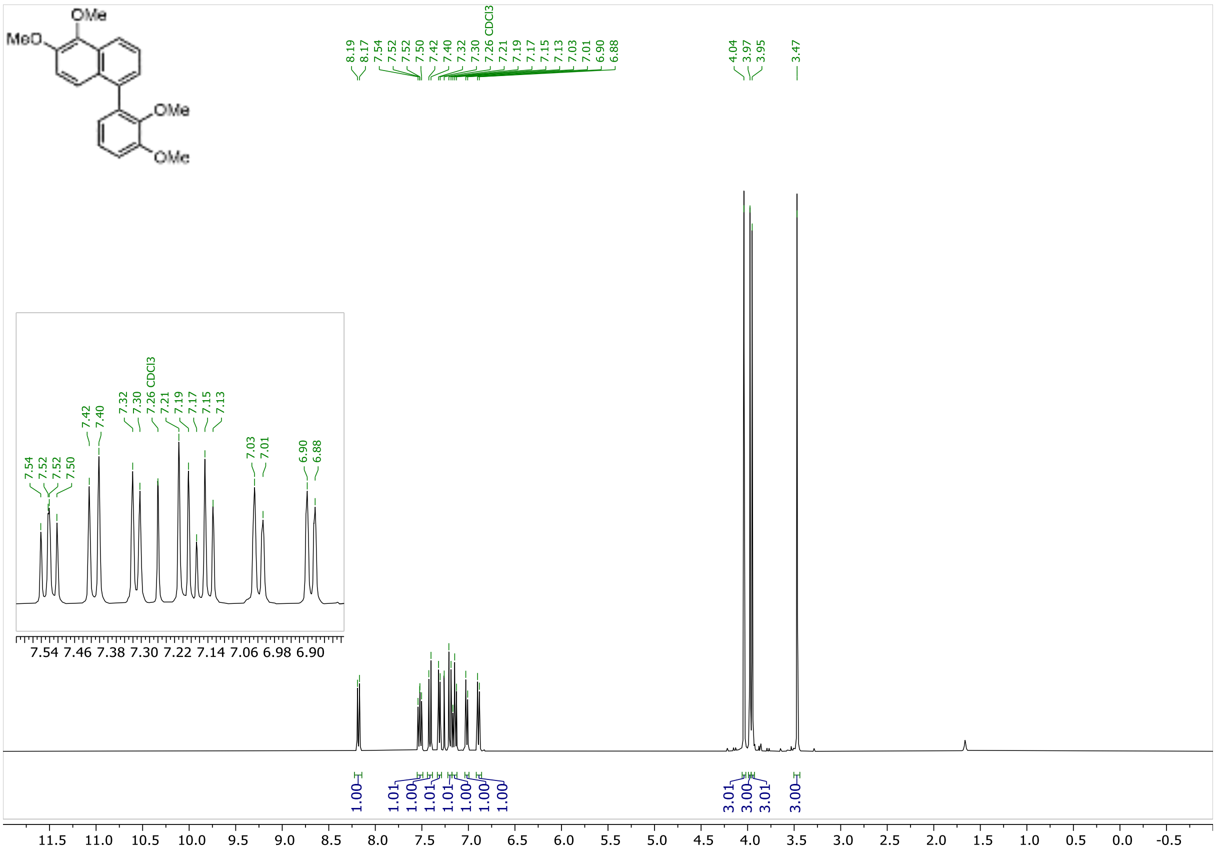

3i, ^{13}C{^{1}H} NMR (101 MHz, $CDCl_3$)

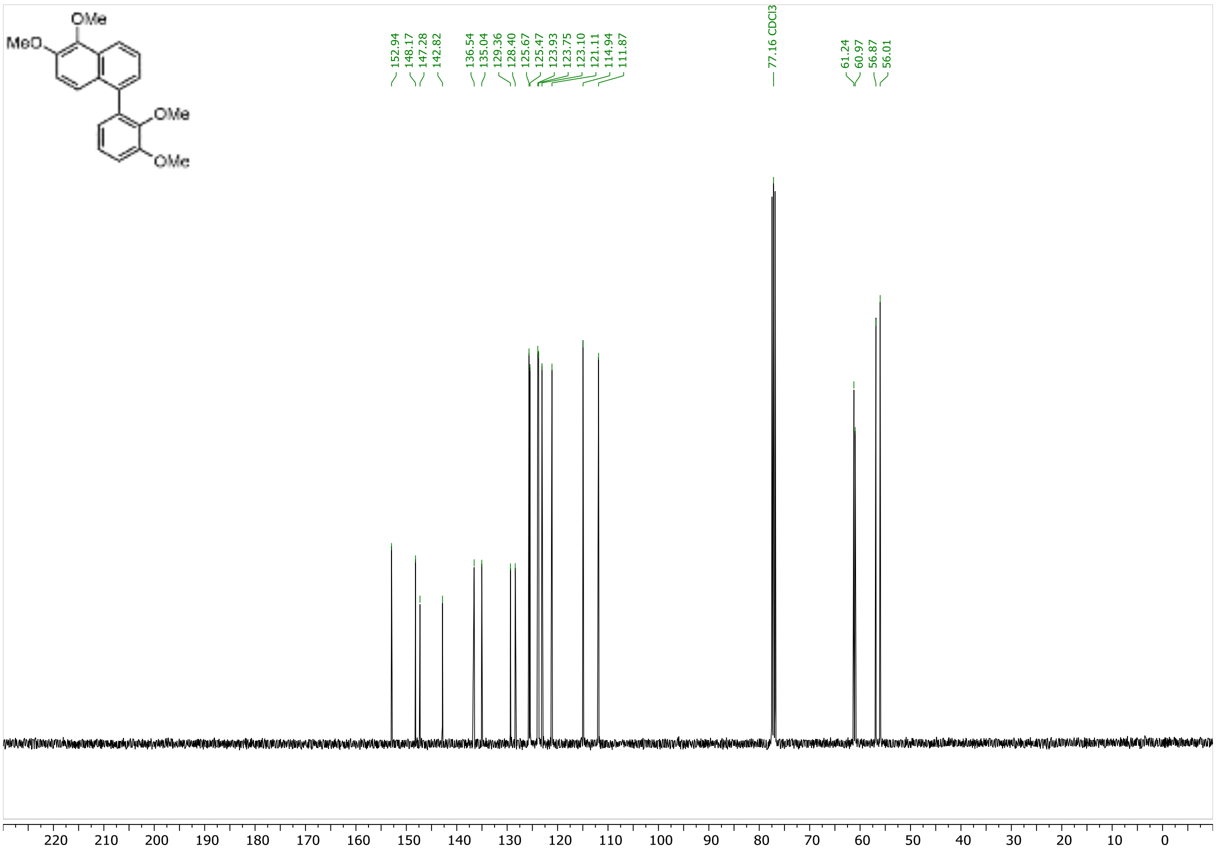

3j, ^{1}H NMR (400 MHz, $CDCl_3$)

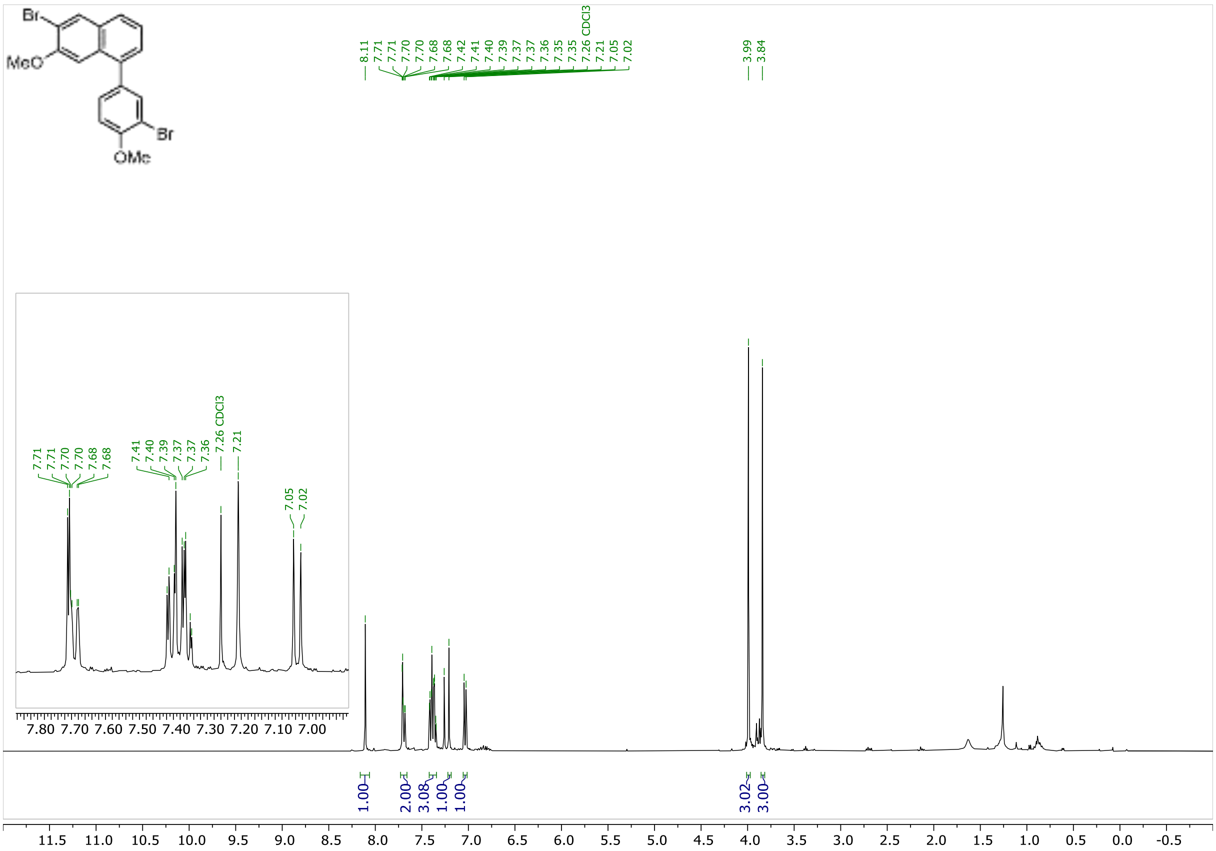

3j, ^{13}C{^{1}H} NMR (101 MHz, $CDCl_3$)

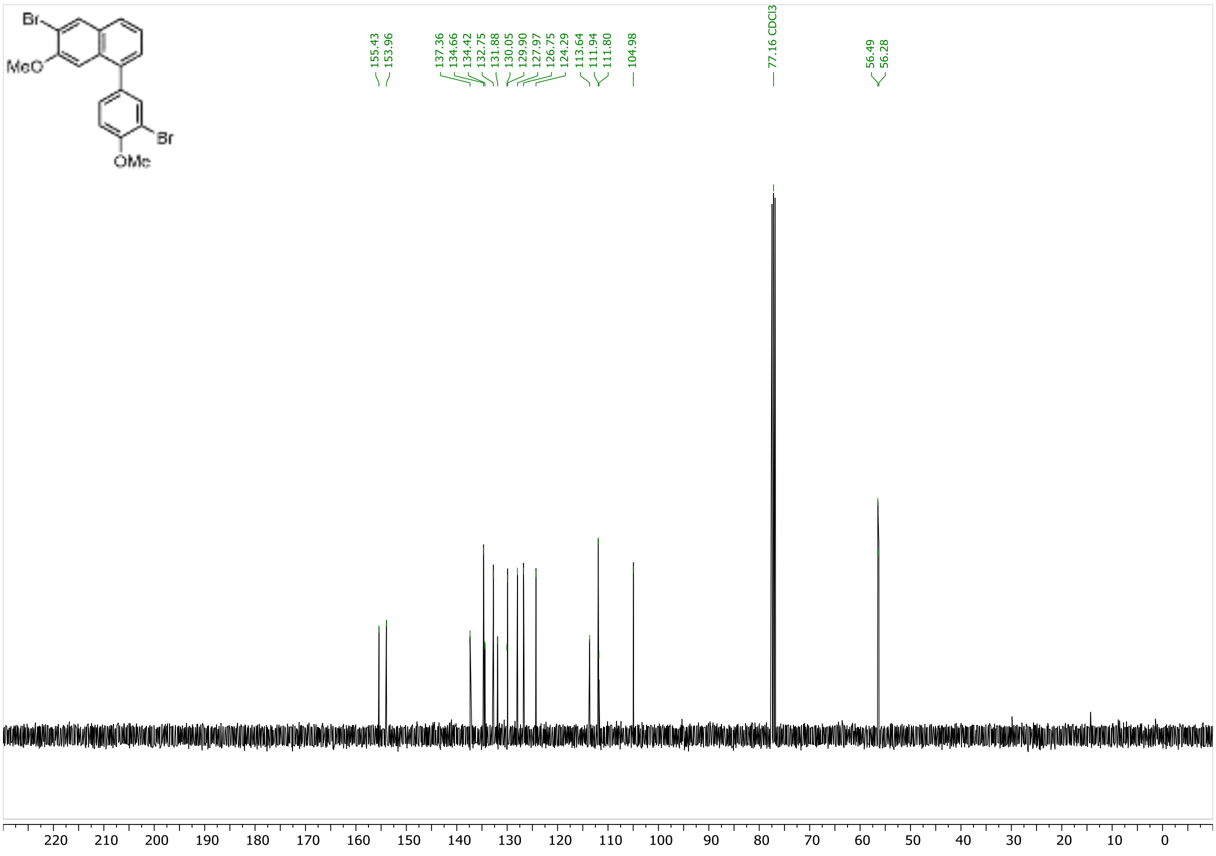

3k, ^{1}H NMR (400 MHz, CDCl$_3$)

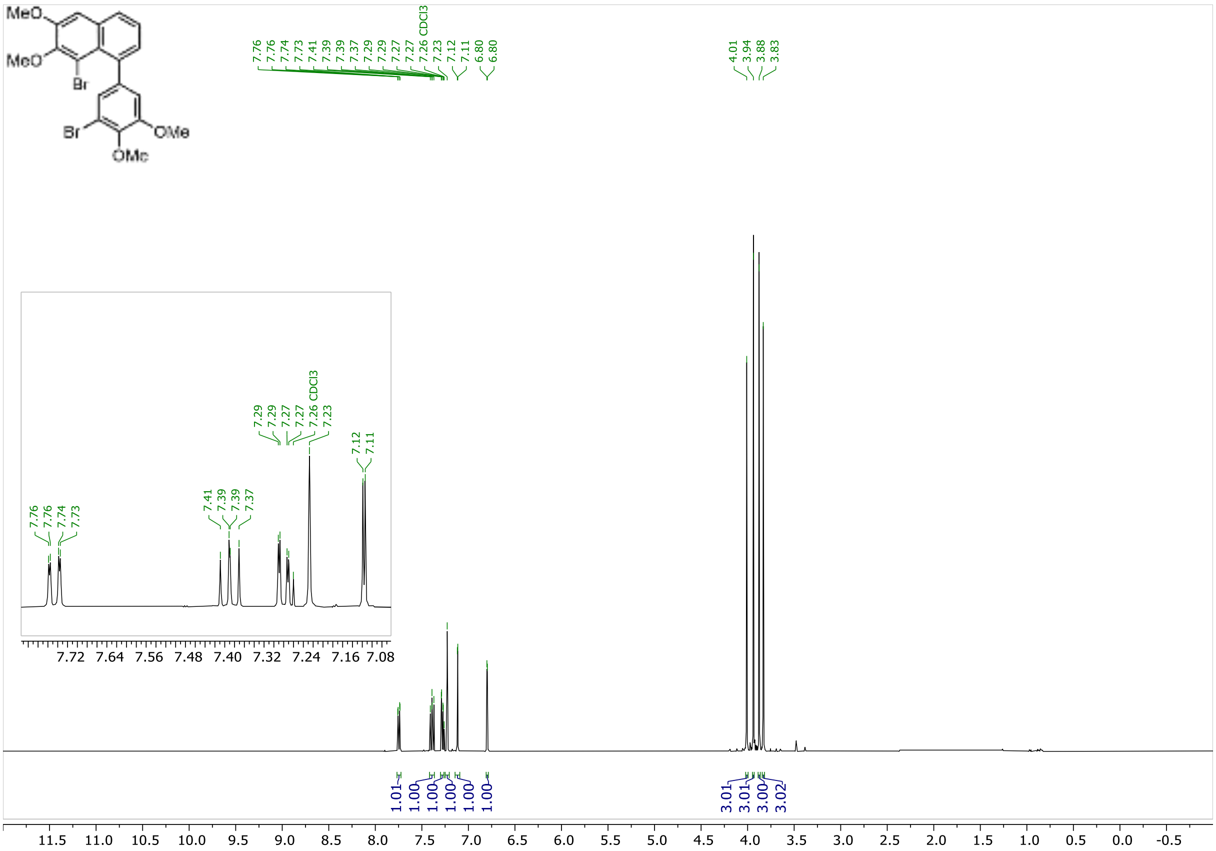

3k, ^{13}C{^{1}H} NMR (101 MHz, CDCl$_3$)

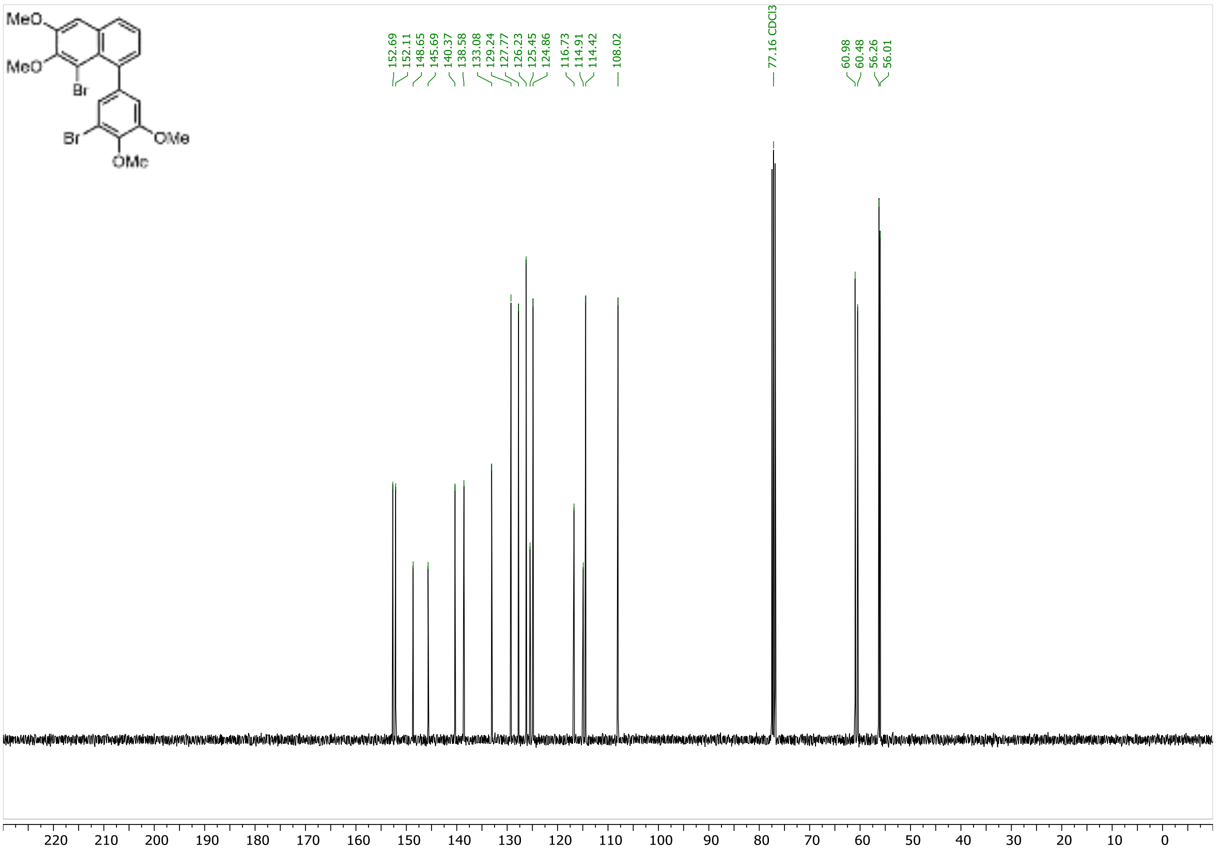

3k, APT $^{13}C\{^{1}H\}$ NMR (101 MHz, $CDCl_3$)

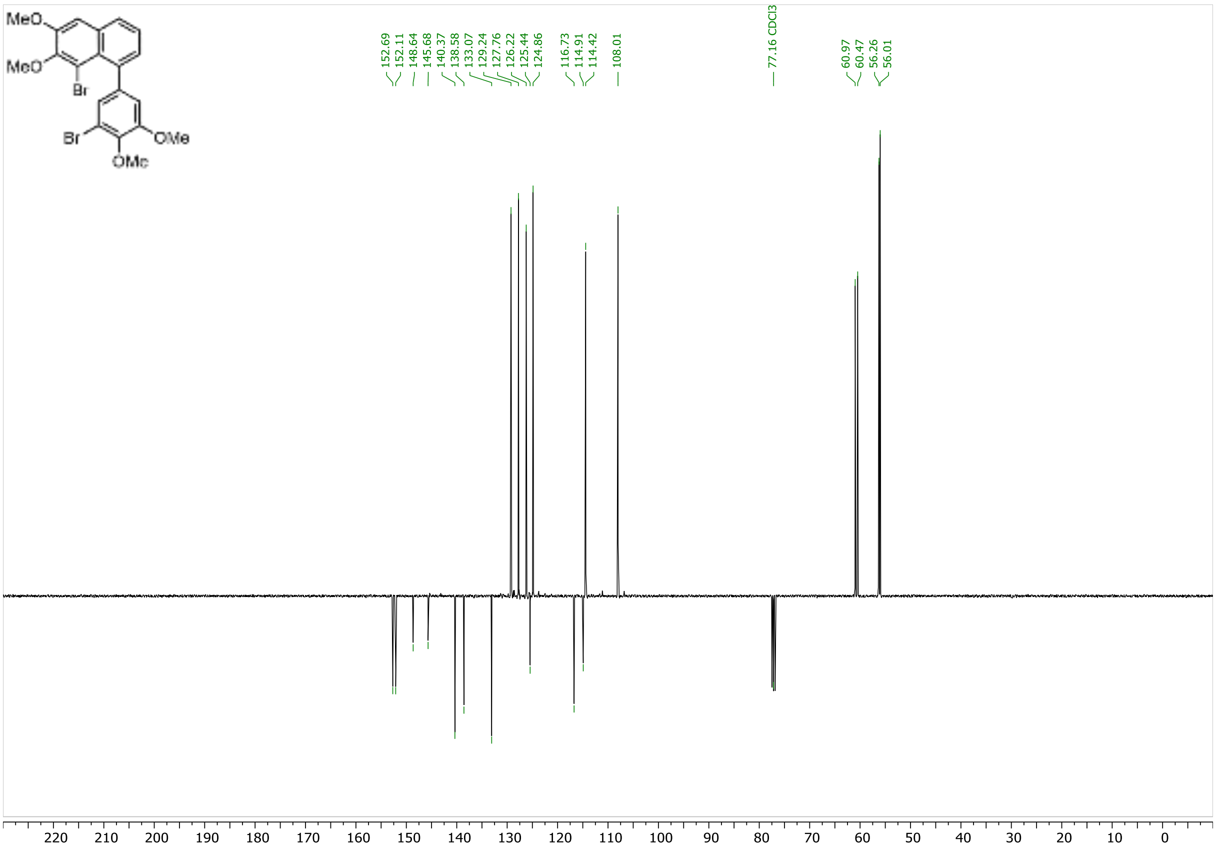

3k, HSQC ($CDCl_3$)

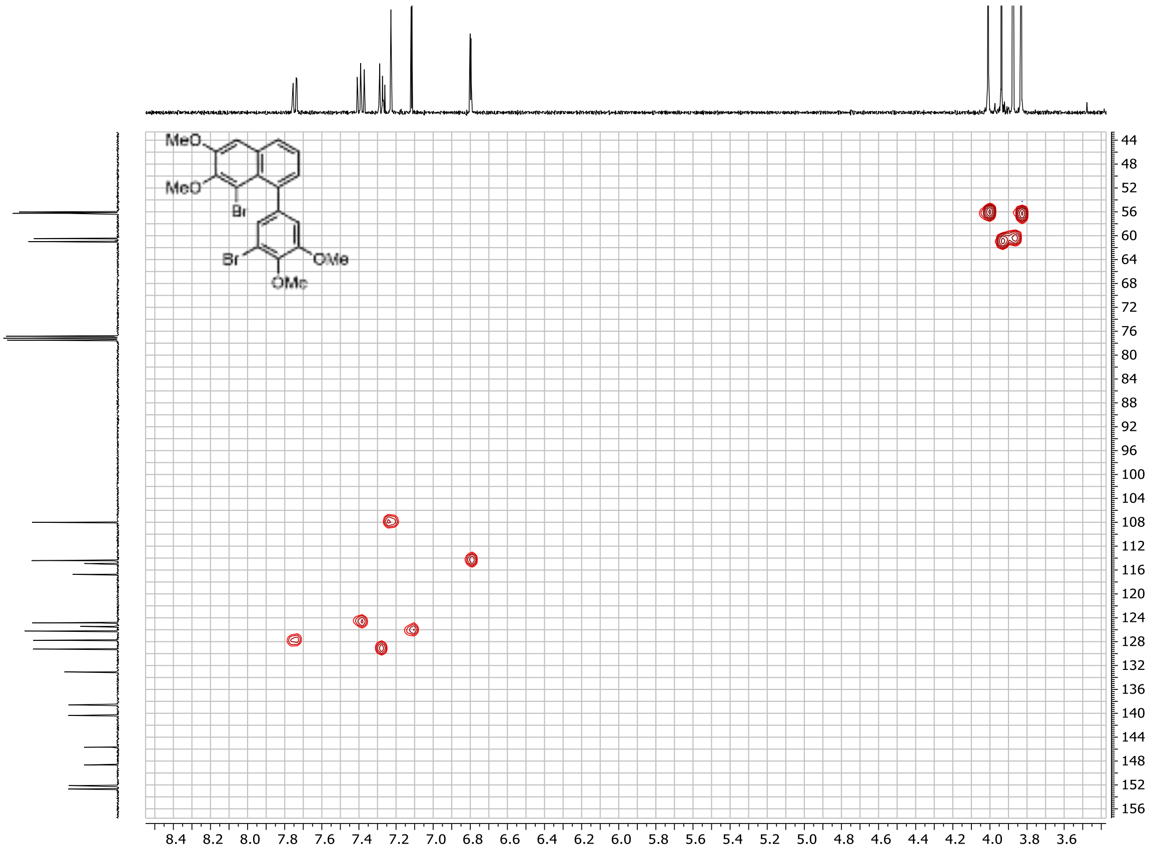

3k, HMBC ($CDCl_3$)

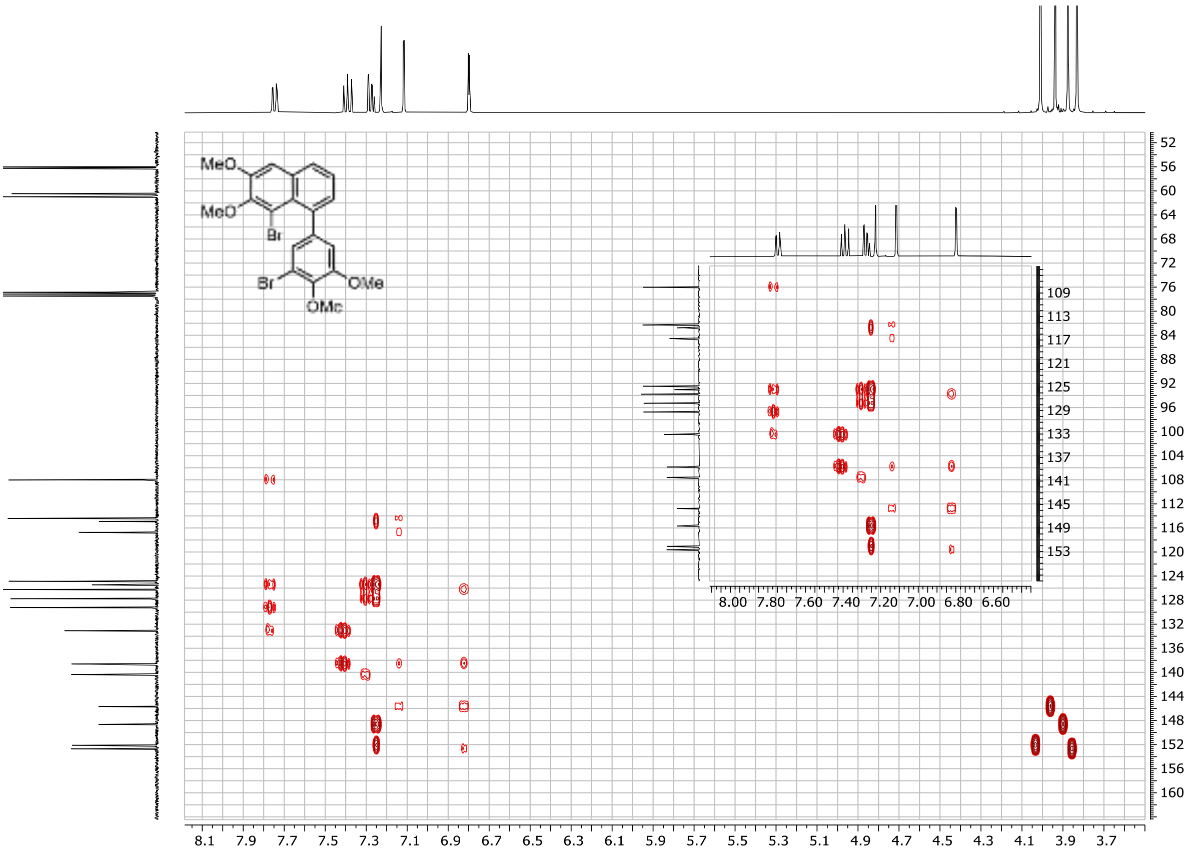

3l, ^{1}H NMR (400 MHz, $CDCl_3$)

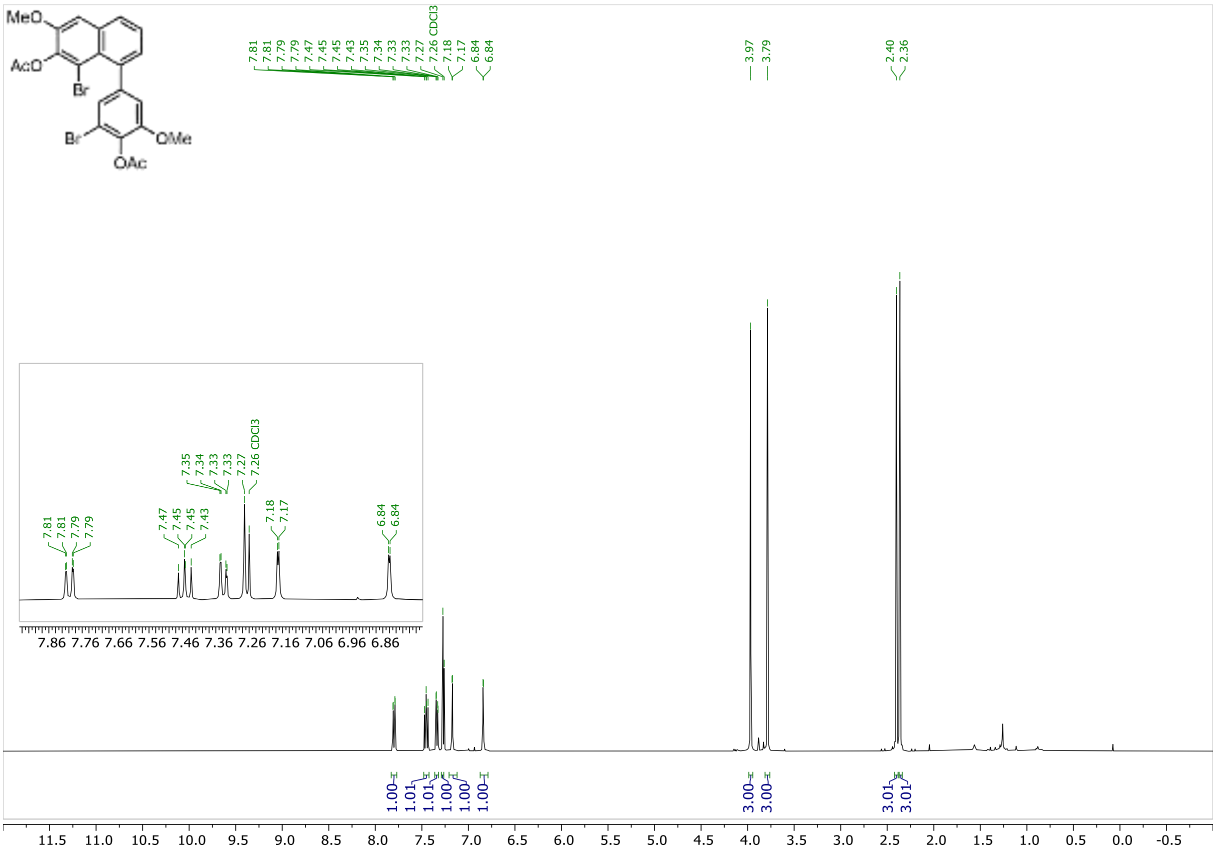

3l, $^{13}C\{^1H\}$ NMR (101 MHz, $CDCl_3$)

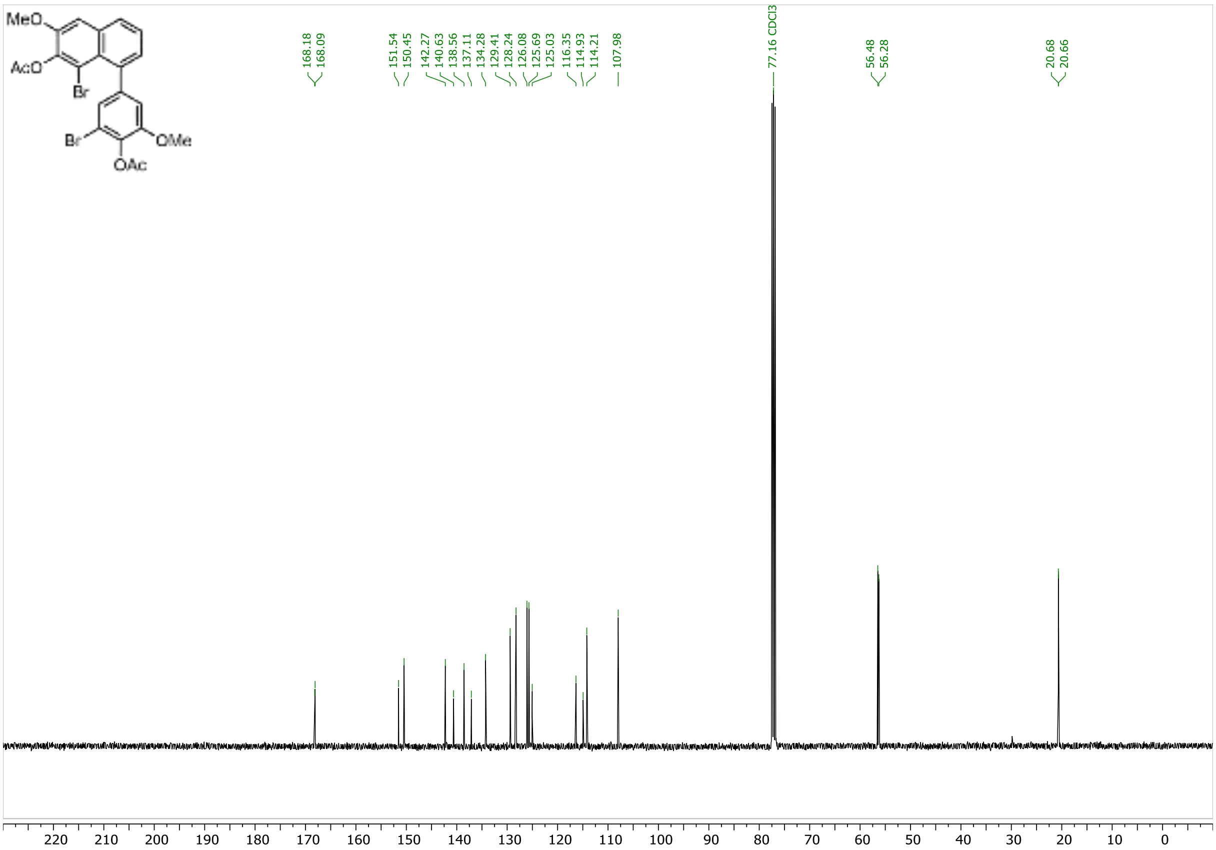

3l, HSQC ($CDCl_3$)

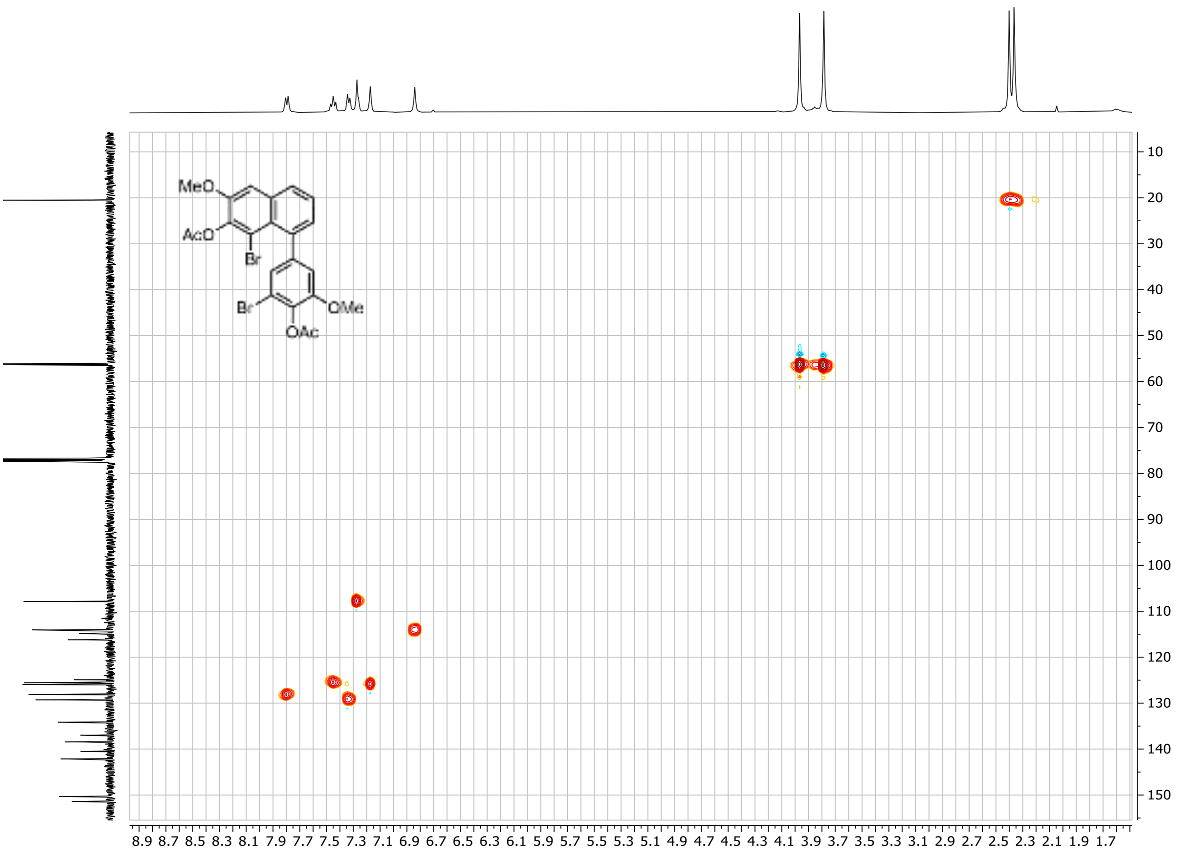

3l, HMBC ($CDCl_3$)

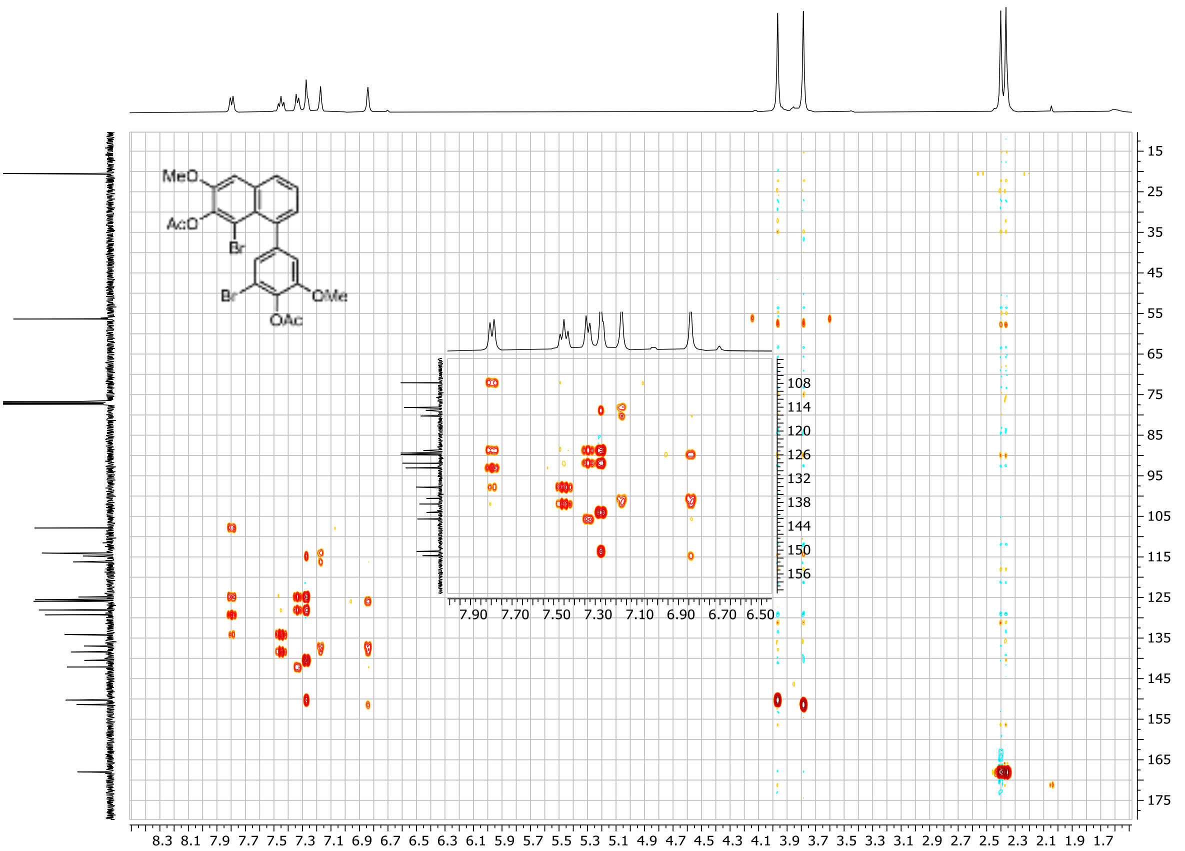

3m, ^{1}H NMR (400 MHz, $CDCl_3$)

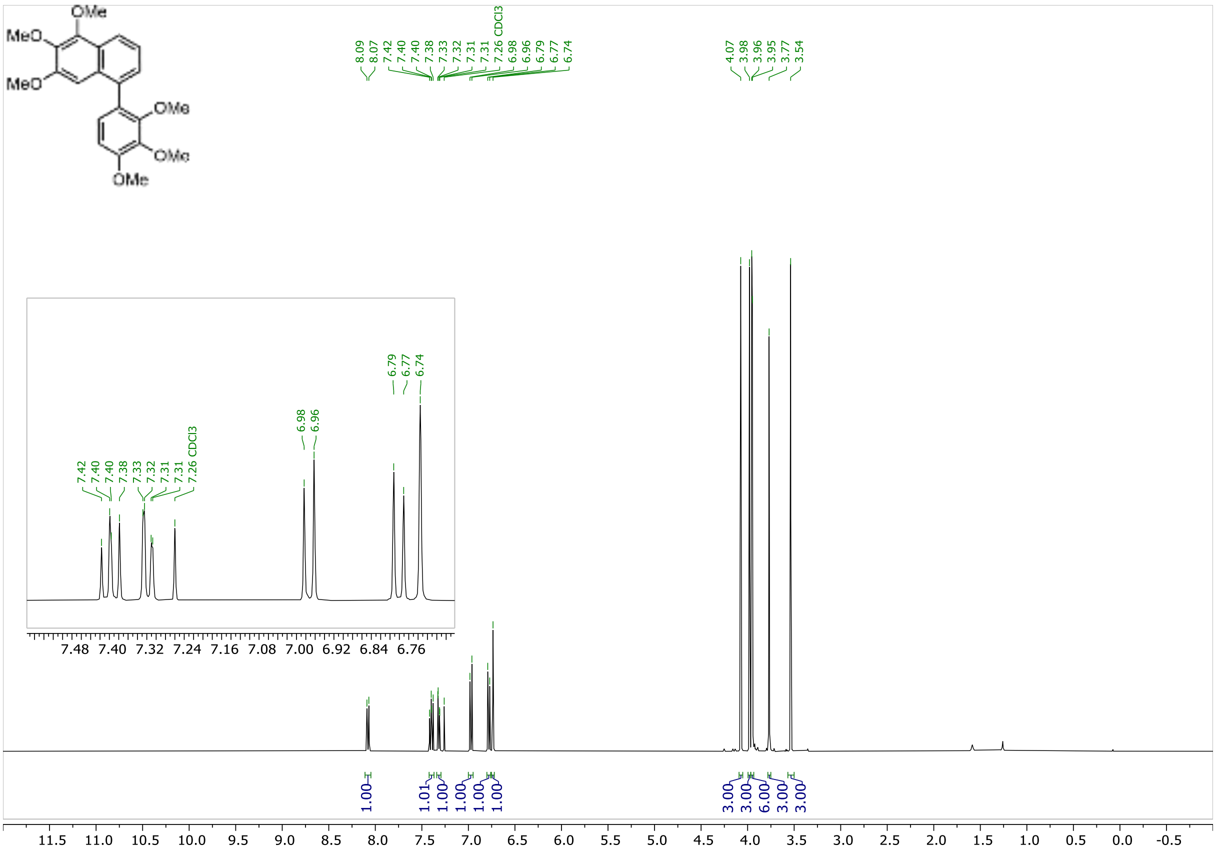

3m, ^{13}C{^{1}H} NMR (101 MHz, $CDCl_3$)

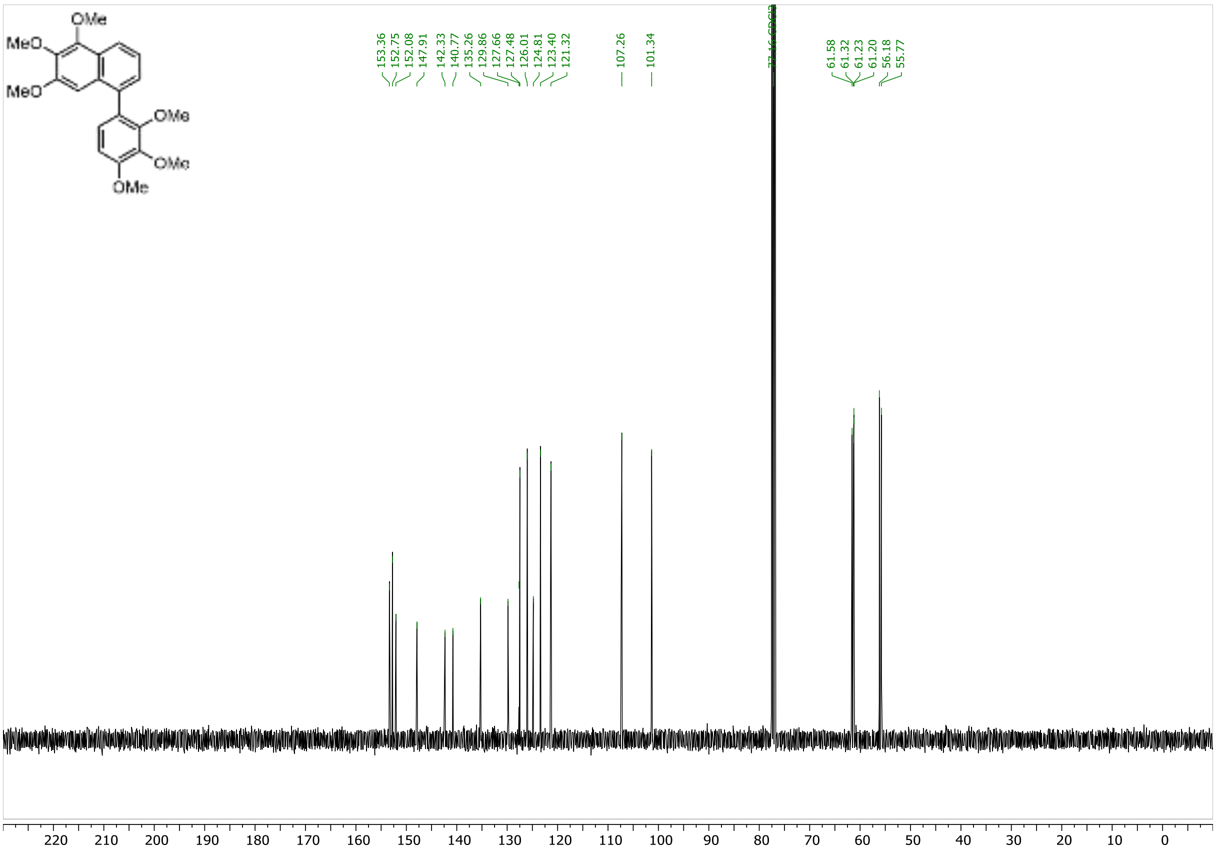

3n, ^{1}H NMR (400 MHz, CDCl$_3$)

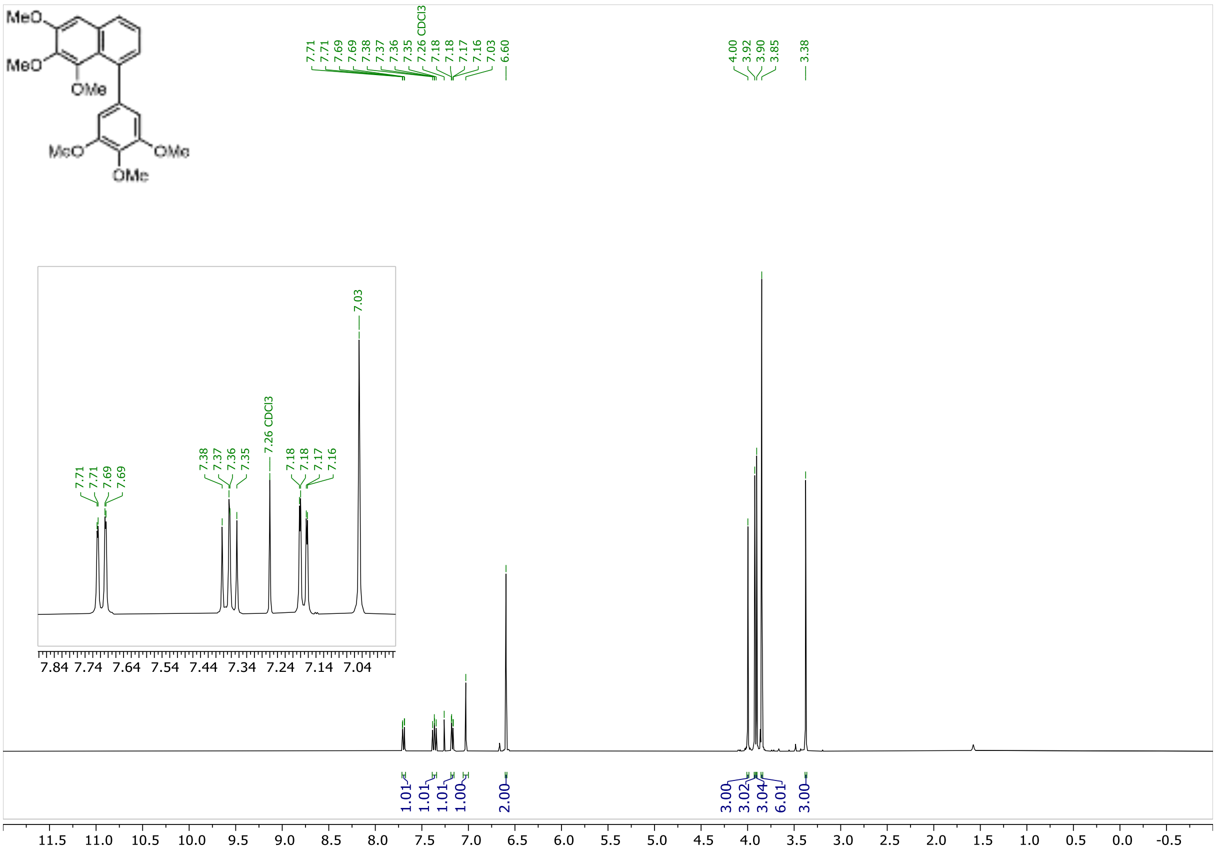

3n, ^{13}C{^{1}H} NMR (101 MHz, CDCl$_3$)

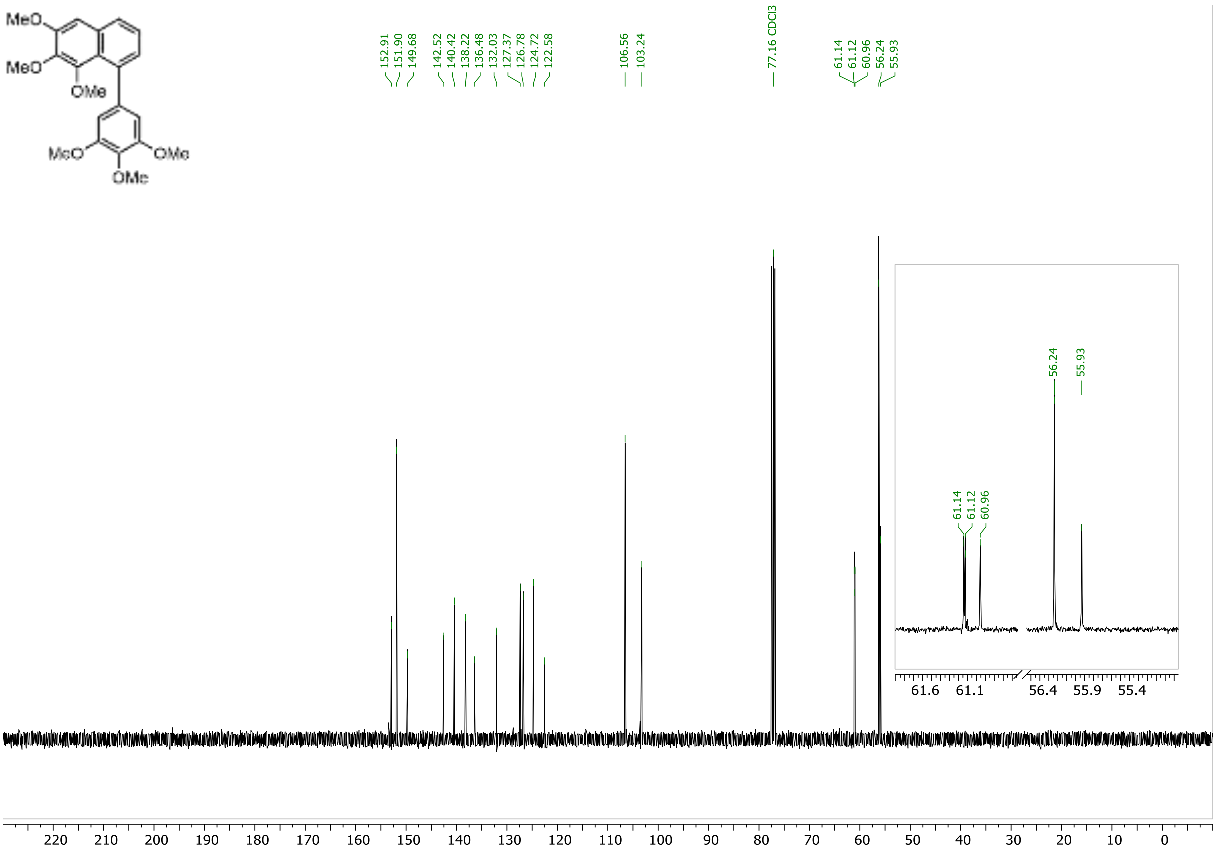

3o, ^{1}H NMR (400 MHz, $CDCl_3$)

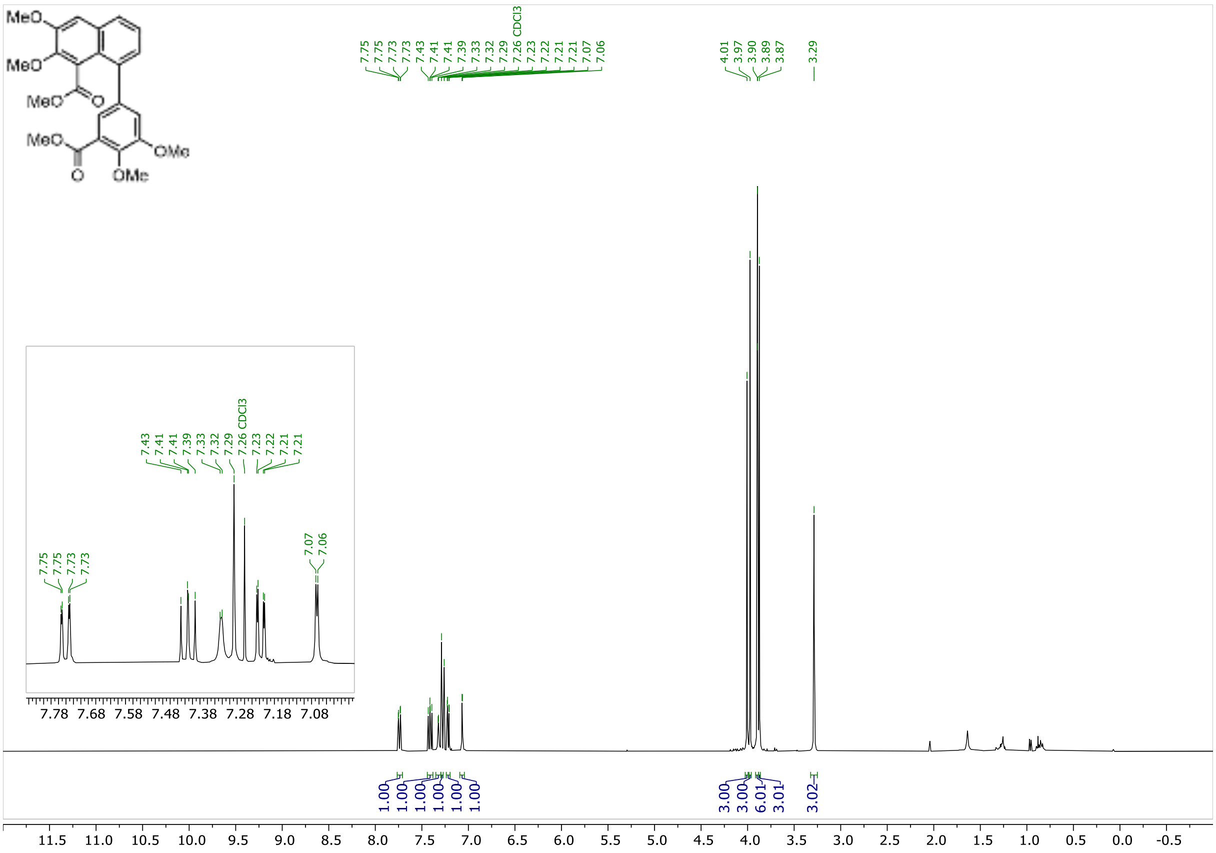

3o, ^{13}C{^{1}H} NMR (101 MHz, $CDCl_3$)

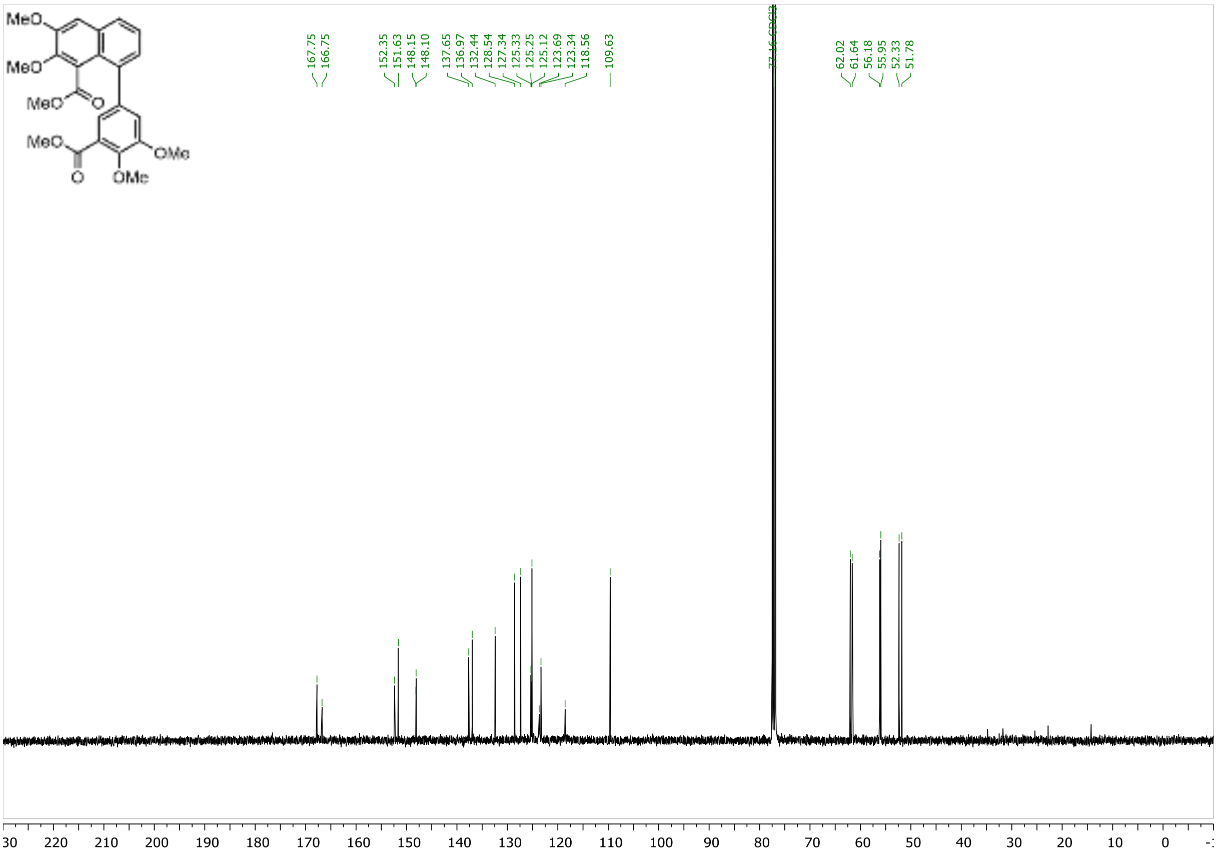

3o, DEPT $^{13}C\{^{1}H\}$ NMR (101 MHz, $CDCl_3$)

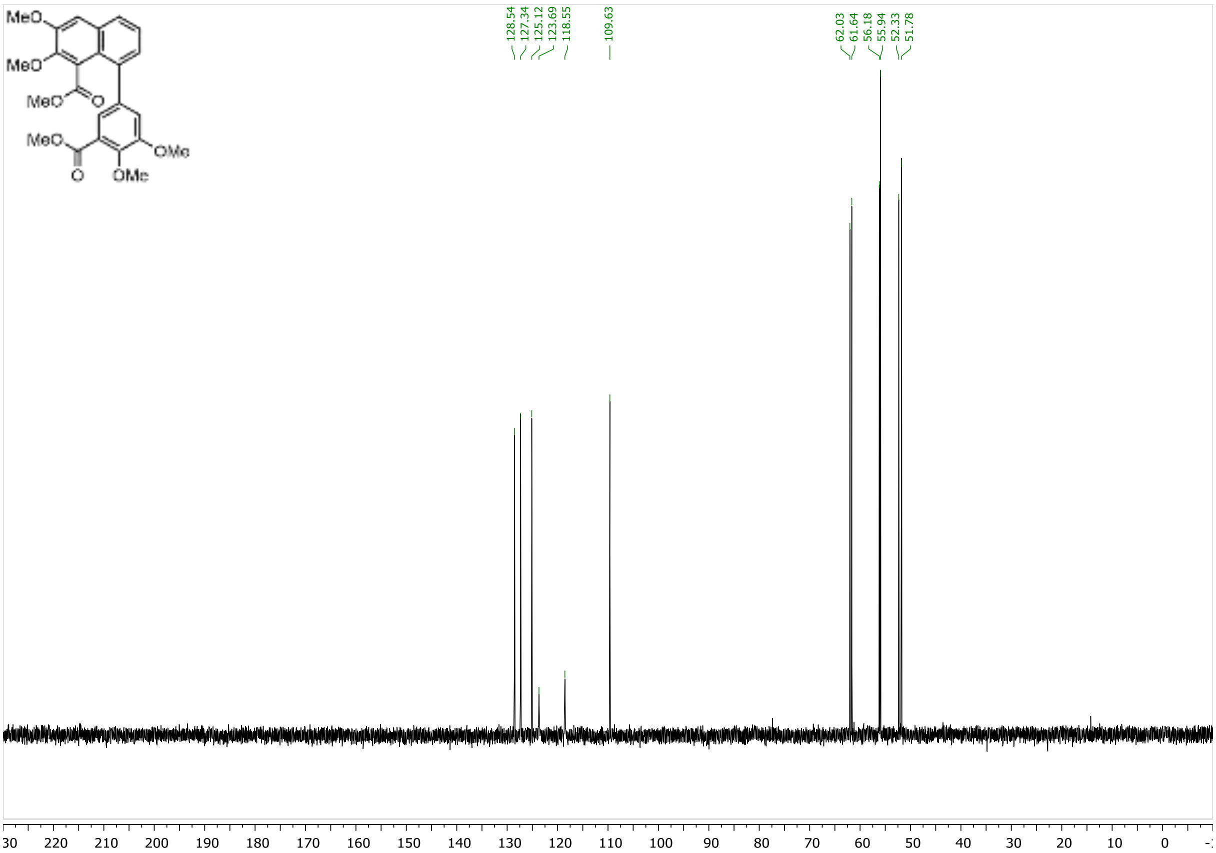

3o, HSQC ($CDCl_3$)

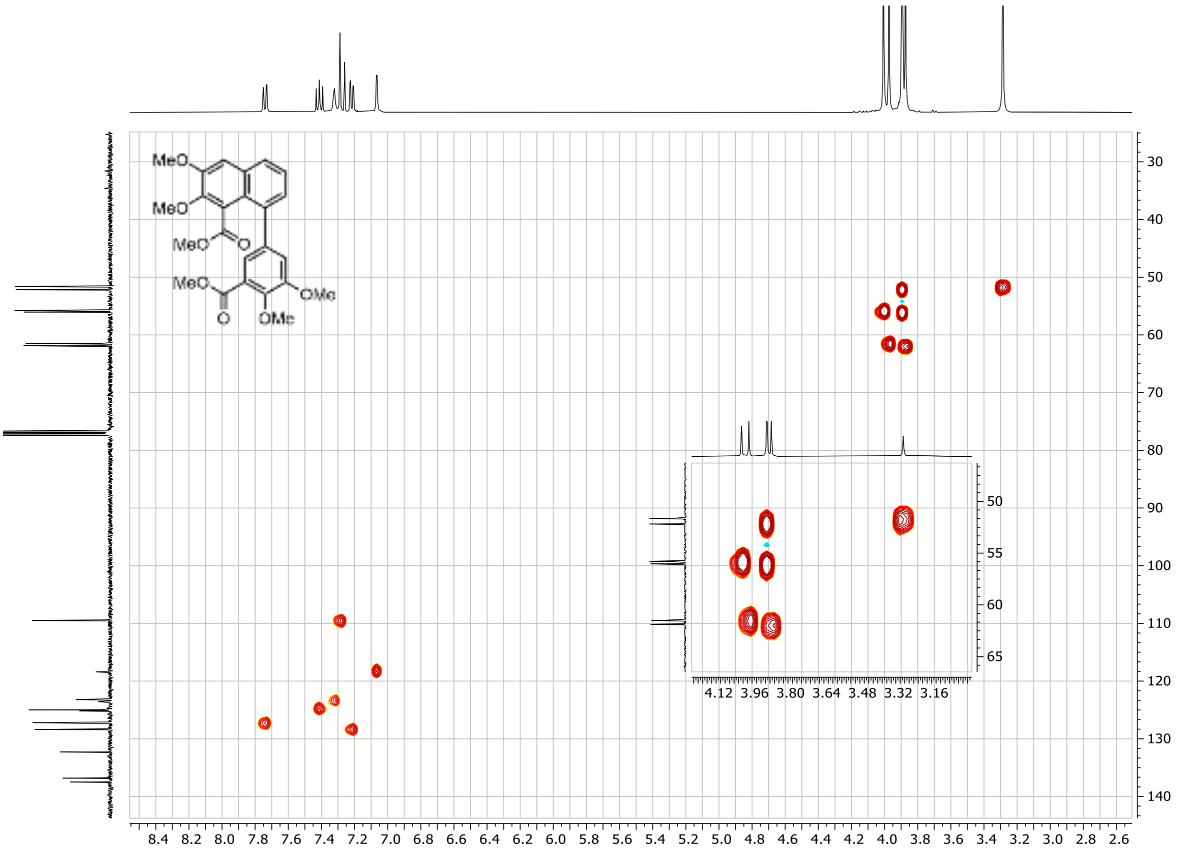

3o, HMBC ($CDCl_3$)

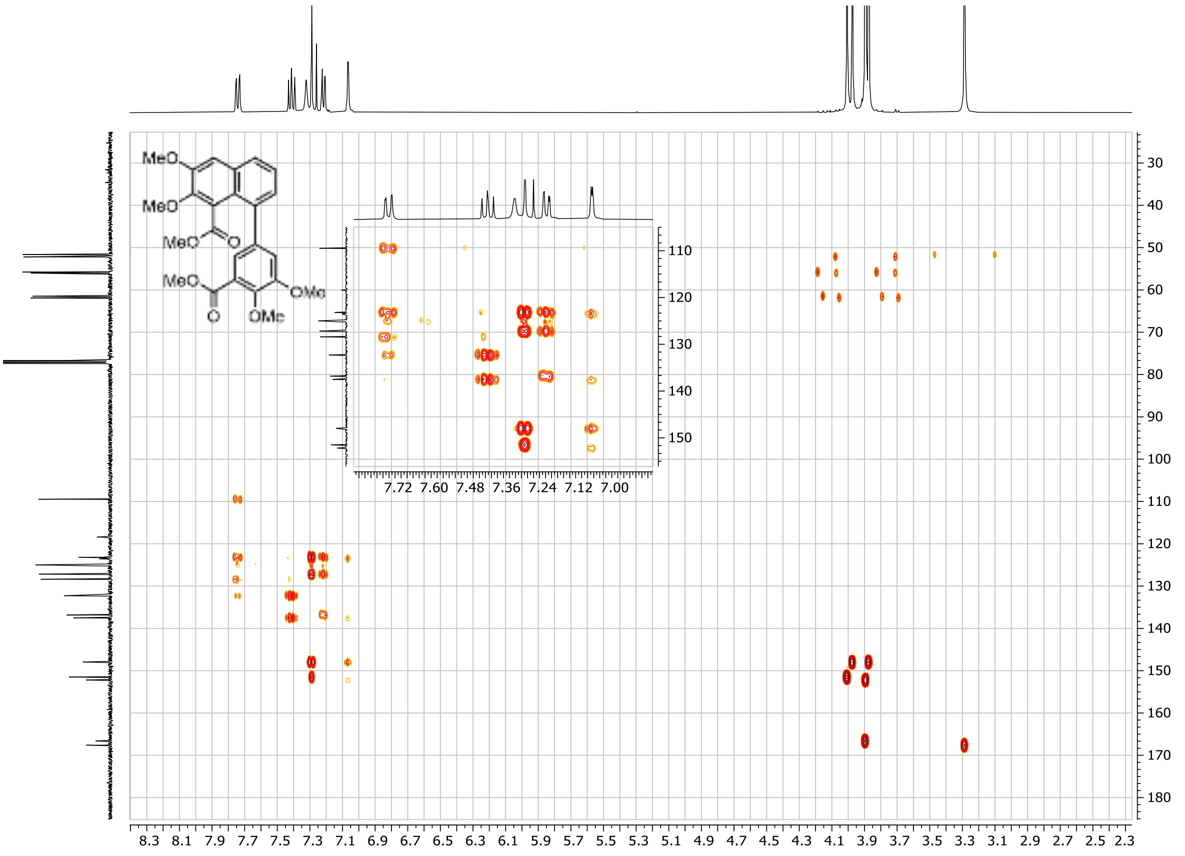

3p, ^{1}H NMR (400 MHz, CDCl$_3$)

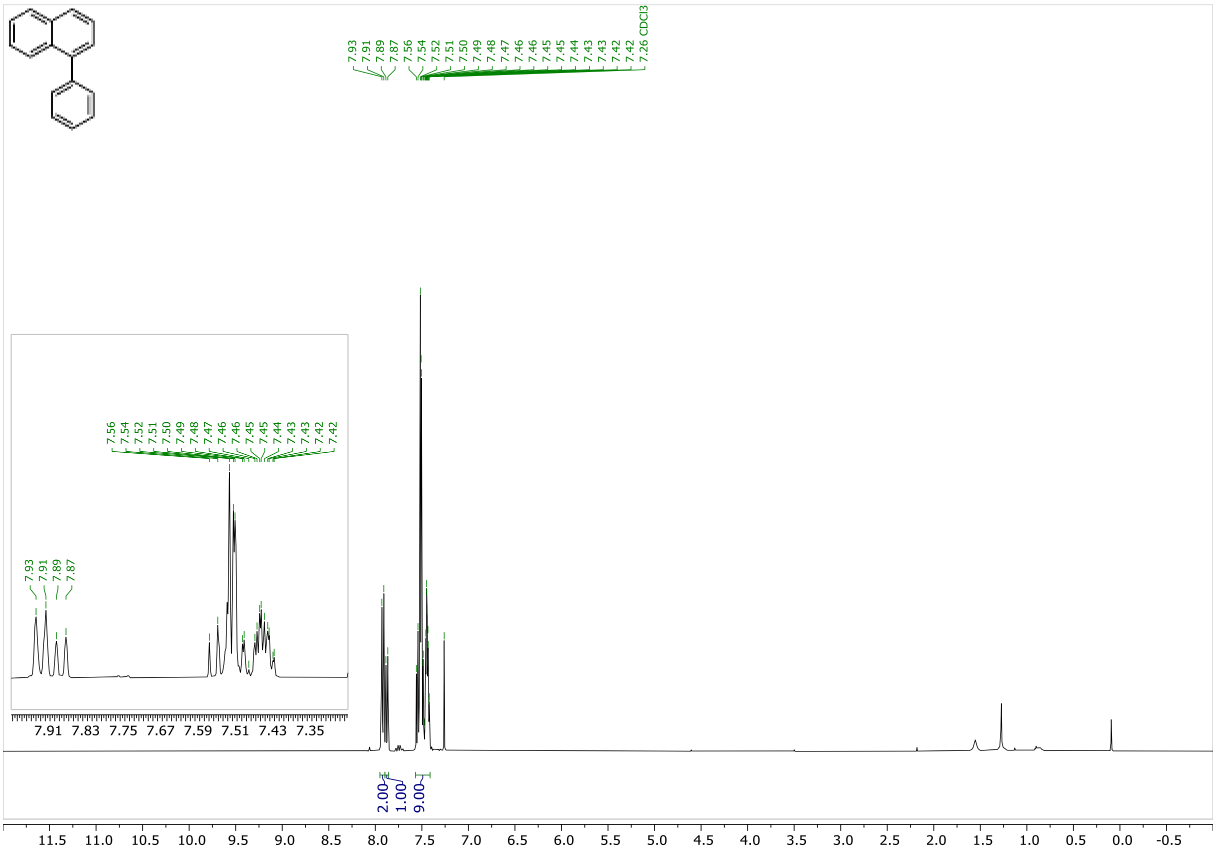

3p, ^{13}C{^{1}H} NMR (101 MHz, CDCl$_3$)

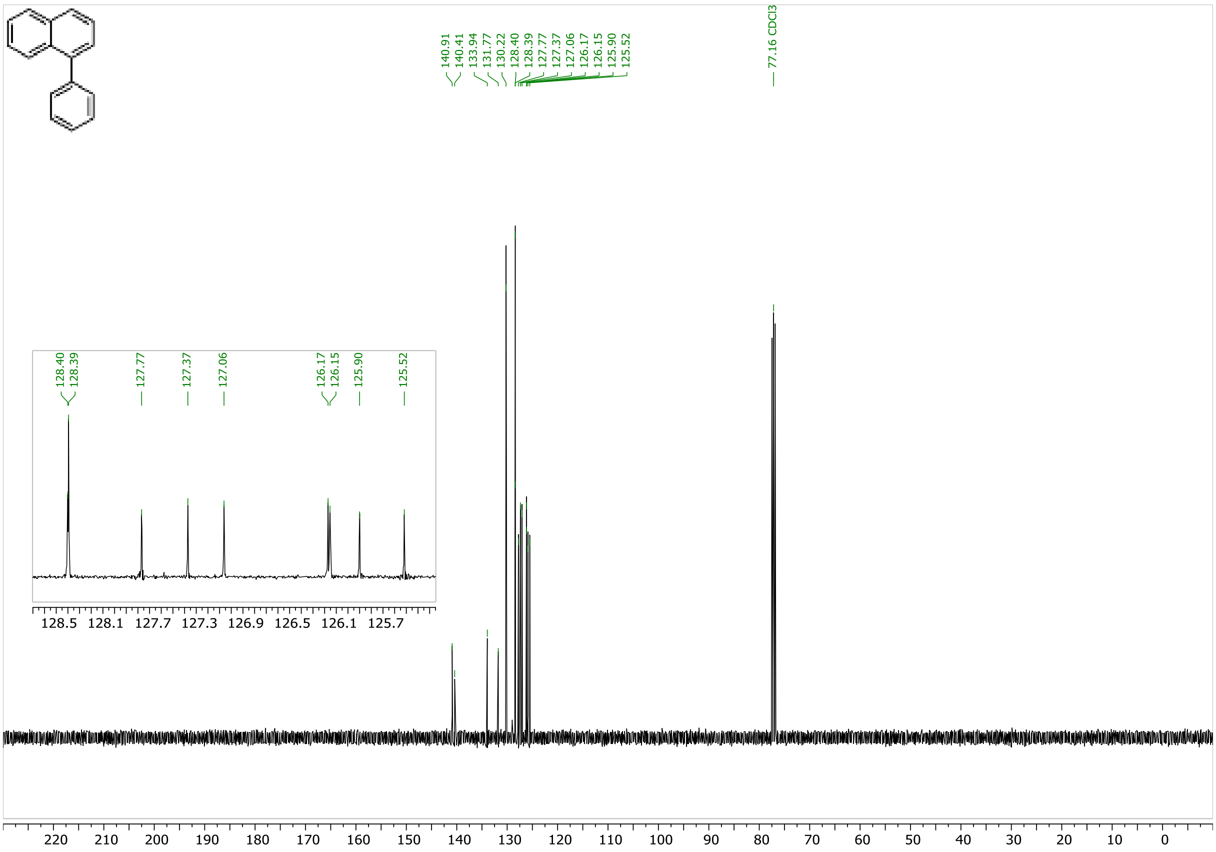

3s, ^{1}H NMR (400 MHz, $CDCl_3$)

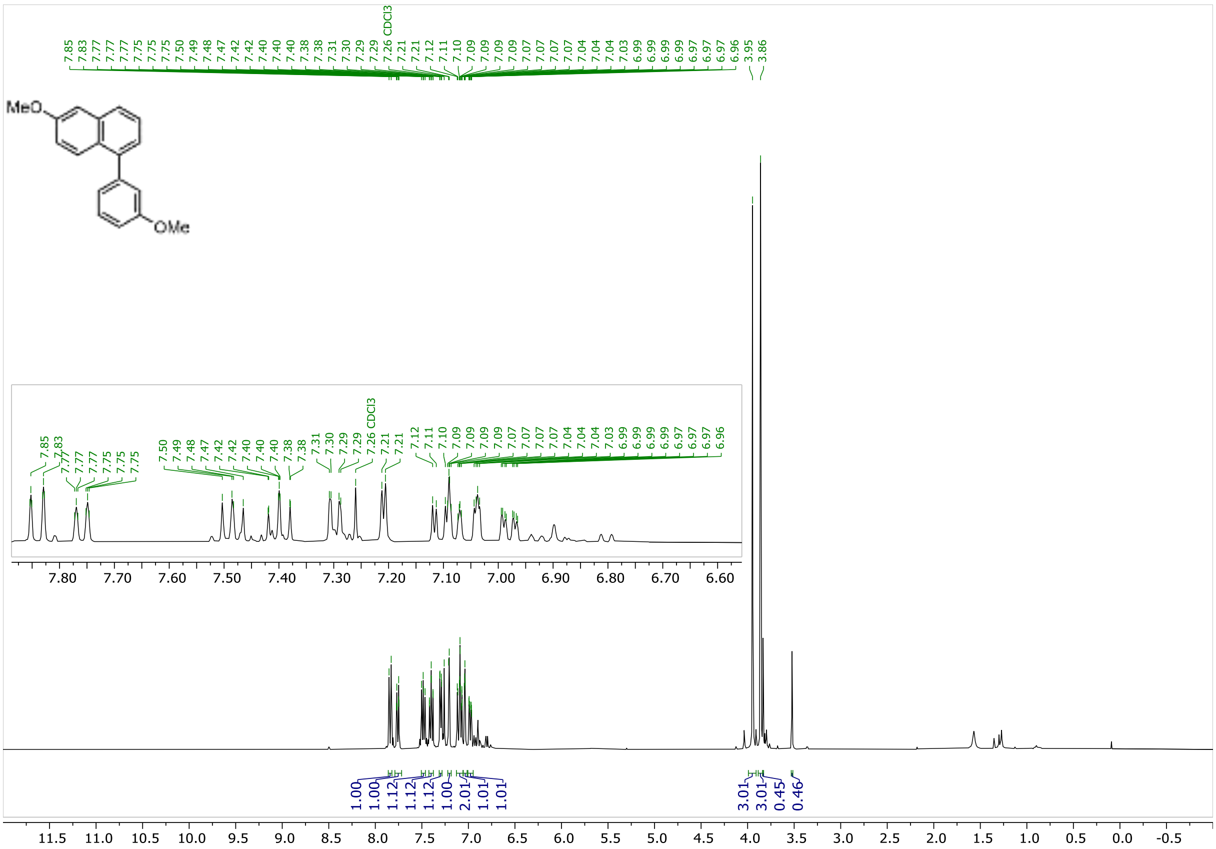

3s, ^{13}C{^{1}H} NMR (101 MHz, $CDCl_3$)

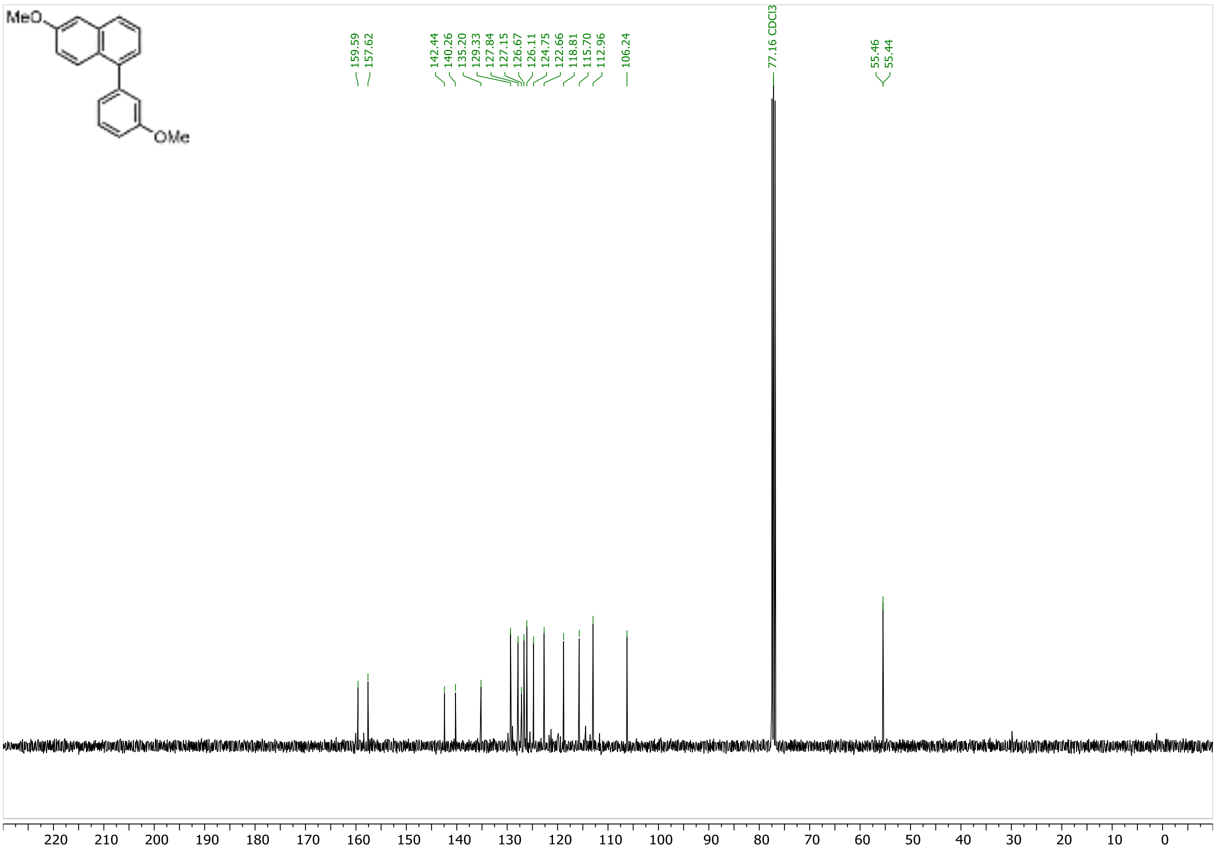

3s, APT $^{13}C\{^{1}H\}$ NMR (101 MHz, $CDCl_3$)

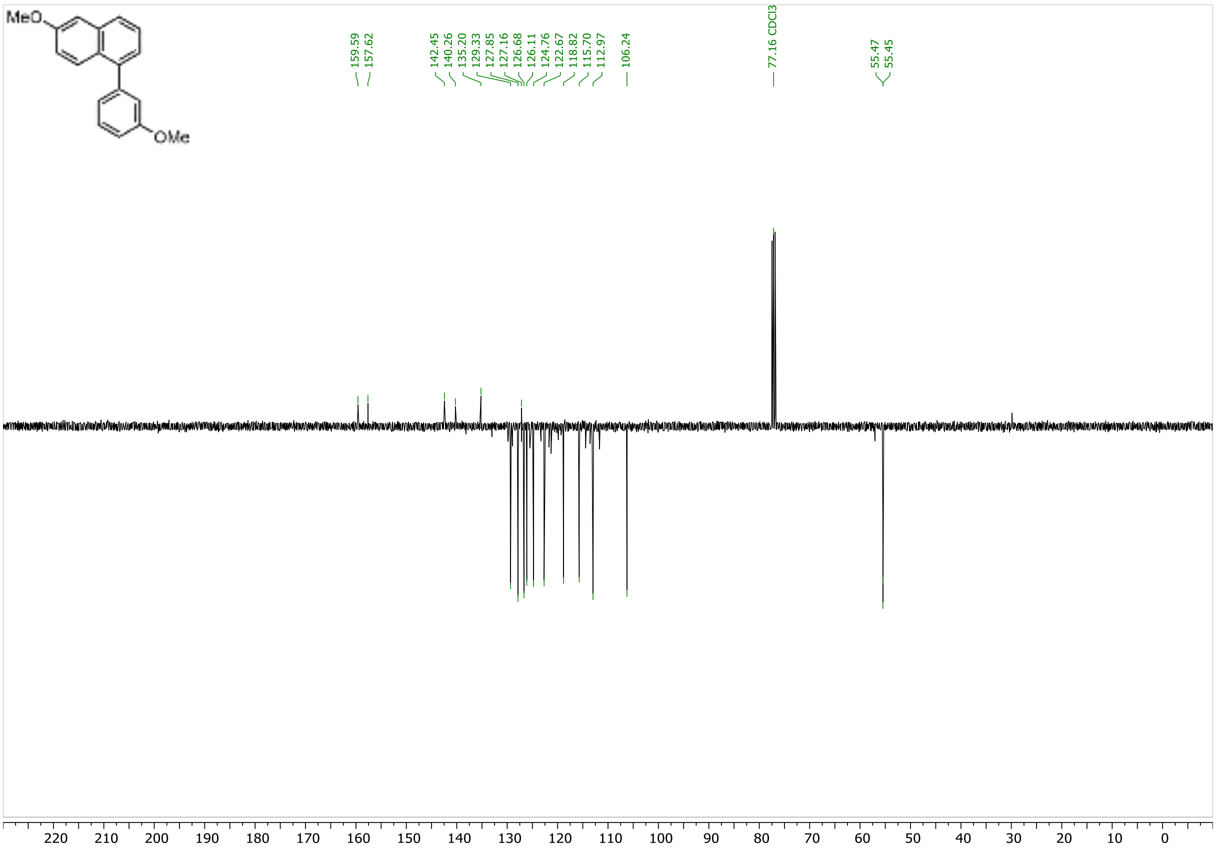

3s, COSY ^{1}H–^{1}H NMR (400 MHz, $CDCl_3$)

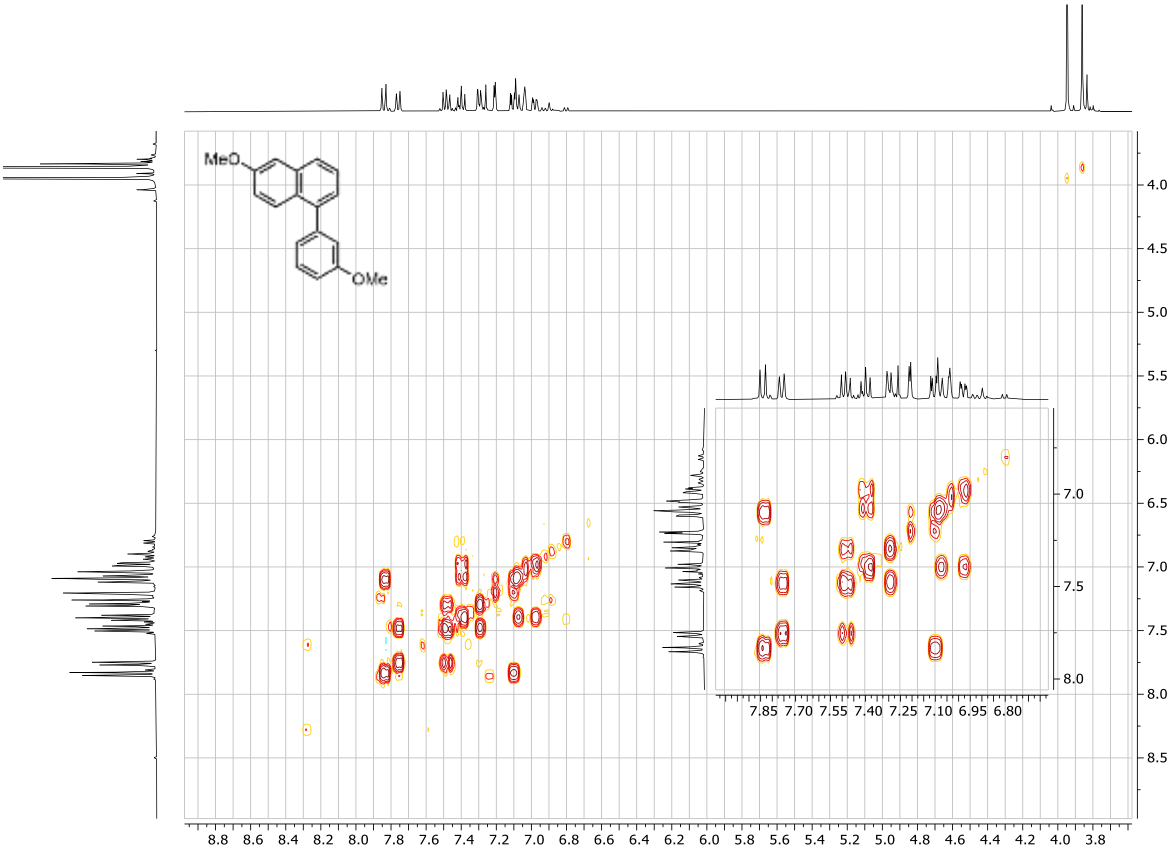

3s, HSQC ($CDCl_3$)

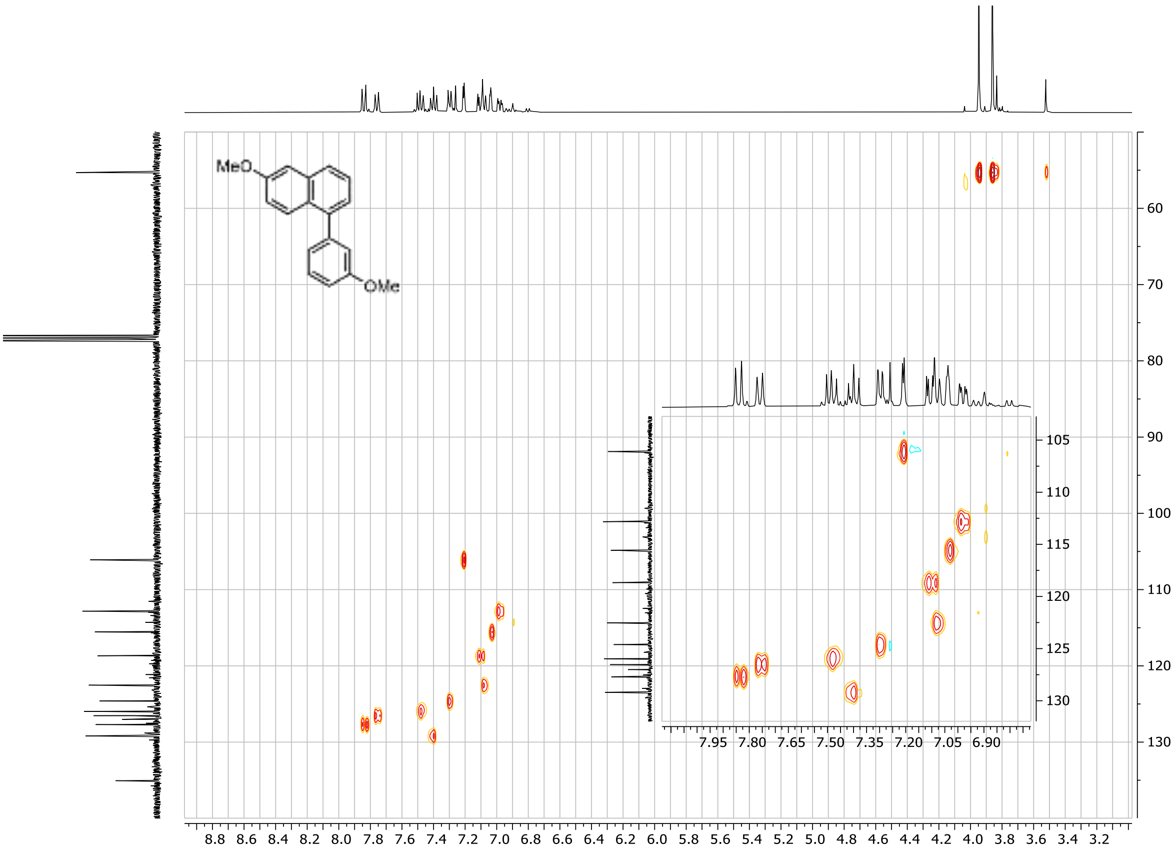

3s, HMBC ($CDCl_3$)

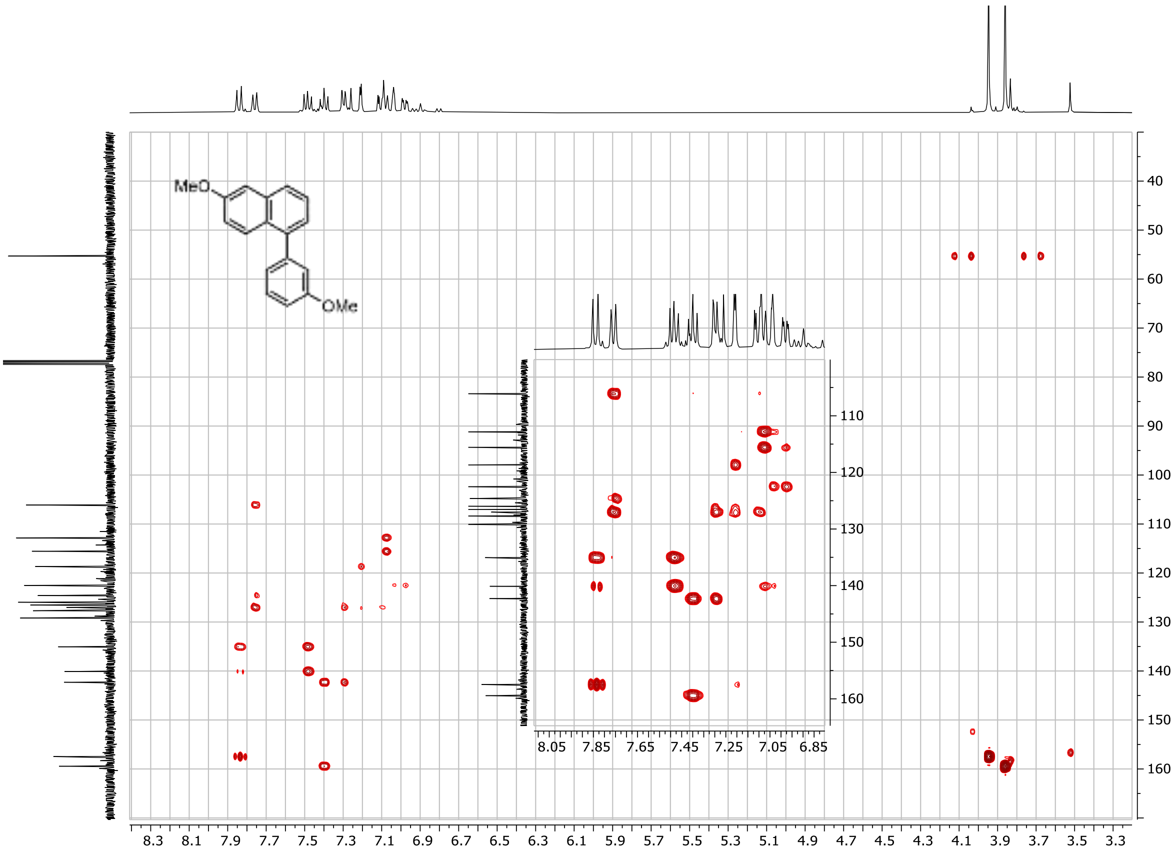

3rs, ^{1}H NMR (400 MHz, $CDCl_3$)

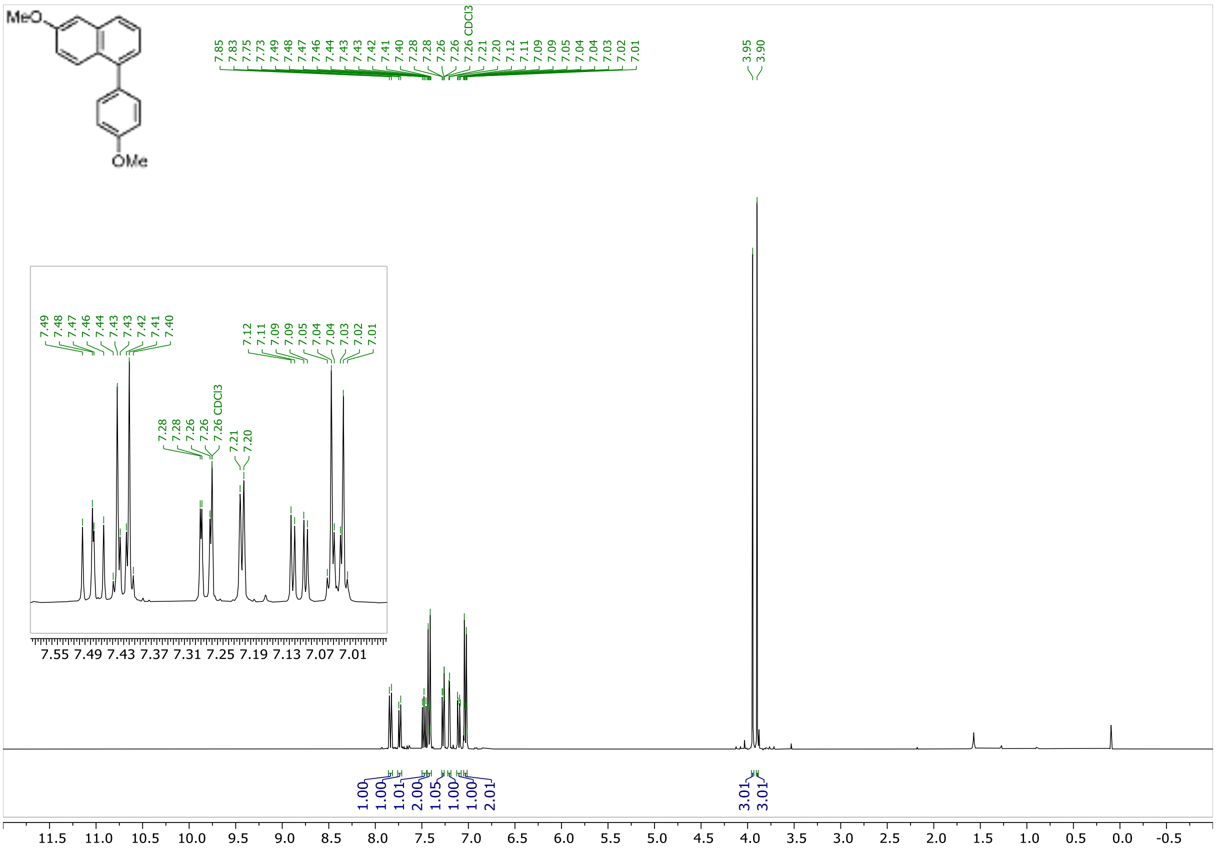

3rs, $^{13}C\{^{1}H\}$ NMR (101 MHz, $CDCl_3$)

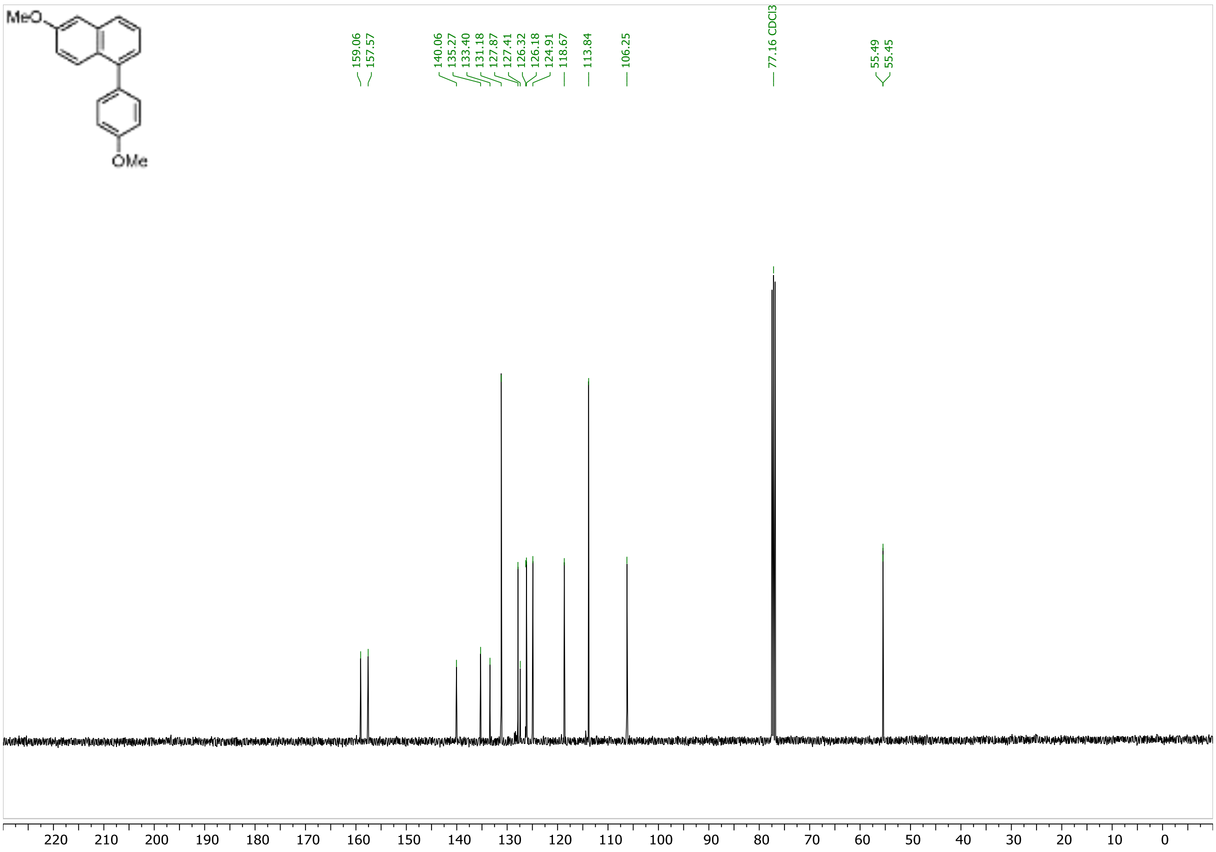

3ar, ^{1}H NMR (400 MHz, $CDCl_3$)

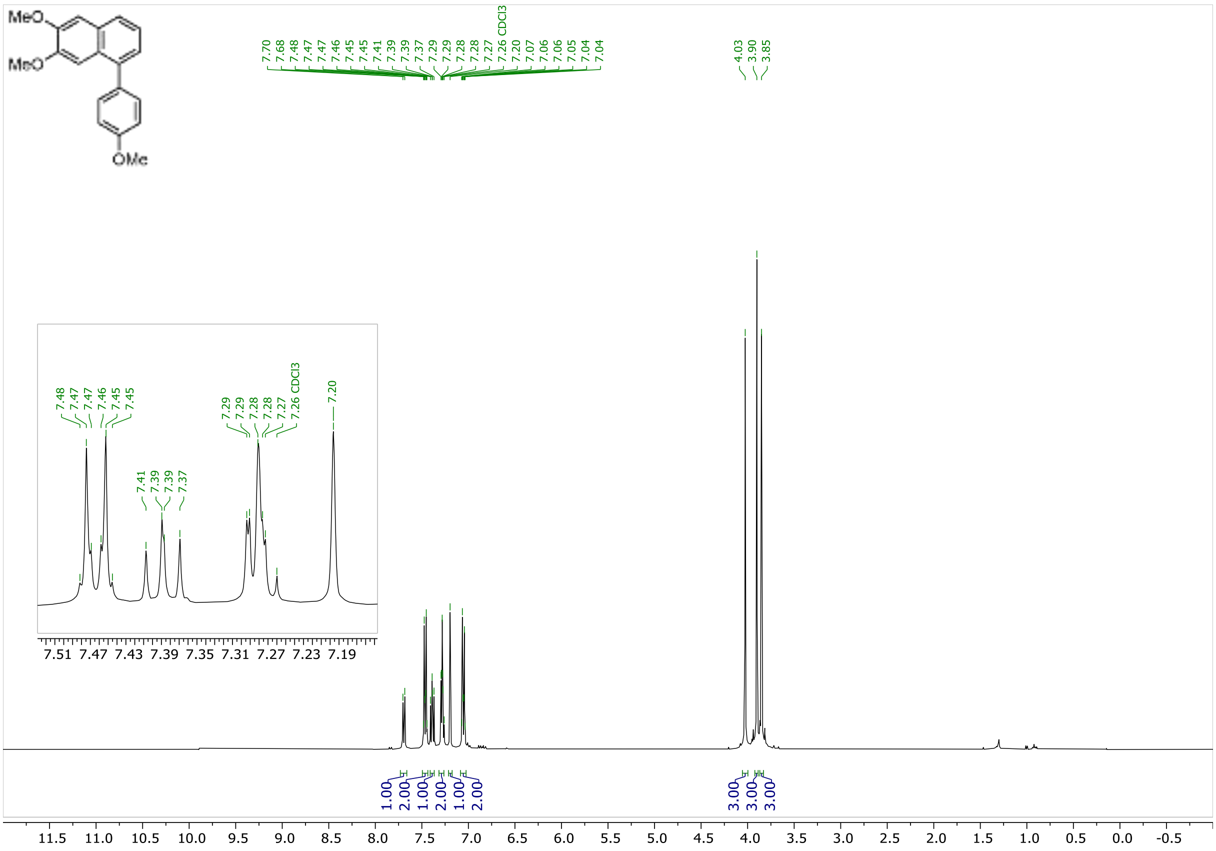

3ar, ^{13}C{^{1}H} NMR (101 MHz, $CDCl_3$)

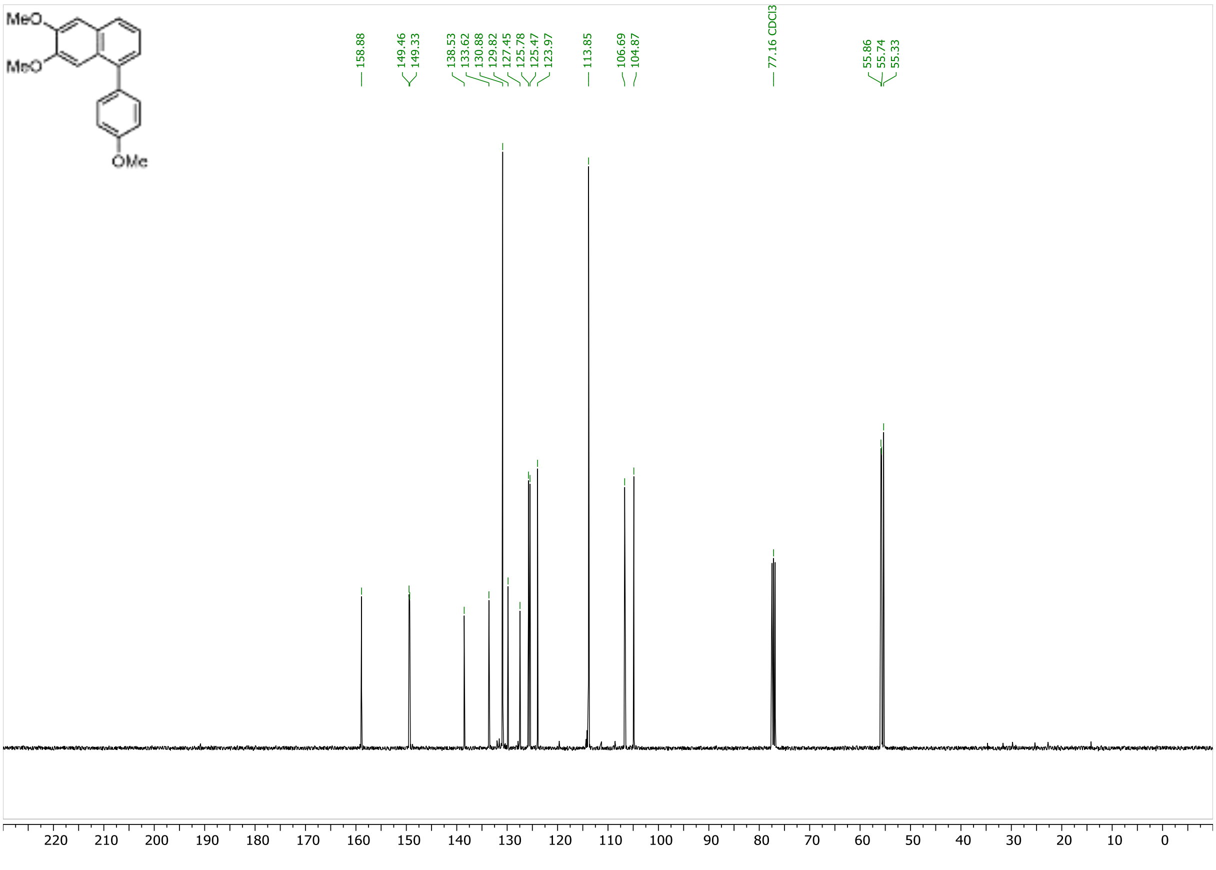

4a, ^{1}H NMR (400 MHz, $CDCl_3$)

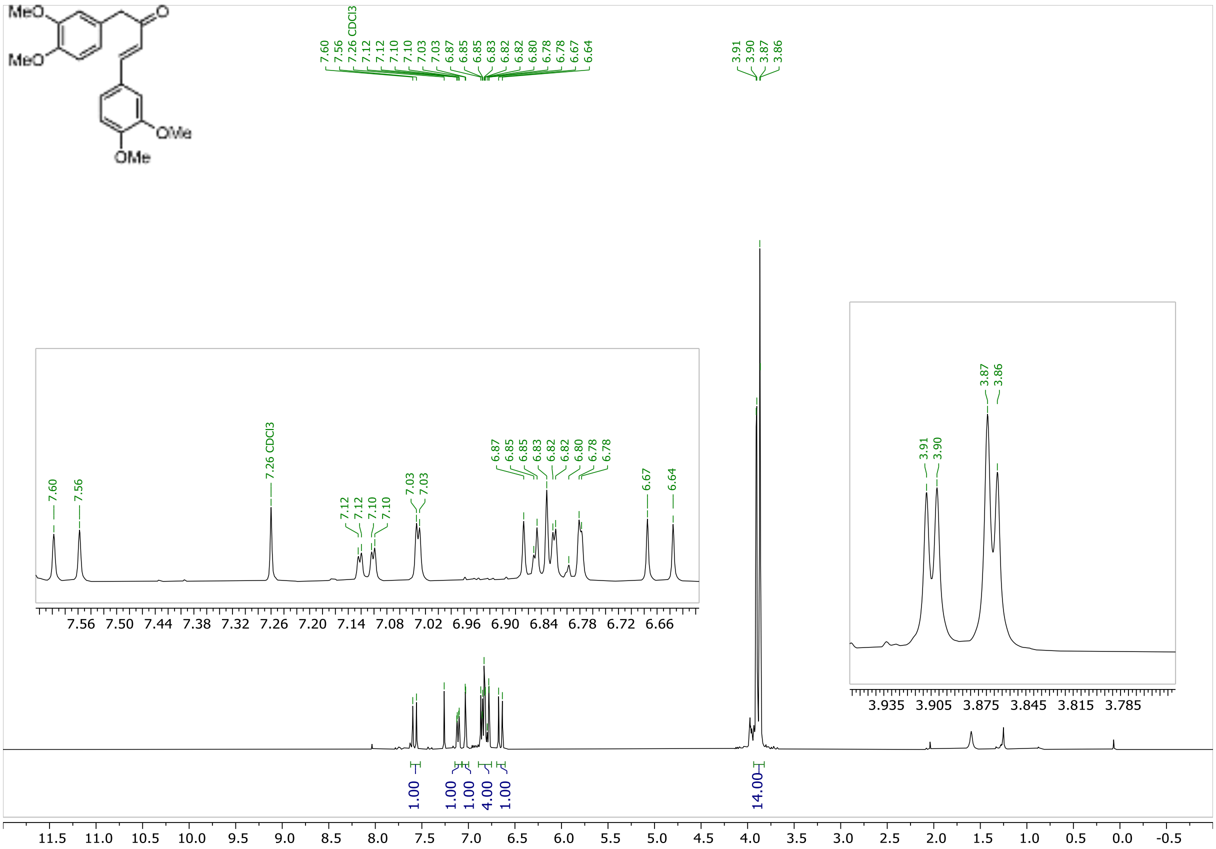

4a, $^{13}C\{^{1}H\}$ NMR (101 MHz, $CDCl_3$)

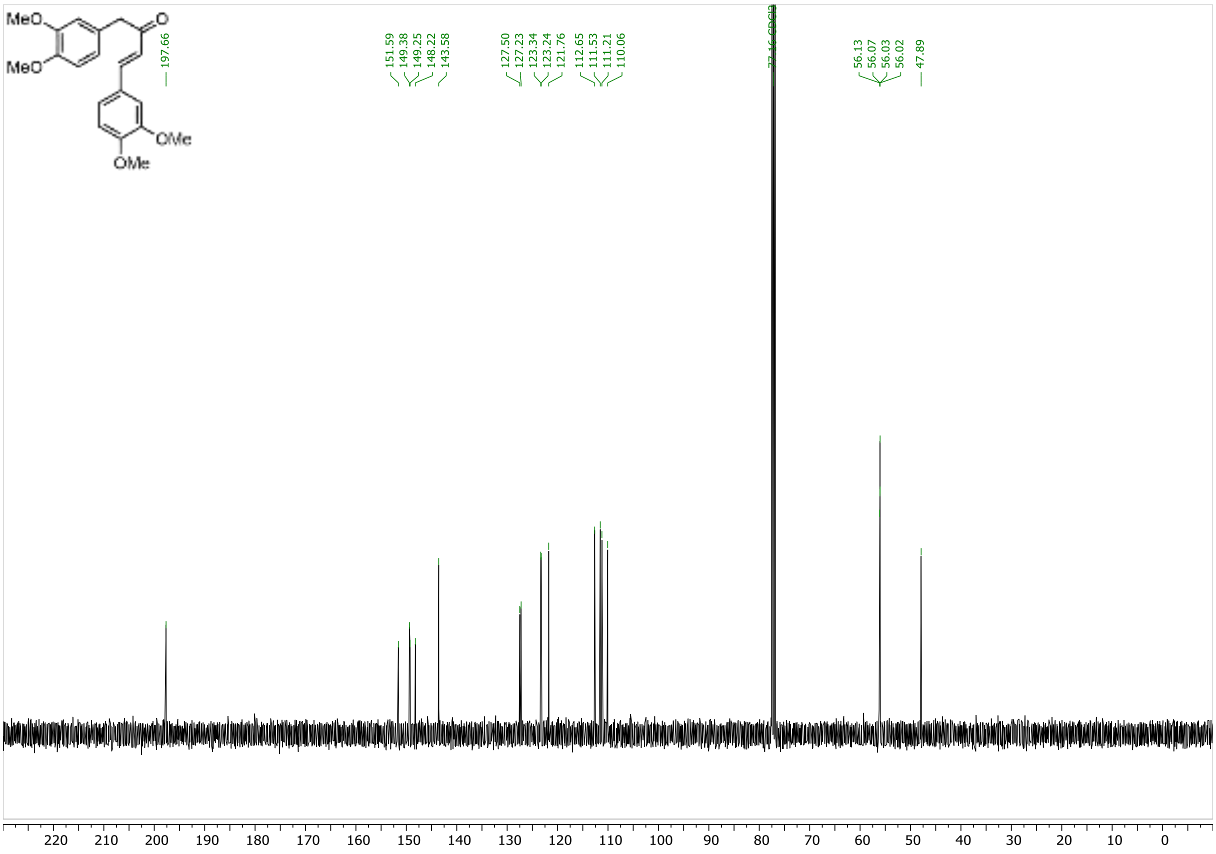

4h, ^{1}H NMR (400 MHz, $CDCl_3$)

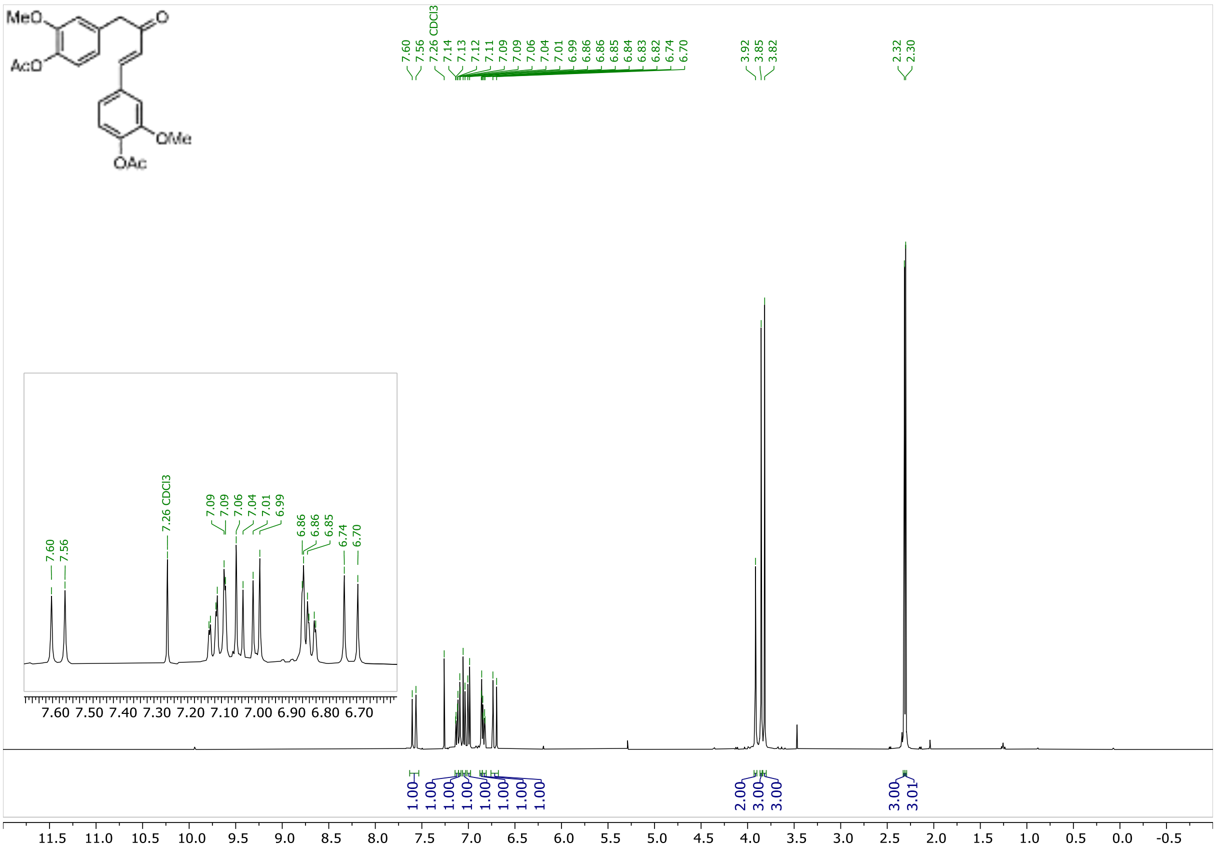

4h, ^{13}C{^{1}H} NMR (101 MHz, $CDCl_3$)

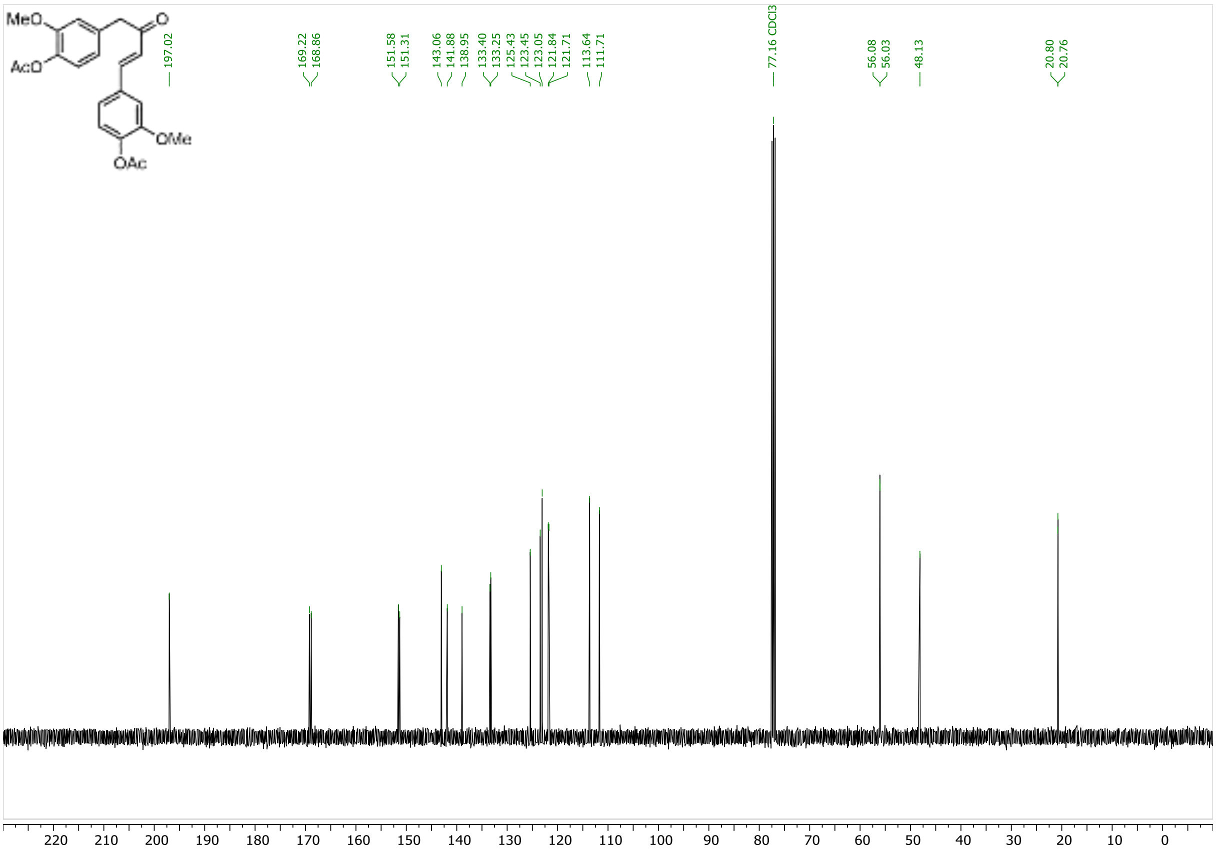

4p, ^{1}H NMR (400 MHz, $CDCl_3$)

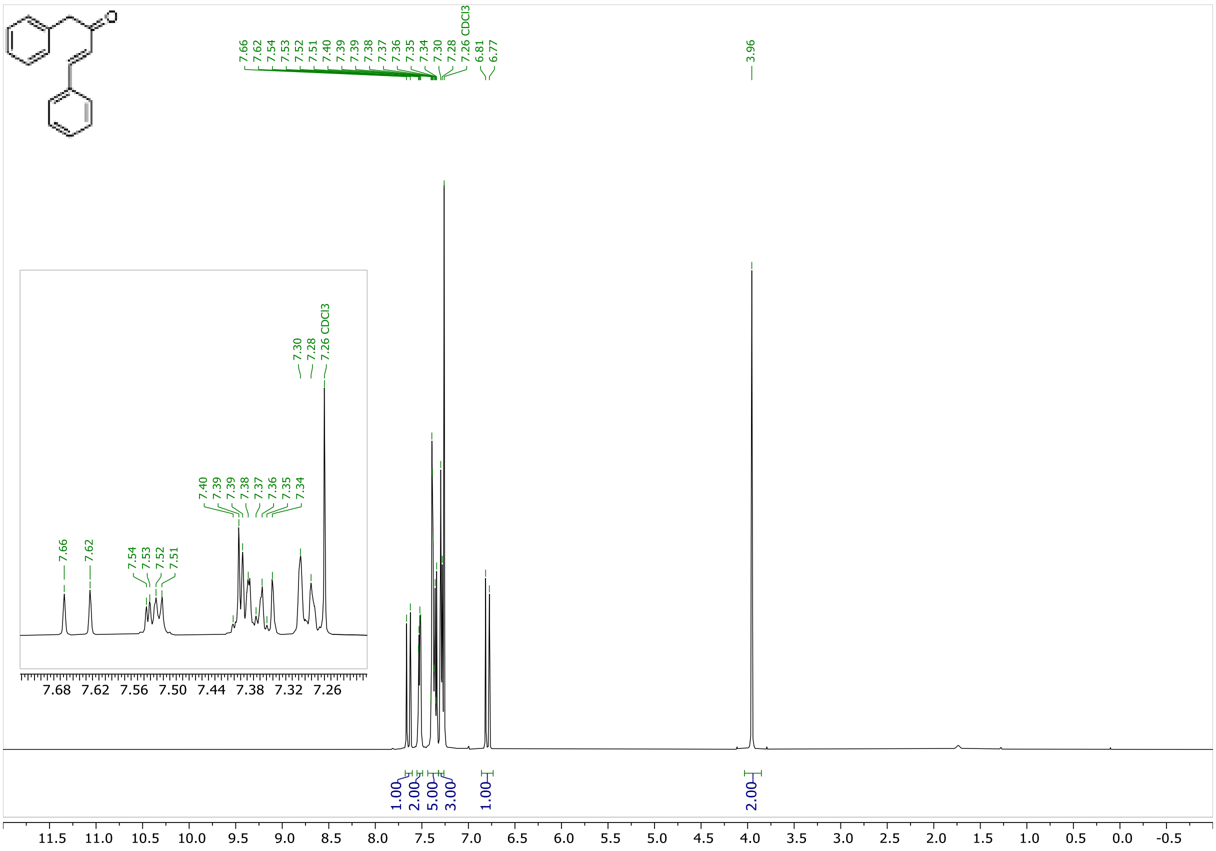

4p, ^{13}C{^{1}H} NMR (101 MHz, $CDCl_3$)

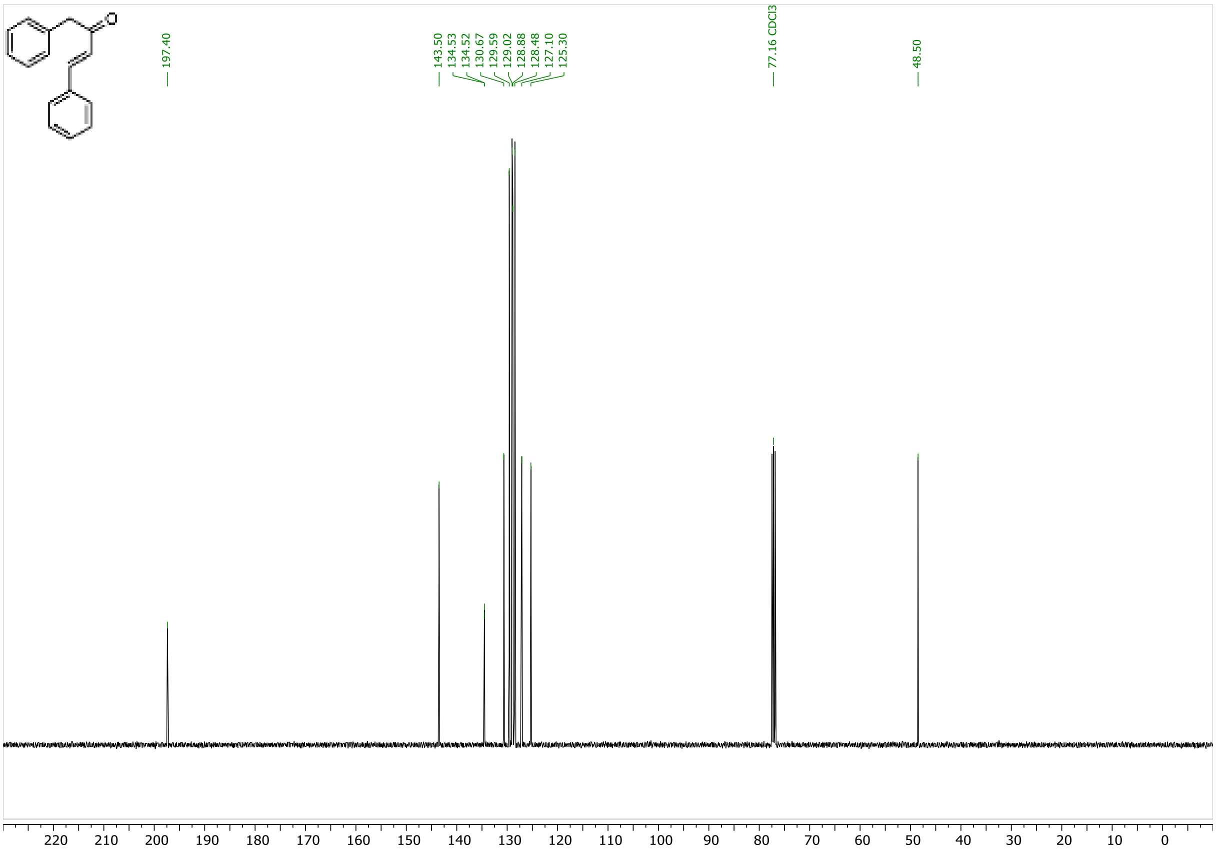

4q, ^{1}H NMR (400 MHz, $CDCl_3$)

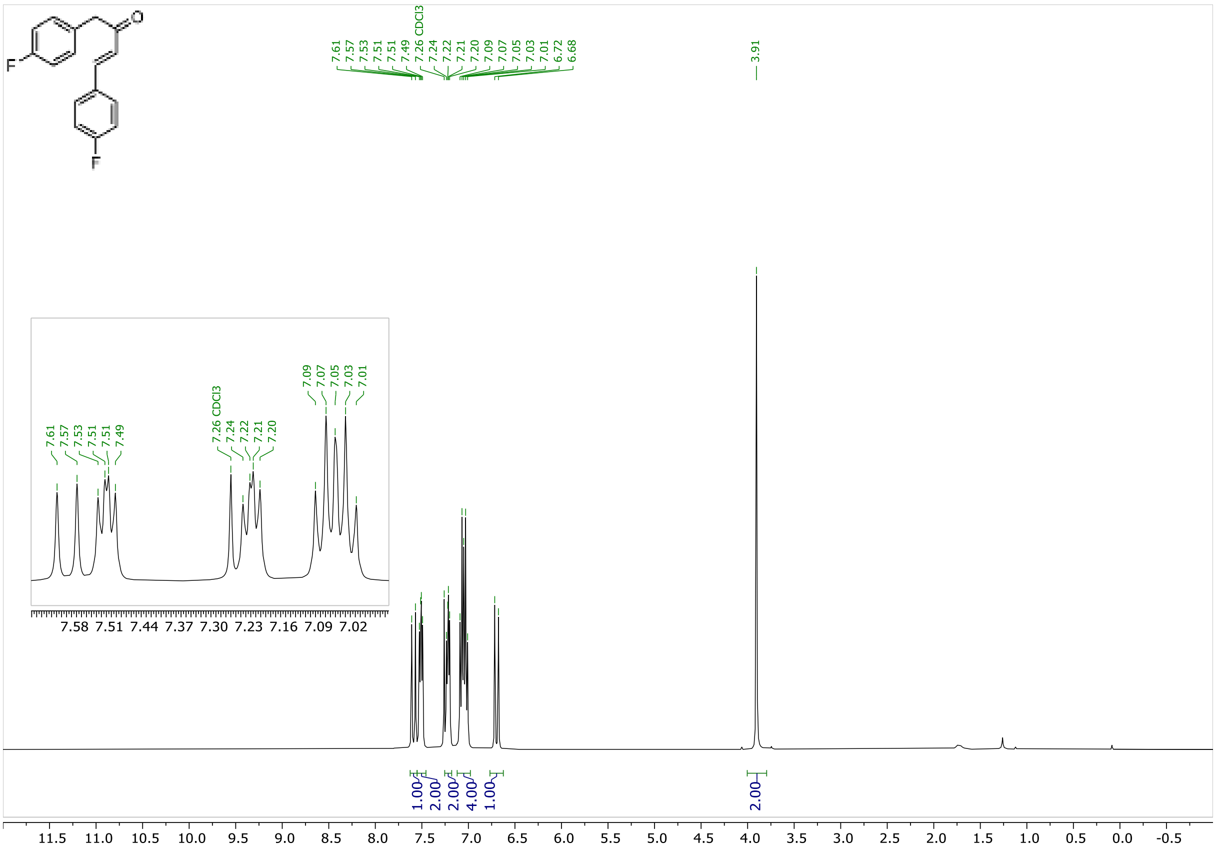

4q, $^{13}C\{^1H\}$ NMR (101 MHz, $CDCl_3$)

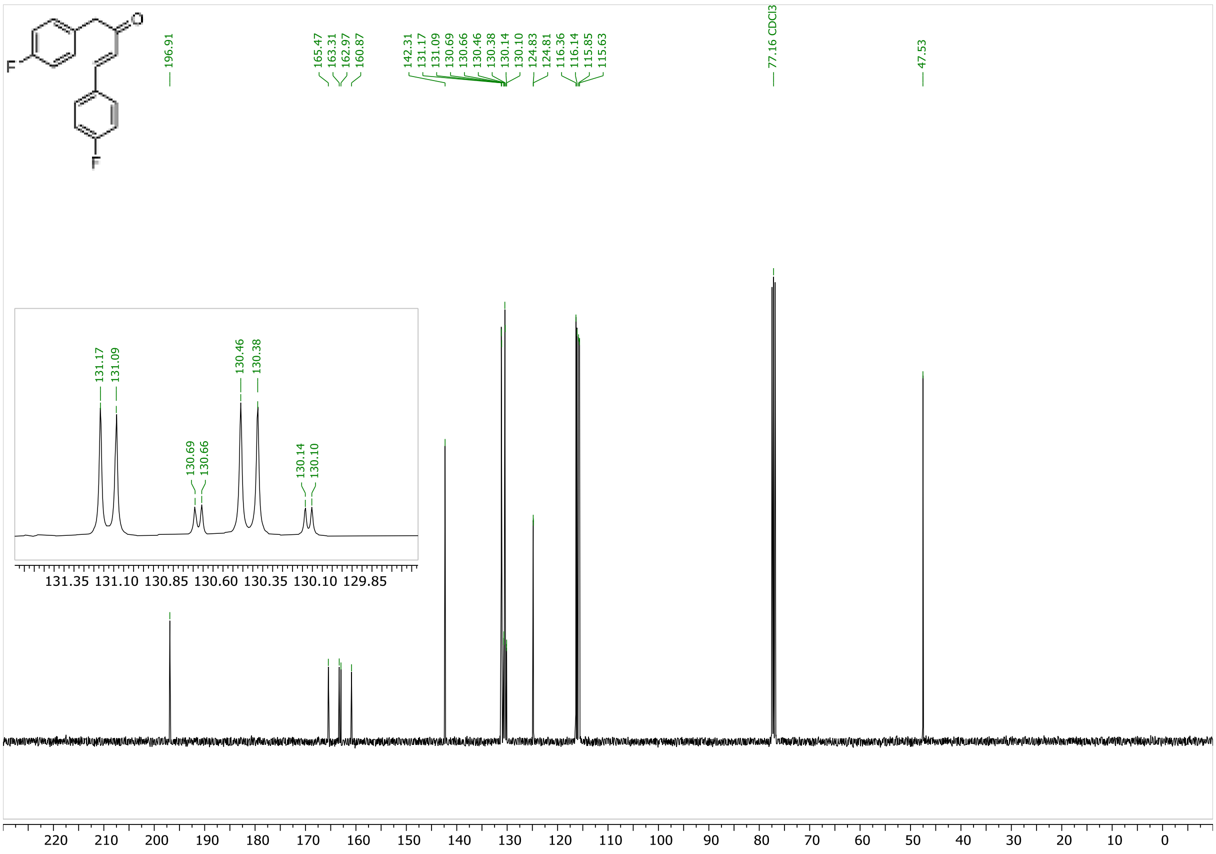

4r, ^{1}H NMR (400 MHz, $CDCl_3$)

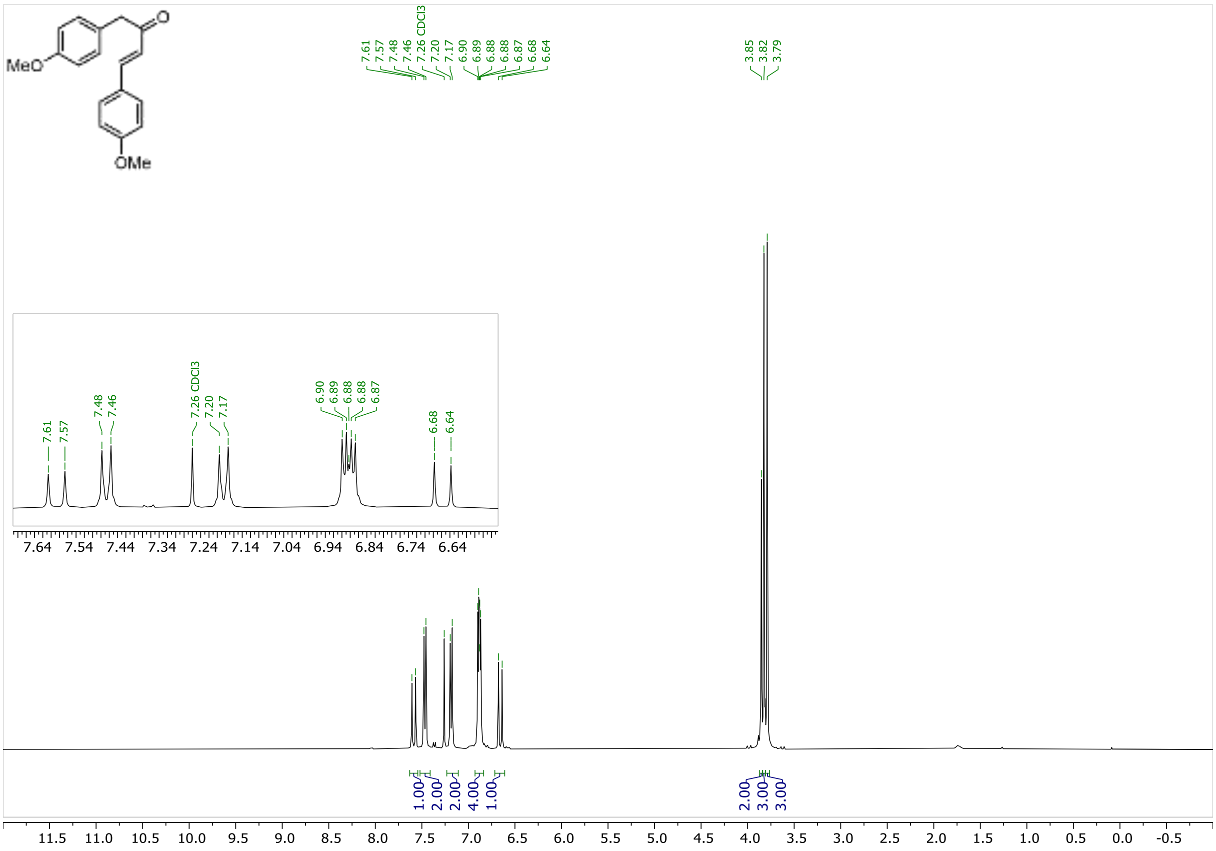

4r, $^{13}C\{^{1}H\}$ NMR (101 MHz, $CDCl_3$)

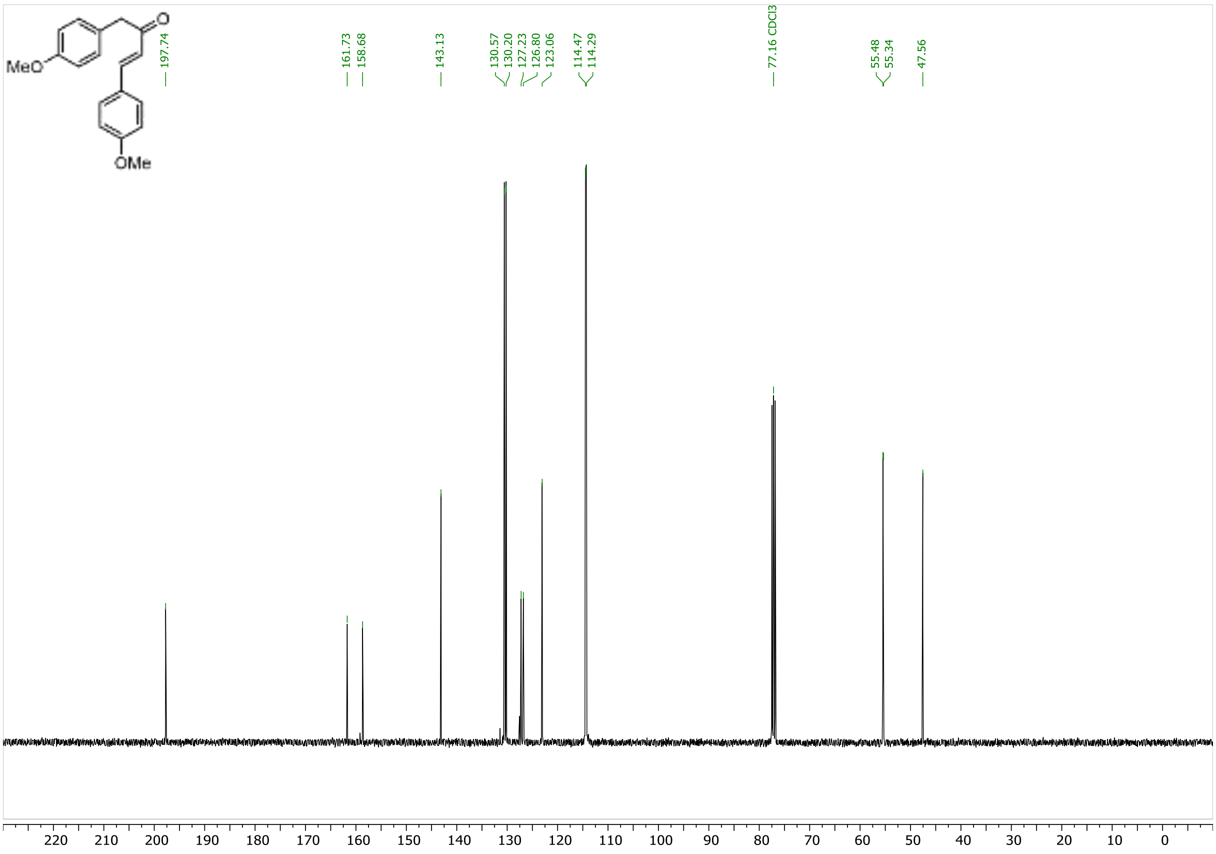

5a, ^{1}H NMR (400 MHz, CDCl$_3$)

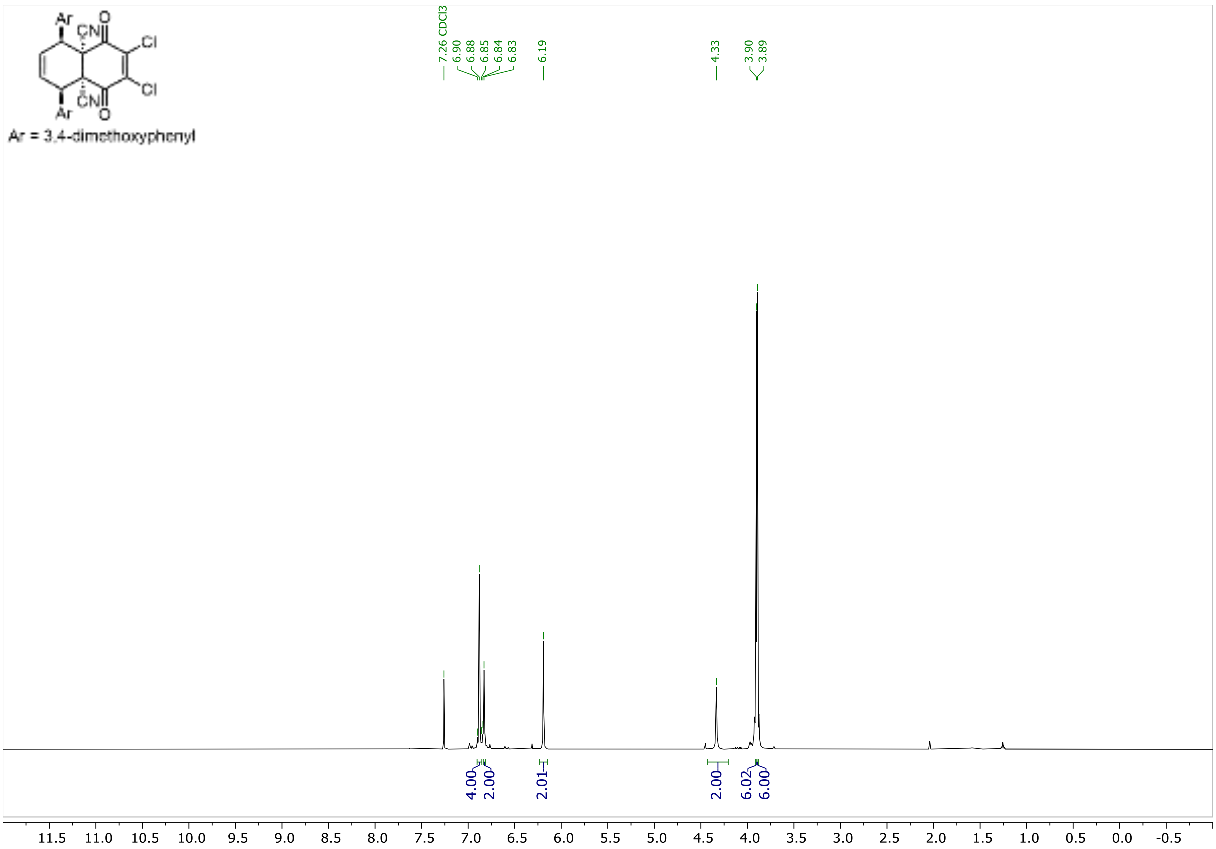

5a, ^{13}C{^{1}H} NMR (101 MHz, CDCl$_3$)

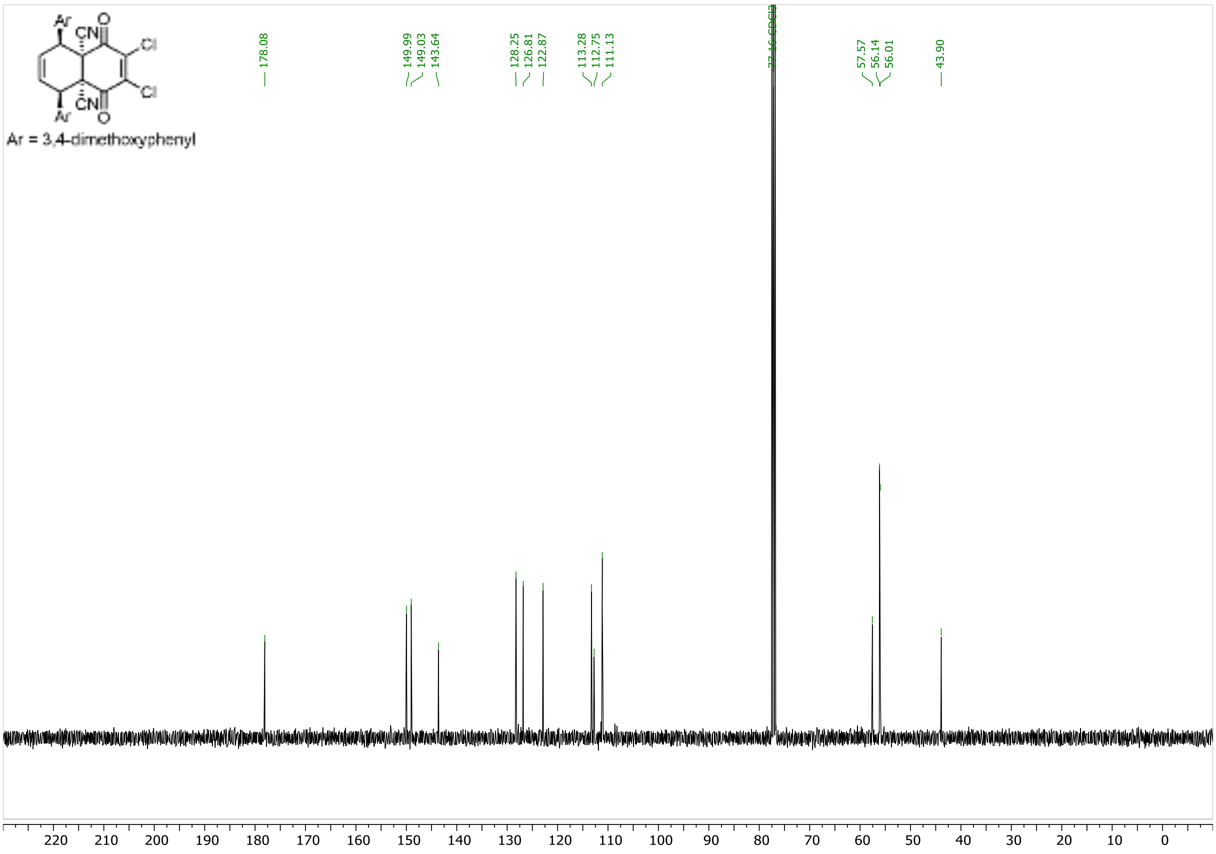

6a, ^{1}H NMR (400 MHz, $CDCl_3$)

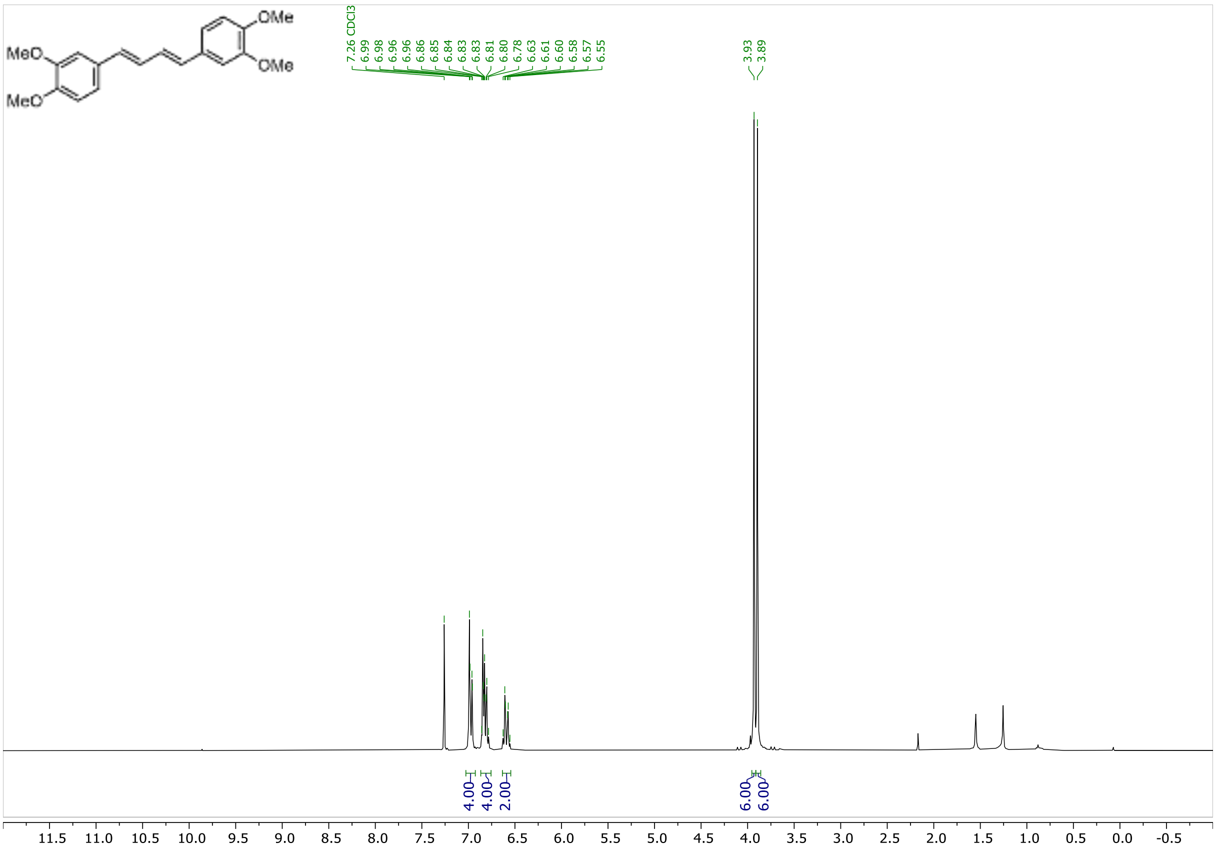

6a, ^{13}C{^{1}H} NMR (101 MHz, $CDCl_3$)

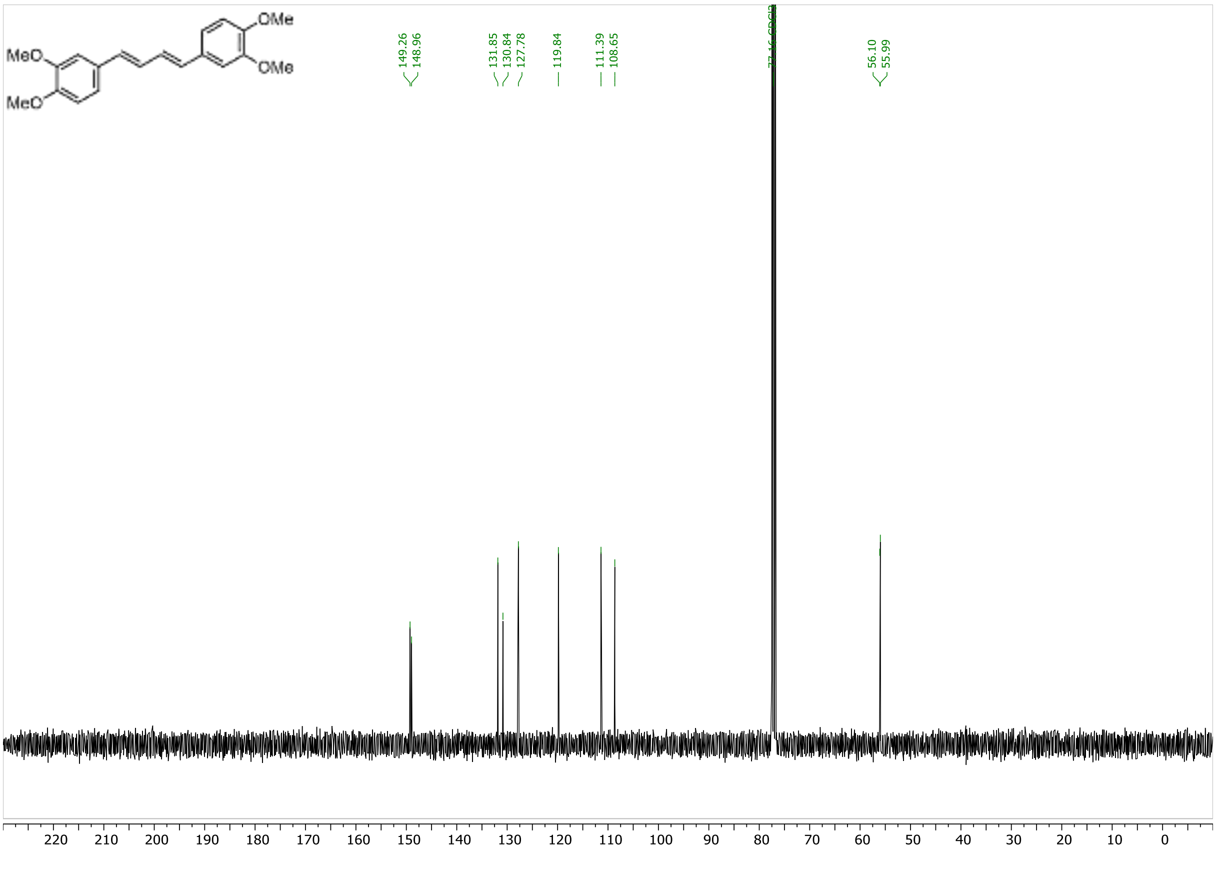

7a, ^{1}H NMR (400 MHz, $CDCl_3$)

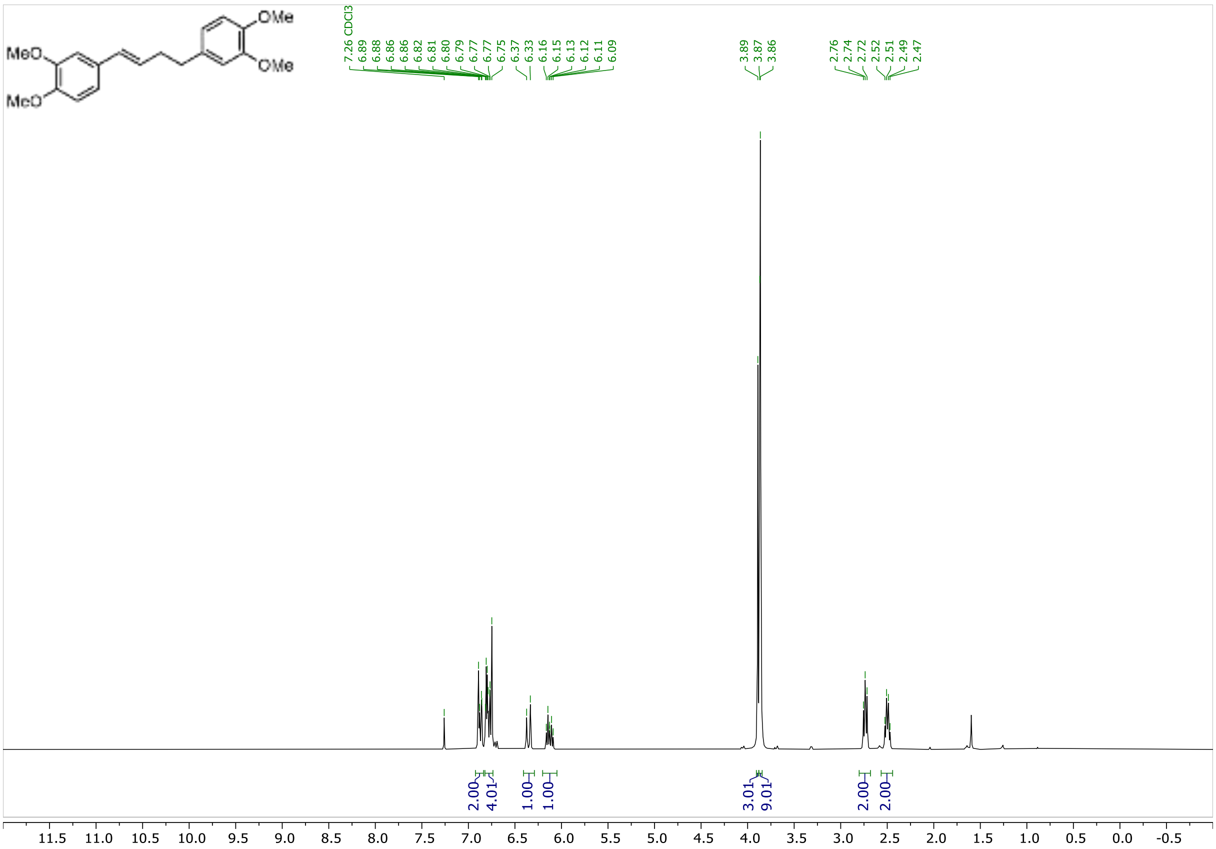

7a, $^{13}C\{^{1}H\}$ NMR (101 MHz, $CDCl_3$)

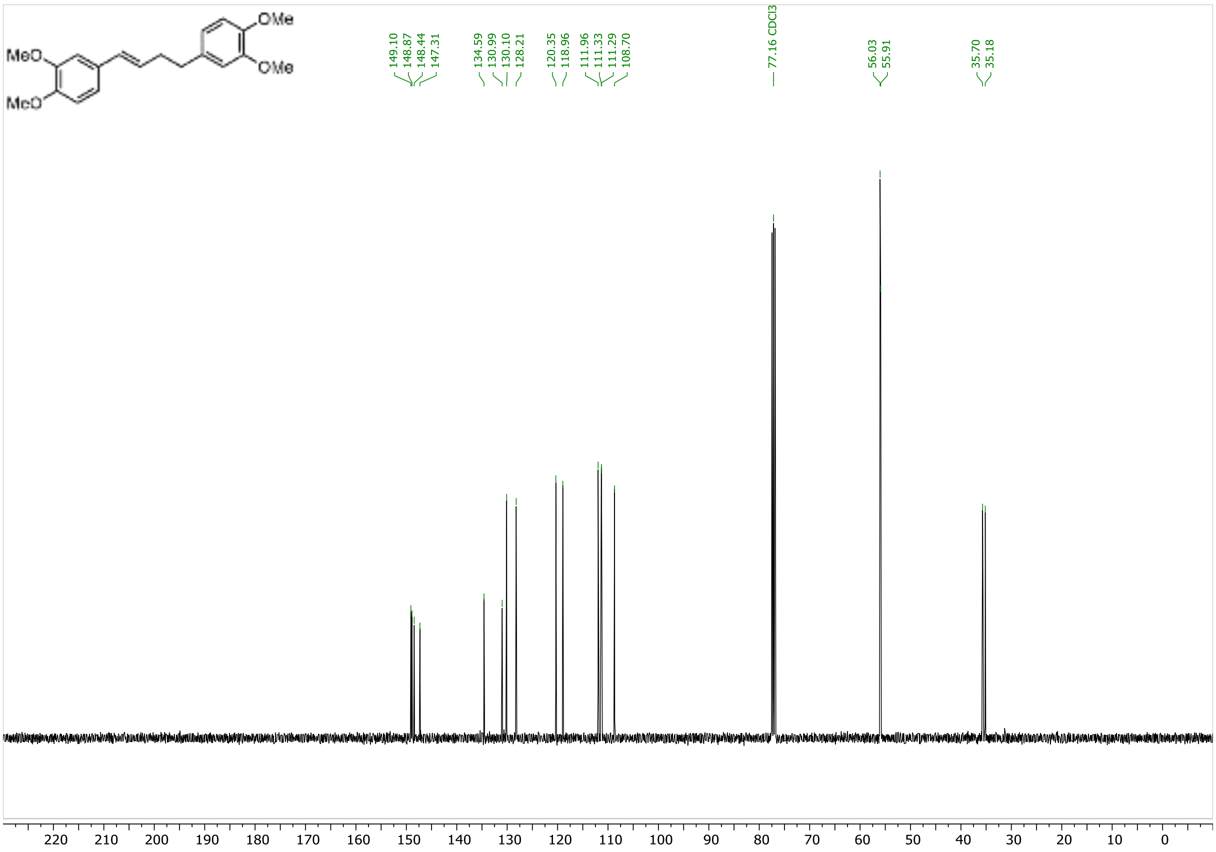

8, ^{1}H NMR (400 MHz, $CDCl_3$)

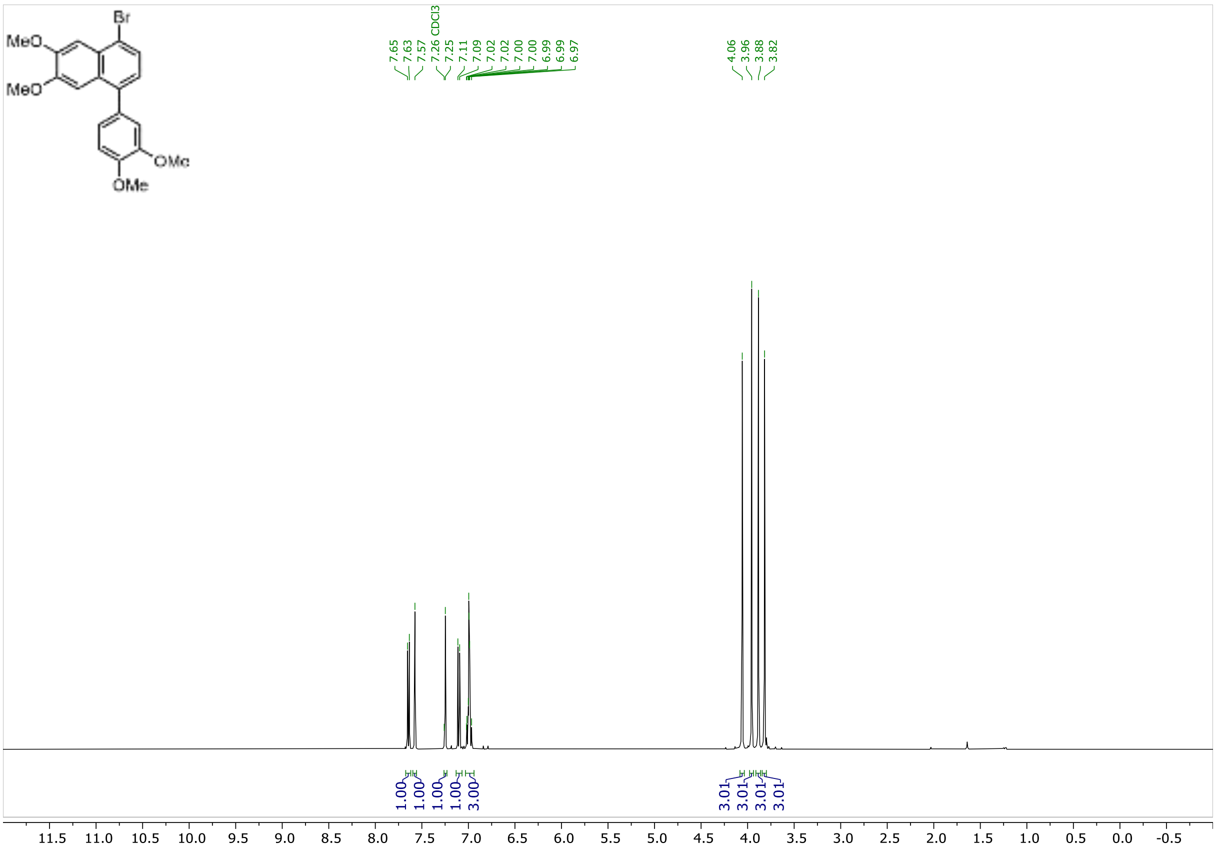

8, ^{13}C{^{1}H} NMR (101 MHz, $CDCl_3$)

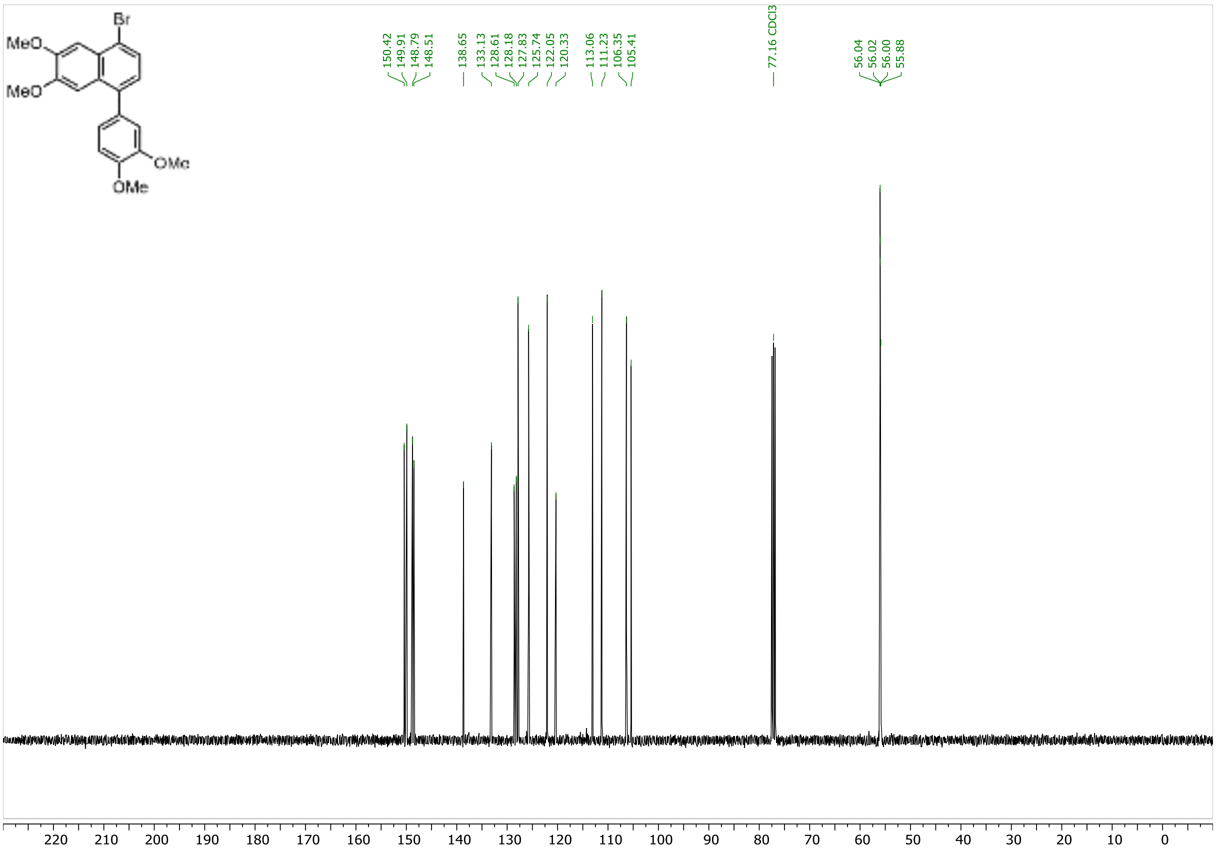

9, ^{1}H NMR (400 MHz, $CDCl_3$)

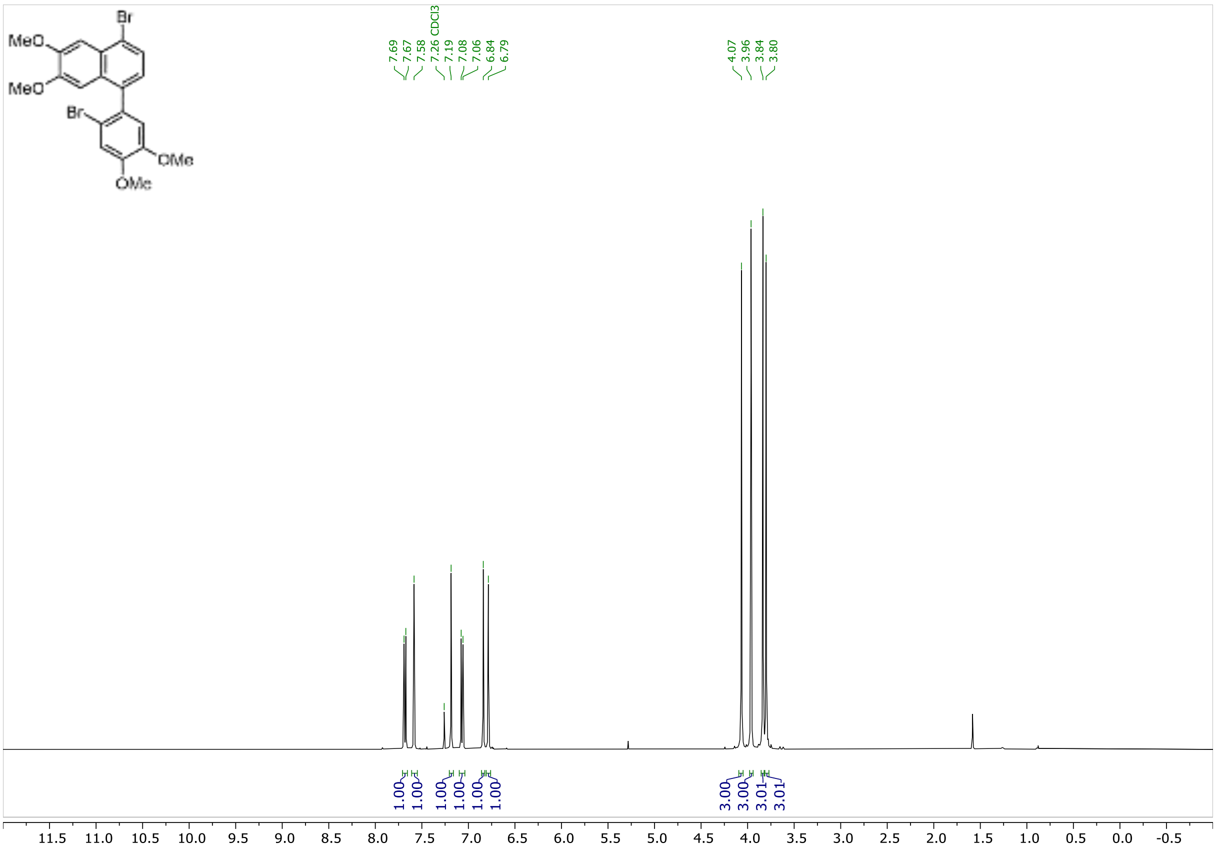

9, ^{13}C{^{1}H} NMR (101 MHz, $CDCl_3$)

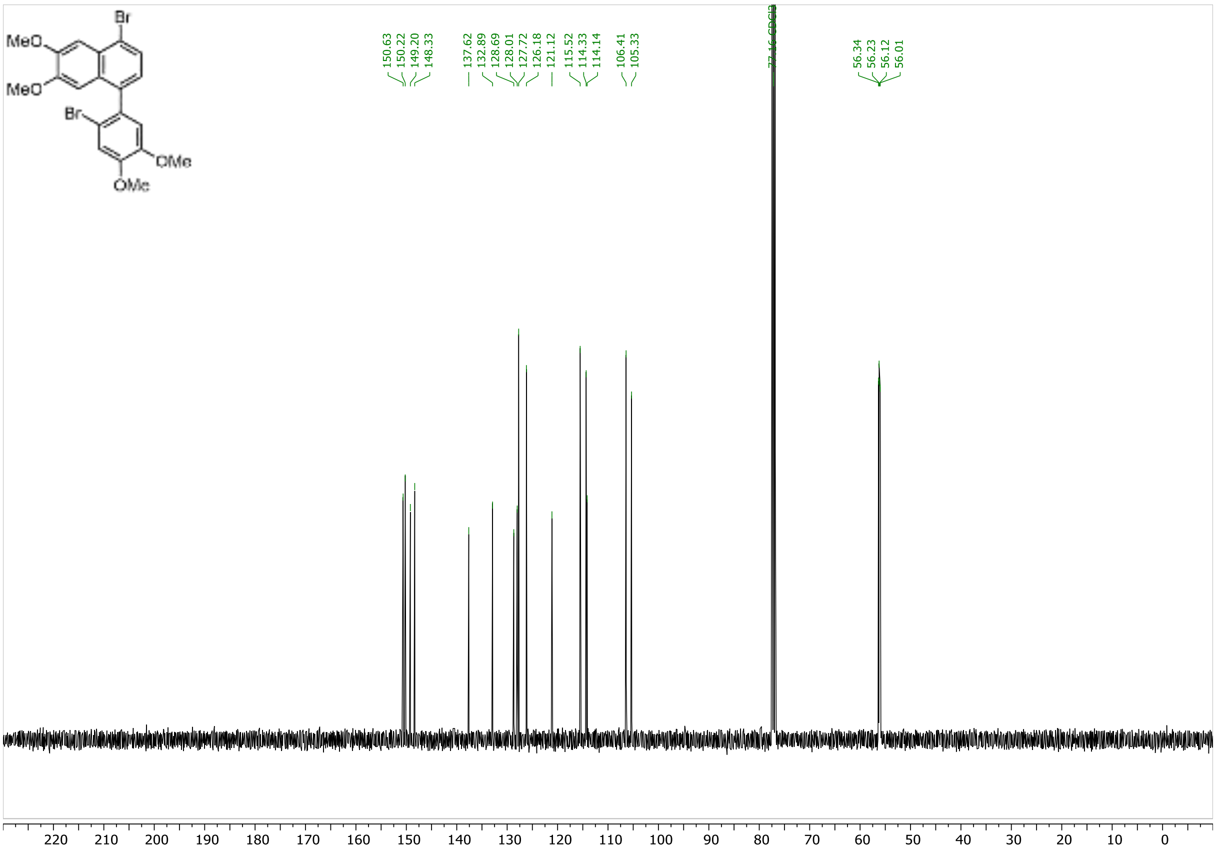

10, ^{1}H NMR (400 MHz, $CDCl_3$)

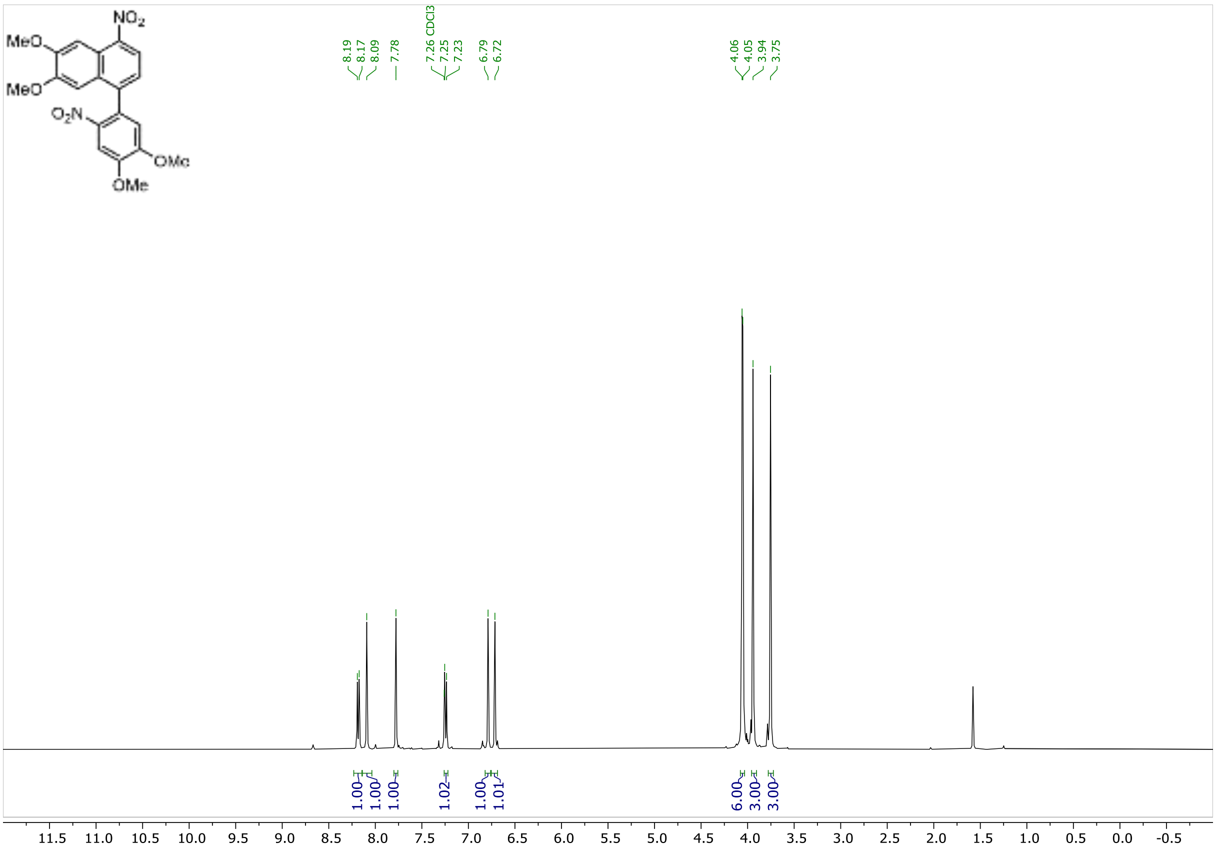

10, ^{13}C{^{1}H} NMR (101 MHz, $CDCl_3$)

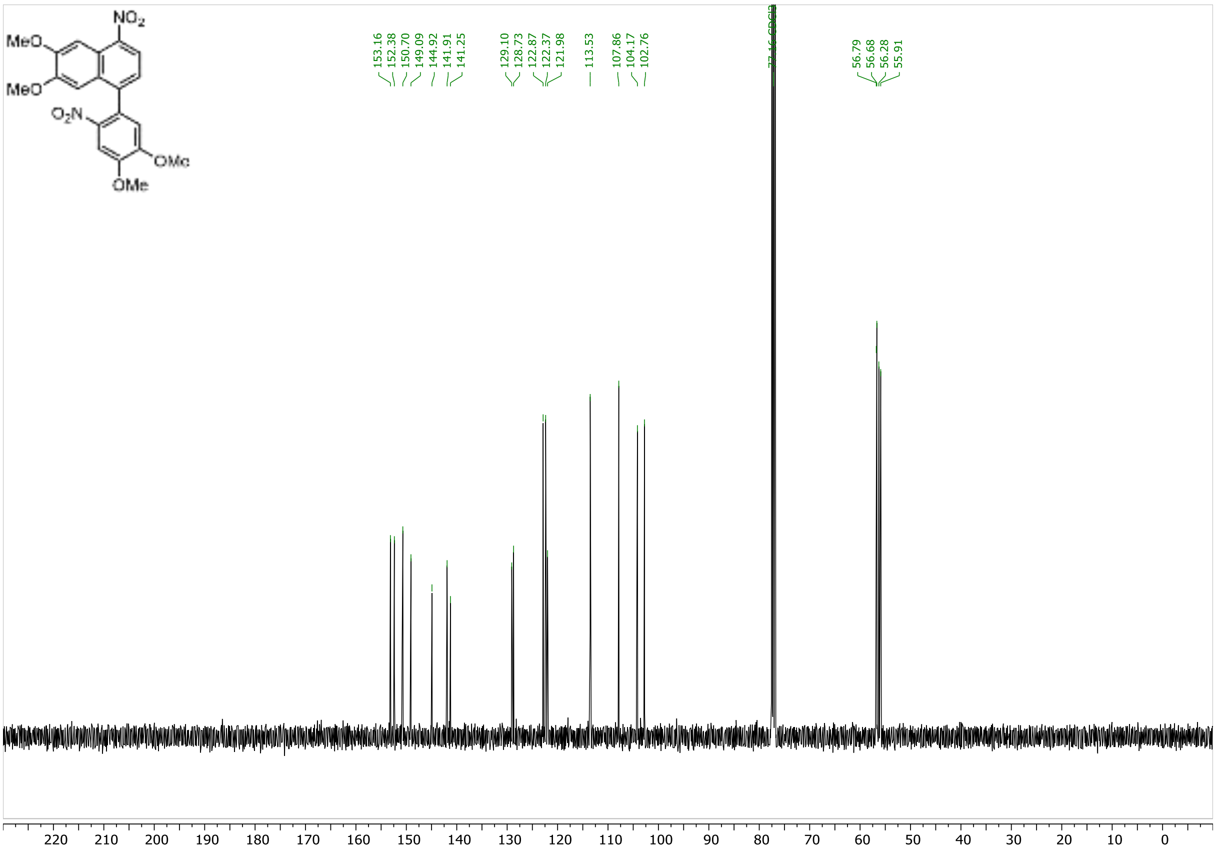

11, ^{1}H NMR (400 MHz, $CDCl_3$)

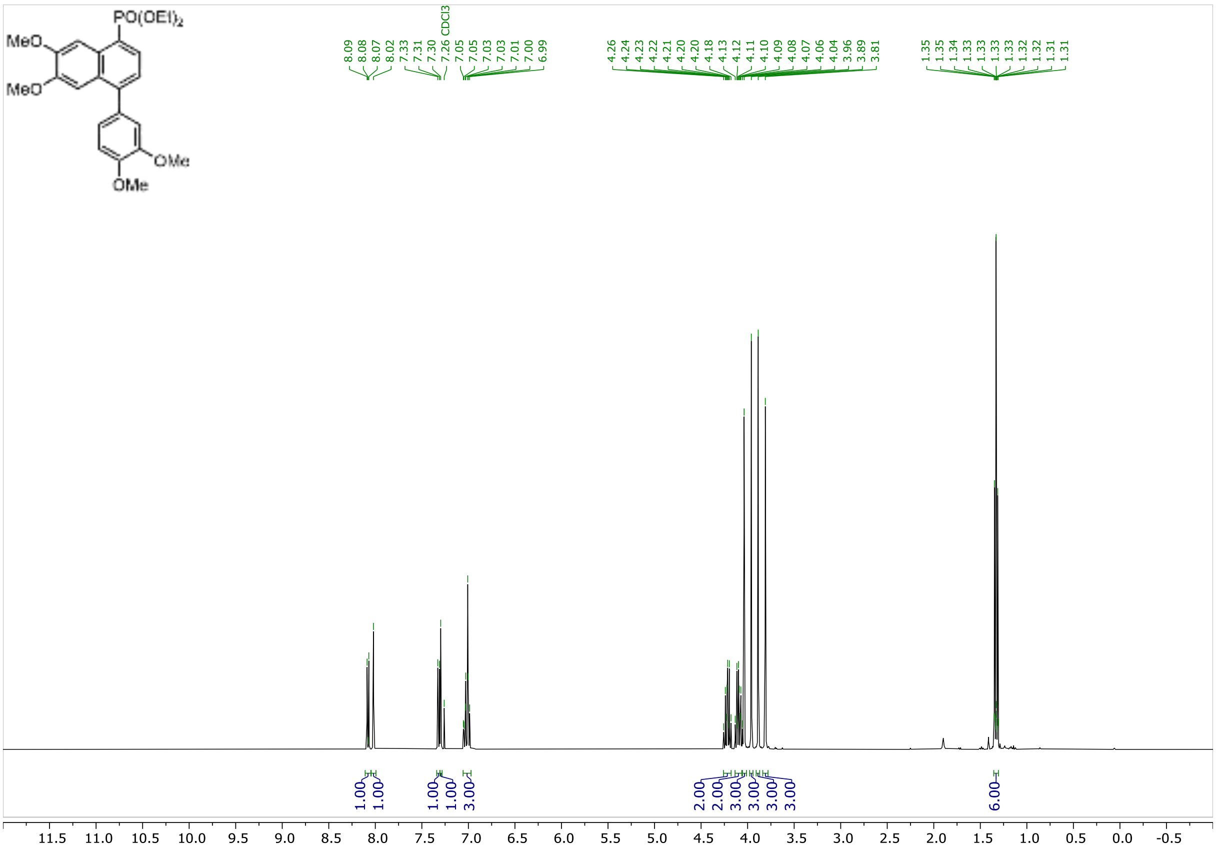

11, $^{13}C\{^1H\}$ NMR (101 MHz, $CDCl_3$)

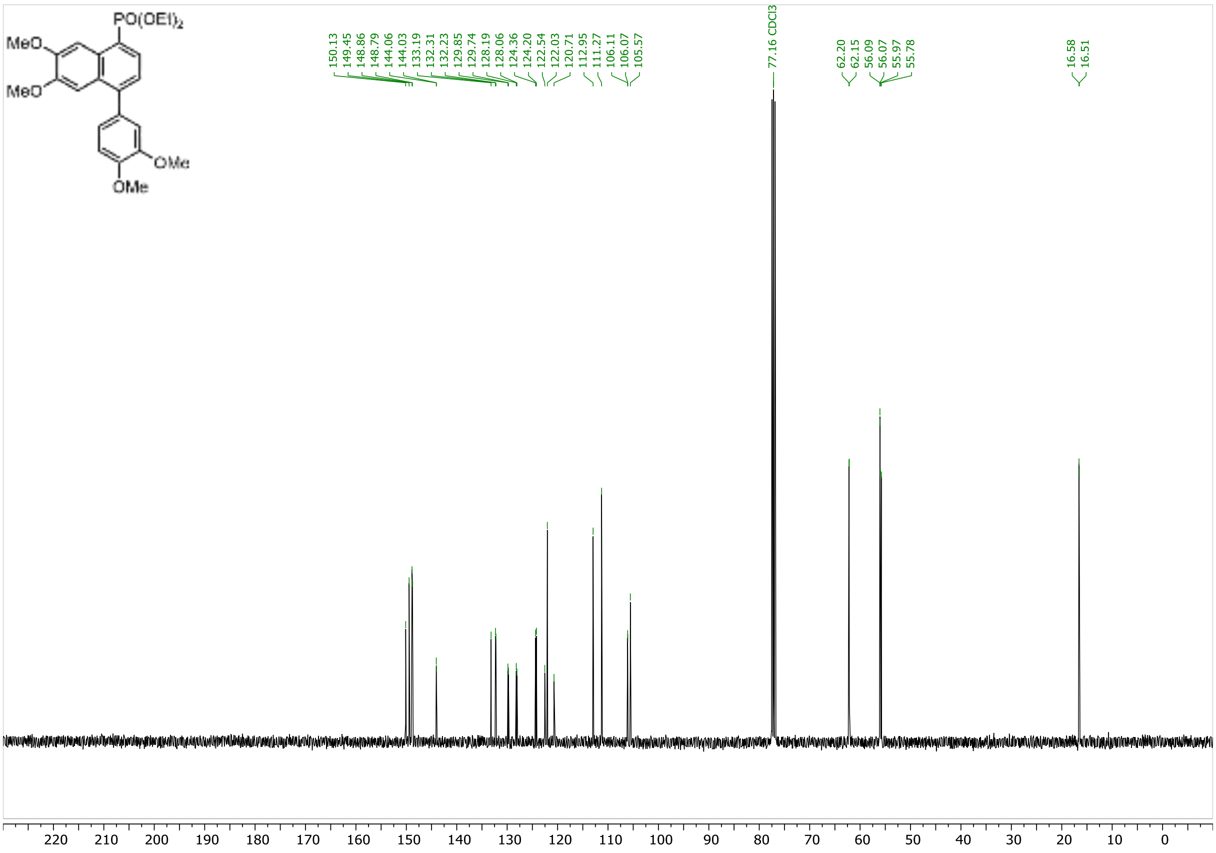

11, ^{31}P NMR (162 MHz, $CDCl_3$)

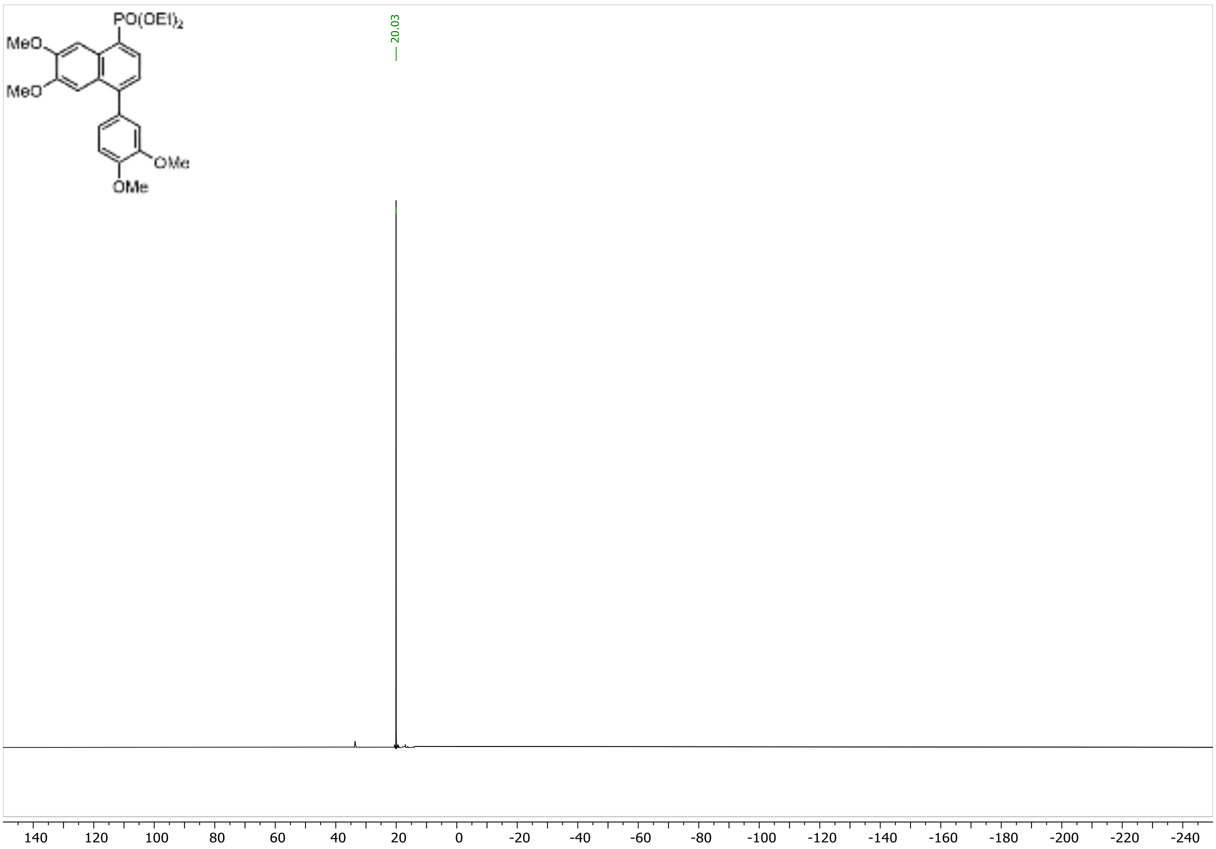

12, ^{1}H NMR (400 MHz, $CDCl_3$)

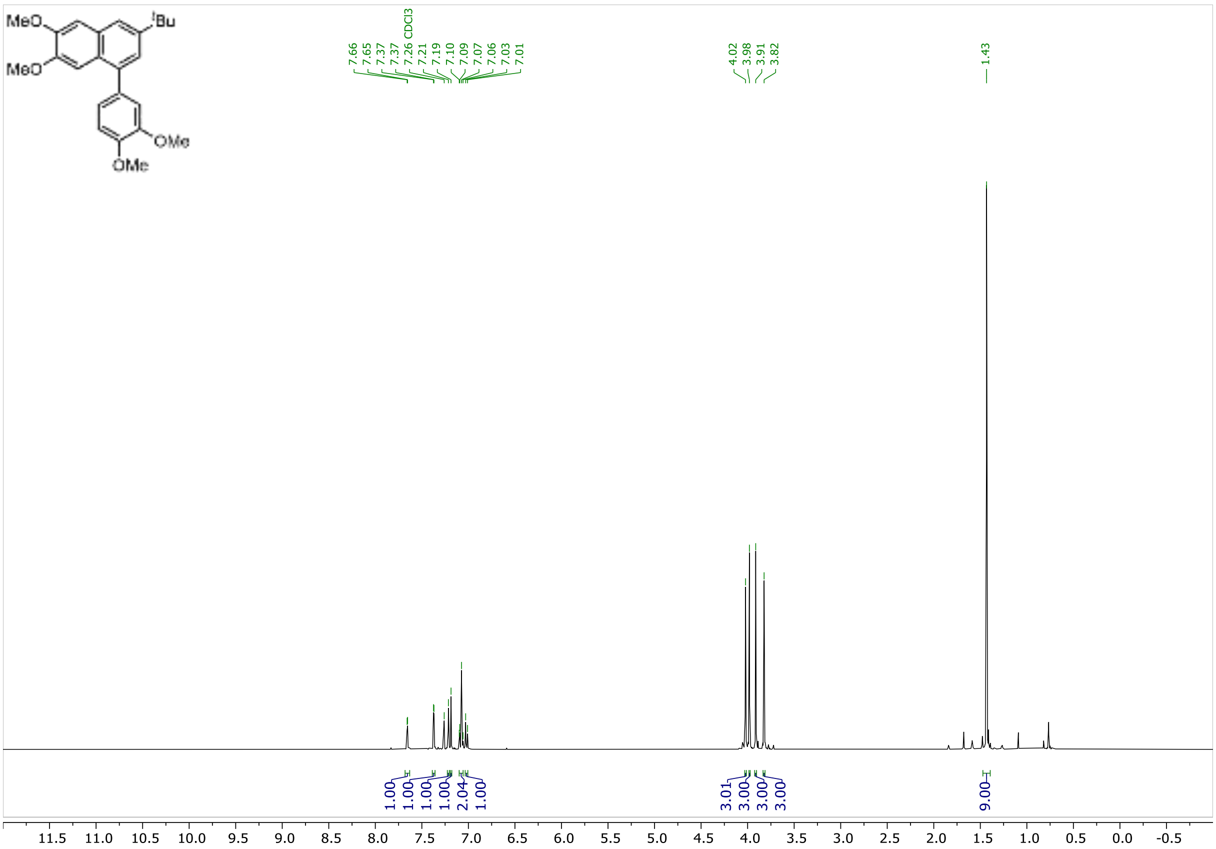

12, ^{13}C{^{1}H} NMR (101 MHz, $CDCl_3$)

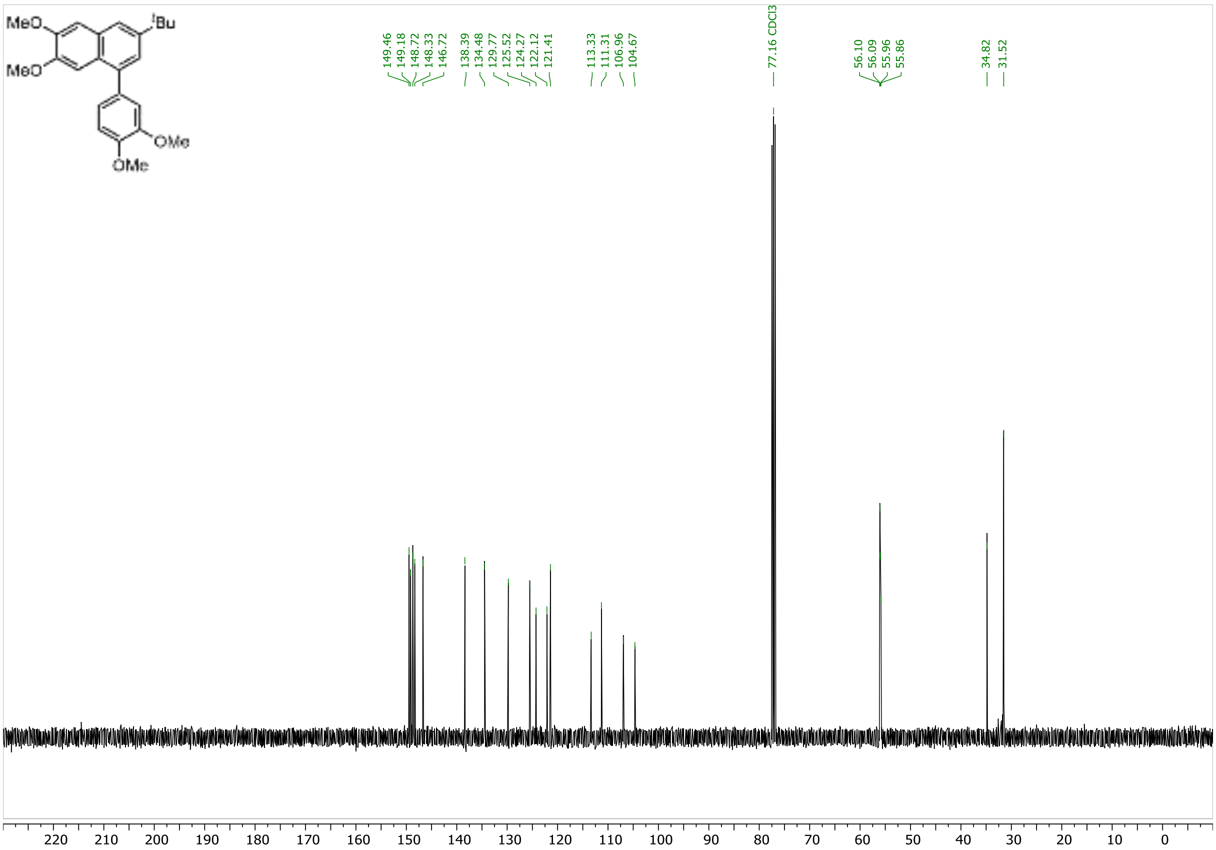

13, ^{1}H NMR (400 MHz, $CDCl_3$)

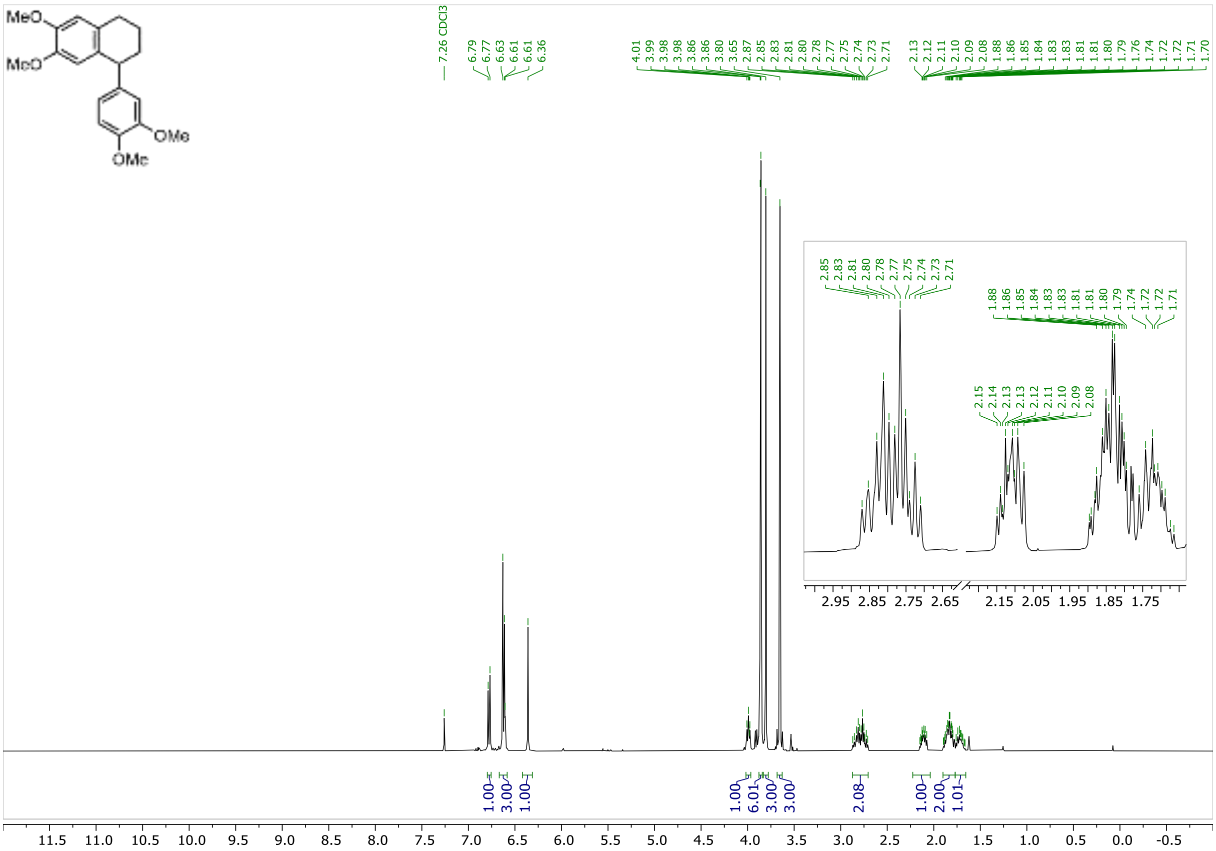

13, ^{13}C{^{1}H} NMR (101 MHz, $CDCl_3$)

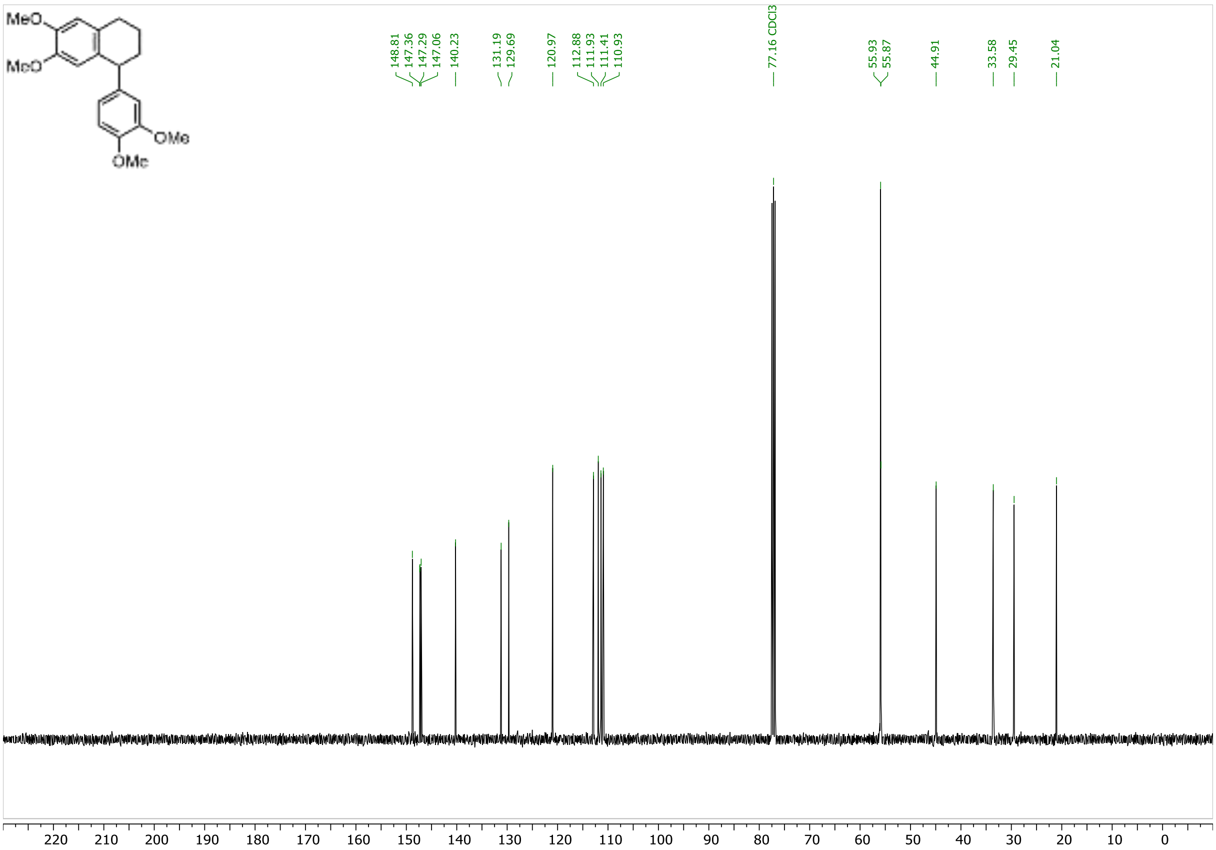

14, ^{1}H NMR (400 MHz, DMSO-d_6)

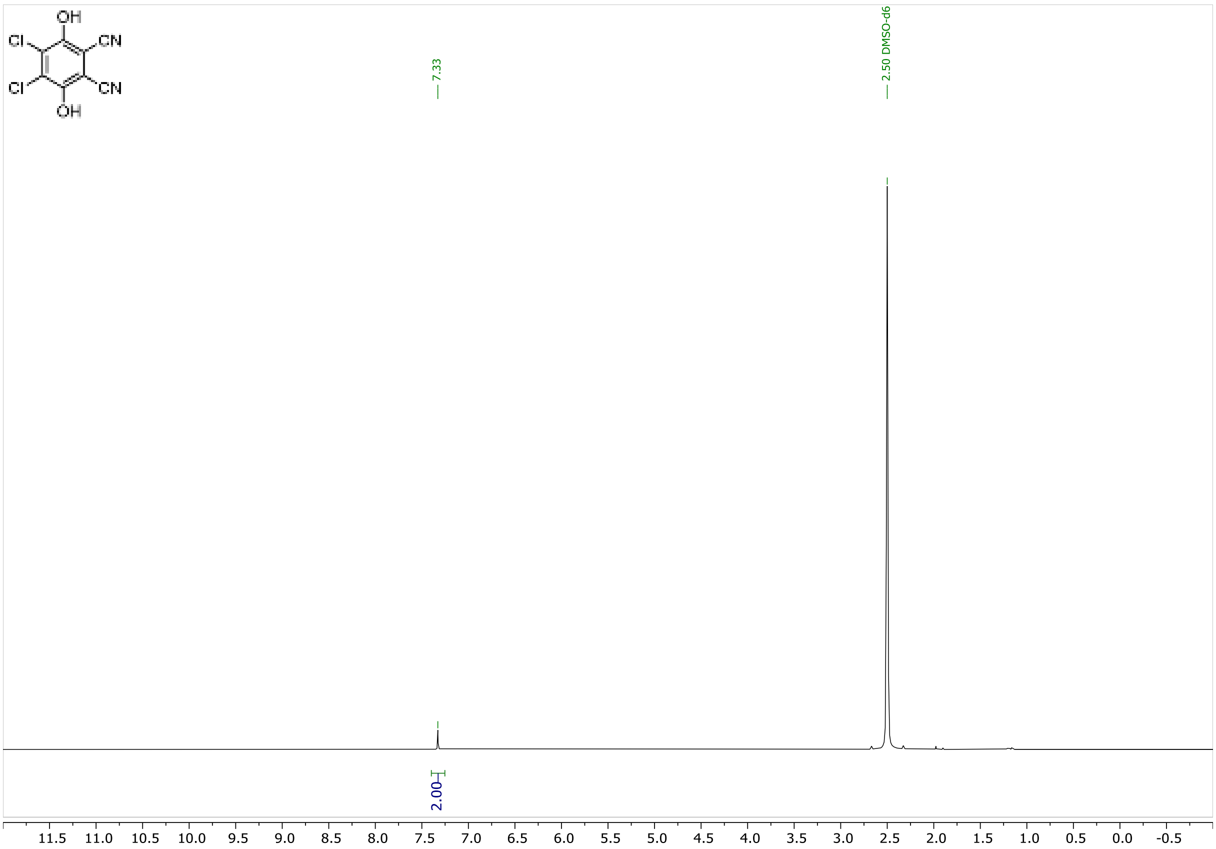

14, ^{13}C{^{1}H} NMR (101 MHz, DMSO-d_6)

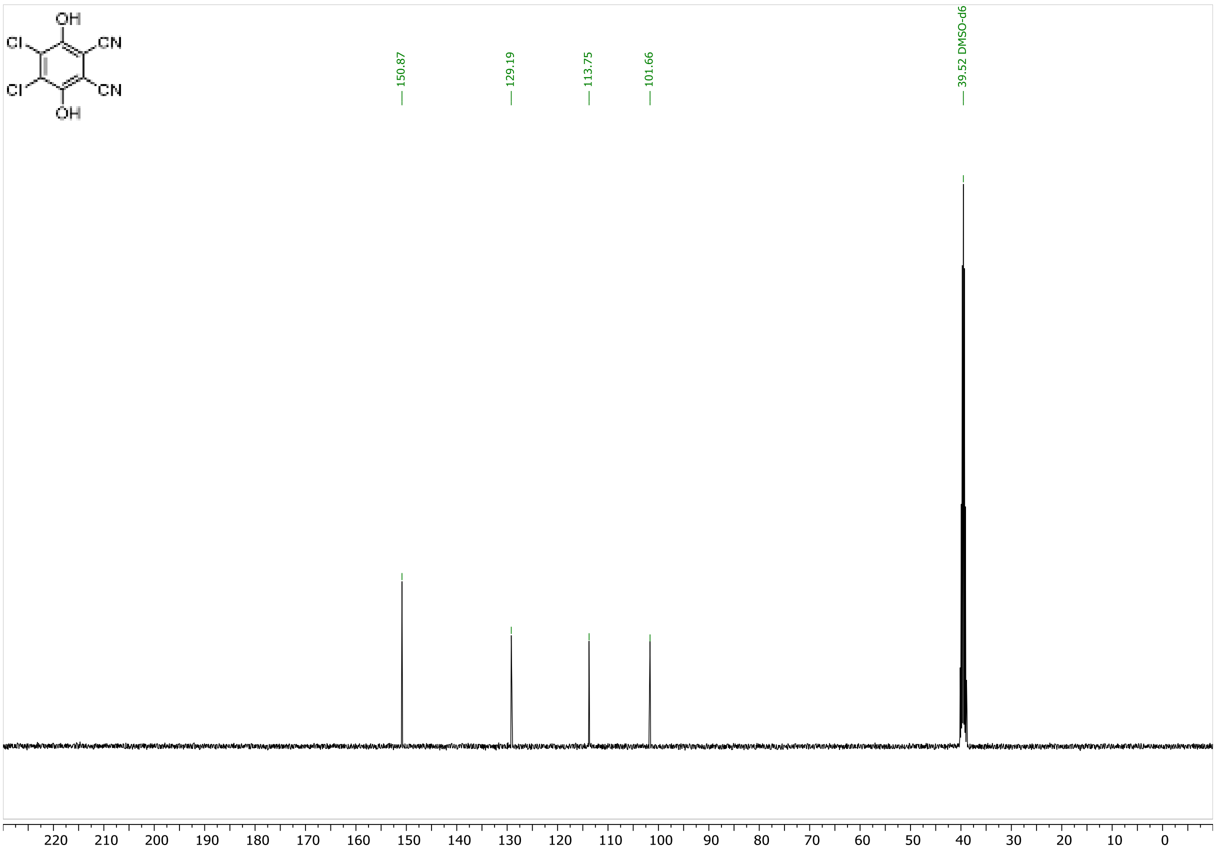

Supplement: Supplementary file 1 [file jo5c02949_si_001.pdf]
